# Supramolecular Binding of Phosphonate Dianions by Nanojars and Nanojar Clamshells

**DOI:** 10.1021/acs.inorgchem.4c02386

**Published:** 2024-07-18

**Authors:** Pooja Singh, Matthias Zeller, Gellert Mezei

**Affiliations:** † Department of Chemistry, Western Michigan University, Kalamazoo, Michigan 49008, United States; ‡ Department of Chemistry, Purdue University, West Lafayette, Indiana 47907, United States

## Abstract

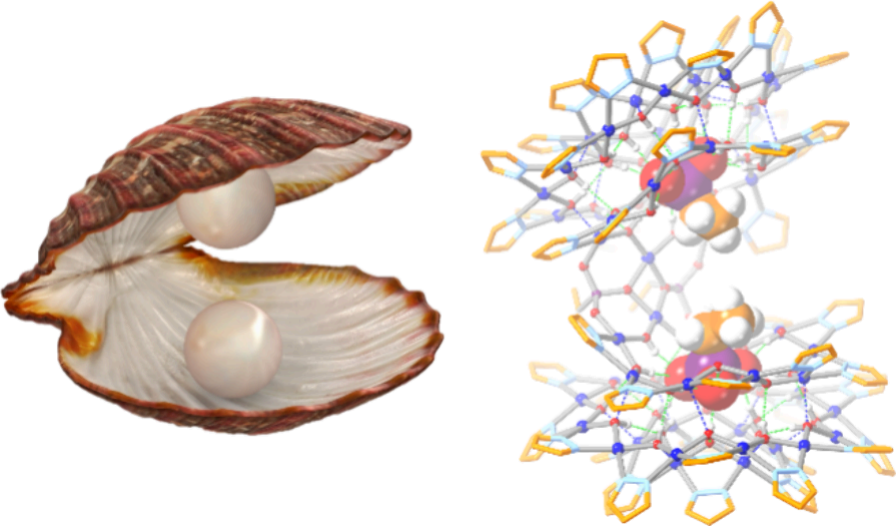

Despite the widespread use of phosphonates (RPO_3_^2–^) in various agricultural, industrial, and household applications and the ensuing eutrophication of polluted water bodies, the capture of phosphonate ions by molecular receptors has been scarcely studied. Herein, we describe a novel approach to phosphonate binding using chemically and thermally robust supramolecular coordination assemblies of the formula [RPO_3_⊂{*cis*-Cu^II^(μ-OH)(μ-pz)}_*n*_]^2–^ (Cu_*n*_; *n* = 27–31; pz = pyrazolate ion, C_3_H_3_N_2_^–^; R = aliphatic or aromatic group). The neutral receptors, termed nanojars, strongly bind phosphonate anions by a multitude of hydrogen bonds within their highly hydrophilic cavities. These nanojars can be synthesized either directly from their constituents or by depolymerization of [*trans*-Cu^II^(μ-OH)(μ-pz)]_∞_ induced by phosphonate anions. Electrospray-ionization mass spectrometry, UV–vis and variable-temperature, paramagnetic ^1^H and ^31^P NMR spectroscopy, single-crystal X-ray diffraction, along with chemical stability studies toward NH_3_ and Ba^2+^ ions, and thermal stability studies in solution are employed to explore the binding of various phosphonate ions by nanojars. Crystallographic studies of 12 different nanojars offer unprecedented structural characterization of host–guest complexes with doubly charged RPO_3_^2–^ ions and reveal a new motif in nanojar chemistry, nanojar clamshells, which consist of phosphonate anion-bridged pairs of nanojars and double the phosphonate-binding capacity of nanojars.

## Abstract 2

The binding of phosphonate anions bearing different aliphatic and aromatic groups by nanojars and nanojar clamshells has been investigated using mass spectrometry, UV−vis and variable-temperature, paramagnetic ^1^H and ^31^P NMR spectroscopy, X-ray crystallography, and chemical stability studies.

## Introduction

Phosphonates (RPO_3_^2–^) are organophosphorus compounds derived from the phosphate ion (PO_4_^3–^) by replacement of one O atom with an organic group (R).^1^ They can also be viewed as derivatives of deprotonated phosphonic (phosphorous) acid, HPO_3_^2–^, although the oxidation state of the phosphorus atom in the latter is 3+ as opposed to 5+ in phosphonates and phosphates. Phosphonates are encountered in nature and are involved in physiological processes, the global phosphorus cycle and the biogeochemical generation of methane.^2,3^ A large variety of industrial, agricultural and household activities also employ phosphonates, in applications including pesticides (e.g., glyphosate), plant growth regulators, detergents, bleach stabilizers, water softening and desalination agents, corrosion inhibitors, antiscalants, concrete retarders, metal extracting agents, as well as inhibitors of enzymes that utilize phosphate or pyrophosphate as a substrate (treatment of osteoporosis, antibiotic and antiviral medications).^4−8^ Phosphonates are also actively studied for different novel applications, such as porous materials for gas storage and catalysis, magnetic and luminescent materials, proton conduction, and prodrugs.^9−13^ Phosphonate esters, such as dimethyl methylphosphonate, are used as flame retardants among various other applications.^14^

Phosphonates are less hydrophilic than phosphate. Nevertheless, they are water-soluble, and despite their low toxicity to aquatic organisms,^15^ their accumulation in water bodies has harmful effects on the environment and contributes to eutrophication.^16,17^ Therefore, the removal of phosphonates from aqueous environments is desirable.^18,19^ Furthermore, phosphorus is a strategic element (phosphate rock is a nonrenewable, finite resource), and its recovery is becoming increasingly important.^20−23^ While several supramolecular receptors for phosphates have been developed,^24−28^ the supramolecular binding of phosphonates is much less studied and only a few receptors are known. These include a tripodal 8-aminochromenone-2-carboxamide-based receptor,^29^ a generation 5 poly(amidoamine) (PAMAM) dendrimer-based sensing array^30^ and fluorescent sensors based on a tripodal receptor with H-bond donor thiourea and/or pyrrole-2-yl-amide appendages.^31^ Receptors for singly charged hydrogenphosphonates, including bis(hydrogenphosphonates) based on aminocoumarin,^32^ anthracene- and 1,8-diphenylnaphthalene-diamidine or -diguanidine,^33^ and cyanostar macrocycles have also been reported,^34^ as well as receptors for neutral phosphonate esters.^35^

Structural characterization of supramolecular host–guest complexes of monophosphonates is even scarcer. In fact, no crystal structure of a supramolecular (without metal-phosphonate coordination) host–guest complex of a fully deprotonated, simple phosphonate anion is known to date. The only reported crystal structure of a monophosphonate supramolecular host–guest complex in the Cambridge Structural Database (CSD)^36^ is for protonated phenylphosphonate in an amide-functionalized cyclodextrin receptor.^37^

**Figure 1:**
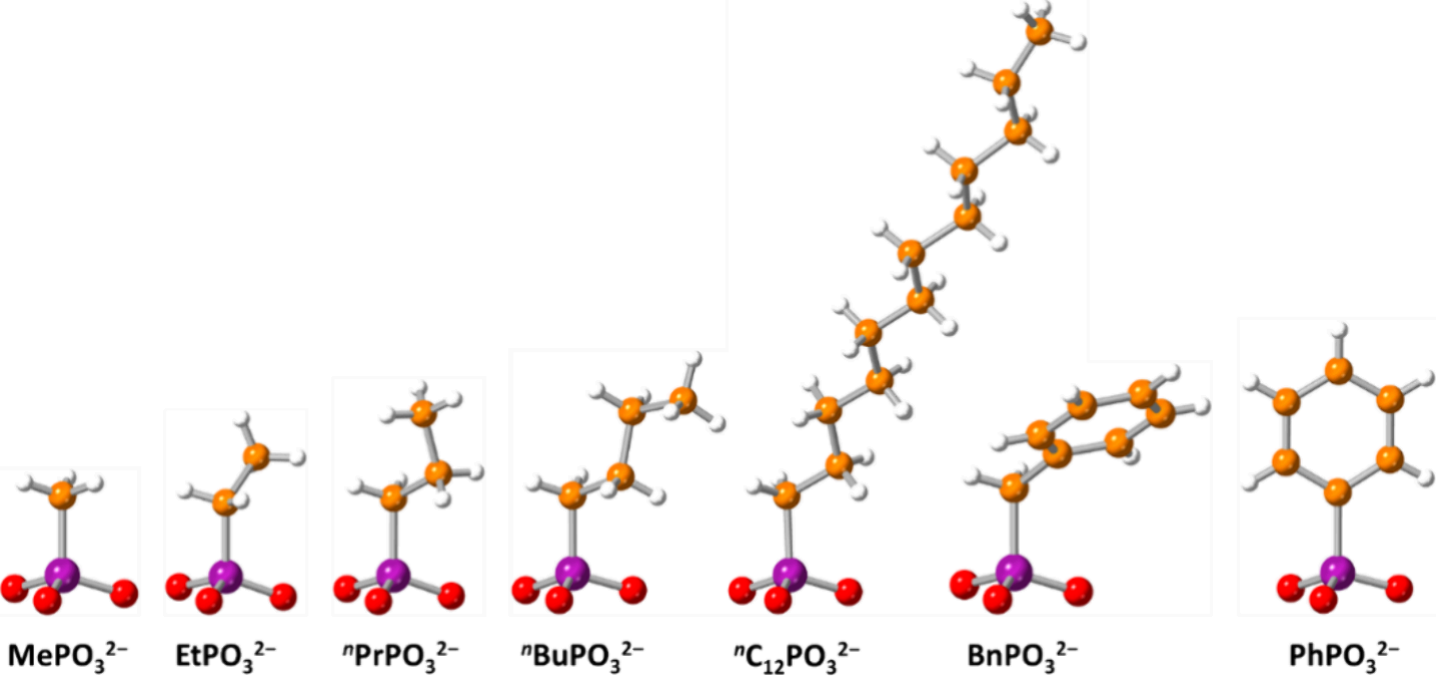
Ball-and-stick representation of the structure of methylphosphonate (MePO_3_^2–^), ethylphosphonate (EtPO_3_^2–^), *n*-propylphosphonate (^*n*^PrPO_3_^2–^), *n*-butylphosphonate (^*n*^BuPO_3_^2–^), *n*-dodecylphosphonate (^*n*^C_12_PO_3_^2–^), benzylphosphonate (BnPO_3_^2–^), and phenylphosphonate (PhPO_3_^2–^) anions. Color code: red, O; purple, P; orange, C; white, H.

We recently provided the first examples of supramolecular binding of phosphite (HPO_3_^2–^) based exclusively on H-bonding, using copper pyrazolate/hydroxide coordination complexes termed nanojars, [HPO_3_⊂{*cis*-Cu^II^(μ-OH)(μ-pz)}_*n*_]^2–^ (Cu_*n*_HPO_3_; *n* = 27–32), as receptors.^38^ Herein, we describe the synthesis of phosphonate nanojars with the formula [RPO_3_⊂{*cis*-Cu^II^(μ-OH)(μ-pz)}_*n*_]^2–^ (Cu_*n*_RPO_3_; *n* = 27–31; R = Me, Et, ^*n*^Pr, ^*n*^Bu, *n*-dodecyl (^*n*^C_12_), Bn, Ph; Figure 1) and report the first structural characterizations of monophosphonate dianions bound to a neutral supramolecular receptor exclusively by H-bonding, based on single-crystal X-ray diffraction. The solid-state crystallographic studies are complemented by solution-phase electrospray ionization mass spectrometric (ESI-MS), variable-temperature (VT), paramagnetic nuclear magnetic resonance (^1^H and ^31^P NMR), and UV–vis spectroscopic studies, along with chemical stability studies toward NH_3_ and Ba^2+^ ions and thermal stability studies in solution. We also report the crystal structures of the first nanojar clamshells, a novel motif in nanojar chemistry, which consist of two nanojar units tethered by two adjacent phosphonate units.

## Results and Discussion

### Synthesis and Mass Spectrometric Studies

**Figure 2:**
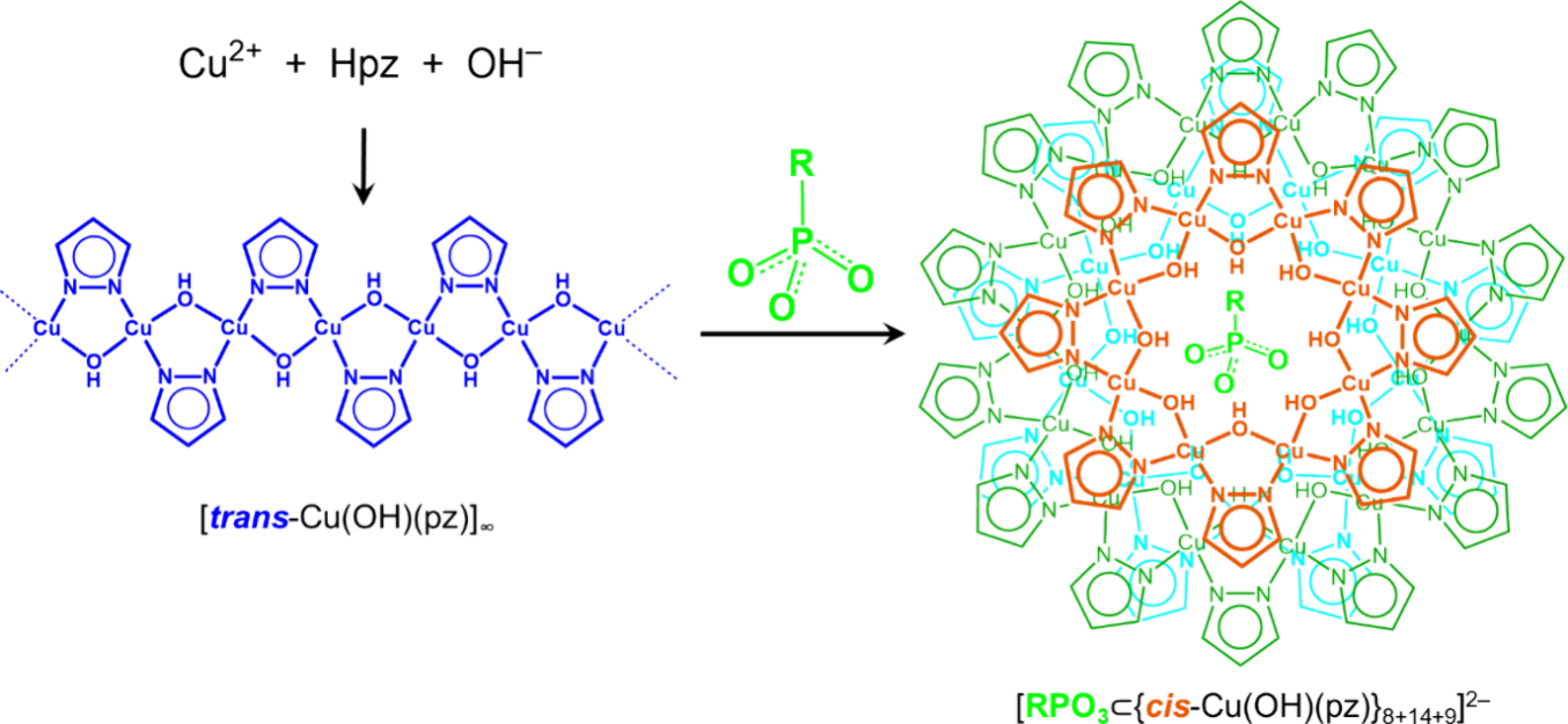
Schematic representation of the depolymerization of [*trans*-Cu(OH)(pz)]_∞_ into phosphonate-incarcerating nanojars [RPO_3_⊂{*cis*-Cu(OH)(pz)}_*n*_]^2–^ (R = Me, Et, ^*n*^Pr, ^*n*^Bu, ^*n*^C_12_, Bn, Ph; *n* = 27–31). Color code for *n* = 31 shown: persimmon, Cu_8_ ring; green, Cu_14_ ring; cyan, Cu_9_ ring.

Two different methods were employed for the synthesis of phosphonate nanojars. The first method involves a direct synthesis using Cu(NO_3_)_2_·2.5H_2_O, pyrazole, NaOH, Bu_4_NOH, and phosphonic acid (H_2_RPO_3_; R = Me, Et, ^*n*^Pr, ^*n*^Bu, ^*t*^Bu, *n*-dodecyl, Bn, Ph) in a 1:1:4:1 molar ratio in tetrahydrofuran (THF) at ambient temperature under an N_2_ atmosphere. Except in the case of R = ^*t*^Bu, a mixture of nanojars of the formula (Bu_4_N)_2_[RPO_3_⊂{*cis*-Cu^II^(μ-OH)(μ-pz)}_*n*_] (Cu_*n*_RPO_3_; *n* = 27–31) was obtained, with varying distributions of the different nanojar sizes. ESI-MS of these mixtures indicates that varying amounts of carbonate nanojars, (Bu_4_N)_2_[CO_3_⊂{*cis*-Cu^II^(μ-OH)(μ-pz)}_*n*_] (Cu_*n*_CO_3_; *n* = 27–31) are also present, except in the case of the nanojars with methylphosphonate. This is due to the fact that nanojars prefer to bind small anions with large hydration energies, such as CO_3_^2–^ (Δ*G*_h_° = −1324 kJ/mol), over larger, less hydrophilic anions. Although the reactions were carried out under a CO_2_-free atmosphere, small amounts of carbonate are present in the bases (Bu_4_NOH or NaOH) used for the synthesis. The use of excess phosphonate anion to minimize the interference of carbonate worked only in the case of the phosphonate with the smallest R substituent. Therefore, a different method was chosen for the synthesis of carbonate-free nanojars with larger phosphonates. This second method, which requires no base, relies on the depolymerization of [*trans*-Cu^II^(μ-OH)(μ-pz)]_∞_ in the presence of (Bu_4_N)_2_RPO_3_ in refluxing toluene (Figure 2). No reaction was observed with (Bu_4_N)_2_^*t*^BuPO_3_. In contrast with nanojars based on other anions, the Cu_*n*_RPO_3_ mixtures are not affected by NH_3_ treatment (which converts Cu_*n*_CO_3_ to Cu_27_CO_3_ and Cu_*n*_SO_4_ to Cu_31_SO_4_).^39^

**Figure 3:**
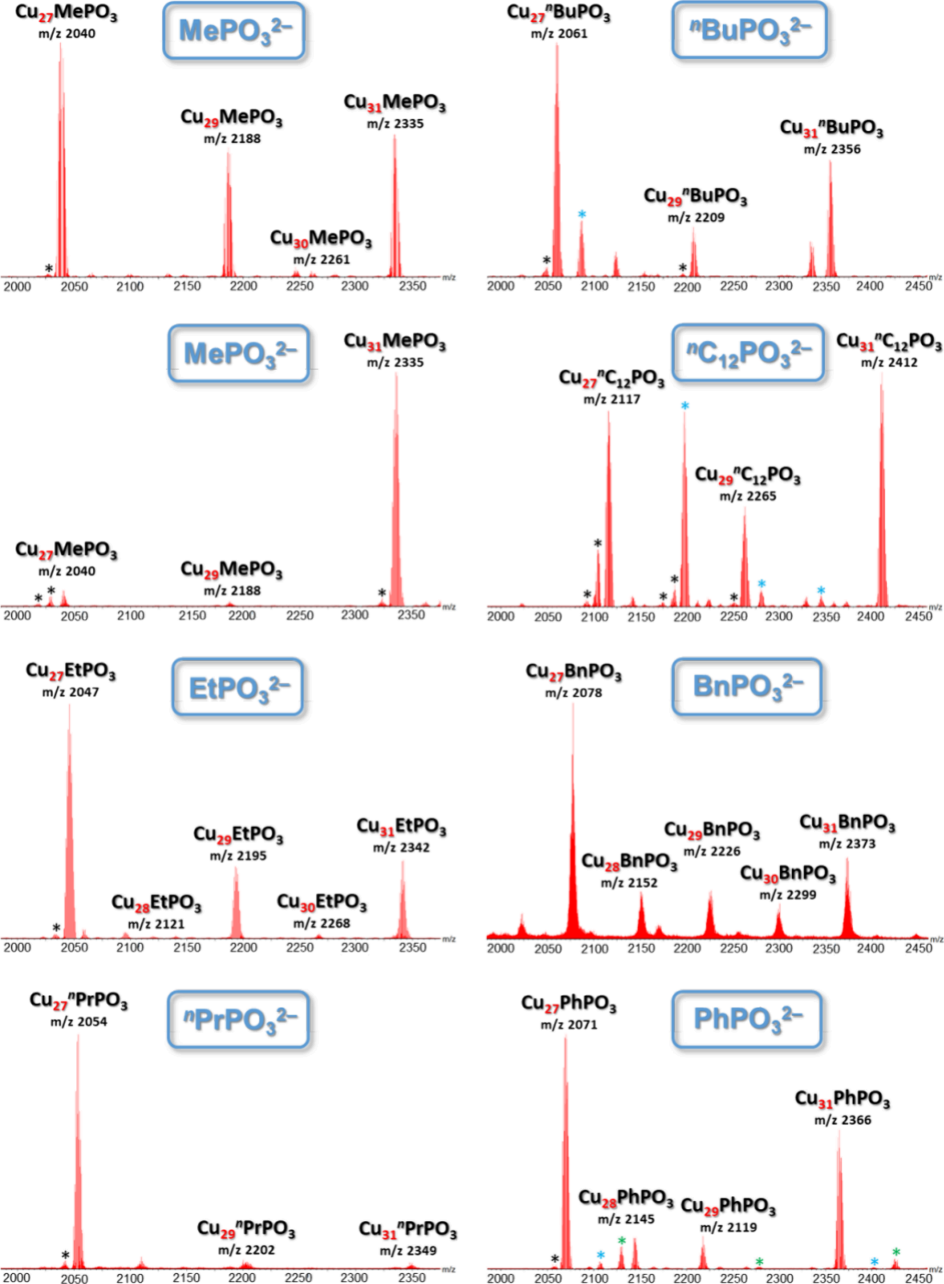
ESI-MS(−) spectra in CH_3_CN of the phosphonate-incarcerating nanojar mixtures [RPO_3_⊂{Cu(OH)(pz)}_*n*_]^2–^ (Cu_*n*_RPO_3_; *n* = 27–31; R = Me, Et, ^*n*^Pr, ^*n*^Bu, ^*n*^C_12_, Bn, Ph) obtained by depolymerization (except uppermost left: Cu_*n*_MePO_3_ obtained by direct reaction). Detailed isotopic distributions are shown in Figure S1. Nanojar derivatives with pairs of pz^–^ and OH^–^ moieties or pairs of two OH^–^ moieties substituted by phosphonate and with one or more pz^–^ moieties substituted by HCOO^–^ are indicated by blue, green, and black asterisks, respectively.

Figure 3 shows the ESI-MS(−) spectra of the nanojar mixtures obtained with different phosphonates with the corresponding *m*/*z* values. Occasionally, substituted nanojar derivatives also form. For example, phosphonate-substituted species (phosphonate substituting one OH and one pz ligand) are present in the case of the *n*-butyl-, *n*-dodecyl-, and phenylphosphonate anions, most prominently in the case of the *n*-dodecylphosphonate nanojars: [^*n*^C_12_PO_3_⊂{Cu_*n*_(OH)_*n*−*y*_(pz)_*n*−*y*_(^*n*^C_12_PO_3_)_*y*_}]^2–^ (*n* = 27: *y* = 1, *m*/*z* 2199; *y* = 2, *m*/*z* 2281; *n* = 29: *y* = 1, *m*/*z* 2347). In the case of the phenylphosphonate nanojars, different phosphonate-substituted species are also observed (phosphonate substituting two OH ligands), [PhPO_3_⊂{Cu_*n*_(OH)_*n*−2*y*_(pz)_*n*_(PhPO_3_)_*y*_}]^2–^ (*n* = 27: *y* = 1, *m*/*z* 2155; *y* = 2, *m*/*z* 2232; *n* = 29: *y* = 1, *m*/*z* 2347). It is noteworthy that despite the large excess (∼30-fold) of phosphonates used during synthesis, only minor amounts of phosphonate-substituted nanojars were obtained, although phosphonates are good ligands for copper and several multinuclear copper(II) phosphonate/pyrazolate complexes are known.^40^ Small amounts of formate-substituted analogues, in which one or more pz^–^ moieties are substituted by HCOO^–^, are also observed in most spectra at 11 *m*/*z* units less than the parent peak. Nanojars are extremely sensitive to even traces of formic acid, which is either present in the mass spectrometer due to its common use as an additive to improve peak shapes and to promote ionization by producing [M + H]^+^ ions or could result from the degradation of formaldehyde-based resins used in vial caps.^41,42^

### Structural Analysis by X-ray Crystallography

Nanojars consist of three stacked [*cis*-Cu^II^(OH)(pz)]_*x*_ metallamacrocyclic rings, with one larger central ring (*x* = 12–14) and two smaller side rings (*x* = 6–10). The rings are held together by axial Cu···O interactions and hydrogen bonds between the central ring and the side rings and by hydrogen bonds between the entrapped anion and the two side rings. The anion guest appears to act as an essential “glue” between the three rings, as an empty nanojar host has never been detected.

X-ray diffraction-quality single crystals of nanojars are difficult to obtain. Due to their toroidal shape, the close-packing of nanojars in 3-D lattices leaves large voids, which are only partially filled by the Bu_4_N^+^ counterions. The remaining voids accommodate several, usually highly disordered, solvent molecules, which are often disordered with the counterions as well. To successfully grow single crystals of the phosphonate nanojars, various uncommon solvents including 1-methylnaphthalene, methoxybenzene (anisole), and different isomers of dimethoxybenzene as the nanojar-dissolving solvent and different isomers of butanol as the precipitating solvent were considered in addition to more frequently used ones. Out of the over 1.3 million structures in the CSD only 83 contain anisole, 13 contain 1,3-dimethoxybenzene, 31 contain 1-methylnaphthalene, 224 contain *n*-butanol, and 107 contain *t*-butanol as a solvate molecule, compared to 18450 structures with toluene and 29266 with methanol.^36^ Crystal growing efforts using various combinations of solvents led to 12 crystal structures with the different phosphonate anions, including Cu_6+12+9_, Cu_6+12+10_, Cu_7+13+9_, and Cu_8+14+9_ nanojars: (Bu_4_N)_2_[MePO_3_⊂{Cu(OH)(pz)}_6+12+9_] (**1**, from 1,2-dichlorobenzene/*n*-heptane), (Bu_4_N)_2_[MePO_3_⊂{Cu(OH)(pz)}_7+13+9_] (**2**, from 1,2-dichlorobenzene/*n*-heptane), (Bu_4_N)_2_[MePO_3_⊂{Cu(OH)(pz)}_8+14+9_] (**3**, from anisole/*n*-butanol), (Bu_4_N)_2_[EtPO_3_⊂{Cu(OH)(pz)}_8+14+9_] (**4**, from 1-methylnaphthalene/hexanes), (Bu_4_N)_2_[EtPO_3_⊂{Cu(OH)(pz)}_8+14+9_]_0.95_[EtPO_3_⊂{Cu(OH)(pz)}_7+13+9_]_0.05_ (**5**, from anisole/*n*-butanol), (Bu_4_N)_2_[^*n*^BuPO_3_⊂{Cu(OH)(pz)}_7+13+9_] (**6**, from chlorobenzene/*n*-heptane), (Bu_4_N)_2_[^*n*^C_12_PO_3_⊂{Cu(OH)(pz)}_8+14+9_] (**7**, from 1,2-dichlorobenzene/*n*-heptane), (Bu_4_N)_2_[BnPO_3_⊂{Cu(OH)(pz)}_8+14+9_] (**8**, from 1,2-dichlorobenzene/*n*-heptane), (Bu_4_N)_1.53_(Bn_3_MeN)_0.47_[PhPO_3_⊂{Cu(OH)(pz)}_6+12+10_]_0.87_[PhPO_3_⊂{Cu(OH)(pz)}_6+12+9_]_0.13_ (**9**, from 1,3-dimethoxybenzene/*n*-heptane), (Bu_4_N)_2_[PhPO_3_⊂{Cu(OH)(pz)}_8+14+9_] (**10**, from 1,3-dimethoxybenzene/*n*-heptane), (Bu_4_N)_4_[EtPO_3_⊂{*cis*-Cu^II^(μ-OH)_27_(μ-pz)_25_(*μ*_*4*_-EtPO_3_)}]_2_ (**11**, from 1-methylnaphthalene/*t*-butanol), and (Bu_4_N)_4_[^*n*^PrPO_3_⊂{*cis*-Cu^II^(μ-OH)_27_(μ-pz)_25_(*μ*_*4*_-^*n*^PrPO_3_)}]_2_ (**12**, from anisole/*t*-butanol). Structure **9** contains an additional counterion, Bn_3_MeN^+^, which was used as an additive in the form of its nitrate salt during crystallization. In all structures, the nanojar unit is located in a general position within the crystal lattice. Tables S1−S53 and Figures S2−S13 (thermal ellipsoid plots) contain details of the structures.

**Figure 4:**
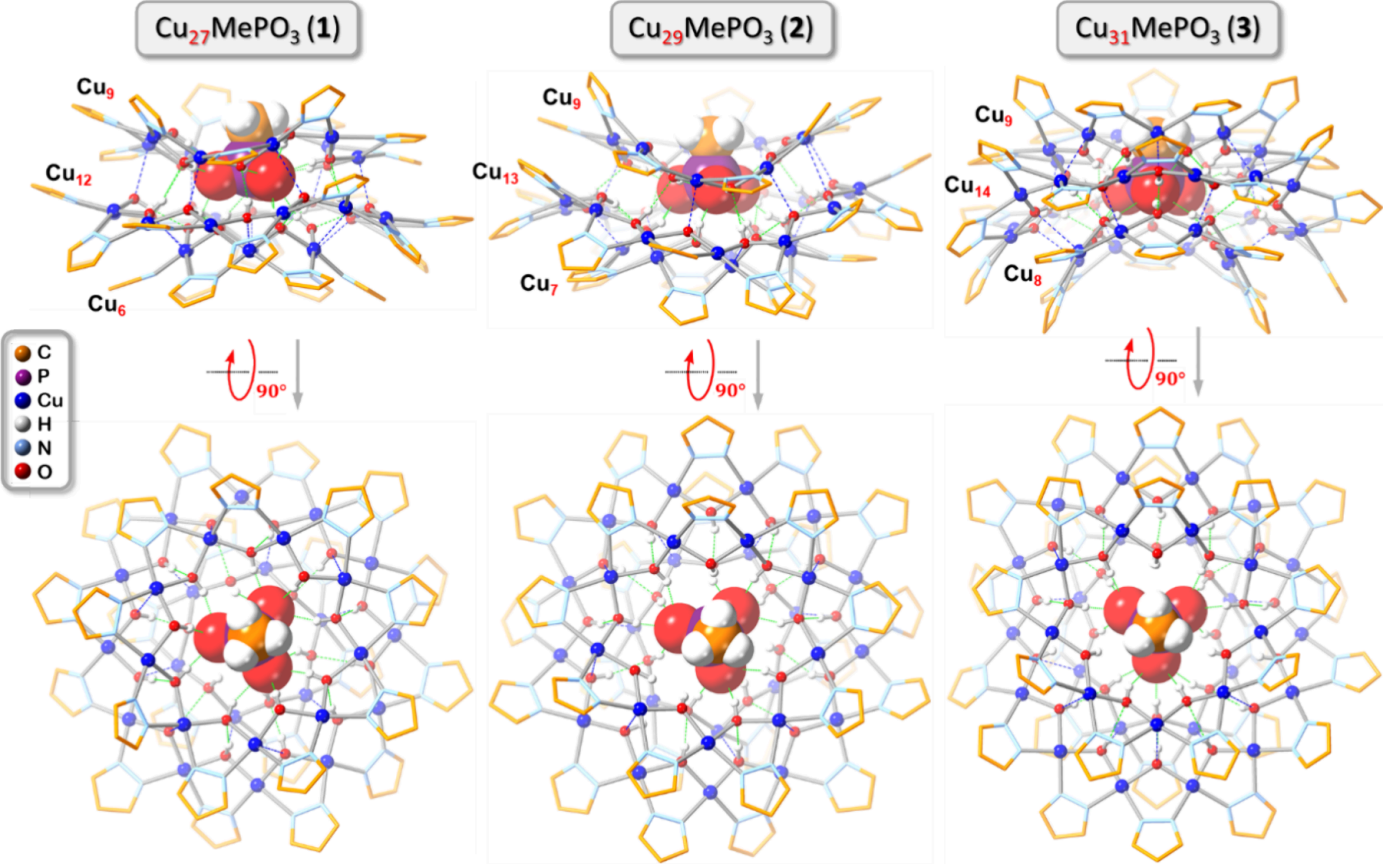
Ball-and-stick representation of the crystal structures of **1**–**3** (side and top views). Green and blue dotted lines indicate hydrogen bonds and axial Cu···O interactions, respectively. Counterions, lattice solvent molecules, and C–H bond H atoms are omitted for clarity, and only the major component is shown for disordered moieties. For **2**, only one of the two crystallographically independent nanojar moieties from the asymmetric unit is shown.

**Figure 5:**
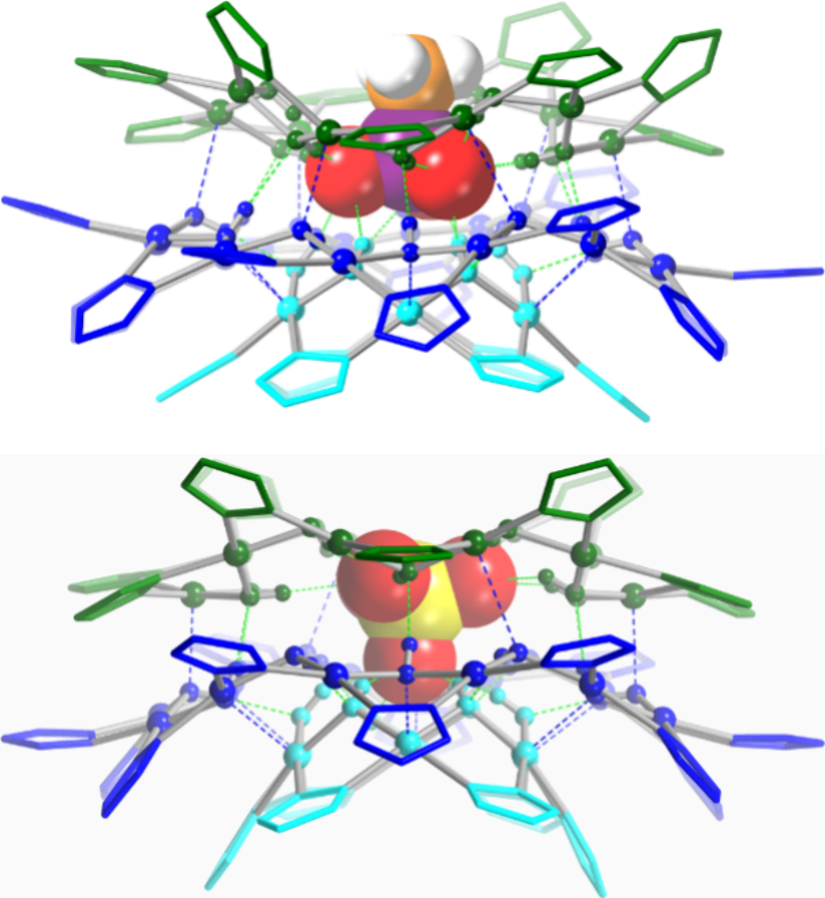
Comparison of the crystal structures of Cu_6+12+9_MePO_3_ and Cu_6+12+10_SO_4_, illustrating the binding of the anions in opposite orientations. Color code: cyan, Cu_6_ ring; blue, Cu_12_ ring; green, Cu_9_ (upper) or Cu_10_ ring (lower).

With MePO_3_^2–^, three different nanojar sizes, Cu_27_MePO_3_ (**1**), Cu_29_MePO_3_ (**2**), and Cu_31_MePO_3_ (**3**) were crystallized, with no disorder in the position of the bound anion (Figure 4). The three structures have a Cu_9_ side ring in common, with Cu_12_, Cu_13_, and Cu_14_ rings as the central ring and Cu_6_, Cu_7_, and Cu_8_ rings as the other side ring. In all three structures, the methyl group of the MePO_3_^2–^ anion points outward through the Cu_9_ ring, while the three O atoms point toward the Cu_6_, Cu_7_, or Cu_8_ rings. In the case of the Cu_6+12+9_ nanojar, this is in stark contrast with the orientation of the SO_4_^2–^ anion in the similar Cu_6+12+10_ nanojar (Cu_6+12+9_SO_4_ has not been crystallized yet).^43^ In Cu_6+12+10_SO_4_, three O atoms of the sulfate ion point toward the larger Cu_10_ side ring, and the fourth O atom (equivalent to the methyl group in MePO_3_^2–^) points toward the smaller Cu_6_ side ring (Figure 5). This is undoubtedly a result of the larger size of the methyl group compared to the O atom and the inability of the former to form hydrogen bonds with the nanojar host. In Cu_8+14+9_SO_4_, the sulfate ion does have the same orientation as MePO_3_^2–^ in Cu_8+14+9_MePO_3_, with three O atoms pointing toward the smaller Cu_8_ ring and the fourth O atom pointing toward the larger Cu_9_ ring (Figure S14). No comparison can be made in the case of the Cu_7+13+9_ nanojar since it could not be crystallized with sulfate (or with any other tetrahedral anion). Nevertheless, crystals of the Cu_7+13+9_ nanojar were also obtained with EtPO_3_^2–^, which is found cocrystallized with Cu_8+14+9_EtPO_3_ in a 5/95 ratio in **5** (wherein the Cu_7_/Cu_8_ and Cu_13_/Cu_14_ rings are disordered with each other and the nondisordered Cu_9_ ring is shared by the Cu_29_ and Cu_31_ nanojars; the whole EtPO_3_^2–^ ion is disordered over three positions in a 62/33/5 ratio) and ^*n*^BuPO_3_^2–^ (**6**; the PO_3_ moiety is disordered over two positions in a 82/18 ratio, by a ∼ 60° rotation approximately around the P–C bond), which show similar binding of the anion (Figure S15). However, as the size of the substituent increases, the number of hydrogen bonds to the phosphonate group shorter than 3.2 Å decreases from 13/12 for Me (two crystallographically independent nanojar moieties in the asymmetric unit; longest O···O distance: 3.172(8)/3.160(7) Å) to 12 for Et (longest O···O distance: 3.05(4) Å) and 11 for ^*n*^Bu (longest O···O distance: 2.943(6) Å) (Tables S5 and S6).

The Cu_6+12+9_ nanojar could also be crystallized with PhPO_3_^2–^ (Figure S16, Table S3). In **9**, Cu_6+12+9_PhPO_3_ is found cocrystallized with Cu_6+12+10_PhPO_3_ in a 18/82 and 9/91 ratio in the two crystallographically independent nanojar units, wherein the Cu_9_/Cu_10_ rings are disordered with each other and the nondisordered Cu_6_ and Cu_12_ rings are shared by the Cu_27_ and Cu_28_ nanojars. Similarly to the methyl derivative and in contrast to SO_4_^2–^, the three O atoms of the PhPO_3_^2–^ moieties point toward the Cu_6_ ring while the phenyl group points outward through the Cu_9_/Cu_10_ ring. The O atoms of the PO_3_ moieties in the two nanojar units are disordered over two positions in a 88/12 and 86/14 ratio, by a 51.2(9) and 50.2(10)° rotation around the P–C bond, respectively. No aromatic interactions between the phenyl group of the PhPO_3_^2–^ anion and pz moieties are observed in **9**.

**Figure 6:**
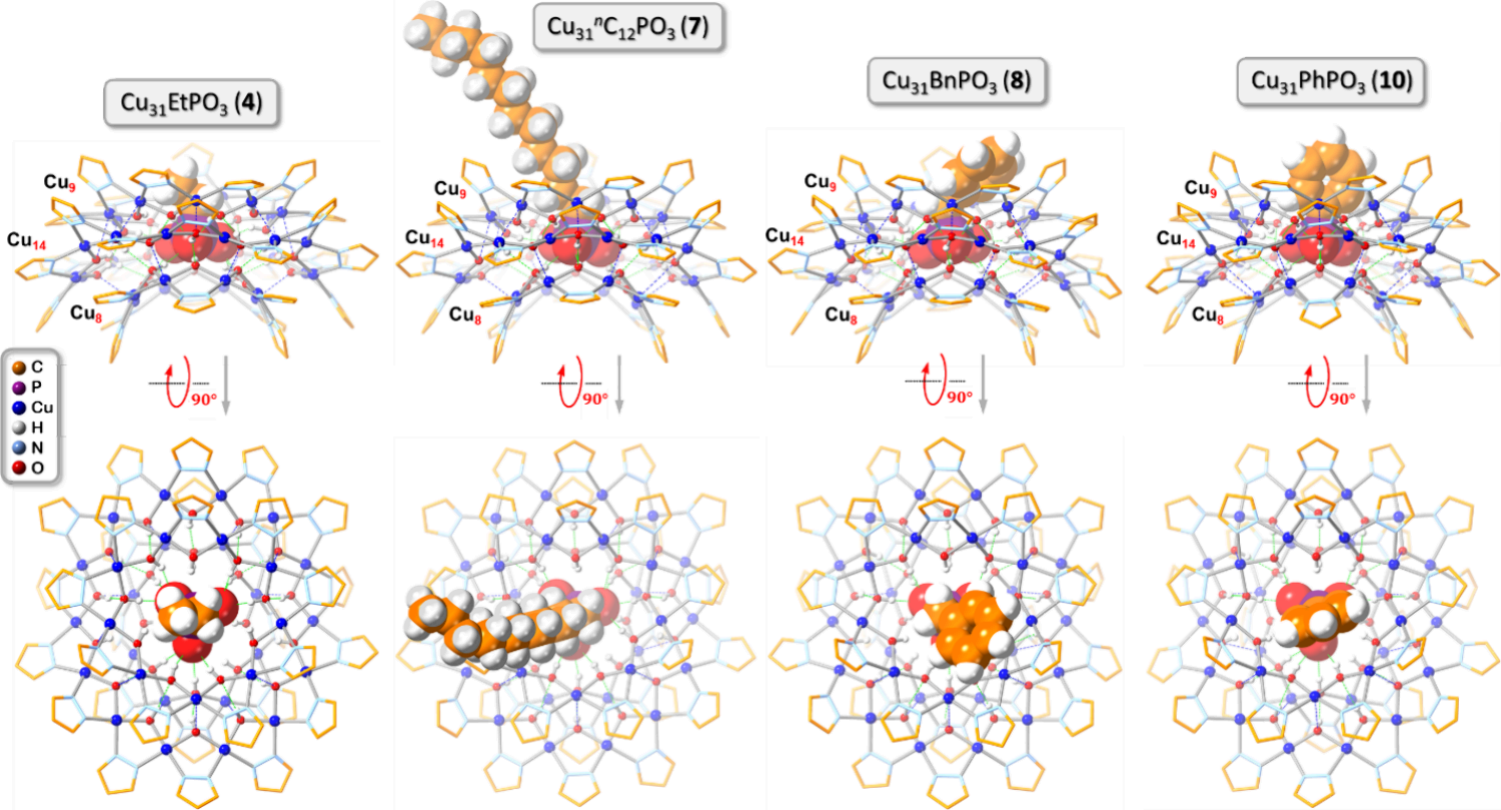
Ball-and-stick representation of the crystal structures of **4**, **7**, **8**, and **10** (side and top views). Green and blue dotted lines indicate hydrogen bonds and axial Cu···O interactions, respectively. Counterions, lattice solvent molecules, and C–H-bond H atoms are omitted for clarity, and only the major component is shown for disordered moieties. For **7**, only one of the two crystallographically independent nanojar moieties from the asymmetric unit is shown.

The Cu_8+14+9_ nanojar was crystallized with MePO_3_^2–^ (**3**), EtPO_3_^2–^ (**4**; also, cocrystallized with Cu_7+13+9_EtPO_3_ in **5**), *n*-dodecylPO_3_^2–^ (**7**), BnPO_3_^2–^ (**8**), and PhPO_3_^2–^ (**10**) (Figure 6). The anion is found in a single orientation in **3**, **7**, and **9**. In **4** and **7**, although the PO_3_ group is also in a unique orientation, the alkyl group is disordered over two positions (0.57/0.43 and 0.70/0.30 occupancy for Et and *n*-dodecyl, respectively). As with other nanojar sizes, the three O atoms of the phosphonate moieties point toward the smaller Cu_8_ side ring, whereas the substituents point outward through the larger Cu_9_ side ring. Aromatic interactions between the phenyl group of the phosphonate anion and a pz ring are observed in the case of **8** and **10**. In **8**, the pz moiety involved in the aromatic interaction is disordered over two positions in a 56/44 ratio, with dihedral angles between Ph/pz mean planes of 12.7(6) and 13.2(7)°, centroid-centroid distances of 4.343(9) and 4.758(10) Å, and shortest H atom to plane centroid distances of 3.180(7) and 3.673(9) Å, respectively. In **10**, the phenyl group of the PhPO_3_^2–^ anion has interactions with two different pz moieties, with dihedral angles between Ph/pz mean planes of 31.3(2) and 65.6(2)°, centroid-centroid distances of 5.435(3) and 6.061(2) Å, and shortest H atom to plane centroid distances of 3.996(2) and 4.213(2) Å, respectively.

Disorder in the positions of some pyrazolate ligands within the nanojar is also common. Except in **2** and **4**, one or more pz moieties are disordered over two positions. In **8**, a whole segment of the Cu_9_ ring including six Cu(pz)(OH) units is disordered over two positions with a 0.56/0.44 occupancy.

Despite the different bound anions and varying ring sizes, the structural parameters within the Cu_*x*_ rings in **1**–**10** (excluding the minor disordered nanojar components in **5** and **9**, which offer less accurate values) are consistent, with average Cu–O bond lengths of 1.920(6)–1.928(4) Å, average Cu–N bond lengths of 1.970(6)–1.981(4) Å, average *trans* and *cis* N–Cu–O angles of 169.4(2)–172.2(2)° and 85.2(1)–86.0(1)°, respectively, and average Cu···Cu distances of 3.277(2)–3.327(1) Å (Tables S5–S8). The noncovalent interactions between individual Cu_*x*_ rings are comparable, with average axial Cu···O distances of 2.483(5)–2.540(5) Å and average H-bonded O···O distances of 2.744(5)–2.868(6) Å. The H-bonding parameters between Cu_*x*_ rings and the bound phosphonate anion are also similar, with average O···O distances ranging from 2.836(5) Å (11 H-bonds <3.2 Å) in **6** to 2.965(13) Å (12 H-bonds <3.2 Å) in **4**.

Differences in structural details of the various Cu_*x*_ rings in the Cu_27_–Cu_31_ nanojars were analyzed using the dihedral angles and the component fold and twist angles between the mean planes of pyrazolate moieties and adjacent Cu–O–Cu units (as defined earlier).^42,44^Tables S10 and S11 illustrate that in **1**–**10** the average fold angles in the larger, flatter central rings (Cu_12_–Cu_14_) are quite consistent at 41.0(6)–43.2(2)°, whereas in the two smaller, more puckered side rings, the angles vary more widely: 37.9(2)–51.8(5)° (Cu_9_ or Cu_10_) and 45.6(4)–56.4(2)° (Cu_6_–Cu_8_). The corresponding average twist angles are 3.4(2)–6.2(4)° in the central rings (Cu_12_–Cu_14_), and 2.1(2)–5.2(3)° (Cu_9_ or Cu_10_) and 0.9(2)–5.2(2)° (Cu_6_ or Cu_8_) in the two side rings. Despite the pronounced variations of 78 and 18° between the individual fold (0.31(18)–78.4(3)°) and twist angles (0.0(4)–17.5(2)°), only small variations of <14 and <5° are observed for the fold and twist angle averages, respectively.

**Figure 7:**
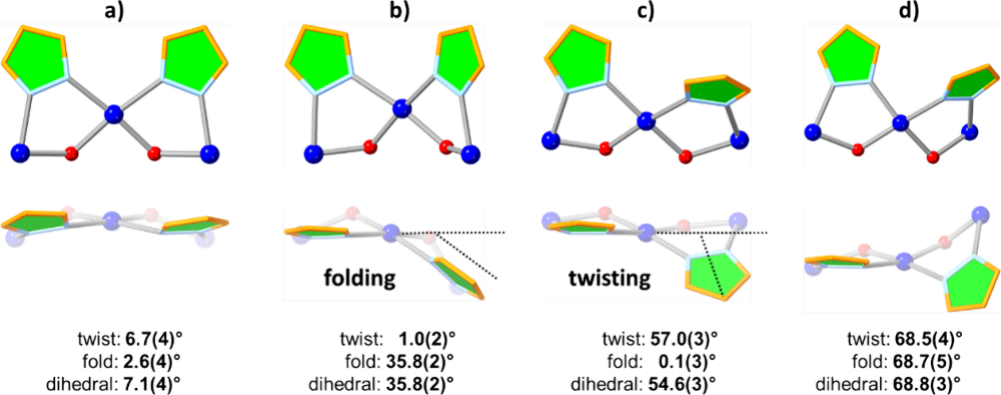
Examples of different pz–pz mean-plane dihedral angles based on (a) small twist and fold angles, (b) small twist angle and large fold angle, (c) large twist angle and small fold angle, and (d) large twist and fold angles. Blue and red spheres represent Cu atoms and OH groups, respectively.

Herein, we introduce a new structural descriptor for Cu_*x*_ ring geometry, namely, the dihedral angles and the component fold and twist angles between the mean planes of adjacent pyrazolate moieties. Because they are placed next to each other in each *cis*-Cu(pz)_2_(OH)_2_ fragment, the pz moieties avoid steric hindrance by twisting and/or folding away from a coplanar geometry (Figure 7). In the large, rather flat Cu_12_–Cu_14_ central rings, the favored distortion is by twisting, as the pyrazolate units can adopt an alternating up/down orientation relative to the mean plane of the Cu atoms. In contrast, in the small, bowl-shaped Cu_6_ side-ring, the favored distortion is by folding. Thus, the average twist and fold angles in **1**–**10** are 55.4(4)–57.8(4) and 12.3(3)–31.9(2)° for the Cu_12_–Cu_14_ rings, whereas for the Cu_6_ ring, the corresponding average values are reversed at 9.9(2)–22.0(3) and 27.1(2)–42.1(3)°. For the Cu_7_ ring, an approximately equal preference for twisting (21.1(4)–24.9(3)°) and folding (23.8(3)–28.1(4)°) is observed. As the ring becomes larger, distortion by twisting is increasingly preferred (Tables S12–S14).

A relationship of the centroid–centroid distances between adjacent pz mean planes with the corresponding dihedral angles is also noteworthy. Although they are not directly proportional, dihedral angles larger than ∼35° are consistently (with very few exceptions) correlated with centroid–centroid distances shorter than 5.0 Å, whereas dihedral angles smaller than ∼35° are linked with centroid–centroid distances longer than 5.0 Å (Tables S12–S14).

The four- or five-coordinate geometry of the Cu atoms in different Cu_*x*_ rings in **1**–**12** was analyzed using the coordination geometry indexes τ_4_ and τ_5_ (Table S15).^45,46^ In the case of τ_4_, a value of 1.00 corresponds to a perfect tetrahedral geometry and a value of zero corresponds to a perfect square planar geometry, whereas in the case of τ_5_, 1.00 and zero indicate a perfect trigonal bipyramidal and perfect square pyramidal geometry, respectively. The τ_4_ values in **1**–**12** range from 0.03 to 0.40 with an average of 0.13, whereas the τ_5_ values range from 0.00 to 0.49 with an average of 0.08, indicating that most of the Cu atoms have coordination geometries very close to square planar or square pyramidal. As seen in Tables S5–S9, 12–14 Cu atoms of nanojars (Cu_27_–Cu_31_) are five-coordinated with axial Cu···O distances shorter than the sum of the van der Waals radii of Cu and O (2.92 Å). All Cu atoms of the larger central Cu_*x*_ ring (*x* = 12–14) in nanojars are four-coordinated lacking a nearby axial O atom, whereas in the smaller side-rings (*x* = 6–10), the number of five-coordinate Cu atoms is approximately equally distributed between the two rings. No correlation is observed between the τ_4_ (or τ_5_) values and the dihedral (or component fold and twist) angles between the two pz moieties bound to a given Cu atom.

In terms of coplanarity of the Cu atoms in the eight different Cu_*x*_ rings of the Cu_27_–Cu_31_ nanojars, the Cu_6_ ring (overall bowl-shaped with all pz groups on one side of the ring and all OH groups on the opposite side) is closest to planar with average deviations of only 0.028–0.098 Å from the Cu_6_ mean plane (largest deviation: 0.049 Å in **1** and 0.155 Å in **9**). This is in contrast with the Cu_12_ ring, which is overall flat with pz groups alternating on opposite sides of the ring, but with large average deviations of 0.227–0.342 Å (largest deviation: 0.439 Å in **1** and 0.584 Å in **9**) of its Cu atoms from the Cu_12_ mean plane. The largest average deviations from coplanarity are observed in the case of the largest Cu_14_ ring, ranging from 0.631 to 0.689 Å (largest deviation: 1.493 Å in **3**, 1.283 Å in **4**, 1.390 Å in **5**, 1.366 Å in **7**, 1.427 Å in **8**, and 1.424 Å in **10**). The average deviations in the Cu_7_ and Cu_10_ rings (0.194–0.299 Å) are similar to the ones of the Cu_12_ ring, whereas in the Cu_8_, Cu_9_, and Cu_13_ rings the values are 0.331–0.648 Å (Table S16).

**Figure 8:**
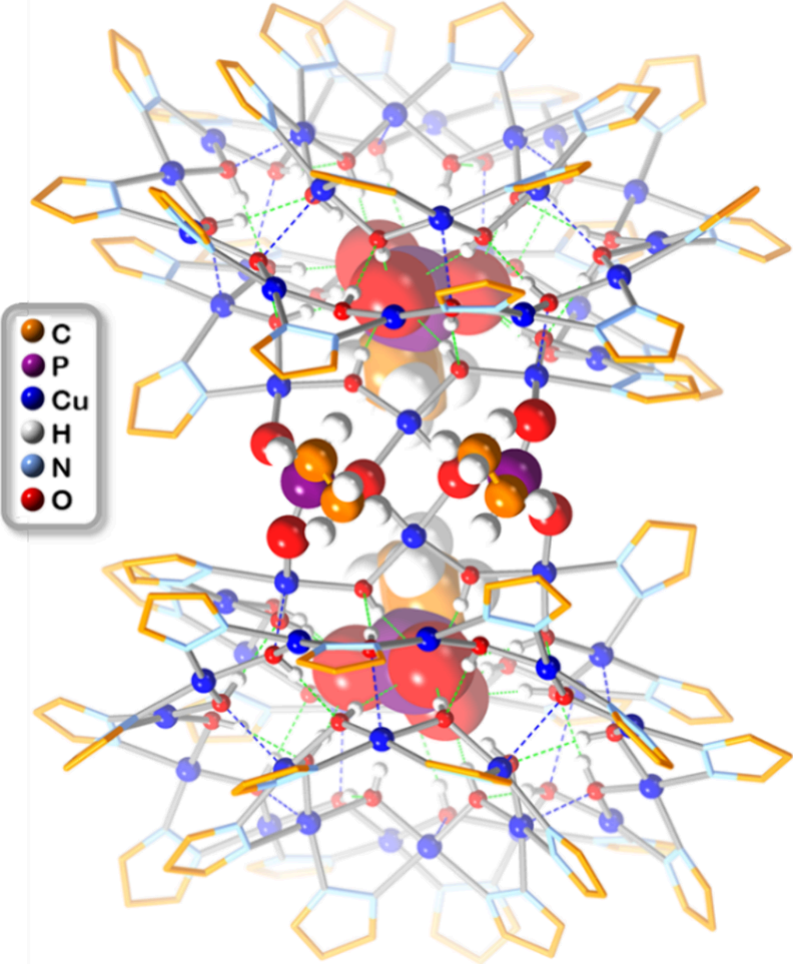
Ball-and-stick representation of the crystal structure of **11**. Green and blue dotted lines indicate hydrogen bonds and axial Cu···O interactions, respectively. Counterions, lattice solvent molecules, and pz H atoms are omitted for clarity.

Besides the intended outcome of favoring the crystallization of different nanojar sizes from a mixture, the use of different crystallization solvents also led to surprising new results. While the vapor diffusion of hexanes into a 1-methylnaphthalene solution of the Cu_*n*_EtPO_3_ nanojars provided crystals of Cu_31_EtPO_3_, changing the precipitant solvent to *t*-butanol led to the unexpected formation of a clamshell-like structure (**11**; Figures 8 and S17). This novel motif consists of a pair of Cu_6+12+9_EtPO_3_ nanojars tethered by two adjacent EtPO_3_^2–^ ions, each replacing two pyrazolate moieties (one from each nanojar unit). The clamshell structure is located on a *C*_2_ rotation axis passing in-between the two EtPO_3_ units. Consequently, only half of the molecule (one nanojar unit) is located within the asymmetric unit of the crystal lattice. The overall structure of the clamshell’s nanojar unit is very similar to the one of the individual nanojars, with the notable exception of the longer average Cu···Cu distances of 3.356(2) Å within the Cu_9_ rings, compared to 3.311 Å in regular nanojars (Table S9). This is due to the larger binding angle of the phosphonate O atoms compared to the pyrazolate N atoms, which causes two of the Cu···Cu distances within the Cu_9_ ring to increase to 3.496(2) and 3.555(2) Å.

No individual nanojars could be crystallized with ^*n*^PrPO_3_^2–^. Instead, a nanojar clamshell similar to **11** was obtained from an anisole solution of Cu_n_^*n*^PrPO_3_ by vapor diffusion of *t*-butanol (Figure S18). In the crystal lattice of **12**, which is also monoclinic (although in the *P*2_1_/*n* space group instead of *C*2/*c* in the case of **11**), the clamshell is not located on a *C*_2_ rotation axis. Consequently, the two nanojar units are not symmetry-related. As in **11**, a longer average Cu···Cu distance of 3.355(1) is observed within the Cu_9_ rings of **12**, due to longer Cu···Cu distances of 3.475(1)–3.559(1) Å between Cu atoms bridged by phosphonate instead of pyrazolate ligands (Table S9).

**Figure 9:**
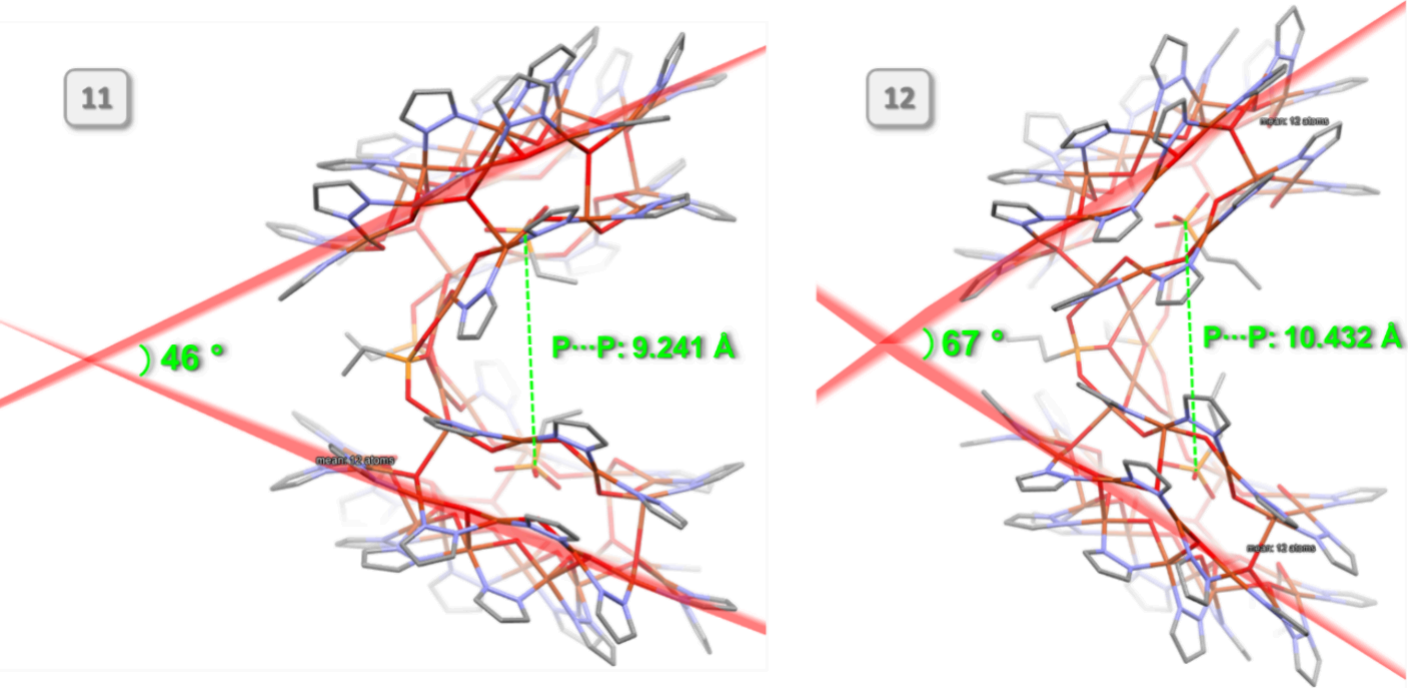
Illustration of the different angles of opening in nanojar clamshells **11** and **12**.

In spite of the apparent similarity, there is an important difference between the structures of **11** and **12**, likely due to the larger size of the ^*n*^Pr chain compared to Et. In **11**, the dihedral angle between mean planes running through the 12 Cu atoms of the Cu_12_ rings is 46.46(1)° with a centroid···centroid distance of 13.632(1) Å and a P···P separation between centrally bound anions of 9.241(2) Å, whereas the corresponding values in **12** increase to 66.89(1)°, 14.455(2) and 10.432(2) Å (Figures 9 and S19). Moreover, not only is the clamshell opening significantly larger in **12** (illustrated by the larger fold angle of 86.57(1)° compared to 66.30(3)° in **11**) but also the twist angle between nanojar units (measured between Cu_12_ mean planes as defined above), which increases from 0° in **11** (crystallographically imposed) to 31.86° in **12**.

### ^1^H NMR Spectroscopy

**Figure 10:**
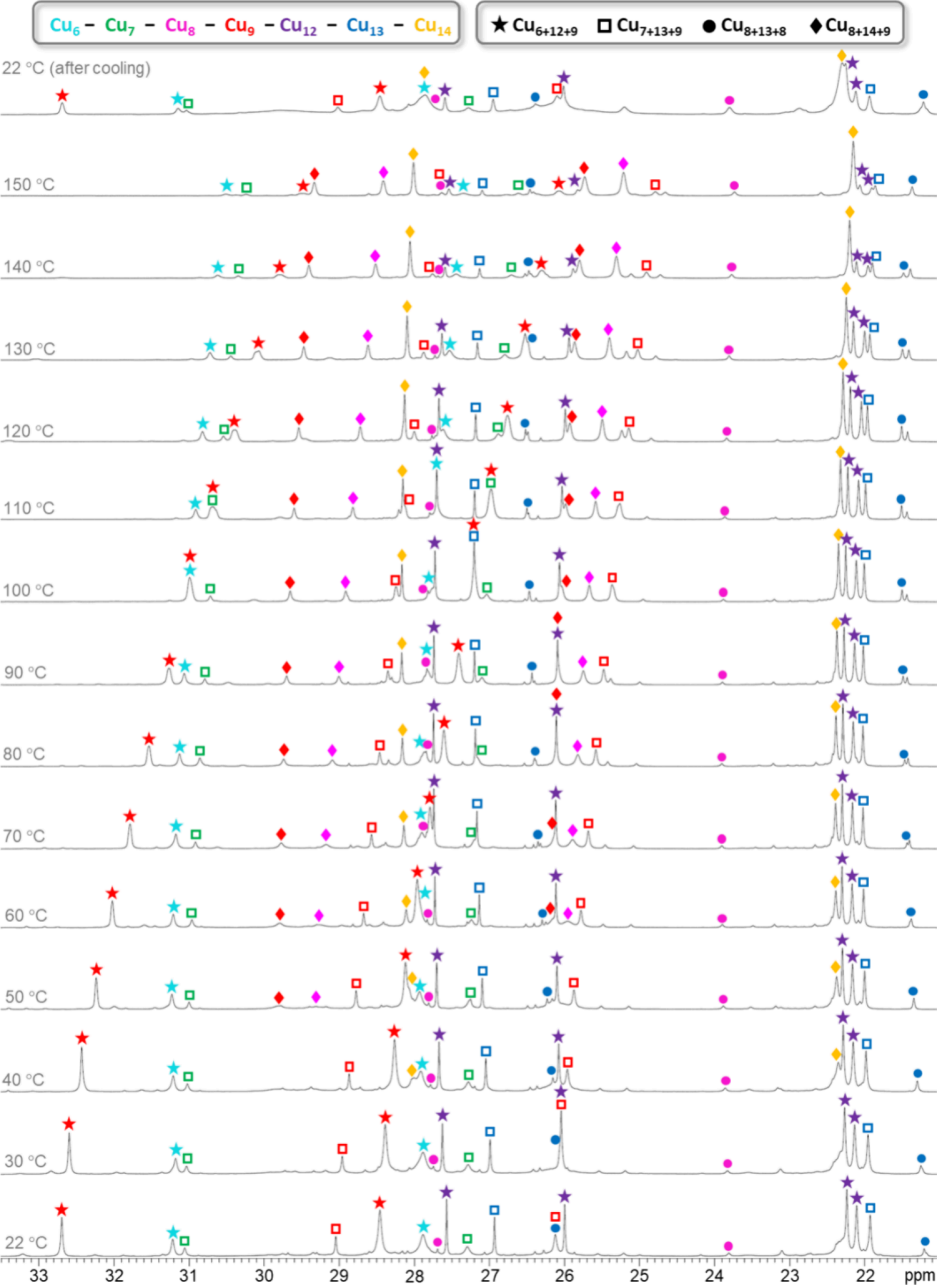
Variable-temperature ^1^H NMR spectra of the Cu_n_MePO_3_ (*n* = 27–31) nanojar mixture in DMSO-*d*_6_, showing pyrazolate proton signals in the 21–33 ppm window. The temperatures shown are the target temperatures of the probe.

**Figure 11:**
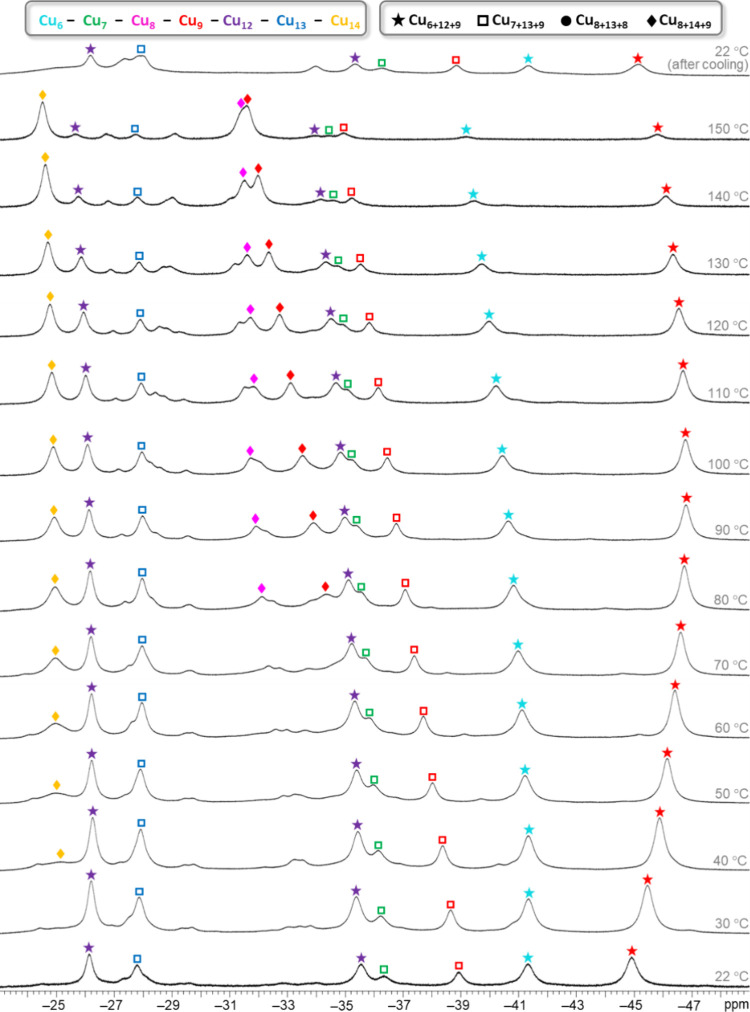
Variable-temperature ^1^H NMR spectra of the Cu_*n*_MePO_3_ (*n* = 27–31) nanojar mixture in DMSO-*d*_6_, showing OH proton signals in the −24 to −48 ppm window. The given temperatures are the target temperatures of the probe.

To enable VT-NMR experiments at high temperatures, DMSO-*d*_6_ was chosen as the solvent. Previous studies have shown that DMSO, despite being a good coordinating solvent, does not interfere with the nanojar framework and does not induce speciation at room temperature.^39^ While the ^1^H NMR spectra of the Cu_*n*_RPO_3_ nanojars (R = Me, Et, ^*n*^Pr, ^*n*^Bu, Bn, and Ph) in DMSO-*d*_6_ are similar to the ones of Cu_*n*_CO_3_ and Cu_*n*_SO_4_ in that the paramagnetism of the Cu^2+^ centers leads to drastic downfield and upfield shifts of the pyrazolate and OH proton peaks, respectively, as well as to broadening of the peaks and loss of the *J* coupling between nuclei, there are significant differences to be pointed out (Figures 10, 11, and S20–S31, Table S54). MePO_3_^2–^ is similar to SO_4_^2–^ considering their tetrahedral shape, but different in terms of charge distribution (formal charge on O atoms is −0.67 in the former and −0.50 in the latter) and number of hydrogen bond acceptor atoms (three vs four). Conversely, MePO_3_^2–^ is different from the trigonal-planar CO_3_^2–^ in terms of shape but identical with regard to the number of hydrogen bond acceptor atoms and their formal charges. Phosphonates (HPO_3_H_2_: p*K*_*a*1_ = 1.43, p*K*_*a*2_ = 6.68; MePO_3_H_2_: p*K*_*a*1_ = 2.12, p*K*_*a*2_ = 7.29; EtPO_3_H_2_: p*K*_*a*1_ = 2.43, p*K*_*a*2_ = 8.05; ^*n*^PrPO_3_H_2_: p*K*_*a*1_ = 2.49, p*K*_*a*2_ = 8.18; BnPO_3_H_2_: p*K*_*a*1_ = 2.4, p*K*_*a*2_ = 7.8; PhPO_3_H_2_: p*K*_*a*1_ = 1.83, p*K*_*a*2_ = 7.07) are more basic than sulfate (H_2_SO_4_: p*K*_*a*1_ ≃ – 3; p*K*_*a*2_ = 1.99) but less basic than carbonate (H_2_CO_3_: p*K*_*a*1_ = 3.49; p*K*_*a*2_ = 10.33).^47−49^

Subsequent discussion will focus on the Cu_6+12+9_ nanojars, since they could be obtained with CO_3_^2–^, SO_4_^2–^, and various phosphonates RPO_3_^2–^ (R = Me, Et, ^*n*^Pr, ^*n*^Bu, Bn, and Ph) as well. At ambient temperature, pyrazolate proton peaks for Cu_6+12+9_CO_3_ and Cu_6+12+9_SO_4_ are observed in the 37.63–22.43 and 34.83–22.56 ppm windows, respectively, whereas the corresponding OH proton peaks are observed from −29.65 to −68.08 ppm and from −26.18 to −52.95 ppm. For Cu_6+12+9_MePO_3_, the corresponding windows are 32.75–22.16 and −26.14 to −45.10 ppm. A closer inspection of the individual chemical shifts (δ) for the different Cu_*x*_ rings in Table S54 shows no consistent correlation with the basicity of the entrapped anions (more deshielding of the H atoms of OH groups hydrogen-bonded to more basic anions is expected). Thus, the variation of chemical shifts on changing the anion is apparently more profoundly influenced by changes in the magnetism of the Cu_*x*_ rings as a consequence of structural changes within the nanojar. In the case of Cu_6+12+9_CO_3_ vs Cu_6+12+9_SO_4_, the differences are caused by the different shape and orientation of the anions. In Cu_6+12+9_CO_3_, the three O atoms of CO_3_^2–^ form H bonds with both the Cu_6_ and Cu_9_ rings,^39^ whereas in Cu_6+12+9_SO_4_, one O atom of SO_4_^2–^ forms H bonds only with the Cu_6_ ring and the other three only with the Cu_9_ ring (assumed by analogy to Cu_6+12+10_SO_4_, as discussed in the Crystallography section).^43a^

**Figure 12:**
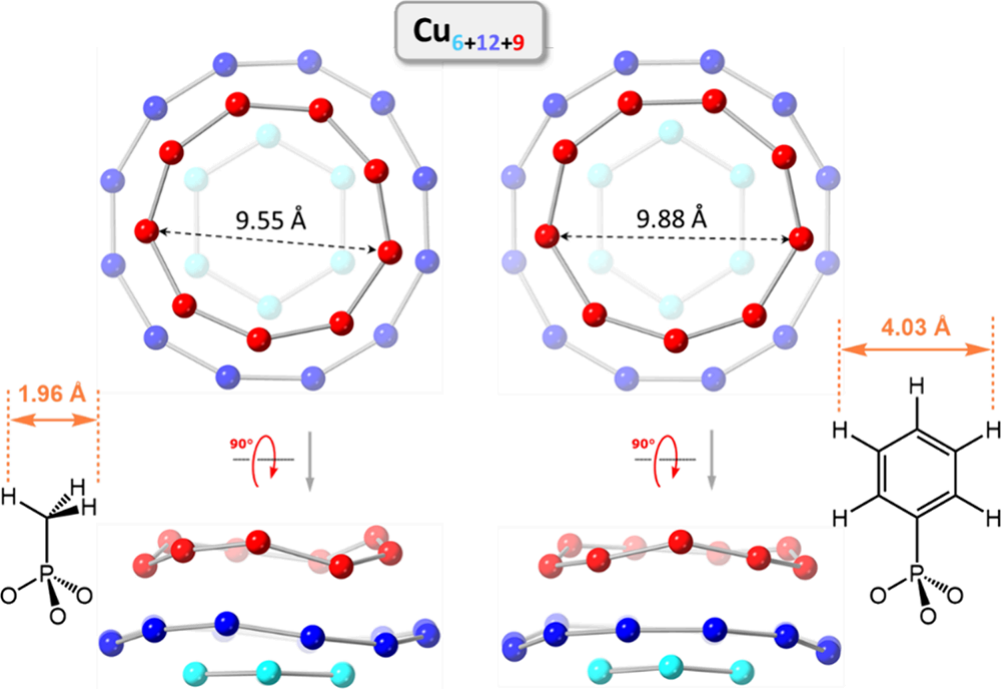
Comparison of the Cu_6+12+9_ rings in **1** and **9** (only Cu atoms shown; the two symmetry-independent nanojar units of **9** are virtually identical) and the corresponding MePO_3_^2–^ and PhPO_3_^2–^ anions.

For the different RPO_3_^2–^ anions, an expected deshielding of the H atoms of the Cu_6_ and Cu_9_ rings H-bonded to the incarcerated anion is observed on going from MePO_3_^2–^ (−41.30 and −45.10 ppm) to the more basic Et (−41.07 and −43.99 ppm), ^*n*^Pr (–41.0 and –41.0 ppm), ^*n*^Bu (−40.40 and −41.33 ppm), and Bn derivatives (−38.60 and −38.86 ppm). However, an even more pronounced deshielding is observed with the less basic PhPO_3_^2–^ anion (−34.14 and −39.68 pm). This inconsistency must be a result of differences in the structure of the nanojar framework as the orientations of the different RPO_3_^2–^ anions relative to the nanojar framework is identical. Indeed, an analysis of the structures of the Cu_6+12+9_ nanojars with MePO_3_^2–^ and PhPO_3_^2–^ reveals subtle, yet significant differences in the shape and orientation of the Cu_*x*_ rings relative to each other, caused by the sterically (laterally) more demanding Ph vs Me (or *n*-alkyl) substituent on the phosphonate moiety (Figure 12). These structural changes are expected to affect the magnetism of the Cu_*x*_ rings, which, in turn, affects the hyperfine shifts of the nanojar protons.

The VT ^1^H NMR measurements of the phosphonate nanojars in DMSO-*d*_6_ over the 20–150 °C range also show significant differences compared to the ones of the carbonate and sulfate nanojars (Table S54). For example, in the case of the Cu_6+12+9_ nanojar, the degree of temperature dependence of the chemical shifts of the Cu_9_ ring protons is significantly smaller with alkyl- and phenylphosphonates (1.5–3.8 ppm for pz and 0.3–1.9 ppm for OH) than with carbonate (4.7–5.2 ppm for pz and 13.7 ppm for OH) and sulfate (3.8–4.6 ppm for pz and 4.9 ppm for OH). The benzylphosphonate analogue shows a much larger variation of 5.1 ppm for the OH protons and a smaller variation of 1.3–2.0 ppm for the pz protons, compared to the other phosphonates. The Cu_6_ and Cu_12_ rings show smaller degrees of variation across all phosphonate nanojars, which are essentially the same as with carbonate and sulfate (Cu_6_: 0.3–0.9 ppm for pz and 1.1–2.5 ppm for OH; Cu_12_: 0.1–0.3 ppm for pz and 0.3–1.4 ppm for OH, with an outlier of 2.7 ppm for phenylphosphonate). A Curie behavior (δ ∝ 1/T) is observed for the Cu_9_ and Cu_7_ rings, which display large variations with temperature becoming less paramagnetically shifted at higher temperatures (Figures S32–S34). The δ values of all other Cu_*x*_ rings show only slight variations with temperature, except for one of the OH signal of the Cu_12_ ring in Cu_6+12+9_PhPO_3_, which shows an anti-Curie behavior becoming more paramagnetically shifted at higher temperatures.

The thermal stability of phosphonate nanojars is strikingly different from those of the carbonate and sulfate analogues. At ambient temperature, the Cu_n_CO_3_ mixture contains larger amounts of Cu_6+12+9_CO_3_ and Cu_8+14+9_CO_3_, smaller amounts of Cu_7+13+9_CO_3_ and Cu_8+13+8_CO_3_, and no Cu_6+12+10_CO_3_. On heating in a DMSO-*d*_6_ solution, the Cu_8+14+9_CO_3_ and Cu_7+13+9_CO_3_ nanojars gradually decompose/rearrange and give rise to a mixture of mostly Cu_8+13+8_CO_3_ and Cu_6+12+9_CO_3_ nanojars at 150 °C.^39^ The Cu_*n*_SO_4_ mixture, which contains larger amounts of Cu_6+12+10_SO_4_ and Cu_8+14+9_SO_4_ and smaller amounts of the other nanojar sizes at ambient temperature, gives rise to a mixture of mostly Cu_8+14+9_SO_4_ and small amounts of Cu_8+13+8_SO_4_ at 150 °C.^43b^ In contrast, the different nanojars in the Cu_*n*_RPO_3_ mixtures appear to be equally robust, as the composition of the mixtures shows very little changes on heating to 150 °C (except for the Cu_6+12+10_PhPO_3_ nanojar, which decomposes by 60 °C). This observation is consistent with the fact that the Cu_*n*_RPO_3_ mixtures are not affected by NH_3_.

### ^31^P NMR Spectroscopy

**Figure 13:**
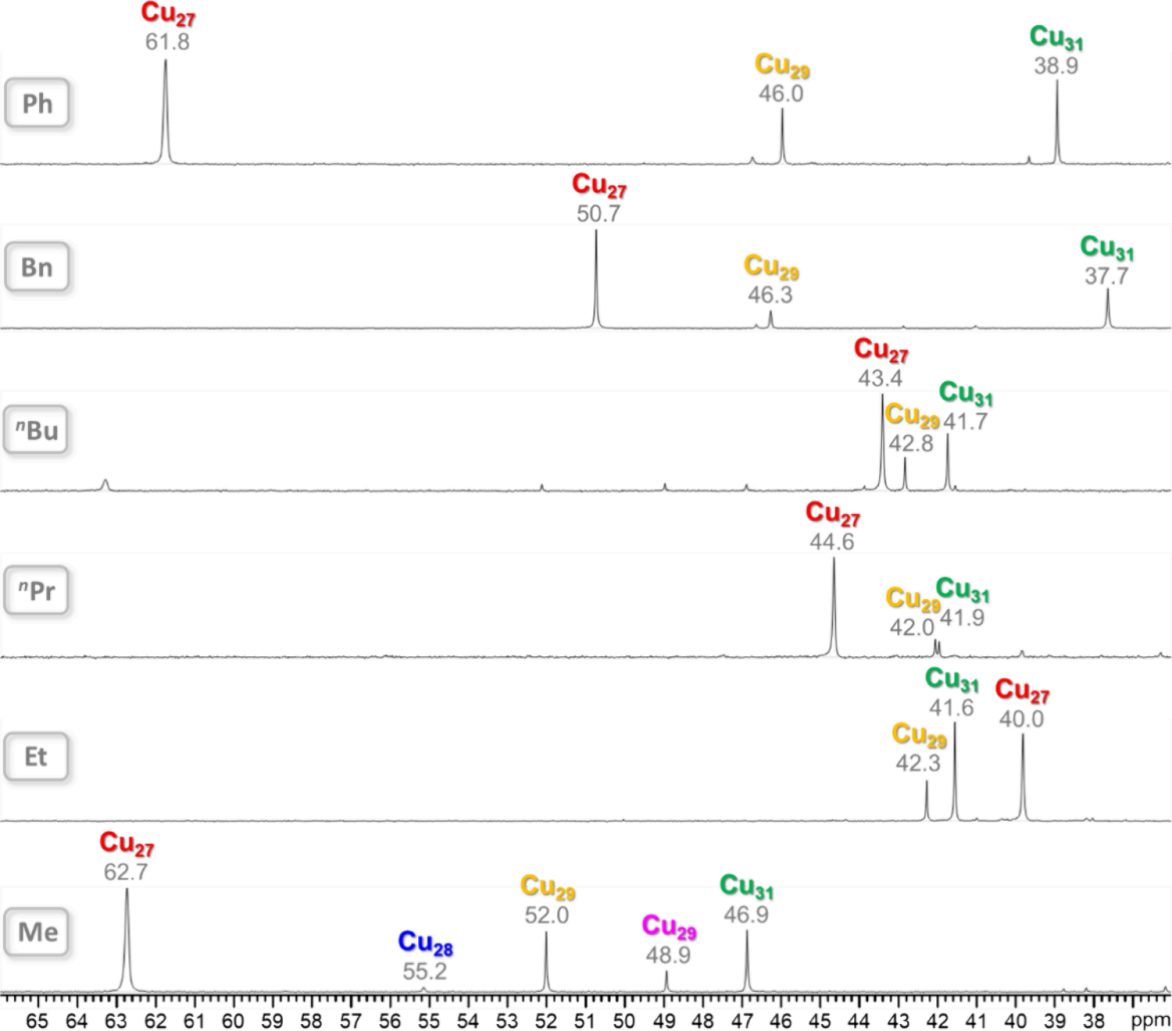
^31^P NMR spectra of the Cu_n_RPO_3_ (Cu_n_; *n* = 27–31; R = Me, Et, ^*n*^Pr, ^*n*^Bu, Bn, and Ph) nanojar mixtures in DMSO-*d*_6_ at ambient temperature. Chemical shift values (ppm) are shown under the nanojar symbols. Color code for the Cu_29_ nanojars: yellow, Cu_7+13+9_; magenta, Cu_8+13+8_. Assignments were made based on correlations with ESI-MS and ^1^H NMR spectra.

In the case of the (Bu_4_N)_2_RPO_3_ salts, prepared in situ by dissolving stoichiometric amounts of Bu_4_NOH (1 M in H_2_O) and the corresponding phosphonic acid in DMSO-*d*_6_, the ^31^P chemical shifts follow a trend that reflects the basicity of the phosphonate ion (Me: 13.4 ppm; Et: 19.1 ppm; ^*n*^Bu: 23.8 ppm; Bn: 16.1 ppm; Ph: 6.5 ppm). In contrast, no correlation with basicity is found for the corresponding hyperfine shifts in the different Cu_*n*_RPO_3_ nanojar species (Figure 13). For example, in the case of Cu_31_RPO_3_, the δ values are 46.9 (Me), 41.6 (Et), 41.7 (^*n*^Bu), 37.7 (Bn), and 38.9 (Ph) ppm. Yet, the average distance from the P atom to the Cu atoms in Cu_31_RPO_3_ (R = Me, Et, Bn, and Ph) is rather consistent (5.809(3), 5.842(3), 5.844(2), and 5.840(1) Å in **3**, **4**, **8**, and **10**, respectively) (Table S16). An even more disparate trend is observed in the case of Cu_27_RPO_3_, with δ values of 62.7 (Me), 40.0 (Et), 43.4 (^*n*^Bu), 50.7 (Bn), and 61.8 (Ph) ppm. Therefore, the origin of the observed differences in the δ values must be a combination of the basicity of the phosphonate anion (related to the electron donating or withdrawing ability of its R group) and the different spin densities on the various Cu_31_RPO_3_ nanojars caused by structural changes in their corresponding framework (as also indicated by the ^1^H NMR studies). Nevertheless, there seems to be a correlation between the observed hyperfine shifts and the average P···Cu distance in different nanojars with the same RPO_3_^2–^ anion (except for R = Et). For example, the δ values for Cu_27_MePO_3_ (ave. P···Cu: 5.546(2) Å), Cu_7+13+9_MePO_3_ (ave. P···Cu: 5.672(2) Å), and Cu_31_MePO_3_ (ave. P···Cu: 5.809(3) Å) are 62.7, 52.0, and 46.9 Å, respectively, showing decreasing paramagnetic shift with increasing average P···Cu separation.

### UV–Vis Spectroscopy

**Figure 14:**
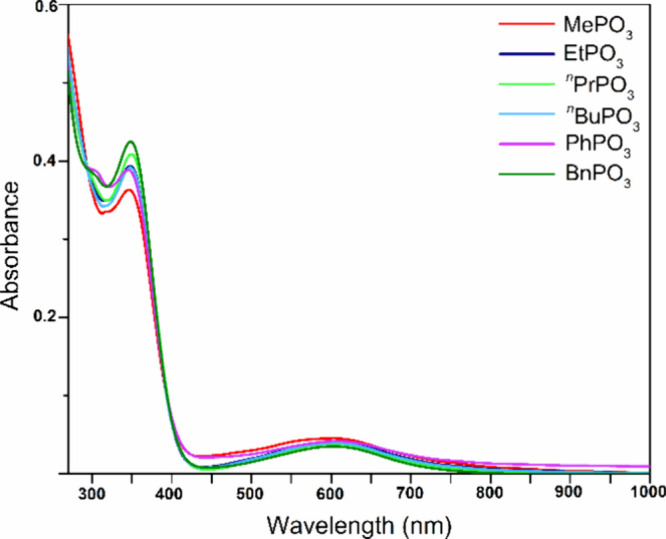
UV–vis spectra of Cu_n_RPO_3_ (*n* = 27–31; R = Me, Et, ^*n*^Pr, ^*n*^Bu, Bn, and Ph) in THF (20 μM).

The UV–vis spectra of Cu_n_RPO_3_ (*n* = 27–31; R = Me, Et, ^*n*^Pr, ^*n*^Bu, Bn, and Ph) in THF are virtually identical and display two peaks with absorption maxima at 345–349 and 602–608 nm, corresponding to charge-transfer and *d*–*d* transitions, respectively (with extinction coefficients of ε_347 nm_ = 2 × 10^4^ L mol^–1^ cm^–1^ and ε_605 nm_ = 2 × 10^3^ L mol^–1^ cm^–1^) (Figure 14). The λ_max_ values for the phosphonate nanojars are similar to the ones measured for the analogous nanojar mixtures with carbonate, sulfate, and chromate (λ_max_ = 350–351 and 599–602 nm).^42,43a,50^ Thus, the entrapped anion has minimal effects on the UV–vis absorption of the nanojar framework.

### Assessment of Phosphonate Binding Strength by Competitive Anion Binding

Given the inability of using host–guest titration (because a guest-free nanojar host cannot be obtained), the binding strength of phosphonates by nanojars was assessed similarly to other anions by using competitive binding experiments with Ba^2+^.^42,43a,50,51^ Neither in the case of heterogeneous conditions, when aqueous Ba(NO_3_)_2_ and water-immiscible 2-methyltetrahydrofuran (2-MeTHF) solutions of Cu_*n*_RPO_3_ nanojars (*n* = 27–31, R = Me or Bn) were vigorously stirred together, nor in the case of homogeneous conditions using 2-MeTHF-soluble barium dioctyl sulfosuccinate, Ba(DOSS)_2_, was any precipitate of a barium salt or a nanojar degradation product observed. Furthermore, ESI-MS analyses of the 2-MeTHF solutions after Ba^2+^ treatment show no nanojar degradation products either nor any significant changes in nanojar composition, confirming the strong binding of phosphonates by the different nanojar species.

## Conclusions

In summary, we demonstrated that nanojars can bind not only small inorganic oxoanions but also their organic derivatives bearing aliphatic or aromatic substituents, such as phosphonates (RPO_3_^2–^). ESI-MS analysis shows that in solution, the favored nanojar sizes are Cu_31_ and Cu_27_ followed by Cu_29_, whereas Cu_28_ and Cu_30_ are only observed occasionally and in small amounts. ^1^H and ^31^P NMR studies offer further details about structural isomers in the case of Cu_29_ and indicate that Cu_7+13+9_ is formed almost exclusively. In contrast to its carbonate- and sulfate-incarcerating analogues, VT ^1^H NMR studies reveal that the differently sized phosphonate-incarcerating nanojars are much more robust on heating to 150 °C in a DMSO-*d*_6_ solution, and with the exception of Cu_28_, which decomposes by 60 °C, resist conversion to a preferred nanojar size (Cu_8+13+8_ and Cu_8+14+9_ in the case of carbonate and sulfate, respectively). Similarly, NH_3_ treatment has a negligible effect on the Cu_*n*_RPO_3_ mixtures (except for Cu_28_), whereas the Cu_*n*_CO_3_ and Cu_*n*_SO_4_ mixtures are converted into Cu_27_CO_3_ and Cu_31_SO_4_.

While ^1^H NMR spectroscopy offers insight into the structure and magnetism of the nanojar host, ^31^P NMR spectroscopy probes the incarcerated phosphonate guest. The hyperfine shift of the P atom appears to be very sensitive to small changes in the magnetic environment, as varying the organic substituent leads to very large changes in the corresponding chemical shifts, not correlated with the basicity of the P atom bearing different R groups. Minor differences in the structure of the nanojar framework, which nevertheless cause significant changes in magnetism, must account for these observations. A similar inconsistency was also observed between the expected deshielding of the OH hydrogen atoms of certain Cu_*x*_ rings and the basicity of the different RPO_3_^2–^ anions involved in H-bonding.

Structural investigations by single-crystal X-ray crystallography on 12 different phosphonate nanojars (with Cu_6+12+9_, Cu_6+12+10_, Cu_7+13+9_, and Cu_8+14+9_ ring combinations, and R = Me, Et, ^*n*^Pr, ^*n*^Bu, ^*n*^C_12_, Bn, and Ph) reveal that only the hydrophilic group of phosphonates (PO_3_^2–^) needs to be buried within the nanojar cavity for efficient binding, while the organic group strings on the outside. Within the OH-lined cavity of the nanojars, the PO_3_^2–^ moiety is oriented toward the smaller Cu_*x*_ side ring (*x* = 6–8) and is bound by a multitude of hydrogen bonds (10–14 bonds with O···O distances shorter than 3.2 Å). Conversely, the organic substituent pokes out through the larger Cu_*x*_ side ring (*x* = 9 or 10). The Cu_8_ ring appears to be unfavorable for accommodating the organic group, as the Cu_8+13+8_ ring combination is only observed in the case of R = Me, in small amounts. The Cu_9_ ring, however, accommodates organic moieties as bulky as Ph, but not *t*-Bu directly bound to the P atom. Despite the larger size of the Cu_10_ ring, the Cu_6+12+10_ ring combination is again elusive and is observed in only small amounts in the case of R = Ph.

For the first time, cocrystallization of nanojars of different sizes by positional disorder has been observed. Thus, Cu_8+14+9_EtPO_3_ is cocrystallized with Cu_7+13+9_EtPO_3_ in a 95/5 ratio in **5** (sharing the Cu_9_ ring), and Cu_6+12+9_PhPO_3_ is cocrystallized with Cu_6+12+10_PhPO_3_ in 82/18 and 91/9 ratios in the two crystallographically independent nanojar units of **9** (sharing both the Cu_6_ and Cu_12_ rings). In spite of the H-bond donor-rich cavity of nanojars, which can often bind the entrapped anion in different orientations, the PO_3_^2–^ anion is only found disordered in **5** and **6** (Cu_7+13+9_^*n*^BuPO_3_). In **9**, the occupancies of the two different positions of the PO_3_^2–^ anion correlate with the occupancies of the Cu_6+12+9_ and Cu_6+12+10_ nanojars.

The studies of phosphonate nanojars led to the discovery of a novel motif in nanojar chemistry, nanojar clamshells, which consist of two nanojar units tethered by two adjacent μ_4_-RPO_3_ (R = Et or ^*n*^Pr) phosphonate anions. This is a surprising result given that they could not be detected in any significant amounts in solution by ESI-MS. In contrast to regular nanojars, nanojar clamshells produce much higher quality crystal structures with less disorder and smaller anisotropic displacement parameters, suggesting better molecular packing. These more robust crystal lattices might drive the formation of nanojar clamshells from phosphonate-substituted nanojars (which have indeed been observed in small amounts in solution by ESI-MS) upon crystallization. Considering that nanojar clamshells double the phosphonate-binding capacity of nanojars and could also accommodate bisphosphonates in the clamshell cavity, future studies will focus on their rational synthesis and further characterization with an aim at developing liquid–liquid extraction agents for phosphonates.

## Experimental Section

### General Information

All commercially available chemicals were used as received (solvents are ACS- or HPLC-grade, and THF is inhibited with 250 ppm of BHT). Cu(NO_3_)_2_·2.5H_2_O (ACS reagent, 98%), NaOH (ACS reagent, 97%), and phenylphosphonic acid (98%) were purchased from Sigma-Aldrich, pyrazole (99%) and *n*-propylphosphonic acid (95%) from Oakwood Chemical, ^*n*^Bu_4_NOH (HPLC grade, 1.0 M in H_2_O) and phosphonic acids (methyl 98%, ethyl 98%+, *n*-butyl 98%, *t*-butyl 98%, *n*-dodecyl 95%, benzyl 97%) from Thermo Scientific, and *t*-butylphosphonic acid (98%) from Acros Organics. Deionized water was freshly boiled and cooled to room temperature under *N*_2_(g). (Bn_3_MeN)NO_3_ and Ba(DOSS)_2_ were prepared according to the published procedures.^38,51^ Gaseous NH_3_ was generated by gently heating a 30% aqueous NH_3_ solution. The synthesis and reactions of nanojars were carried out under a *N*_2_(g) atmosphere. NMR spectra were collected on a Jeol JNM-ECZS (400 MHz) instrument, and UV–vis measurements were carried out on a Shimadzu UV-1650PC spectrophotometer. ^31^P NMR spectra (162 MHz) were recorded in NMR tubes with fused, coaxial glass inserts, and the corresponding chemical shifts are referenced to H_3_PO_4_ (85% in H_2_O). Because phosphonate nanojars have only one single P atom per molecule, their phosphorus content is very low (0.6% in the larger Cu_31_BnPO_3_ to 0.7% in the smaller Cu_27_MePO_3_). Therefore, long acquisition times of up to 3 days are needed for a good signal-to-noise ratio (especially for the less abundant nanojar species).

### Synthesis of [*trans*-Cu^II^(μ-OH)(μ-pz)]_∞_

Cu(NO_3_)_2_·2.5H_2_O (16.250 g, 0.0700 mol) was dissolved in H_2_O (300 mL) in a 1 L round-bottom flask. A solution of pyrazole (4.760 g, 0.0700 mol) and NaOH (5.600 g, 0.140 mol) in H_2_O (300 mL) was added dropwise to this solution under stirring, which resulted in the immediate formation of a dark blue/purple precipitate. The mixture was stirred overnight and filtered the next day. The dark blue/purple solid, which is insoluble in all common solvents, was washed thoroughly with water, followed by drying in air and then in high vacuum. Yield = 9.808 g (95%).

### Synthesis of (Bu_4_N)_2_[RPO_3_⊂{Cu(OH)(pz)}_n_] (Cu_n_RPO_3_; *n* = 27–31)

For method A, Cu(NO_3_)_2_·2.5H_2_O (200 mg, 0.859 mmol) and pyrazole (58 mg, 0.859 mmol) are dissolved in THF (10 mL). A solution of ^*n*^Bu_4_NOH (1 M in H_2_O, 3.439 mL, 3.439 mmol) and the corresponding phosphonic acid (0.859 mmol) in THF (10 mL) is added dropwise under stirring. The resulting deep blue solution is stirred for 10 min and then slowly added to water (300 mL) under stirring. The dark blue precipitate is filtered out, washed with water, and dried under high vacuum. Yields vary between 84 and 99%, except for R = Bn (52%) and R = ^*n*^C_12_ (17%). For method B, [*trans*-Cu^II^(μ-OH)(μ-pz)]_∞_ (0.2000 g, 1.355 mmol), ^*n*^Bu_4_NOH (1 M in H_2_O, 1.355 mL, 1.355 mmol), and the corresponding phosphonic acid (1.355 mmol) were added to toluene (15 mL) and the mixture was refluxed at 105 °C overnight. After cooling, the solid residue was filtered out and rinsed with toluene, yielding a dark blue filtrate. Then, the solvent was evaporated under a vacuum, and the solid product was washed with water to remove the excess ^*n*^Bu_4_N^+^ salts. Yields are similar to those obtained using Method A.

### Treatment of Cu_*n*_RPO_3_ with NH_3_

Cu_*n*_RPO_3_ (50 mg) was dissolved in THF (10 mL), and gaseous NH_3_ was slowly bubbled through the resulting solution for 20 min. Then, the flask was stoppered, sealed with Parafilm, and left standing for 7 days. The solution was filtered, and the solvent was evaporated to give a dark blue residue (43–46 mg).

### Competitive Anion Binding under Heterogeneous Conditions

A clear, blue solution was prepared by dissolving Cu_*n*_RPO_3_ nanojars (*n* = 27–31, R = Me or Bn; ∼0.02 mmol) in 2-MeTHF (20 mL). This solution was then transferred onto a solution of Ba(NO_3_)_2_ (0.0104 g, 0.04 mmol) in H_2_O (20 mL) by using a cannula. The mixture was stirred vigorously for an hour, followed by separation of the two layers and analysis of the 2-MeTHF layer by ESI-MS.

### Competitive Anion Binding under Homogeneous Conditions

The experiments were conducted similarly to the heterogeneous setup, using 2-MeTHF (20 mL) solutions for both Cu_n_RPO_3_ and Ba(DOSS)_2_ (0.0198 g, 0.02 mmol).

### Mass Spectrometry

Mass spectrometric analysis of the nanojars was performed with a Waters Synapt G1 HDMS instrument using electrospray ionization (ESI). Solutions (10^–4^–10^–5^ M) were prepared in CH_3_CN using either solids or aliquots taken from solutions. Samples were infused by a syringe pump at 5 μL/min, and nitrogen was supplied as the nebulizing gas at 500 L/h. The electrospray capillary voltage was set to −2.5 or +2.5 kV, with a desolvation temperature of 110 °C. The sampling and extraction cones were maintained at 40 and 4.0 V, respectively, at 80 °C.

### X-ray Crystallography

Single-crystals of **1**–**12** were grown at room temperature by solvent vapor diffusion using the solvents indicated in the Structural Analysis section above. Once removed from the mother liquor, the crystals are extremely sensitive to solvent loss at ambient conditions and were quickly mounted under a cryostream (150 K) to prevent decomposition. X-ray diffraction data were collected from a single-crystal mounted atop a MiTeGen micromesh mount under Fomblin oil with Bruker AXS D8 Quest diffractometers with either Photon III C14 charge-integrating and photon counting pixel array detector (CPAD) using a microfocus X-ray tube with multilayer optics for monochromatization with Cu *K*α (λ = 1.54178 Å) radiation or Photon II CPAD detector and graphite-monochromated Mo-*K*_α_ (λ = 0.71073 Å) radiation (for **7**). The data were collected using APEX4,^52^ integrated using SAINT,^53^ and scaled and corrected for absorption and other effects using SADABS.^54^ The structures were solved by employing direct methods using ShelXS^55^ or ShelXT^56^ and refined by full-matrix least-squares on *F*^*2*^ using ShelXL.^57^ C–H hydrogen atoms were placed in idealized positions and refined by using the riding model. Further refinement details and thermal ellipsoid plots (Figures S2–S13) are provided in the Supporting Information. Supramolecular features (angles and distances) were measured using OLEX2.^58^

## Supporting Information Available

Additional data for mass spectrometry, X-ray crystallography, and ^1^H NMR spectroscopy (PDF)
