# Supplementary material for: Supramolecular Binding of Phosphonate Dianions by Nanojars and Nanojar Clamshells
Source: Inorg Chem. 2024 Jul 18;63(30):14216–30. doi: 10.1021/acs.inorgchem.4c02386 (PMC11289757; doi:10.1021/acs.inorgchem.4c02386)
Supplement: Supplementary file 1 — ic4c02386_si_001.pdf [file ic4c02386_si_001.pdf]

Supporting Information for

Supramolecular Binding of Phosphonate Dianions by Nanojars and Nanojar Clamshells

Pooja Singh,[a] Matthias Zeller[b] and Gellert Mezei[a]*

[a] *Department of Chemistry, Western Michigan University, Kalamazoo, Michigan 49008, USA*

[b] *Department of Chemistry, Purdue University, West Lafayette, Indiana 47907, USA*

* Corresponding author. Email: gellert.mezei@wmich.edu

CONTENTS **PAGE**

1. Mass spectrometric data (Figure S1) S2

2. X-ray crystallographic data and refinement details (Figures S2−S19 and Tables S1–S53) S3–S76

3. NMR spectroscopic data (Figures S20−S34 and Table S54) S77–S97

4. References S97

1. MASS SPECTROMETRIC DATA

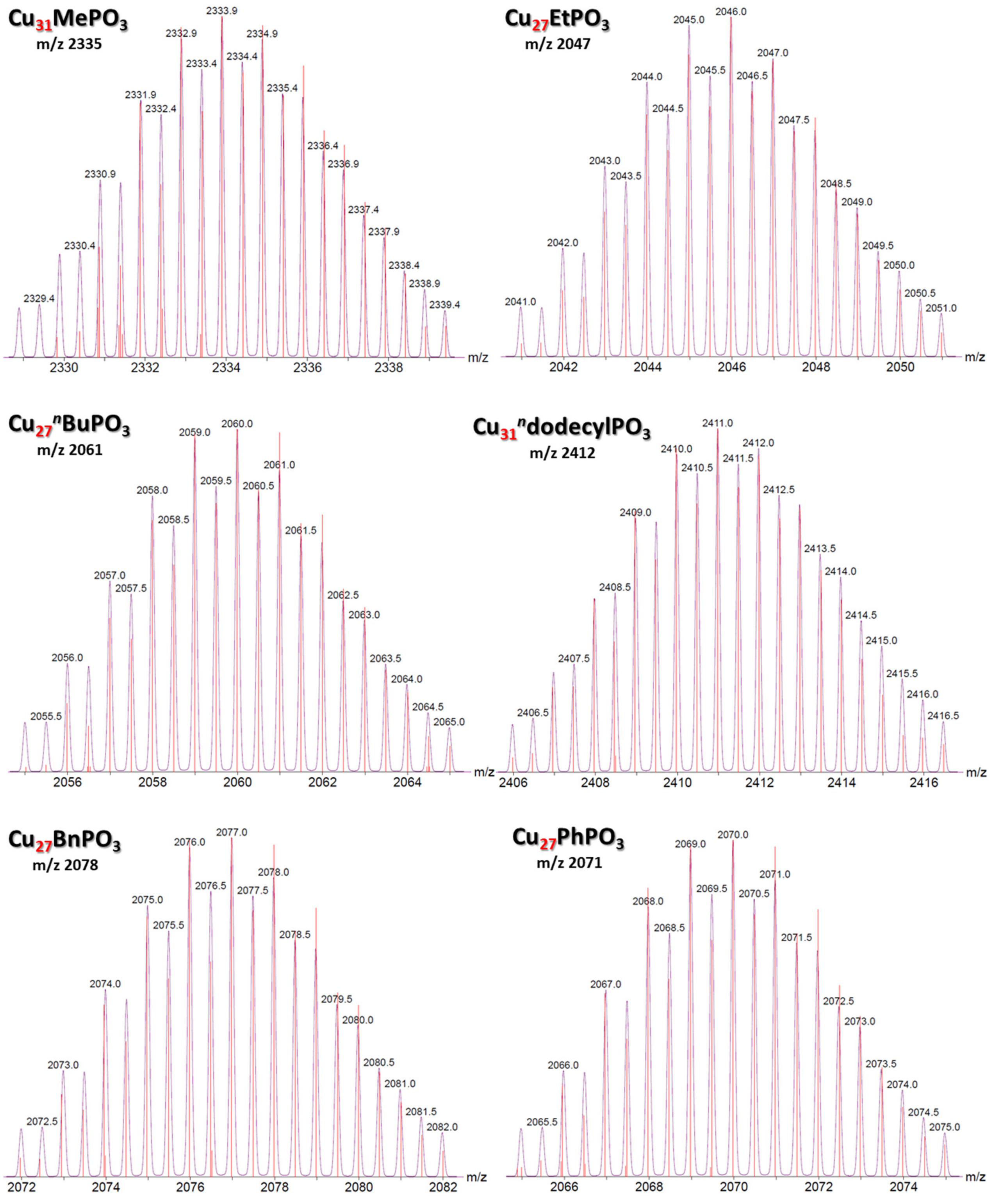

Figure S1. Examples of isotopic distributions observed (centroid) and predicted (continuum) for phosphonate nanojars.

2. X-RAY CRYSTALLOGRAPHIC DATA

1: Hydroxyl H atom positions were refined and O–H distances were restrained to 0.84(2) Å, respectively.

Two pyrazole ligands were refined as disordered. The disordered moieties were restrained to have similar geometries as another non-disordered pyrazole. U_{ij} components of ADPs for disordered atoms closer to each other than 2.0 Å were restrained to be similar. Subject to these conditions the occupancy ratio refined to 0.646(8)/0.354(8) (pyrazole of N1) and 0.552(11)/0.448(11) (pyrazole of N18).

One of the two Bu_4N^+ cations was refined as disordered. The two disordered moieties were restrained to have similar geometries as the other non-disordered cation. U_{ij} components of ADPs for disordered atoms closer to each other than 2.0 Å were restrained to be similar. Subject to these conditions the occupancy ratio refined to 0.595(5)/0.405(5).

Pockets and channels filled with solvate molecules were refined as occupied by extensively disordered dichlorobenzene as well as heptane molecules. All dichlorobenzene moieties were restrained to have similar geometries. Less well-defined dichlorobenzene moieties were restrained to be close to planar. Bonds and angles of heptane molecules were restrained to expected target values. U_{ij} components of ADPs for disordered atoms closer to each other than 2.0 Å were restrained to be similar. Occupancies were not strictly constrained to unity for all solvate occupied areas. Subject to these conditions the occupancy ratios refined to the values given in the atom_site tables.

2: Hydroxyl H atom positions were refined and O–H distances were restrained to 0.84(2) Å, respectively. H13O of the second nanojar (residue 3) was further restrained based on hydrogen bonding considerations.

Pockets and channels filled with solvate molecules were refined as occupied by extensively disordered dichlorobenzene as well as heptane molecules. All dichlorobenzene moieties were restrained to have similar geometries. Less well-defined dichlorobenzene moieties were restrained to be close to planar. Bonds and angles of heptane molecules were restrained to expected target values. U_{ij} components of ADPs for disordered atoms closer to each other than 2.0 Å were restrained to be similar. Occupancies were not strictly constrained to unity for all

solvate occupied areas. Subject to these conditions, the occupancy ratios refined to the values given in the atom_site tables.

The structure contains additional 2438 Å^3 of solvent accessible voids. No substantial electron density peaks were found in the solvent accessible voids and the residual electron density peaks are not arranged in an interpretable pattern. The structure factors were instead augmented via reverse Fourier transform methods using the SQUEEZE routine as implemented in the program Platon.[1] The resultant FAB file containing the structure factor contribution from the electron content of the void space was used together with the original HKL file for further refinement. The FAB file with details of the SQUEEZE results is appended to the CIF file. The SQUEEZE procedure corrected for 709 electrons within the solvent accessible voids.

3: Hydroxyl H atom positions were refined and O–H distances were restrained to 0.84(2) Å. Some hydroxyl H atom were further restrained based on hydrogen bonding considerations.

One pyrazole ligand was refined as disordered over two orientations. The two disordered moieties were restrained to have similar geometries. U_{ij} components of ADPs for disordered atoms closer to each other than 2.0 Å were restrained to be similar. Subject to these conditions, the occupancy ratio refined to 0.750(10)/0.250(10).

The Bu_4N^+ ions were refined as disordered. One was refined as disordered in a general position. The other was refined disordered over two positions, with one of the two positions being in conflict with its symmetry equivalent counterpart by an inversion center. The two moieties were thus set to be each half occupied. The ion in conflict with its symmetry equivalent is disordered with an anisole solvate molecule. Another anisole solvate molecule was refined as 1:1 disordered over two positions, with one of the two positions being in conflict with its symmetry equivalent counterpart by an inversion center. One of the positions is in conflict with the minor disordered pyrazole moiety. A third and fourth anisole were refined as partially occupied, and another as fully occupied.

All disordered Bu_4N^+ ion moieties were restrained to have similar geometries. All C–N bond distances of the ion in the general position were refined to be similar to each other. All anisole moieties were restrained to have similar geometries. Some anisole moieties were restrained to be close to planar (residues 7, 7B and 8) and/or to be close to isotropic (residues 6 and 8). U_{ij}

components of ADPs for disordered atoms closer to each other than 2.0 Å were restrained to be similar (but not for the atoms of the Bu_4N^+ and anisole disordered with each other). Subject to these conditions, the occupancy ratio refined to 0.626(12)/0.374(12) for the Bu_4N^+ ion in the general position. The occupancy of the partially occupied anisoles refined to 0.717(14)/0.291(11).

4. The ethyl group of the ethyl phosphonate anion is disordered over two orientations. The disordered moieties were restrained to have similar geometries. U_{ij} components of ADPs for disordered atoms closer to each other than 2.0 Å were restrained to be similar. Subject to these conditions, the occupancy ratio refined to 0.570(16)/0.430(16).

One butyl group of each of the two Bu_4N^+ cations were refined as disordered. The disordered moieties were restrained to have similar geometries as another better-defined butyl group. U_{ij} components of ADPs for disordered atoms closer to each other than 2.0 Å were restrained to be similar. Subject to these conditions the occupancy ratio refined to 0.69(2)/0.31(2) (C9 to C12 of residue 3) and 0.837(8)/0.163(8) (C13 to C16 of residue 4).

Several 1-methylnaphthalene solvate molecules are disordered. One is disordered across an inversion center and by general disorder, and several others by general disorder over two or more orientations. A hexane molecule is located on an inversion center and is disordered with a 1-methylnaphthalene moiety. All 1-methylnaphthalene moieties were restrained to have similar geometries and to be close to planar. 1-Methylnaphthalene C–C(methyl) distances were restrained to expected target values. U_{ij} components of ADPs for disordered atoms closer to each other than 2.0 Å were restrained to be similar. Subject to these conditions, the occupancy ratio refined to the values given in the _atom_site of the CIF file.

5. Hydroxyl H atom positions were initially refined and O–H distances were restrained to 0.84(2) Å while a damping factor was applied. Some hydroxyl H atom positions were further restrained based on hydrogen bonding considerations. In the final refinement cycles minor disordered hydroxyl H atoms were constrained to ride on their carrier oxygen atom and the damping factor was removed.

The nanojar consists of a major Cu_{31} and a minor Cu_{29} moiety. About half the nanojar atoms are shared between the two moieties. The others were refined as disordered. The ethyl

phosphonate anion was refined as three-fold disordered, with the B-moiety matching the occupancy of the Cu_{29} nanojar moiety. The minor nanojar moiety was restrained to have a similar geometry as an equivalent major moiety segment. Three minor moiety pyrazoles were restrained to be close to planar (of N27B, N31B, N33B). Two pairs of minor moiety atoms were restrained to have a minimum distance from each other (O18B_1 and O28B_1, and C40B_1 and C7B_6 of a disordered anisole). Minor moiety atom C56 is located in exactly the same position as O15, and the two atoms were given a common ADP. The three disordered ethyl phosphonate moieties were restrained to have similar geometries. U_{ij} components of ADPs for disordered atoms closer to each other than 2.0 Å were restrained to be similar. Subject to these conditions the occupancy ratio refined to 0.9465(8)/0.0535(8) in favor of the Cu_{31} nanojar. The phosphonate disorder refined to 0.622(9)/0.0535(8)/0.328(9).

One and two butyl groups of the two Bu_4N^+ cations were refined as disordered. The disordered moieties were restrained to have similar geometries as equivalent non-disordered moieties. U_{ij} components of ADPs for disordered atoms closer to each other than 2.0 Å were restrained to be similar. Subject to these conditions, the occupancy ratios refined to 0.531(14)/0.469(14) for C9–C12 of the cation of residue 3, to 0.711(11)/0.289(11) for C1–C4 of the cation of residue 4, and to 0.804(15)/0.196(15) for C5–C8 of the cation of residue 4.

Four anisole solvate molecules were refined, three of them as disordered: residue 5 as non-disordered, residue 6 as partially occupied and two-fold disordered (with the minor moiety matching the occupancy of the Cu_{29} nanojar), residue 7 as two-fold disordered, and residue 8 as three-fold disordered. All anisole moieties were restrained to have similar geometries. The minor moieties of residues 6 and 8 were restrained to be close to planar. The atom C1B of residue 8 was constrained to be planar. U_{ij} components of ADPs for disordered atoms closer to each other than 2.0 Å were restrained to be similar. Subject to these conditions, the occupancy rates refined to 0.853(9)/0.0535(8) (residue 6), 0.794(8)/0.206(8) (residue 7), and 0.581(3)/0.258(3)/0.161(3) (residue 8).

The structure contains additional 194 $Å^3$ of solvent accessible voids. No substantial electron density peaks were found in the solvent accessible voids (less than 1.5 electron per $Å^3$) and the residual electron density peaks are not arranged in an interpretable pattern. The structure factors were instead augmented via reverse Fourier transform methods using the SQUEEZE routine as implemented in the program Platon.[1] The resultant FAB file containing the structure

factor contribution from the electron content of the void space was used together with the original HKL file in the further refinement. The FAB file with details of the SQUEEZE results is appended to the CIF file. The SQUEEZE procedure corrected for 25 electrons within the solvent accessible voids.

6. Hydroxyl H atom positions were refined and O–H distances were restrained to 0.84(2) Å. Two pyrazole ligands were refined as disordered. The disordered moieties were restrained to have similar geometries as another better-defined pyrazole. U_{ij} components of ADPs for disordered atoms closer to each other than 2.0 Å were restrained to be similar. Subject to these conditions the occupancy ratio refined to 0.727(12)/0.273(12) (pyrazole of N33/N34) and 0.636(11)/0.364(11) (pyrazole of N35/N36).

The PO_3 unit was refined as rotationally disordered. The two disordered moieties were restrained to have similar geometries. U_{ij} components of ADPs for disordered atoms closer to each other than 2.0 Å were restrained to be similar. Subject to these conditions the occupancy ratio refined to 0.816(5)/0.184(5).

One butyl chain of each Bu_4N^+ cation was refined as disordered. The disordered moieties were restrained to have similar geometries as another better-defined butyl group. U_{ij} components of ADPs for disordered atoms closer to each other than 2.0 Å were restrained to be similar. Subject to these conditions, the occupancy ratio refined to 0.781(11)/0.219(11) (C13_3 to C16_3) and 0.59(2)/0.41(2) (C9_4 to C12_4).

A heptane solvate molecule was refined as disordered. The two disordered moieties were restrained to have similar geometries. U_{ij} components of ADPs for disordered atoms closer to each other than 2.0 Å were restrained to be similar. Subject to these conditions, the occupancy ratio refined to 0.66(2)/0.34(2).

The structure contains additional 3744 Å^3 of solvent-accessible voids occupied by excessively disordered chlorobenzene and heptane solvate molecules. The residual electron density peaks are not arranged in an interpretable pattern. The structure factors were instead augmented via reverse Fourier transform methods using the SQUEEZE routine as implemented in the program Platon.[1] The resultant FAB file containing the structure factor contribution from the electron content of the void space was used together with the original HKL file in further refinement.

The FAB file with details of the SQUEEZE results is appended to the CIF file. The SQUEEZE procedure corrected for 964 electrons within the solvent-accessible voids.

7. The alkyl chains of the *n*-dodecylphosphonate anion are disordered (except for the first two and four C atoms next to the P atom in the two symmetry-independent units). All their C–C bonds were restrained to be similar in length and the C–C–C bond angles were restrained to a target value of 2.50(2) Å. U_{ij} components of ADPs for disordered atoms closer to each other than 2.0 Å were restrained to be similar. Subject to these conditions the occupancy ratios refined to 0.334(8)/0.666(8) and 0.372(9)/0.628(9).

Four of the pyrazole ligands were refined as disordered (N27/18_1, N55/56_1, N31/32_3, N41/42_3). The disordered moieties were restrained to have similar geometries as other non-disordered pyrazoles. U_{ij} components of ADPs for disordered atoms closer to each other than 2.0 Å were restrained to be similar. Subject to these conditions, the occupancy ratios refined to 0.62(2)/0.38(2), 0.74(2)/0.26(2), 0.670(13)/0.330(13) and 0.48(3)/0.52(3).

Several butyl chains of the Bu_4N^+ countercations were refined as disordered. The disordered moieties were restrained to have similar geometries as other non-disordered butyl groups. U_{ij} components of ADPs for disordered atoms closer to each other than 2.0 Å were restrained to be similar. Subject to these conditions, the occupancy ratios refined to the values given in the atom_site tables.

Several 1,2-dichlorobenzene solvate molecules are disordered and/or ill-defined. All 1,2-dichlorobenzene moieties were restrained to have similar geometries. Less well-defined moieties were restrained to be close to planar and their atoms to be close to isotropic. C1B_12 was constrained to be planar. U_{ij} components of ADPs for disordered atoms closer to each other than 2.0 Å were restrained to be similar. A mild global anti-bumping restraint was applied. Subject to these conditions, the occupancy ratios refined to the values given in the atom_site tables.

8. Hydroxyl H atom positions were initially refined and O–H distances were restrained to 0.84(2) Å while a damping factor was applied. Some hydroxyl H atom positions were further restrained based on hydrogen bonding considerations. In the final refinement cycles minor

disordered hydroxyl H atoms were constrained to ride on their carrier oxygen atom and the damping factor was removed.

Two segments of the nanojar were refined as disordered. One ranged from Cu2 to Cu8 (including the hydroxyl and pyrazole moieties), the other was limited to one pyrazole (of N37/N38). Major moiety pyrazoles and the two pyrazoles of the second disordered segment were restrained to have a similar geometry as a well-defined non-disordered pyrazole. The minor large disordered moiety was restrained to have a similar geometry as the major one. U_{ij} components of ADPs for disordered atoms closer to each other than 2.0 Å were restrained to be similar. Subject to these conditions the occupancy ratio refined to 0.557(5)/0.443(5) and 0.560(14)/0.440(14), respectively.

The two Bu_4N^+ cations were refined as disordered. The disordered moieties were restrained to have similar geometries. U_{ij} components of ADPs for disordered atoms closer to each other than 2.0 Å were restrained to be similar. Subject to these conditions, the occupancy ratios refined to 0.636(6)/0.364(6) for the cation of residue 3, and to 0.561(8)/0.439(8) for the cation of residue 4.

The structure contains additional 2619 Å^3 of solvent accessible voids. No substantial electron density peaks were found in the solvent accessible voids (less than 1.5 electron per Å^3) and the residual electron density peaks are not arranged in an interpretable pattern. The structure factors were instead augmented via reverse Fourier transform methods using the SQUEEZE routine as implemented in the program Platon.[1] The resultant FAB file containing the structure factor contribution from the electron content of the void space was used together with the original HKL file in the further refinement. The FAB file with details of the SQUEEZE results is appended to the CIF file. The SQUEEZE procedure corrected for 1155 electrons within the solvent accessible voids.

9. Two crystallographically independent copper nanojar sites are present in the structure. Both consist of a mixture of Cu_{28} and Cu_{27} nanojars. Disorder is present only in the Cu-ring closest to the $PhPO_3$ anions, with one Cu(pz)(OH) unit missing for the smaller nanojar. Equivalent disordered moieties were restrained to have similar geometries. U_{ij} components of ADPs for disordered atoms closer to each other than 2.0 Å were restrained to be similar. Subject to these conditions, the occupancy ratio refined to 0.8242(15)/0.1758(15) for the first nanojar (residue

3) in favor of Cu_{28}, and to 0.9078(11)/0.0922(11) for the second nanojar (residue 4) in favor of Cu_{28}.

The PO_3 units are disordered by rotation. Major and minor moieties were restrained to have similar geometries. U_{ij} components of ADPs for disordered atoms closer to each other than 2.0 Å were restrained to be similar. P atoms were excluded from the disorder. Subject to these conditions, the occupancy ratio refined to 0.858(6)/0.142(6) for the first anion (residue 1), and to 0.885(5)/0.115(5) for the second (residue 2).

Four cation sites are present in the structure. Three consist of Bu_4N^+ cations with various kinds of disorder (disorder of one butyl group for residue 6, whole cation disorder of residues 7 and 9; atoms C7 and C8 of residue 7 were excluded from the disorder). The fourth (residue 8) is occupied by a mixture of Bu_4N^+ and $MeN(CH_2Ph)_3$. All Bu_4N^+ moieties were restrained to have similar geometries. Phenyl rings of $MeN(CH_2Ph)_3$ were restrained to resemble ideal hexagons with C–C distances of 1.39 Å (AFIX 66). N–C(benzyl) bond lengths were restrained to be similar and the C···C distances of the atoms around the N atom of $MeN(CH_2Ph)_3$ were restrained to be similar. C15 to C21 of this cation were restrained to be coplanar. The N–C(methyl) distance was restrained to a target value of 1.45(2) Å. U_{ij} components of ADPs for disordered atoms closer to each other than 2.0 Å were restrained to be similar. Subject to these conditions, the occupancy ratio refined to 0.621(15)/0.379(15) (disordered butyl group of residue 6), to 0.579(6)/0.421(6) for residue 7, to 0.466(2)/0.534(2) for residue 8 (in favor of NBu_4), and to 0.572(9)/0.428(9) for residue 9.

The remainder of the structure is made up of a mixture of 1,3-dimethoxybenzene and heptane solvent molecules. Equivalent disordered moieties were restrained to have similar geometries. Heptane C–C bond lengths and angles and some torsion angles were restrained to expected target values. 1,3-Dimethoxybenzene molecules were restrained to be close to planar. Where possible, occupancy rates were constrained to unity. U_{ij} components of ADPs for disordered atoms closer to each other than 2.0 Å were restrained to be similar. Subject to these conditions, the occupancy ratio refined to the values given in _atom_site table in the CIF.

10. Hydroxyl H atom positions were refined and O–H distances were restrained to 0.84(2) Å. Two pyrazole ligands were refined as disordered. The disordered moieties were restrained to have similar geometries as another not disordered pyrazole. U_{ij} components of ADPs for

disordered atoms closer to each other than 2.0 Å were restrained to be similar. Subject to these conditions, the occupancy ratio refined to 0.517(12)/0.483(12) for the pyrazole of N5/N6, and to 0.747(9)/0.253(9) for the pyrazole of N11/N12.

One of the two Bu_4N^+ cations was refined as disordered. The two disordered moieties were restrained to have similar geometries as the other not disordered Bu_4N^+ cation. U_{ij} components of ADPs for disordered atoms closer to each other than 2.0 Å were restrained to be similar. Subject to these conditions, the occupancy ratio refined to 0.624(5)/0.376(5).

A large part of the 1,3-dimethoxybenzene solvent molecules are disordered. One solvent molecule is disordered across an inversion center and is additionally disordered. Other 1,3-dimethoxybenzene solvent molecules exhibit general disorder. All 1,3-dimethoxybenzene solvent molecules (including three without disorder) were restrained to have similar geometries. The benzene ring of a minor moiety unit was constrained to resemble an ideal hexagon with C–C bond distances of 1.39 Å (AFIX 66). Occupancies of two-fold and some three-fold disordered molecules were constrained to sum up to unity, but full occupancy was not strictly enforced for all units and presence of diffuse additional electron density overlapping with partially occupied solvent molecules is evident. U_{ij} components of ADPs for disordered atoms closer to each other than 2.0 Å were restrained to be similar. Subject to these conditions, the occupancy ratios refined to the values given in the atom_site table of the CIF.

11. H atoms of the OH groups of the fused nanojar were refined and O–H distances were restrained to 0.84(2) Å, respectively. Water H atom positions were initially refined and O–H and H···H distances were restrained to 0.84(2) and 1.36(2) Å, respectively, and *t*-butanol H atom positions were initially allowed to rotate. Water and *t*-butanol H atom positions were further restrained based on hydrogen bonding considerations while a damping factor was applied. In the final refinement cycles, these H atoms were constrained to ride on their carrying oxygen atom (AFIX 3) and the damping factor was removed.

One of the two independent Bu_4N^+ ions was refined as disordered (whole cation disorder). The two disordered moieties were restrained to have similar geometries as the other not disordered Bu_4N^+ ion. U_{ij} components of ADPs for disordered atoms closer to each other than 2.0 Å were restrained to be similar. Subject to these conditions, the occupancy ratio refined to 0.756(4)/0.244(4).

Three 1-methylnaphthalene molecules were disordered (one over two orientations, two over three orientations). Another 1-methylnaphthalene molecule was refined as disordered with a *t*-butanol molecule across an inversion center. One *t*-butanol molecule was refined as disordered across a two-fold axis. A cluster solvate molecule was refined as extensively disordered with *t*-butanol molecules disordered over two or three orientations, and two partially occupied water molecules and one partially occupied 1-methylnaphthalene molecule disordered with the *t*-butanol molecules. All 1-methylnaphthalene moieties were restrained to have similar geometries and to be close to planar. U_{ij} components of ADPs for disordered atoms closer to each other than 2.0 Å were restrained to be similar. The atoms of the two molecules of residue 13 were also restrained to be close to isotropic. All *t*-butanol moieties were restrained to have similar geometries. O–C bond lengths were restrained to a target value of 1.446(20) Å. U_{ij} components of ADPs for disordered atoms closer to each other than 2.0 Å were restrained to be similar. Occupancies were constrained for each site to unity. Subject to these conditions, the occupancy rates of solvate molecules refined to the values given in the atom_site tables of the CIF.

12. Hydroxyl H atom positions were refined and O–H distances were restrained to 0.84(2) Å. One pyrazole ring (of N49/N50 of residue 1) was refined as disordered. The disordered moieties were restrained to have similar geometries as another non-disordered pyrazole ring. U_{ij} components of ADPs for disordered atoms closer to each other than 2.0 Å were restrained to be similar. Subject to these conditions, the occupancy ratio refined to 0.8963(17)/0.1037(17).

One propyl chain of one $^{n}PrPO_3$ anion (residue 4) was refined as disordered. The two disordered moieties were restrained to have similar geometries. U_{ij} components of ADPs for disordered atoms closer to each other than 2.0 Å were restrained to be similar. Subject to these conditions, the occupancy ratio refined to 0.770(9)/0.230(9).

Butyl chains of two of the four Bu_4N^+ cations were refined as disordered (one butyl chain as three-fold disordered for residue 8, two butyl chains as two-fold disordered for residue 10). The disordered moieties were restrained to have similar geometries. U_{ij} components of ADPs for disordered atoms closer to each other than 2.0 Å were restrained to be similar. Subject to these conditions, the occupancy ratios refined to 0.619(3)/0.1037(17)/0.278(3) for residue 8, and 0.568(9)/0.432(9) and 0.522(9)/0.478(9) for residue 10.

Several anisole solvate molecules were refined as disordered with each other. Several *t*-butanol and one more anisole solvent molecules were refined as disordered with each other. All anisole moieties were restrained to have similar geometries. U_{ij} components of ADPs for disordered anisole and *t*-butanol atoms closer to each other than 2.0 Å were restrained to be similar. Selected anisole moieties were restrained to be close to planar (moieties of residues 13B, 14, 14B, 15B and 21). The angles of the *t*-Bu methyl groups were restrained to be close to tetrahedral. *t*-Butanols were also restrained to be close to isotropic. For the *t*-butanol disorder, multiple overlaps are observed and no attempts were made to exactly match occupancy ratios and rates were refined with some occupancy constraints. Subject to these conditions, the anisole occupancy ratios refined to 0.675(3)/0.221(3) (RESI 12), 0.591(7)/0.409(7) (RESI 13), 0.771(7)/0.229(7) (RESI 14), and 0.717(6)/0.283(6) (RESI 15). The occupancies for the *t*-butanol and one anisole solvate molecules refined to 0.574(6) (RESI 16/17), 0.426(6) (RESI 18), 0.447(3) (RESI 19), 0.321(3) (RESI 20) and 0.232(3) (RESI 21, an anisole overlapping with *t*-butanols).

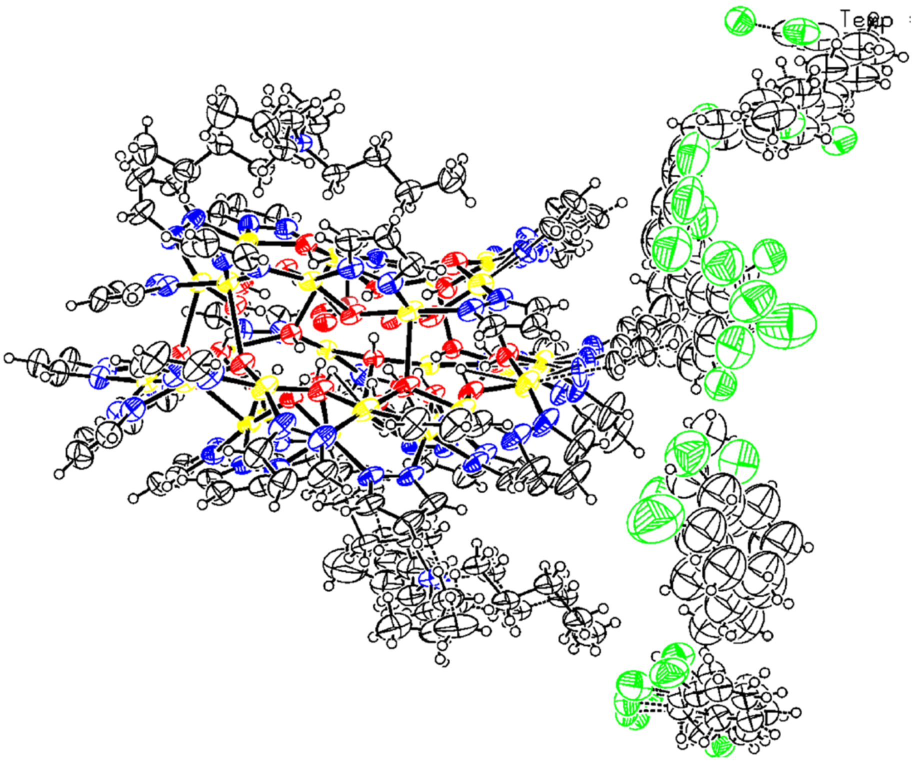

Figure S2. Thermal ellipsoid plot of the crystal structure of **1**.

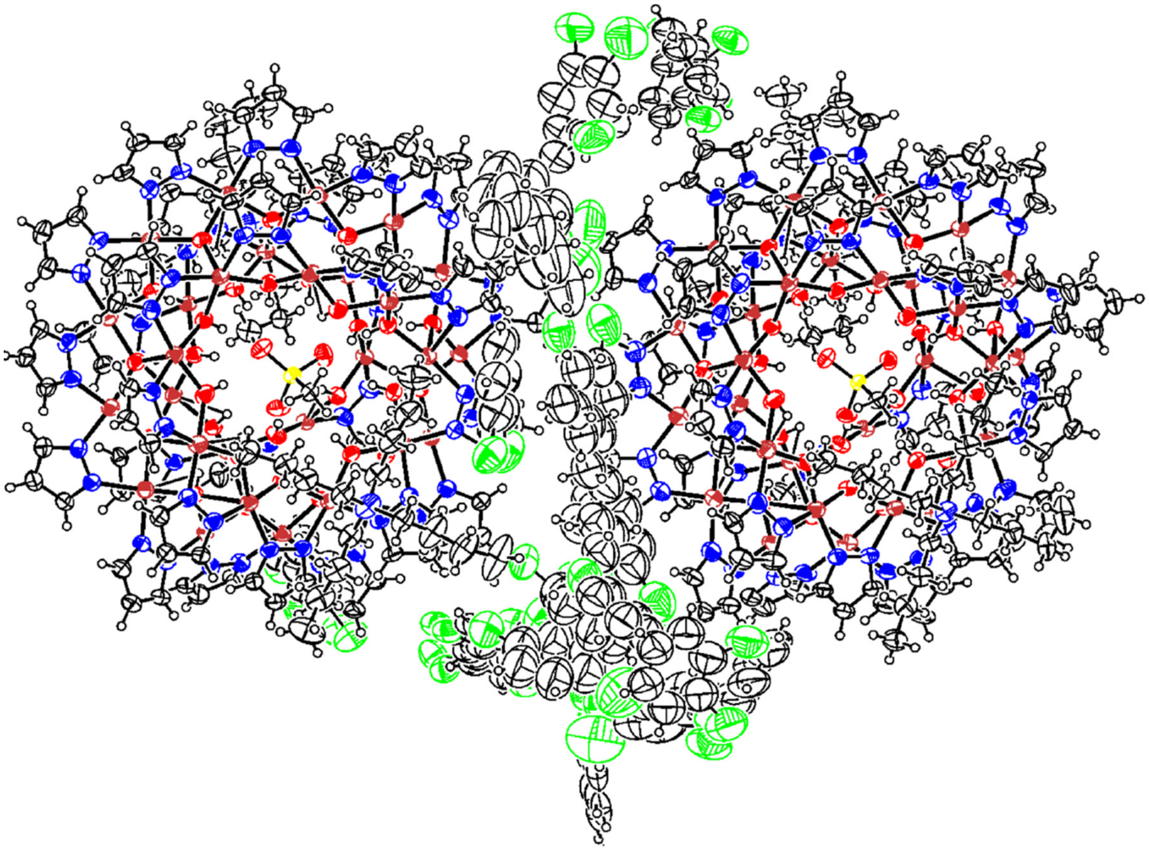

Figure S3. Thermal ellipsoid plot of the crystal structure of **2**.

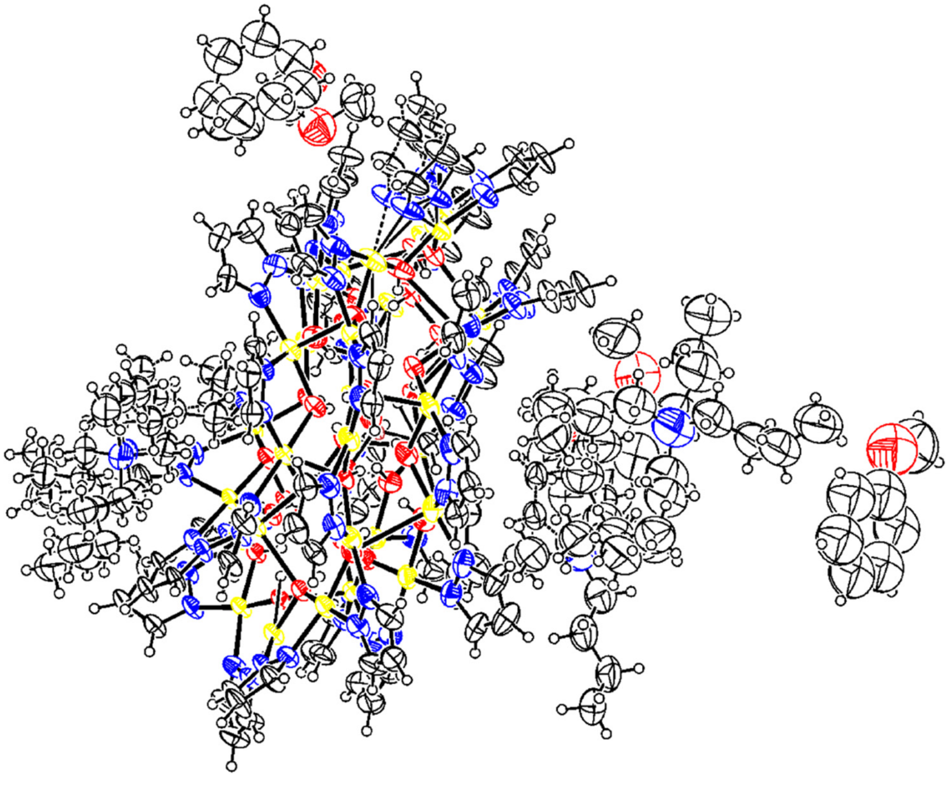

Figure S4. Thermal ellipsoid plot of the crystal structure of **3**.

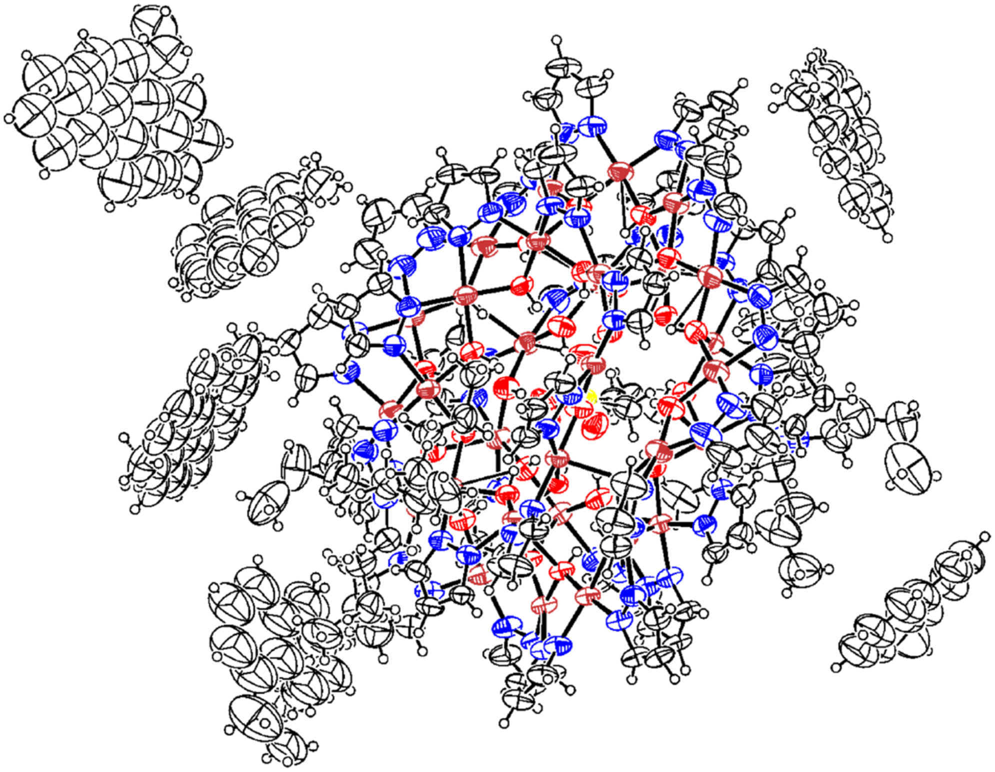

Figure S5. Thermal ellipsoid plot of the crystal structure of **4**.

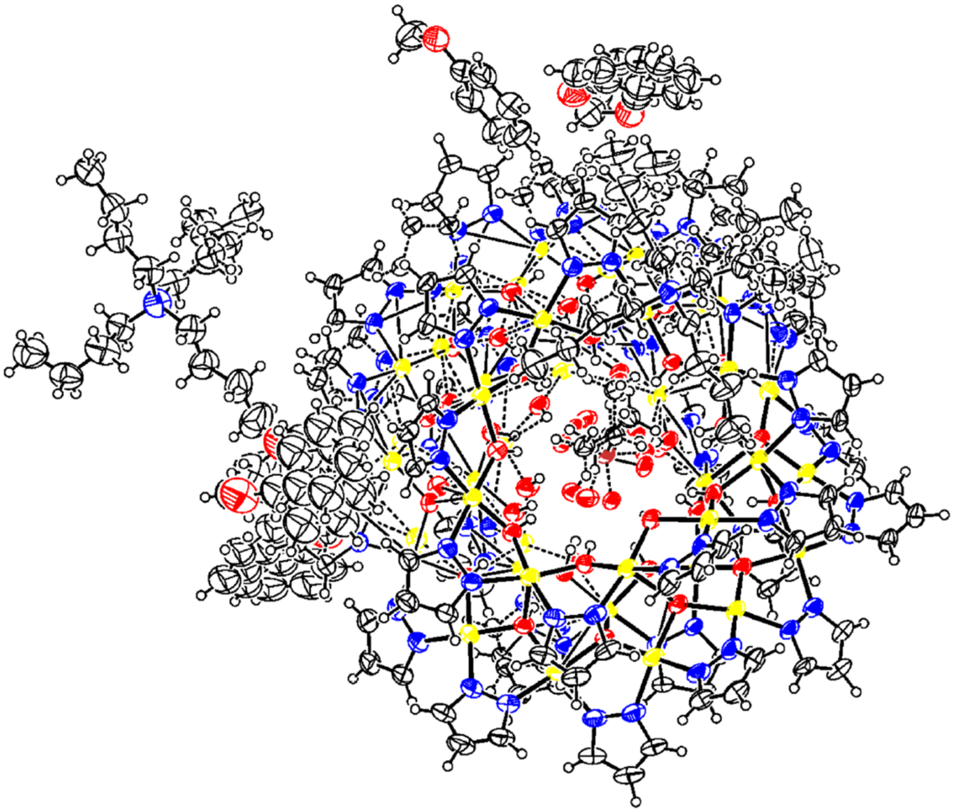

Figure S6. Thermal ellipsoid plot of the crystal structure of **5**.

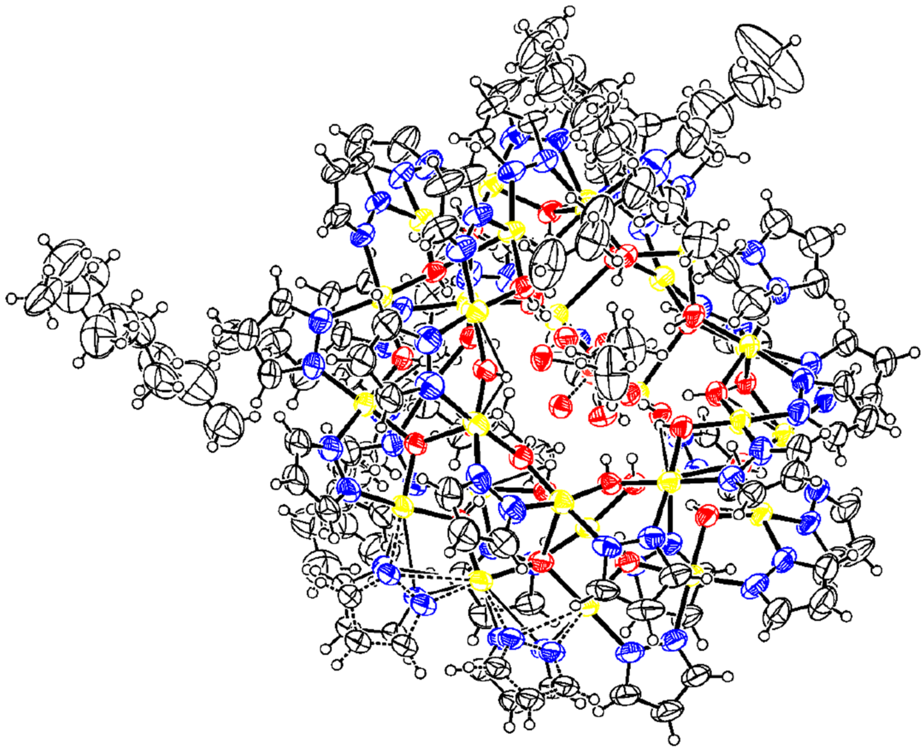

Figure S7. Thermal ellipsoid plot of the crystal structure of **6**.

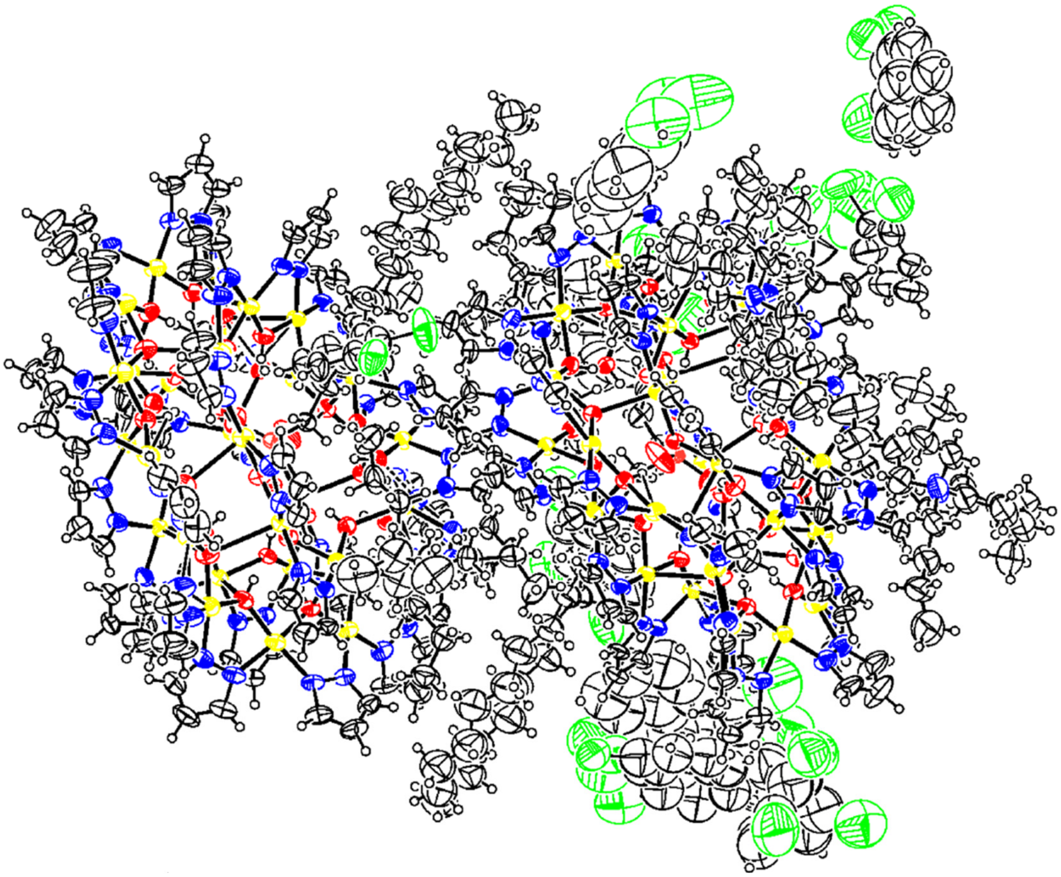

Figure S8. Thermal ellipsoid plot of the crystal structure of **7**.

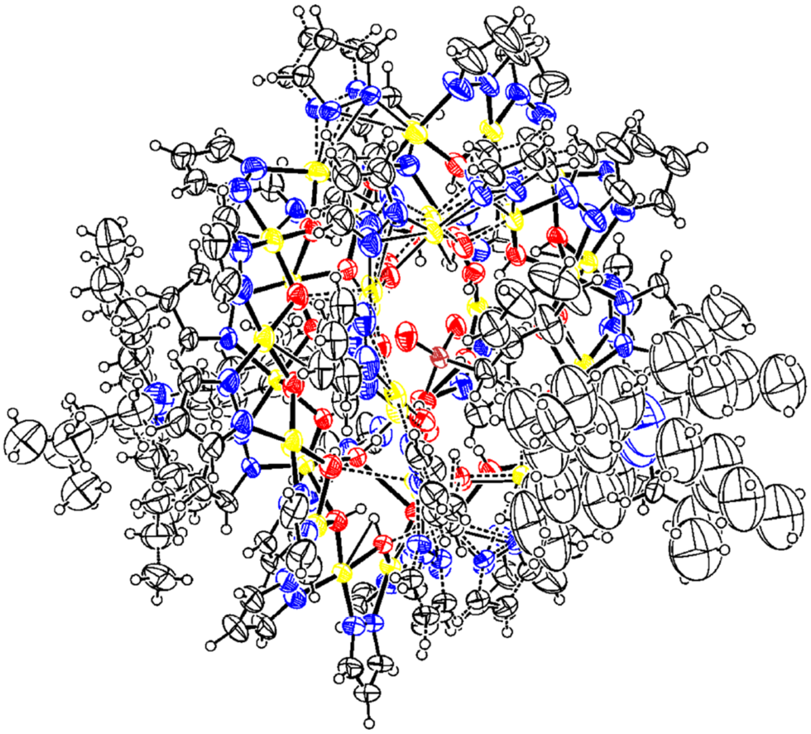

Figure S9. Thermal ellipsoid plot of the crystal structure of **8**.

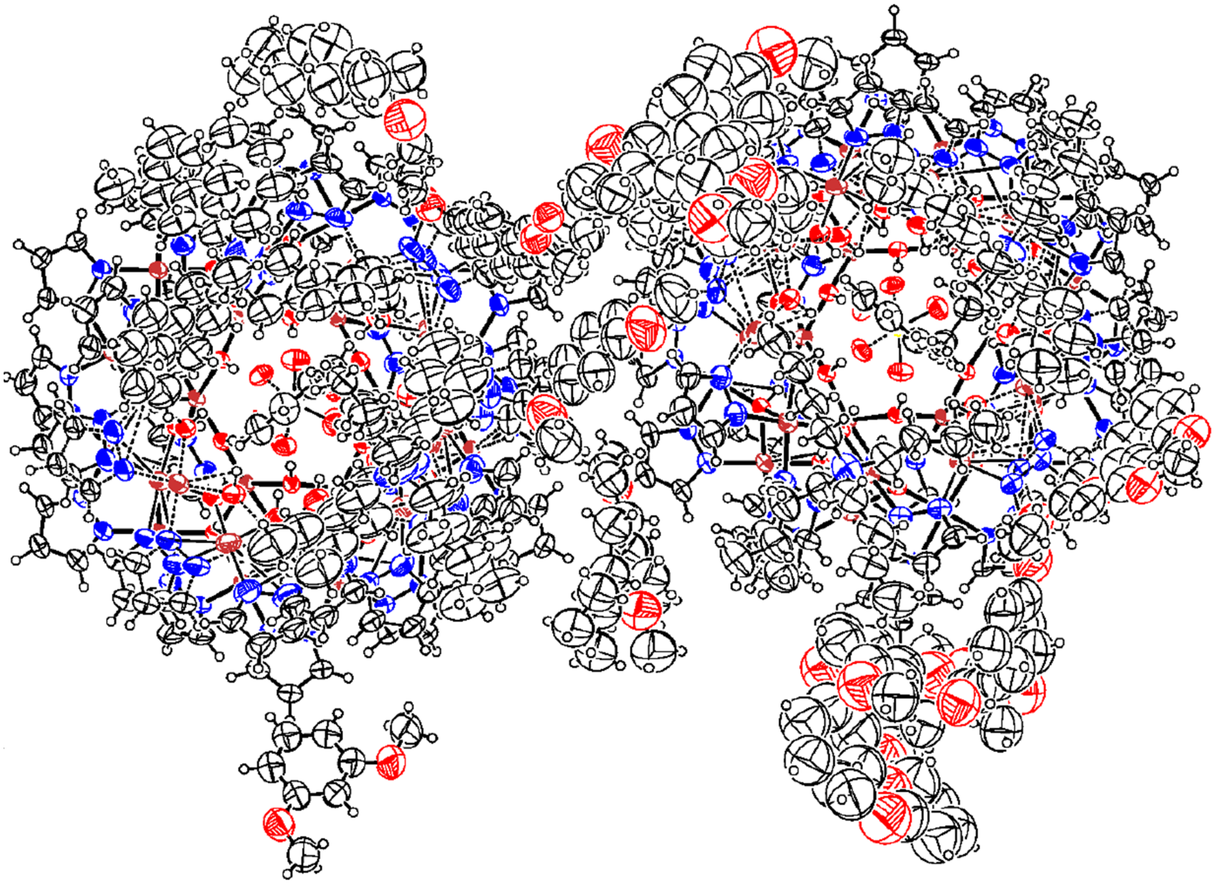

Figure S10. Thermal ellipsoid plot of the crystal structure of **9**.

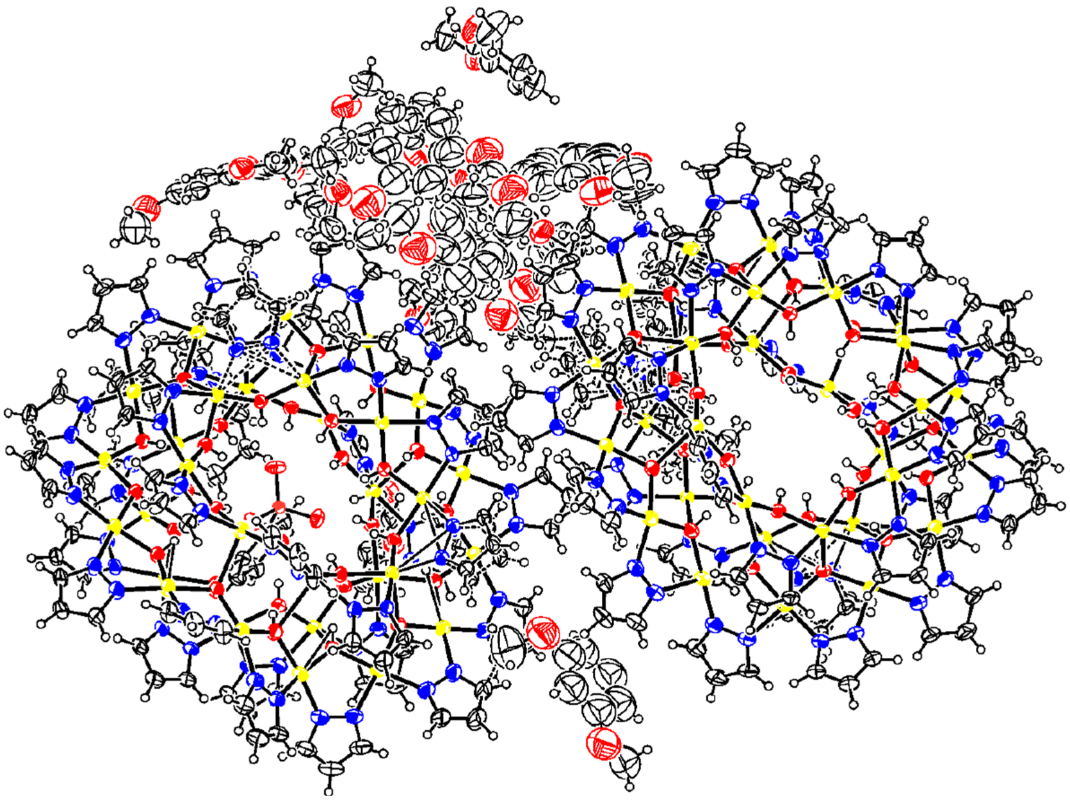

Figure S11. Thermal ellipsoid plot of the crystal structure of **10**.

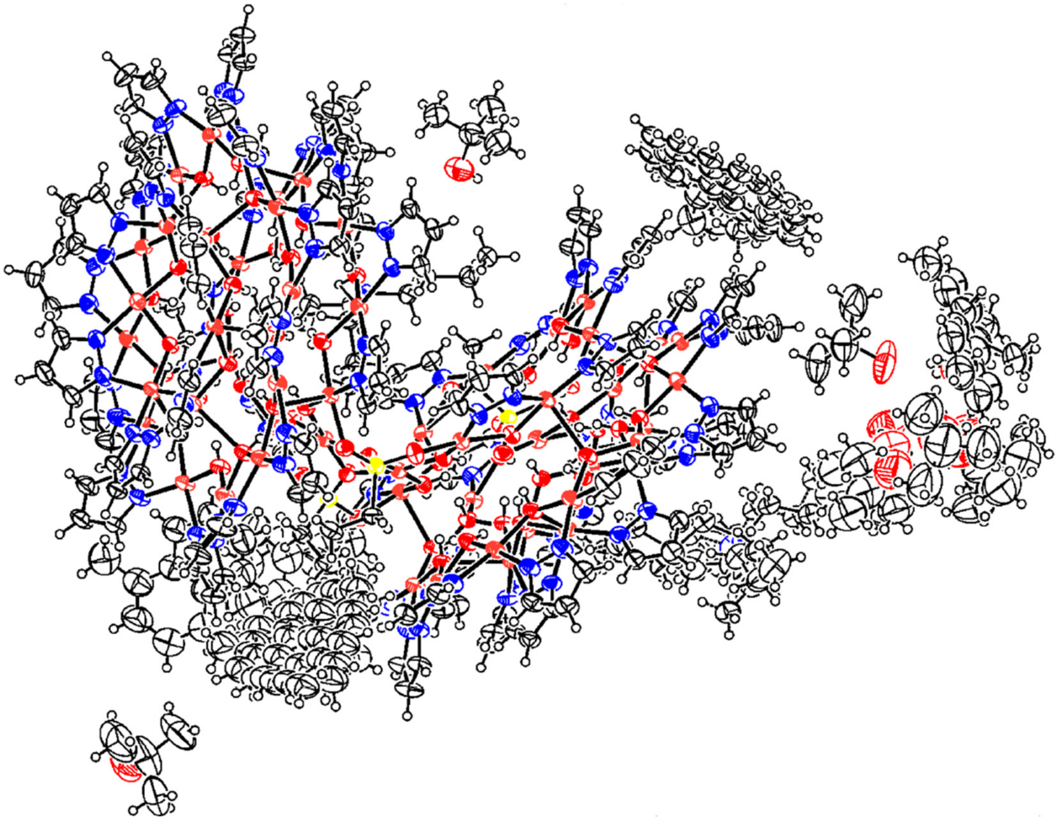

Figure S12. Thermal ellipsoid plot of the crystal structure of **11**.

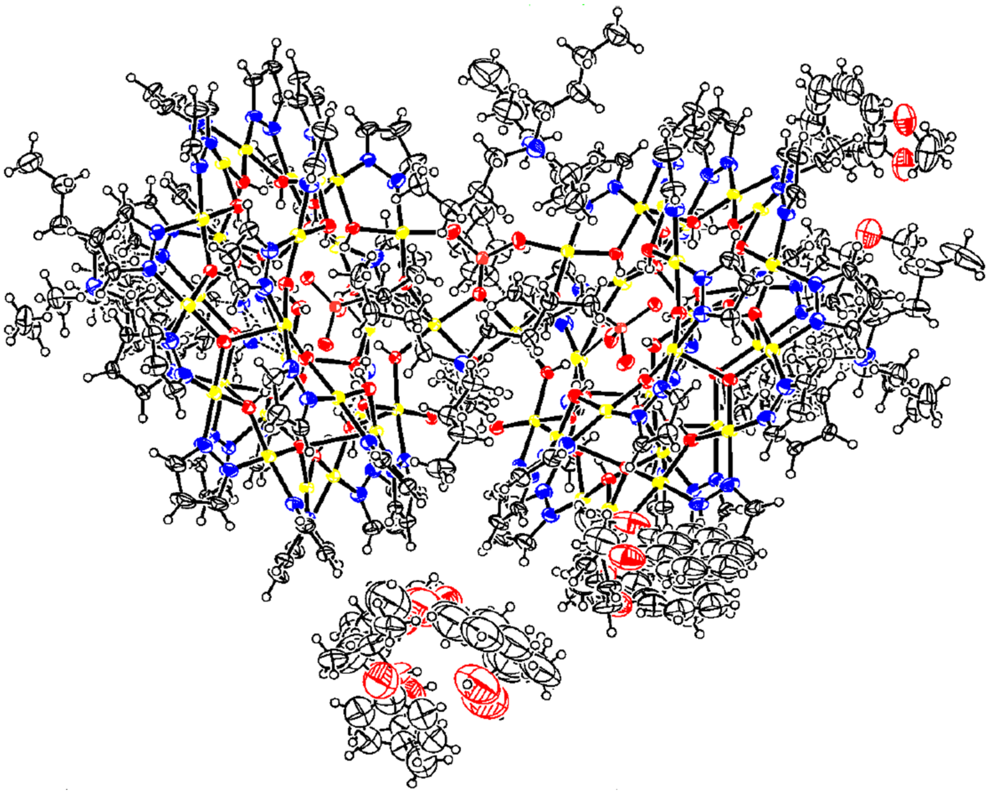

Figure S13. Thermal ellipsoid plot of the crystal structure of **12**.

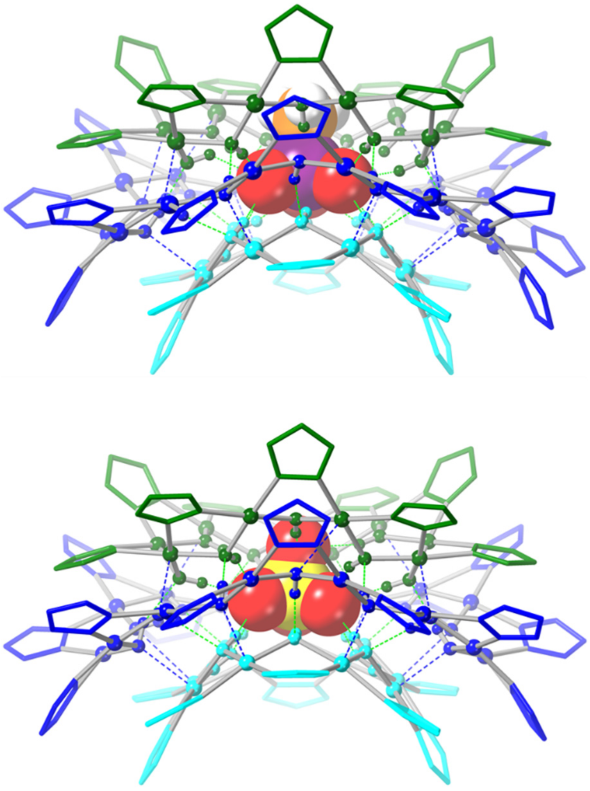

Figure S14. Comparison of the crystal structures of $Cu_{8+14+9}PO_3$ and $Cu_{8+14+9}SO_4$, illustrating the similar binding of the anions. Color code: light blue – Cu_8 ring; blue – Cu_{14} ring; green – Cu_9 ring.

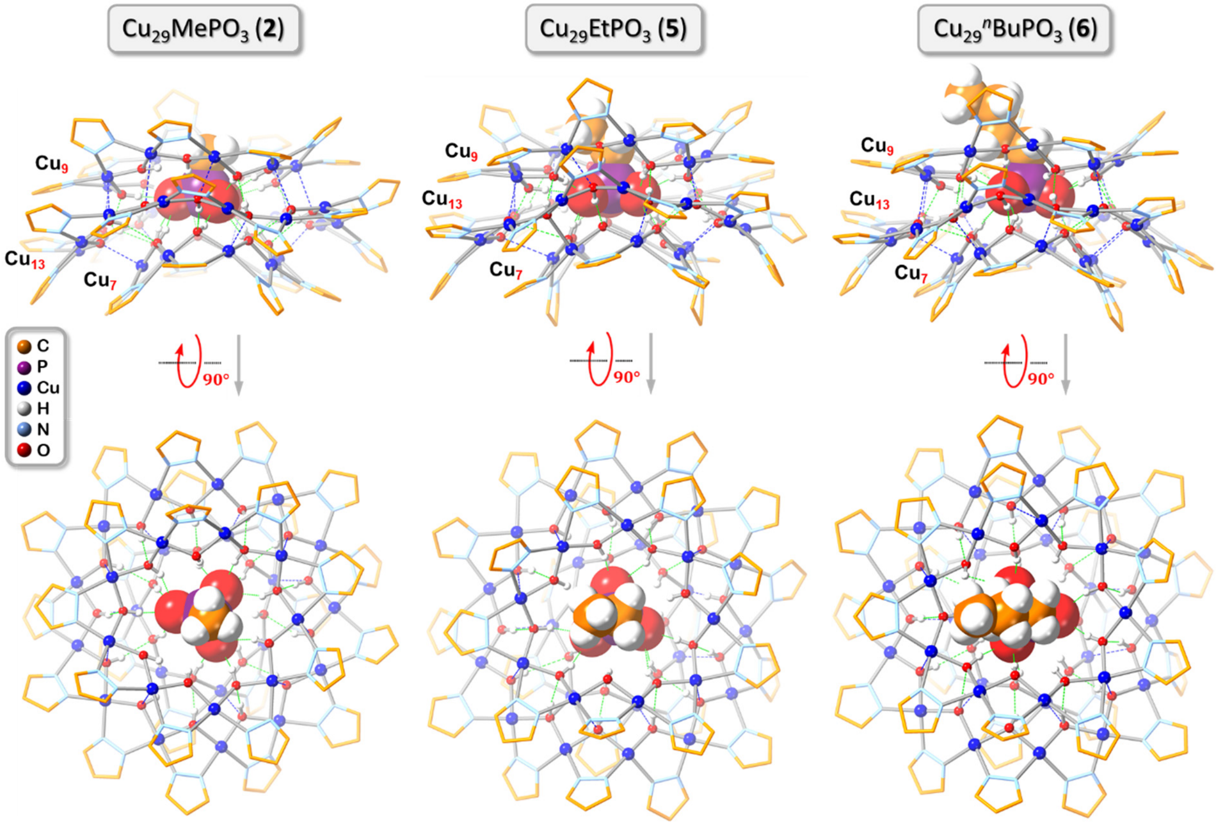

Figure S15. Ball-and-stick representation of the crystal structures of **2**, **5** and **6** (side- and top-views). Green and blue dotted lines indicate hydrogen bonds and axial Cu···O interactions, respectively. Counterions, lattice solvent molecules and C–H bond H-atoms are omitted for clarity, and only the major component is shown for disordered moieties.

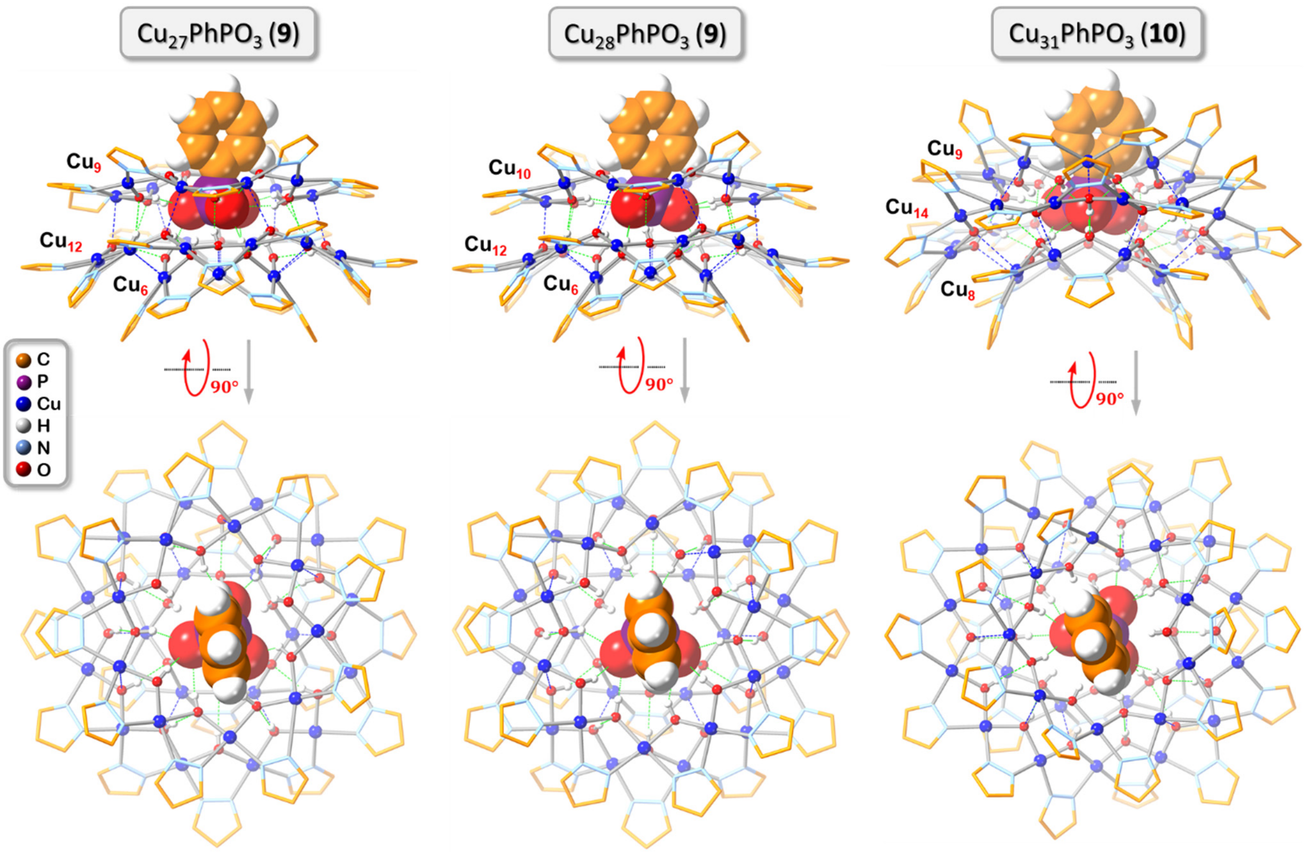

Figure S16. Ball-and-stick representation of the crystal structures of **9** and **10** (side- and top-views). Green and blue dotted lines indicate hydrogen bonds and axial Cu···O interactions, respectively. Counterions, lattice solvent molecules and C–H bond H-atoms are omitted for clarity, and only the major component is shown for disordered moieties. For **9**, only one of the two crystallographically independent nanojar moieties from the asymmetric unit is shown.

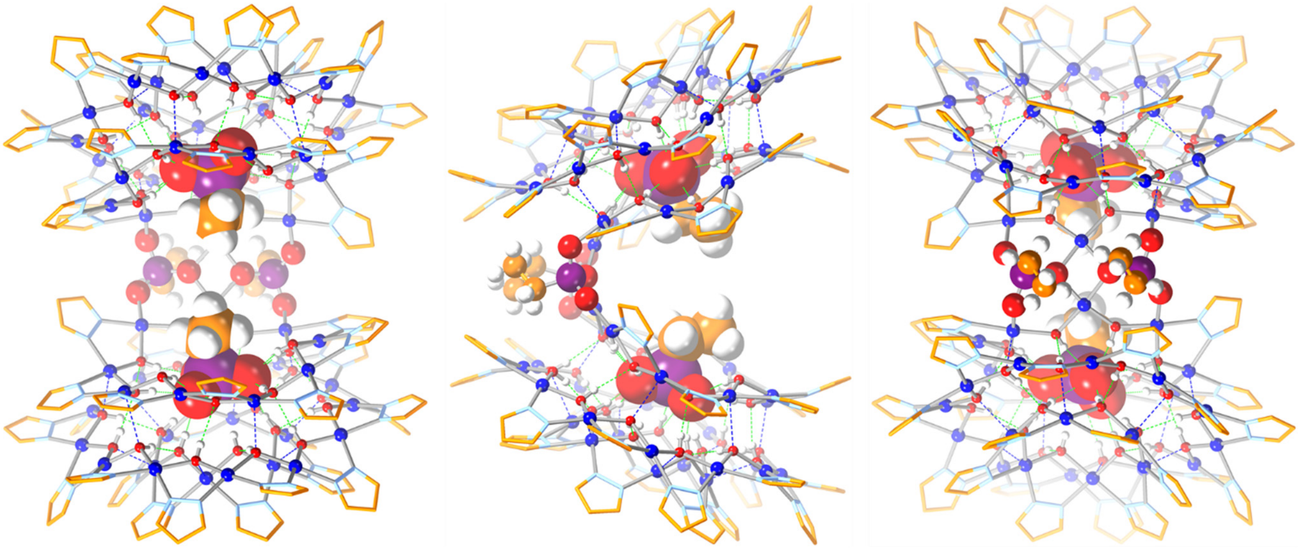

Figure S17. Different views of the crystal structure of **11**.

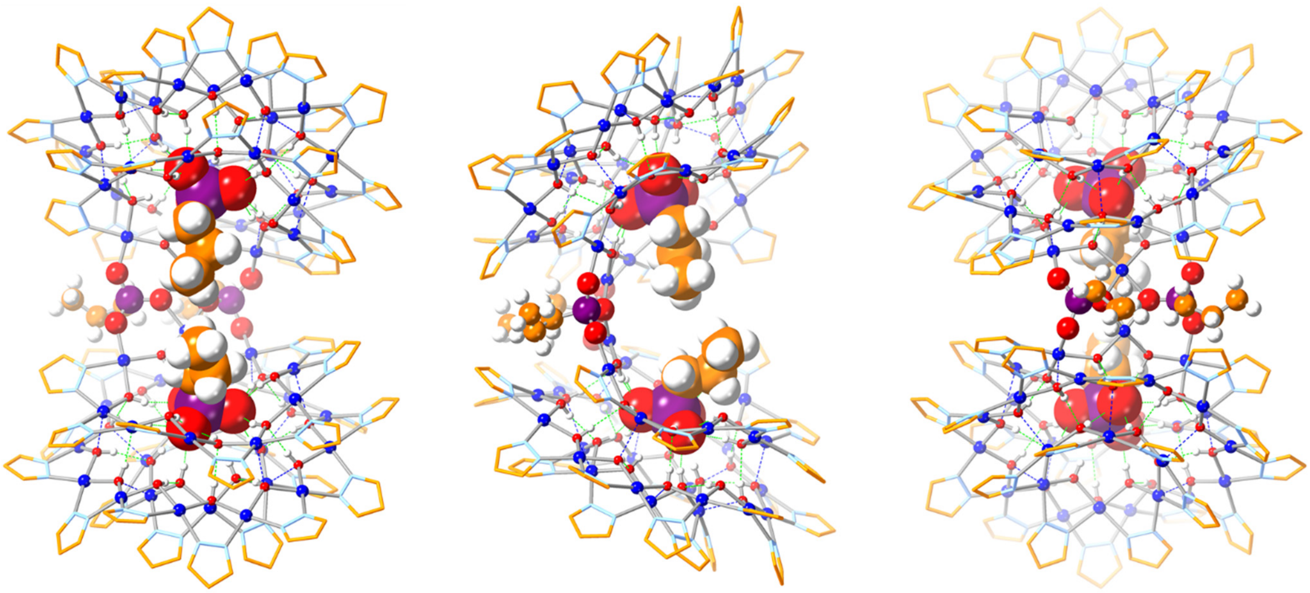

Figure S18. Different views of the crystal structure of **12**.

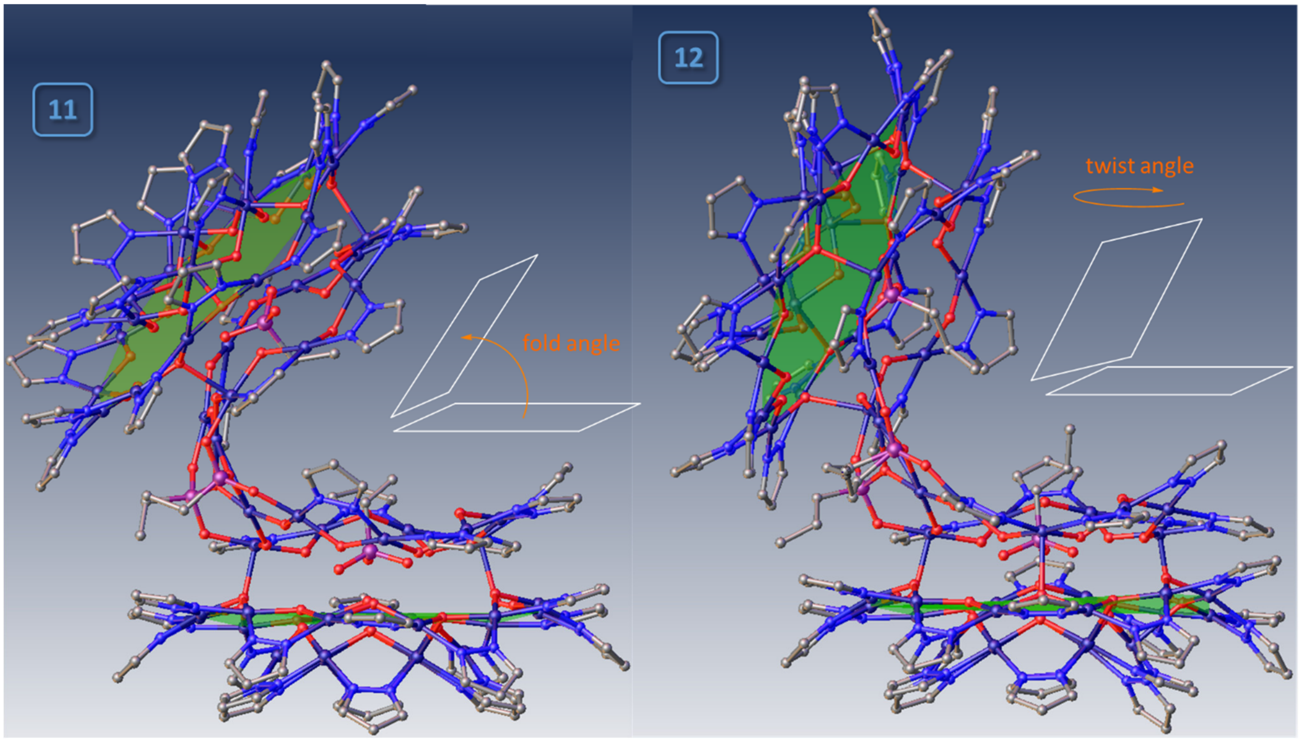

Figure S19. Comparison of the crystal structures of **11** and **12**, illustrating the larger fold and twist angles in the latter. Green planes indicate mean-planes through the Cu atoms of Cu_{12} rings. Counterions, lattice solvent molecules and H-atoms are omitted for clarity, and only the major component is shown for disordered moieties.

Table S1. Crystallographic data for the $MePO_3^{2-}$ nanojars (**1–3**).

	**1**	**2**	**3**
Formula (sum)	$C_{141}H_{215.98}Cl_{5.96}Cu_{27}N_{56}O_{30}P$	$C_{156.19}H_{224.91}Cl_{10.06}Cu_{29}N_{60}O_{32}P$	$C_{147.06}H_{223.06}Cu_{31}N_{64}O_{37}P$
Formula (moiety)	$C_{81}H_{108}Cu_{27}N_{54}O_{27}$, CH_3PO_3, 2($C_{16}H_{36}N$), 2.76($C_6H_4Cl_2$), 0.222(C_6H_{14})	$C_{87}H_{116}Cu_{29}N_{58}O_{29}$, CH_3PO_3, 2($C_{16}H_{36}N$), 4.413($C_6H_4Cl_2$), 0.506(C_6H_{14})	$C_{93}H_{124}Cu_{31}N_{62}O_{31}$, CH_3PO_3, 2($C_{16}H_{36}N$), 3.008(C_7H_8O), solvent
FW ($g\cdot mol^{-1}$)	5134.00	5685.77	5480.75
Crystal system	Triclinic	Monoclinic	Triclinic
Space group	$P\bar{1}$ (No. 2)	$P2_1/c$ (No. 14)	$P\bar{1}$ (No. 2)
a (Å)	18.845(3)	42.627(11)	20.280(4)
b (Å)	22.174(3)	40.868(6)	20.556(5)
c (Å)	25.843(3)	26.790(5)	28.980(8)
α (deg)	104.650(7)	90.000	92.280(19)
β (deg)	107.310(6)	106.984(9)	107.636(16)
γ (deg)	96.040(7)	90.000	97.742(11)
V (Å^3)	9783(2)	44634(16)	11367(5)
Z	2	8	2
D_{calc} ($g\cdot cm^{-3}$)	1.743	1.692	1.601
μ (mm^{-1})	4.506	4.653	3.680
θ range (deg)	2.10–82.56	2.04–80.64	2.18–81.08
Reflns collected	528247	356654	133634
R_{int}	0.0553	0.0983	0.0852
Obsd reflns [$I > 2\sigma(I)$]	36462	58260	30388
Data/restraints/parameters	42623/6079/3597	93312/6311/6170	45211/2217/3133
GOF (on F^2)	1.058	1.054	1.004
R factors [$I > 2\ \sigma(I)$]	R_1 = 0.0483<br>wR_2 = 0.1345	R_1 = 0.0707<br>wR_2 = 0.1904	R_1 = 0.0849<br>wR_2 = 0.2156
R factors (all data)	R_1 = 0.0553<br>wR_2 = 0.1414	R_1 = 0.1115<br>wR_2 = 0.2213	R_1 = 0.1197<br>wR_2 = 0.2421
Maximum peak/hole (e·Å^{-3})	1.668/−2.139	1.567/−1.103	1.927/−1.190
CCDC number	2359476	2359478	2359472

Table S2. Crystallographic data for the $EtPO_3^{2-}$ (**4**, **5**) and $^{n}BuPO_3^{2-}$ (**6**) nanojars.

	**4**	**5**	**6**
Formula (sum)	$C_{180.39}H_{252.38}Cu_{31}N_{64}O_{34}P$	$C_{154.03}H_{231.84}Cu_{30.89}N_{63.79}O_{37.81}P$	$C_{130}H_{213}Cu_{29}N_{60}O_{32}P$
Formula (moiety)	$C_{93}H_{124}Cu_{31}N_{62}O_{31}$, $C_2H_5PO_3$, 2($C_{16}H_{36}N$), 4.672($C_{11}H_{10}$), 0.331(C_6H_{14})	0.9465($C_{93}H_{124}Cu_{31}N_{62}O_{31}$), 0.0535($C_{87}H_{116}Cu_{29}N_{58}O_{29}$), $C_2H_5PO_3$, 2($C_{16}H_{36}N$), 3.9065(C_7H_8O), solvent	$C_{87}H_{116}Cu_{29}N_{58}O_{29}$, 2($C_{16}H_{36}N$), $C_4H_9PO_3$, C_7H_{16}
FW (g·mol^{-1})	5862.47	5576.07	5002.50
Crystal system	Triclinic	Triclinic	Monoclinic
Space group	$P\bar{1}$ (No. 2)	$P\bar{1}$ (No. 2)	$P2_1/c$ (No. 14)
a (Å)	20.919(4)	20.577(6)	20.9059(9)
b (Å)	23.597(5)	20.982(6)	35.2467(15)
c (Å)	27.100(6)	27.544(9)	28.6590(12)
α (deg)	64.896(10)	73.530(18)	90.000
β (deg)	71.398(9)	70.880(10)	93.404(2)
γ (deg)	89.117(10)	72.522(11)	90.000
V (Å^3)	11368(4)	10489(5)	21080.5(16)
Z	2	2	4
D_{calc} (g·cm^{-3})	1.713	1.766	1.576
μ (mm^{-1})	3.716	3.993	3.690
θ range (deg)	1.92–80.94	2.33–80.26	1.99–80.34
Reflns collected	138211	125041	291491
R_{int}	0.0580	0.0733	0.0759
Obsd reflns [$I > 2\sigma(I)$]	29599	31117	38047
Data/restraints/parameters	45129/4556/3624	42769/3881/3597	45614/999/2619
GOF (on F^2)	1.021	1.024	1.015
R factors [$I > 2\sigma(I)$]	R_1 = 0.0857<br>wR_2 = 0.2201	R_1 = 0.0615<br>wR_2 = 0.1568	R_1 = 0.0620<br>wR_2 = 0.1635
R factors (all data)	R_1 = 0.1226<br>wR_2 = 0.2551	R_1 = 0.0850<br>wR_2 = 0.1757	R_1 = 0.0695<br>wR_2 = 0.1683
Maximum peak/hole (e·Å^{-3})	1.119/−1.206	1.129/−1.209	1.457/−1.420
CCDC number	2359471	2359473	2359475

Table S3. Crystallographic data for the $BnPO_3^{2-}$ (**8**) and $PhPO_3^{2-}$ (**9**, **10**) nanojars.

	**8**	**9**	**10**
Formula (sum)	$C_{132}H_{203}Cu_{31}N_{64}O_{34}P$	$C_{323.76}H_{485.71}Cu_{55.73}N_{115.46}O_{77.03}P_2$	$C_{188.84}H_{273.30}Cu_{31}N_{64}O_{48.46}P$
Formula (moiety)	$C_{93}H_{124}Cu_{31}N_{62}O_{31}$, $C_7H_7PO_3$, 2($C_{16}H_{36}N$), solvent	1.732($C_{84}H_{112}Cu_{28}N_{56}O_{28}$), 0.268($C_{81}H_{108}Cu_{27}N_{54}O_{27}$), 2($C_6H_5PO_3$), 3.534($C_{16}H_{36}N$), 0.466($C_{19}H_{18}N$), 7.649($C_8H_{10}O_2$), 2.367($C_7H_{16}$)	$C_{93}H_{124}Cu_{31}N_{62}O_{31}$, 2($C_{16}H_{36}N$), $C_6H_5PO_3$, 7.23($C_8H_{10}O_2$)
FW (g·mol^{-1})	5231.59	10831.73	6216.45
Crystal system	Triclinic	Monoclinic	Triclinic
Space group	$P\bar{1}$ (No. 2)	$P2_1/n$ (No. 14)	$P\bar{1}$ (No. 2)
a (Å)	20.4825(12)	14.939(3)	20.4646(5)
b (Å)	20.5299(9)	34.946(5)	25.0830(8)
c (Å)	28.6796(14)	80.333(14)	25.4815(9)
α (deg)	92.956(2)	90.000	109.196(2)
β (deg)	108.837(2)	92.640(10)	96.5120(10)
γ (deg)	97.153(2)	90.000	91.6450(10)
V (Å^3)	11270.9(10)	41895(12)	12242.6(7)
Z	2	4	2
D_{calc} (g·cm^{-3})	1.541	1.717	1.686
μ (mm^{-1})	3.671	3.680	3.535
θ range (deg)	2.93–80.29	2.59–80.35	2.67–83.67
Reflns collected	139748	442276	298378
R_{int}	0.0609	0.0762	0.0468
Obsd reflns [$I > 2\sigma(I)$]	36529	71397	48638
Data/restraints/parameters	46053/4045/3175	88977/13623/7495	52414/4098/3970
GOF (on F^2)	1.018	1.005	1.078
R factors [$I > 2\ \sigma(I)$]	R_1 = 0.0726<br>wR_2 = 0.2085	R_1 = 0.0622<br>wR_2 = 0.1793	R_1 = 0.0437<br>wR_2 = 0.1181
R factors (all data)	R_1 = 0.0850<br>wR_2 = 0.2185	R_1 = 0.0737<br>wR_2 = 0.1889	R_1 = 0.0466<br>wR_2 = 0.1202
Maximum peak/hole (e·Å^{-3})	1.739/−0.872	1.124/−1.161	0.950/−1.051
CCDC number	2359477	2359482	2359483

Table S4. Crystallographic data for the $^{n}C_{12}PO_3^{2-}$ (**7**), $EtPO_3^{2-}$ (**11**) and $^{n}PrPO_3^{2-}$ (**12**) nanojars.

	**7**	**11**	**12**
Formula (sum)	$C_{312.59}H_{467.73}Cl_{12.86}Cu_{62}N_{128}O_{68}P_2$	$C_{342.44}H_{544.47}Cu_{54}N_{104}O_{77.64}P_4$	$C_{272.54}H_{439.60}Cu_{54}N_{104}O_{73.47}P_4$
Formula (moiety)	$C_{93}H_{124}Cu_{31}N_{62}O_{31}$, $C_{12}H_{25}O_3P$, 2($C_{16}H_{36}N$), 3.216($C_6H_4Cl_2$)	$C_{154}H_{214}Cu_{54}N_{100}O_{60}P_2$, 2($C_2H_5PO_3$), 4($C_{16}H_{36}N$), 7.308($C_{11}H_{10}$), 11.64($C_4H_{10}O$)	$C_{156}H_{218}Cu_{54}N_{100}O_{60}P_2$, 2($C_3H_7PO_3$), 4($C_{16}H_{36}N$), 5.554($C_7H_8O$), 2.1916($C_4H_{10}O$)
FW (g·mol^{-1})	11564.34	10916.45	9903.95
Crystal system	Triclinic	Monoclinic	Monoclinic
Space group	$P\bar{1}$ (No. 2)	C2/c (No. 15)	$P2_1/n$ (No. 14)
a (Å)	28.269(14)	61.082(3)	22.525(3)
b (Å)	29.264(14)	21.5785(11)	53.875(8)
c (Å)	32.900(8)	39.569(2)	30.867(4)
α (deg)	64.03(2)	90.000	90.000
β (deg)	80.59(2)	122.381(2)	96.704(12)
γ (deg)	72.10(2)	90.000	90.000
V (Å^3)	23271(18)	44044(4)	37202(8)
Z	2	4	4
D_{calc} (g·cm^{-3})	1.650	1.646	1.768
μ (mm^{-1})	2.916	3.484	4.043
θ range (deg)	1.86–28.34	2.34–80.72	2.14–80.55
Reflns collected	147222	306978	632717
R_{int}	0.0854	0.0388	0.0454
Obsd reflns [$I > 2\sigma(I)$]	54645	44582	77420
Data/restraints/parameters	109761/7309/6566	47662/5594/3758	80771/3380/5460
GOF (on F^2)	0.925	1.031	1.139
R factors [$I > 2\sigma(I)$]	R_1 = 0.0531<br>wR_2 = 0.1330	R_1 = 0.0373<br>wR_2 = 0.1047	R_1 = 0.0438<br>wR_2 = 0.1069
R factors (all data)	R_1 = 0.1369<br>wR_2 = 0.1644	R_1 = 0.0394<br>wR_2 = 0.1064	R_1 = 0.0459<br>wR_2 = 0.1079
Maximum peak/hole (e·Å^{-3})	1.302/−0.803	1.719/−0.763	2.420/−1.619
CCDC number	2359474	2359480	2359481

Table S5. Comparison of bond lengths (Å) and angles (°), Cu···O distances shorter than the sum of the van der Waals radii of Cu and O (2.92 Å) and H-bonding (with D···A distances shorter than 3.2 Å) in **1**–**3** (based on the major component in the case of disordered moieties).

	**$Cu_{27}MePO_3$ (1)**	**$Cu_{29}MePO_3$ (2)**	**$Cu_{29}MePO_3$ (2)**	**$Cu_{31}MePO_3$ (3)**
Cu–O within Cu_n rings	1.895(3)–1.950(2)<br>avg: 1.926(3)	1.884(5)–1.970(5)<br>avg: 1.922(5)	1.887(5)–1.965(4)<br>avg: 1.922(5)	1.846(6)–1.960(6)<br>avg: 1.921(6)
Cu–N within Cu_n rings	1.828(7)–2.169(11)<br>avg: 1.981(4)	1.948(6)–2.024(5)<br>avg: 1.979(6)	1.935(6)–2.038(5)<br>avg: 1.977(6)	1.939(8)–2.015(7)<br>avg: 1.973(8)
N–Cu–O (*trans*) within Cu_{12}, Cu_{13} or Cu_{14} ring	164.59(16)–174.71(13)<br>avg: 170.8(2)	163.1(2)–174.9(2)<br>avg: 169.4(2)	162.3(2)–174.2(2)<br>avg: 169.3(2)	158.7(5)–174.6(3)<br>avg: 169.5(3)
N–Cu–O (*trans*) within Cu_9 ring	150.83(13)–176.19(12)<br>avg: 165.9(2)	164.4(3)–175.5(2)<br>avg: 171.8(2)	162.8(3)–175.2(2)<br>avg: 171.4(2)	169.7(3)–178.0(3)<br>avg: 173.9(3)
N–Cu–O (*trans*) within Cu_6, Cu_7 or Cu_8 ring	168.82(14)–173.68(13)<br>avg: 171.4(2)	165.2(2)–179.14(19)<br>avg: 171.2(2)	163.3(2)–178.2(2)<br>avg: 171.0(2)	166.0(3)–176.7(2)<br>avg: 172.5(3)
Average of all N–Cu–O (trans) angles	*169.4(2)*	*170.8(2)*	*170.6(2)*	*172.0(3)*
N–Cu–O (*cis*) within Cu_{12}, Cu_{13} or Cu_{14} ring	86.26(12)–87.15(11)<br>avg: 86.6(1)	84.5(2)–88.2(2)<br>avg: 86.9(2)	84.8(2)–88.7(2)<br>avg: 87.1(2)	85.2(2)–89.7(3)<br>avg: 87.7(3)
N–Cu–O (*cis*) within Cu_9 ring	82.25(11)–88.59(12)<br>avg: 85.2(1)	82.80(19)–86.6(2)<br>avg: 84.6(2)	83.11(19)–86.6(2)<br>avg: 84.7(2)	83.1(3)–87.2(3)<br>avg: 84.9(3)
N–Cu–O (*cis*) within Cu_6, Cu_7 or Cu_8 ring	83.35(11)–84.39(11)<br>avg: 83.9(1)	82.45(19)–87.4(2)<br>avg: 84.6(2)	82.58(19)–88.7(2)<br>avg: 84.9(2)	82.9(3)–87.3(2)<br>avg: 85.2(3)
Average of all N–Cu–O (cis) angles	*85.2(1)*	*85.4(2)*	*85.6(2)*	*85.9(3)*
Cu···O between Cu_9 and Cu_{12} (or Cu_{13} or Cu_{14}) rings	2.373(2)–2.850(2)<br>(6 interactions)<br>avg: 2.627(2)	2.380(4)–2.860(5)<br>(7 interactions)<br>avg: 2.547(5)	2.389(4)–2.867(5)<br>(7 interactions)<br>avg: 2.560(5)	2.361(5)–2.833(7)<br>(6 interactions)<br>avg: 2.556(6)
Cu···O between Cu_6 (or Cu_7 or Cu_8) and Cu_{12} (or Cu_{13} or Cu_{14}) rings	2.352(3)–2.552(3)<br>(6 interactions)<br>avg: 2.449(2)	2.346(3)–2.520(4)<br>(5 interactions)<br>avg: 2.418(4)	2.364(4)–2.524(4)<br>(5 interactions)<br>avg: 2.438(4)	2.402(6)–2.745(5)<br>(7 interactions)<br>avg: 2.500(6)
Average of all Cu···O interactions between Cu_n rings	*2.538(2)*<br>*(12 interactions)*	*2.483(5)*<br>*(12 interactions)*	*2.499(6)*<br>*(12 interactions)*	*2.528(6)*<br>*(13 interactions)*
Cu···Cu distances in Cu_{12} (or Cu_{13} or Cu_{14} ring)	3.2071(10)–3.3857(10)<br>avg: 3.303(1)	3.1875(12)–3.3845(17)<br>avg: 3.300(1)	3.1819(12)–3.4086(12)<br>avg: 3.299(1)	3.164(2)–3.3774(19)<br>avg: 3.262(2)
Cu···Cu distances in Cu_9 ring	3.2126(10)–3.4170(10)<br>avg: 3.313(1)	3.2340(12)–3.3913(13)<br>avg: 3.344(1)	3.2404(12)–3.4043(12)<br>avg: 3.327(1)	3.179(2)–3.3846(18)<br>avg: 3.304(2)
Cu···Cu distances in Cu_6, Cu_7 or Cu_8 ring	3.2720(10)–3.3705(10)<br>avg: 3.323(1)	3.1563(12)–3.4436(12)<br>avg: 3.338(1)	3.1458(12)–3.4327(12)<br>avg: 3.328(1)	3.236(2)–3.3772(19)<br>avg: 3.321(2)
Average of all Cu···Cu distances in Cu_n rings	*3.313(1)*	*3.327(1)*	*3.318(1)*	*3.296(2)*
H-bonded O···O distances between Cu_9 and Cu_{12} (or Cu_{13} or Cu_{14}) rings	2.746(3)–2.901(3)<br>(6 interactions)<br>avg: 2.810(4)	2.703(6)–2.804(6)<br>(5 interactions)<br>avg: 2.756(7)	2.707(5)–2.793(6)<br>(5 interactions)<br>avg: 2.755(6)	2.768(8)–2.922(9)<br>(6 interactions)<br>avg: 2.835(9)
H-bonded O···O distances between Cu_6 (or Cu_7 or Cu_8) and Cu_{12} (or Cu_{13} or Cu_{14}) rings	2.756(4)–2.785(4)<br>(6 interactions)<br>avg: 2.770(4)	2.746(6)–2.940(6)<br>(7 interactions)<br>avg: 2.858(6)	2.764(6)–2.933(6)<br>(7 interactions)<br>avg: 2.858(6)	2.771(6)–2.913(9)<br>(6 interactions)<br>avg: 2.842(9)
Average of all H-bonded O···O distances between Cu_n rings	*2.790(4)*<br>*(12 interactions)*	*2.807(7)*<br>*(12 interactions)*	*2.807(6)*<br>*(12 interactions)*	*2.839(9)*<br>*(12 interactions)*
H-bonded O···O distances between Cu_n rings and $MePO_3^{2-}$	2.713(3)–3.184(4)<br>(14 interactions)<br>avg: 2.874(4)	2.724(7)–3.172(8)<br>(13 interactions)<br>avg: 2.873(7)	2.693(7)–3.160(7)<br>(12 interactions)<br>avg: 2.852(7)	2.814(8)–3.116(9)<br>(13 interactions)<br>avg: 2.954(8)

Table S6. Comparison of bond lengths (Å) and angles (°), Cu⋯O distances shorter than the sum of the van der Waals radii of Cu and O (2.92 Å) and H-bonding (with D⋯A distances shorter than 3.2 Å) in **4**–**6** (based on the major component in the case of disordered moieties).

	**$Cu_{31}EtPO_3$ (4)**	**$Cu_{31}EtPO_3$ (5)**	**$Cu_{29}EtPO_3$ (5)**	**$Cu_{29}{}^nBuPO_3$ (6)**
Cu–O within Cu_n rings	1.887(6)–1.967(6) avg: 1.920(6)	1.884(4)–1.960(4) avg: 1.921(4)	1.875(18)–2.140(19) avg: 1.933(4)	1.875(3)–1.954(3) avg: 1.923(3)
Cu–N within Cu_n rings	1.932(8)–2.010(7) avg: 1.973(8)	1.942(5)–2.017(5) avg: 1.975(5)	1.952(5)–2.458(18) avg: 1.990(5)	1.890(7)–2.032(5) avg: 1.975(5)
N–Cu–O (*trans*) within Cu_{12}, Cu_{13} or Cu_{14} ring	160.1(3)–174.2(3) avg: 169.1(3)	161.4(2)–173.94(19) avg: 169.1(2)	150(3)–179.4(12) avg: 168.1(2)	162.83(18)–175.3(4) avg: 170.0(2)
N–Cu–O (*trans*) within Cu_9 ring	170.2(3)–178.6(3) avg: 173.7(3)	168.23(18)–177.32(18) avg: 173.5(2)	168.23(18)–177.32(18) avg: 173.5(2)	158.56(17)–178.48(17) avg: 172.6(2)
N–Cu–O (*trans*) within Cu_6, Cu_7 or Cu_8 ring	165.4(3)–176.9(3) avg: 171.7(3)	164.72(17)–176.64(18) avg: 171.4(2)	150.1(15)–176.32(17) avg: 164.4(2)	165.28(18)–176.87(18) avg: 171.8(2)
Average of all N–Cu–O (trans) angles	*171.5(3)*	*171.3(2)*	*168.6(2)*	*171.5(2)*
N–Cu–O (*cis*) within Cu_{12}, Cu_{13} or Cu_{14} ring	85.6(3)–89.3(3) avg: 87.7(3)	85.37(19)–89.27(19) avg: 87.7(2)	70.0(5)–90.0(11) avg: 86.3(2)	83.90(17)–91.0(4) avg: 87.0(2)
N–Cu–O (*cis*) within Cu_9 ring	83.0(3)–86.9(3) avg: 85.1(3)	82.34(17)–86.91(19) avg: 84.7(2)	82.34(17)–86.91(19) avg: 84.7(2)	82.52(18)–87.40(18) avg: 84.8(2)
N–Cu–O (*cis*) within Cu_6, Cu_7 or Cu_8 ring	83.6(3)–88.2(3) avg: 85.2(3)	83.06(18)–88.0(2) avg: 85.4(2)	76.0(10)–86.2(14) avg: 83.8(2)	82.05(15)–87.18(16) avg: 84.7(2)
Average of all N–Cu–O (cis) angles	*86.0(3)*	*85.9(2)*	*84.9(2)*	*85.5(2)*
Cu⋯O between Cu_9 and Cu_{12} (or Cu_{13} or Cu_{14}) rings	2.392(7)–2.899(9) (7 interactions) avg: 2.610(8)	2.365(4)–2.757(4) (7 interactions) avg: 2.567(5)	2.365(4)–2.77(4) (7 interactions) avg: 2.58(4)	2.373(4)–2.787(3) (5 interactions) avg: 2.525(4)
Cu⋯O between Cu_6 (or Cu_7 or Cu_8) and Cu_{12} (or Cu_{13} or Cu_{14}) rings	2.398(6)–2.527(5) (6 interactions) avg: 2.462(6)	2.412(6)–2.849(6) (7 interactions) avg: 2.513(5)	2.41(8)–2.832(17) (6 interactions) avg: 2.51(5)	2.368(3)–2.706(4) (7 interactions) avg: 2.542(4)
Average of all Cu⋯O interactions between Cu_n rings	*2.536(8) (13 interactions)*	*2.540(5) (14 interactions)*	*2.54(5) (13 interactions)*	*2.533(4) (12 interactions)*
Cu⋯Cu distances in Cu_{12} (or Cu_{13} or Cu_{14} ring)	3.159(2)–3.3857(19) avg: 3.264(2)	3.1471(16)–3.3703(15) avg: 3.262(2)	3.1471(16)–3.70(2) avg: 3.339(2)	3.1827(10)–3.4130(11) avg: 3.290(1)
Cu⋯Cu distances in Cu_9 ring	3.177(2)–3.409(2) avg: 3.326(2)	3.1726(16)–3.4381(16) avg: 3.332(2)	3.1726(16)–3.4381(16) avg: 3.332(2)	3.1977(11)–3.4551(10) avg: 3.330(1)
Cu⋯Cu distances in Cu_6, Cu_7 or Cu_8 ring	3.2312(18)–3.368(2) avg: 3.321(2)	3.2258(13)–3.3557(16) avg: 3.315(2)	3.25(2)–3.69(2) avg: 3.38(2)	3.2150(10)–3.4498(9) avg: 3.320(1)
Average of all Cu⋯Cu distances in Cu_n rings	*3.304(2)*	*3.303(2)*	*3.349(2)*	*3.313(1)*
H-bonded O⋯O distances between Cu_9 and Cu_{12} (or Cu_{13} or Cu_{14}) rings	2.763(7)–3.011(9) (6 interactions) avg: 2.869(9)	2.787(5)–2.982(10) (6 interactions) avg: 2.862(6)	2.48(3)–2.813(5) (5 interactions) avg: 2.739(6)	2.791(5)–2.949(6) (7 interactions) avg: 2.864(5)
H-bonded O⋯O distances between Cu_6 (or Cu_7 or Cu_8) and Cu_{12} (or Cu_{13} or Cu_{14}) rings	2.792(11)–2.904(11) (6 interactions) avg: 2.832(9)	2.771(7)–3.158(6) (7 interactions) avg: 2.874(6)	2.67(4)–2.839(6) (4 interactions) avg: 2.760(7)	2.710(4)–2.930(5) (6 interactions) avg: 2.816(5)
Average of all H-bonded O⋯O distances between Cu_n rings	*2.851(9) (12 interactions)*	*2.868(6) (13 interactions)*	*2.750(7) (9 interactions)*	*2.839(5) (13 interactions)*
H-bonded O⋯O distances between Cu_n rings and $EtPO_3^{2-}$	2.768(13)–3.151(17) (12 interactions) avg: 2.965(13)	2.808(11)–3.034(11) (10 interactions) avg: 2.891(11)	2.58(4)–3.05(4) (12 interactions) avg: 2.81(4)	2.743(5)–2.943(6) (11 interactions) avg: 2.836(5)

Table S7. Comparison of bond lengths (Å) and angles (°), Cu⋯O distances shorter than the sum of the van der Waals radii of Cu and O (2.92 Å) and H-bonding (with D⋯A distances shorter than 3.2 Å) in **9** (based on the major component in the case of disordered moieties).

	**$Cu_{28}PhPO_3$ (9)**	**$Cu_{28}PhPO_3$ (9)**	**$Cu_{27}PhPO_3$ (9)**	**$Cu_{27}PhPO_3$ (9)**
Cu–O within Cu_n rings	1.868(5)–1.957(3) avg: 1.927(5)	1.891(4)–1.949(3) avg: 1.928(4)	1.881(16)–2.157(14) avg: 1.936(4)	1.84(2)–1.974(19) avg: 1.927(4)
Cu–N within Cu_n rings	1.945(5)–2.047(7) avg: 1.975(5)	1.926(5)–2.013(6) avg: 1.975(5)	1.945(5)–2.77(3) avg: 1.994(5)	1.953(4)–2.305(15) avg: 1.989(5)
N–Cu–O (*trans*) within Cu_{12} ring	166.2(2)–174.4(3) avg: 172.0(2)	165.86(18)–175.1(2) avg: 172.0(2)	166.2(2)–174.4(3) avg: 172.0(2)	165.86(18)–175.1(2) avg: 172.0(2)
N–Cu–O (*trans*) within Cu_{10} (or Cu_9) ring	163.4(2)–178.7(3) avg: 173.4(3)	163.24(17)–178.0(2) avg: 173.6(3)	160.1(10)–176.9(17) avg: 168.7(17)	159.1(18)–177.4(9) avg: 170.2(9)
N–Cu–O (*trans*) within Cu_6 ring	164.62(17)–176.74(14) avg: 170.9(2)	165.35(17)–176.84(15) avg: 170.9(2)	164.62(17)–176.74(14) avg: 170.9(2)	165.35(17)–176.84(15) avg: 170.9(2)
Average of all N–Cu–O (trans) angles	*172.1(2)*	*172.2(2)*	*170.5(2)*	*171.0(2)*
N–Cu–O (*cis*) within Cu_{12} ring	85.40(18)–87.58(16) avg: 86.4(2)	85.10(17)–87.37(15) avg: 86.4(2)	85.40(18)–87.58(16) avg: 86.4(2)	85.10(17)–87.37(15) avg: 86.4(2)
N–Cu–O (*cis*) within Cu_{10} (or Cu_9) ring	83.5(3)–87.8(3) avg: 85.8(3)	84.01(18)–86.72(17) avg: 85.7(2)	74.2(8)–89.6(8) avg: 84.3(8)	75.1(5)–87.7(9) avg: 84.6(9)
N–Cu–O (*cis*) within Cu_6 ring	84.83(15)–85.55(17) avg: 85.2(2)	85.11(14)–85.65(14) avg: 85.4(2)	84.83(15)–85.55(17) avg: 85.2(2)	85.11(14)–85.65(14) avg: 85.4(2)
Average of all N–Cu–O (cis) angles	*85.8(2)*	*85.8(2)*	*85.3(2)*	*85.5(2)*
Cu⋯O between Cu_{10} (or Cu_9) and Cu_{12} rings	2.318(5)–2.873(4) (6 interactions) avg: 2.607(5)	2.342(5)–2.846(5) (6 interactions) avg: 2.617(5)	2.399(7)–2.706(5) (6 interactions) avg: 2.579(7)	2.308(11)–2.796(5) (6 interactions) avg: 2.604(9)
Cu⋯O between Cu_6 and Cu_{12} rings	2.340(3)–2.478(4) (6 interactions) avg: 2.403(4)	2.329(3)–2.460(4) (6 interactions) avg: 2.396(4)	2.340(3)–2.478(4) (6 interactions) avg: 2.403(4)	2.329(3)–2.460(4) (6 interactions) avg: 2.396(4)
Average of all Cu⋯O interactions between Cu_n rings	*2.505(5) (12 interactions)*	*2.506(5) (12 interactions)*	*2.491(5) (12 interactions)*	*2.500(5) (12 interactions)*
Cu⋯Cu distances in Cu_{12} ring	3.210(2)–3.3832(11) avg: 3.310(2)	3.2222(16)–3.373(2) avg: 3.311(2)	3.210(2)–3.3832(11) avg: 3.310(2)	3.2222(16)–3.373(2) avg: 3.311(2)
Cu⋯Cu distances in Cu_{10} (or Cu_9) ring	3.090(2)–3.367(3) avg: 3.230(2)	3.1126(17)–3.348(2) avg: 3.238(2)	3.149(2)–3.576(8) avg: 3.385(8)	3.248(17)–3.437(12) avg: 3.333(12)
Cu⋯Cu distances in Cu_6 ring	3.246(2)–3.3639(16) avg: 3.290(2)	3.2424(16)–3.379(2) avg: 3.291(2)	3.246(2)–3.3639(16) avg: 3.290(2)	3.2424(16)–3.379(2) avg: 3.291(2)
Average of all Cu⋯Cu distances in Cu_n rings	*3.277(2)*	*3.280(2)*	*3.328(2)*	*3.312(2)*
H-bonded O⋯O distances between Cu_{10} (or Cu_9) and Cu_{12} rings	2.674(7)–2.866(11) (6 interactions) avg: 2.745(9)	2.674(5)–2.852(6) (6 interactions) avg: 2.761(5)	2.685(5)–3.06(5) (5 interactions) avg: 2.887(5)	2.698(4)–3.12(5) (5 interactions) avg: 2.878(5)
H-bonded O⋯O distances between Cu_6 and Cu_{12} rings	2.712(4)–2.762(4) (6 interactions) avg: 2.742(5)	2.728(5)–2.763(4) (6 interactions) avg: 2.745(4)	2.712(4)–2.762(4) (6 interactions) avg: 2.742(5)	2.728(5)–2.763(4) (6 interactions) avg: 2.745(4)
Average of all H-bonded O⋯O distances between Cu_n rings	*2.744(5) (12 interactions)*	*2.753(5) (12 interactions)*	*2.826(5) (11 interactions)*	*2.812(5) (11 interactions)*
H-bonded O⋯O distances between Cu_n rings and $PhPO_3^{2-}$	2.782(6)–3.20(2) (12 interactions) avg: 2.945(7)	2.784(5)–3.044(6) (12 interactions) avg: 2.919(6)	2.53(2)–3.20(2) (12 interactions) avg: 2.82(2)	2.537(17)–2.946(6) (12 interactions) avg: 2.755(6)

Table S8. Comparison of bond lengths (Å) and angles (°), Cu···O distances shorter than the sum of the van der Waals radii of Cu and O (2.92 Å) and H-bonding (with D···A distances shorter than 3.2 Å) in **7**, **8** and **10** (based on the major component in the case of disordered moieties).

	**$Cu_{31}{}^{n}C_{12}PO_3$ (7)**	**$Cu_{31}{}^{n}C_{12}PO_3$ (7)**	**$Cu_{31}BnPO_3$ (8)**	**$Cu_{31}PhPO_3$ (10)**
Cu–O within Cu_n rings	1.890(4)–1.954(4)<br>avg: 1.919(4)	1.888(4)–1.9504(4)<br>avg: 1.918(4)	1.873(4)–1.996(8)<br>avg: 1.923(5)	1.896(2)–1.957(2)<br>avg: 1.921(2)
Cu–N within Cu_n rings	1.945(6)–2.008(5)<br>avg: 1.974(5)	1.934(5)–2.014(5)<br>avg: 1.973(5)	1.843(12)–2.031(10)<br>avg: 1.970(6)	1.947(3)–2.078(12)<br>avg: 1.977(3)
N–Cu–O (*trans*) within Cu_{14} (or Cu_{12}) ring	159.74(19)–174.57(16)<br>avg: 169.2(2)	160.2(6)–174.24(19)<br>avg: 169.0(2)	161.2(6)–175.0(3)<br>avg: 169.4(3)	160.37(13)–174.61(11)<br>avg: 169.1(2)
N–Cu–O (*trans*) within Cu_9 ring	168.54(18)–178.64(18)<br>avg: 173.2(2)	166.90(19)–178.47(19)<br>avg: 173.3(2)	162.6(8)–176.6(2)<br>avg: 172.1(5)	165.47(11)–178.63(11)<br>avg: 171.7(2)
N–Cu–O (*trans*) within Cu_8 (or Cu_6) ring	159.62(18)–176.63(15)<br>avg: 171.7(2)	165.20(17)–178.44(18)<br>avg: 171.7(2)	165.2(2)–176.9(2)<br>avg: 172.4(2)	160.8(4)–177.5(5)<br>avg: 171.6(2)
Average of all N–Cu–O (trans) angles	*171.4(2)*	*171.3(2)*	*171.3(3)*	*170.8(2)*
N–Cu–O (*cis*) within Cu_{14} (or Cu_{12}) ring	84.75(18)–89.47(18)<br>avg: 87.4(2)	85.13(18)–89.11(19)<br>avg: 87.4(2)	84.4(4)–89.7(2)<br>avg: 87.6(2)	85.20(12)–89.45(11)<br>avg: 87.4(1)
N–Cu–O (*cis*) within Cu_9 ring	82.8(7)–87.06(17)<br>avg: 85.0(2)	82.98(19)–87.47(17)<br>avg: 85.0(2)	82.7(2)–87.5(4)<br>avg: 84.7(4)	83.41(10)–88.18(11)<br>avg: 85.2(1)
N–Cu–O (*cis*) within Cu_8 (or Cu_6) ring	83.65(16)–87.49(19)<br>avg: 85.4(2)	83.40(16)–87.4(2)<br>avg: 85.3(2)	83.3(2)–87.4(2)<br>avg: 85.3(2)	82.8(4)–87.67(10)<br>avg: 85.4(1)
Average of all N–Cu–O (cis) angles	*85.9(2)*	*85.9(2)*	*85.9(2)*	*86.0(1)*
Cu···O between Cu_9 and Cu_{14} (or Cu_{12}) rings	2.349(4)–2.887(5)<br>(7 interactions)<br>avg: 2.600(5)	2.384(4)–2.873(6)<br>(7 interactions)<br>avg: 2.637(5)	2.273(6)–2.690(8)<br>(6 interactions)<br>avg: 2.503(6)	2.342(2)–2.665(2)<br>(5 interactions)<br>avg: 2.511(2)
Cu···O between Cu_8 (or Cu_6) and Cu_{14} (or Cu_{12}) rings	2.443(5)–2.908(5)<br>(7 interactions)<br>avg: 2.549(5)	2.430(4)–2.912(5)<br>(7 interactions)<br>avg: 2.533(5)	2.398(5)–2.739(5)<br>(7 interactions)<br>avg: 2.499(5)	2.438(2)–2.814(3)<br>(8 interactions)<br>avg: 2.562(3)
Average of all Cu···O interactions between Cu_n rings	*2.575(5)<br>(14 interactions)*	*2.585(5)<br>(14 interactions)*	*2.501(6)<br>(13 interactions)*	*2.537(3)<br>(13 interactions)*
Cu···Cu distances in Cu_{14} (or Cu_{12}) ring	3.1566(12)–3.3824(19)<br>avg: 3.270(2)	3.1612(13)–3.3759(19)<br>avg: 3.272(2)	3.1850(17)–3.3722(14)<br>avg: 3.264(2)	3.1675(8)–3.3880(6)<br>avg: 3.2716(8)
Cu···Cu distances in Cu_9 ring	3.1738(12)–3.3945(14)<br>avg: 3.321(2)	3.1845(13)–3.4052(15)<br>avg: 3.321(2)	3.199(6)–3.428(8)<br>avg: 3.335(8)	3.1737(8)–3.3944(7)<br>avg: 3.3148(8)
Cu···Cu distances in Cu_8 (or Cu_6) ring	3.2163(17)–3.3804(14)<br>avg: 3.309(2)	3.2239(14)–3.3751(14)<br>avg: 3.316(2)	3.2284(13)–3.3595(13)<br>avg: 3.313(2)	3.2521(8)–3.3611(7)<br>avg: 3.3160(8)
Average of all Cu···Cu distances in Cu_n rings	*3.300(2)*	*3.303(2)*	*3.304(2)*	*3.3008(8)*
H-bonded O···O distances between Cu_9 and Cu_{14} (or Cu_{12}) rings	2.762(6)–3.045(7)<br>(6 interactions)<br>avg: 2.865(6)	2.761(6)–2.996(7)<br>(6 interactions)<br>avg: 2.860(6)	2.660(11)–2.947(5)<br>(6 interactions)<br>avg: 2.809(11)	2.753(3)–3.037(3)<br>(6 interactions)<br>avg: 2.853(3)
H-bonded O···O distances between Cu_8 (or Cu_6) and Cu_{14} (or Cu_{12}) rings	2.776(5)–2.931(5)<br>(6 interactions)<br>avg: 2.841(5)	2.782(6)–2.951(5)<br>(6 interactions)<br>avg: 2.856(6)	2.768(5)–2.901(6)<br>(6 interactions)<br>avg: 2.832(6)	2.775(4)–2.905(3)<br>(6 interactions)<br>avg: 2.826(4)
Average of all H-bonded O···O distances between Cu_n rings	*2.853(6)<br>(12 interactions)*	*2.858(6)<br>(12 interactions)*	*2.821(6)<br>(12 interactions)*	*2.840(4)<br>(12 interactions)*
H-bonded O···O distances between Cu_n rings and RPO_3^{2-} (R = ^{n}Bu, Ph, Bn)	2.772(5)–3.126(6)<br>(10 interactions)<br>avg: 2.923(6)	2.789(5)–3.182(8)<br>(12 interactions)<br>avg: 2.963(6)	2.693(11)–3.170(6)<br>(12 interactions)<br>avg: 2.957(6)	2.791(3)–3.159(3)<br>(12 interactions)<br>avg: 2.957(3)

Table S9. Comparison of bond lengths (Å) and angles (°), Cu···O distances shorter than the sum of the van der Waals radii of Cu and O (2.92 Å) and H-bonding (with D···A distances shorter than 3.2 Å) in **11** and **12** (based on the major component in the case of disordered moieties).

	**$(Cu_{27}EtPO_3)_2$ (11)**	**$(Cu_{27}{}^nPrPO_3)_2$ (12)**	**$(Cu_{27}{}^nPrPO_3)_2$ (12)**
Cu–O within Cu_n rings	1.8744(18)–1.9754(18)<br>avg: 1.928(2)	1.875(2)–1.982(2)<br>avg: 1.927(2)	1.879(2)–1.977(2)<br>avg: 1.926(2)
Cu–N within Cu_n rings	1.951(2)–2.029(2)<br>avg: 1.978(2)	1.957(3)–2.037(3)<br>avg: 1.983(3)	1.949(3)–2.050(3)<br>avg: 1.980(3)
N–Cu–O (*trans*) within Cu_{14} (or Cu_{12}) ring	165.88(10)–174.98(10)<br>avg: 170.9(1)	165.22(11)–175.36(14)<br>avg: 171.4(2)	165.14(12)–175.01(12)<br>avg: 171.3(2)
N–Cu–O (*trans*) within Cu_9 ring	153.98(9)–177.06(9)<br>avg: 167.4(1)	167.70(11)–177.98(14)<br>avg: 172.8(2)	168.39(11)–177.15(11)<br>avg: 173.2(2)
N–Cu–O (*trans*) within Cu_8 (or Cu_6) ring	169.17(9)–172.85(9)<br>avg: 171.2(1)	165.96(11)–174.96(11)<br>avg: 171.2(2)	165.93(11)–175.18(11)<br>avg: 171.1(2)
Average of all N–Cu–O (trans) angles	*169.8(1)*	*171.8(2)*	*171.8(2)*
N–Cu–O (*cis*) within Cu_{14} (or Cu_{12}) ring	85.46(9)–87.81(9)<br>avg: 86.7(1)	84.50(12)–87.73(10)<br>avg: 86.7(2)	85.41(11)–87.50(11)<br>avg: 86.6(2)
N–Cu–O (*cis*) within Cu_9 ring	83.24(8)–88.72(8)<br>avg: 85.8(1)	82.81(11)–86.00(11)<br>avg: 84.3(2)	82.82(10)–86.40(11)<br>avg: 84.3(2)
N–Cu–O (*cis*) within Cu_8 (or Cu_6) ring	83.78(8)–84.58(8)<br>avg: 84.2(1)	83.63(10)–85.00(10)<br>avg: 84.1(2)	83.60(10)–85.13(10)<br>avg: 84.3(2)
Average of all N–Cu–O (cis) angles	*85.6(1)*	*85.0(2)*	*85.1(2)*
Cu···O between Cu_9 and Cu_{14} (or Cu_{12}) rings	2.3353(18)–2.637(2)<br>(4 interactions)<br>avg: 2.471(2)	2.352(2)–2.814(3)<br>(4 interactions)<br>avg: 2.476(3)	2.329(2)–2.785(3)<br>(4 interactions)<br>avg: 2.471(3)
Cu···O between Cu_8 (or Cu_6) and Cu_{14} (or Cu_{12}) rings	2.351(3)–2.548(3)<br>(6 interactions)<br>avg: 2.452(3)	2.364(3)–2.593(3)<br>(6 interactions)<br>avg: 2.440(3)	2.350(3)–2.561(2)<br>(6 interactions)<br>avg: 2.424(3)
Average of all Cu···O interactions between Cu_n rings	*2.461(3)*<br>*(10 interactions)*	*2.458(3)*<br>*(10 interactions)*	*2.447(3)*<br>*(10 interactions)*
Cu···Cu distances in Cu_{14} (or Cu_{12}) ring	3.2206(7)–3.4117(16)<br>avg: 3.311(2)	3.1791(10)–3.3973(9)<br>avg: 3.309(1)	3.2074(11)–3.3799(10)<br>avg: 3.304(1)
Cu···Cu distances in Cu_9 ring	3.1402(7)–3.5553(13)<br>avg: 3.356(2)	3.1587(10)–3.5586(10)<br>avg: 3.353(1)	3.1419(11)–3.5190(11)<br>avg: 3.355(1)
Cu···Cu distances in Cu_8 (or Cu_6) ring	3.2428(13)–3.3769(7)<br>avg: 3.317(2)	3.2405(10)–3.3945(11)<br>avg: 3.315(1)	3.2419(11)–3.3855(10)<br>avg: 3.312(1)
Average of all Cu···Cu distances in Cu_n rings	*3.328(2)*	*3.326(1)*	*3.324(1)*
H-bonded O···O distances between Cu_9 and Cu_{14} (or Cu_{12}) rings	2.751(3)–2.948(2)<br>(6 interactions)<br>avg: 2.838(3)	2.753(3)–2.945(3)<br>(6 interactions)<br>avg: 2.815(3)	2.734(3)–2.905(3)<br>(6 interactions)<br>avg: 2.809(3)
H-bonded O···O distances between Cu_8 (or Cu_6) and Cu_{14} (or Cu_{12}) rings	2.734(3)–2.823(4)<br>(6 interactions)<br>avg: 2.771(3)	2.735(3)–2.756(3)<br>(6 interactions)<br>avg: 2.749(3)	2.720(3)–2.760(3)<br>(6 interactions)<br>avg: 2.742(3)
Average of all H-bonded O···O distances between Cu_n rings	*2.804(3)*<br>*(12 interactions)*	*2.779(3)*<br>*(12 interactions)*	*2.776(3)*<br>*(12 interactions)*
H-bonded O···O distances between Cu_n rings and RPO_3^{2-} (R = ^{n}Bu, Ph, Bn)	2.662(2)–3.194(3)<br>(13 interactions)<br>avg: 2.884(3)	2.691(3)–3.146(3)<br>(13 interactions)<br>avg: 2.855(3)	2.717(3)–3.075(4)<br>(12 interactions)<br>avg: 2.842(3)

Table S10. Comparison of the dihedral, twist and fold angles (°) between pyrazolate moieties and adjacent Cu–O–Cu units in **1–6** (based on the major component in the case of disordered moieties).

	DIHEDRAL ANGLE	TWIST ANGLE	FOLD ANGLE
**$Cu_{27}MePO_3$ (1)** Cu_{12}-ring	29.0(2)–58.2(4) avg: 42.9(2)	0.15(14)–7.6(2) avg: 3.4(2)	28.9(2)–58.2(4) avg: 42.7(2)
**$Cu_{27}MePO_3$ (1)** Cu_9-ring	2.85(18)–60.6(3) avg: 38.7(2)	0.15(13)–11.98(12) avg: 4.9(2)	1.0(2)–60.4(3) avg: 37.9(2)
**$Cu_{27}MePO_3$ (1)** Cu_6-ring	45.9(2)–65.99(19) avg: 56.1(2)	0.33(14)–4.73(13) avg: 1.9(2)	45.8(2)–66.00(19) avg: 56.4(2)
Avg. **$Cu_{27}MePO_3$ (1)**	*45.9(2)*	*3.4(2)*	*45.7(2)*
**$Cu_{29}MePO_3$ (2)** Cu_{13}-ring	12.6(5)–54.1(4) avg: 40.9(4)<br>12.6(4)–53.1(4) avg: 41.0(4)	0.0(4)–17.5(2) avg: 5.1(3)<br>0.9(2)–10.5(2) avg: 5.4(3)	12.6(5)–57.8(8) avg: 41.6(4)<br>11.8(4)–59.6(4) avg: 41.6(4)
**$Cu_{29}MePO_3$ (2)** Cu_9-ring	18.9(3)–59.5(4) avg: 43.9(4)<br>20.4(4)–63.1(3) avg: 44.4(4)	1.4(3)–12.8(2) avg: 5.2(3)<br>1.1(3)–13.5(2) avg: 4.8(3)	14.2(4)–60.4(4) avg: 43.0(4)<br>15.6(4)–63.1(3) avg: 43.8(4)
**$Cu_{29}MePO_3$ (2)** Cu_7-ring	24.4(4)–76.5(3) avg: 48.6(4)<br>13.9(3)–78.5(3) avg: 46.9(3)	0.2(3)–13.7(3) avg: 5.0(3)<br>0.4(3)–9.5(3) avg: 4.4(3)	20.5(4)–76.4(3) avg: 48.0(4)<br>10.3(4)–78.4(3) avg: 46.3(4)
Avg. **$Cu_{29}MePO_3$ (2)**	*44.5(4)*<br>*44.1(4)*	*5.1(3)*<br>*4.9(3)*	*46.0(4)*<br>*43.9(4)*
**$Cu_{31}MePO_3$ (3)** Cu_{14}-ring	9.9(7)–56.6(4) avg: 42.4(5)	1.2(4)–15.3(4) avg: 6.2(4)	9.5(8)–62.1(6) avg: 42.8(5)
**$Cu_{31}MePO_3$ (3)** Cu_9-ring	28.8(5)–71.0(4) avg: 49.1(5)	0.5(4)–9.8(3) avg: 4.5(4)	28.1(5)–71.3(4) avg: 49.2(5)
**$Cu_{31}MePO_3$ (3)** Cu_8-ring	23.9(4)–64.5(5) avg: 47.4(5)	1.1(3)–10.2(3) avg: 3.9(4)	21.7(4)–65.2(5) avg: 47.2(5)
Avg. **$Cu_{31}MePO_3$ (3)**	*46.3(5)*	*4.9(4)*	*46.4(5)*
**$Cu_{31}EtPO_3$ (4)** Cu_{14}-ring	3.1(7)–52.4(5) avg: 40.7(6)	2.2(6)–14.6(3) avg: 5.8(5)	2.2(8)–60.9(9) avg: 41.0(6)
**$Cu_{31}EtPO_3$ (4)** Cu_9-ring	20.6(5)–67.9(6) avg: 45.2(6)	0.1(4)–9.8(3) avg: 5.2(4)	19.7(5)–67.9(6) avg: 45.8(6)
**$Cu_{31}EtPO_3$ (4)** Cu_8-ring	21.1(6)–68.6(5) avg: 46.9(6)	0.4(4)–11.4(4) avg: 4.5(4)	17.9(6)–68.6(5) avg: 46.2(6)
Avg. **$Cu_{31}EtPO_3$ (4)**	*44.3(6)*	*5.2(4)*	*44.3(6)*
**$Cu_{31}EtPO_3$ (5)** Cu_{14}-ring	14.5(5)–57.0(3) avg: 42.2(4)	0.3(2)–14.8(3) avg: 6.1(3)	14.4(5)–60.7(3) avg: 43.1(4)
**$Cu_{31}EtPO_3$ (5)** Cu_9-ring	17.4(3)–67.5(3) avg: 45.4(3)	0.35(17)–11.4(3) avg: 4.4(3)	17.4(3)–67.9(3) avg: 45.3(3)
**$Cu_{31}EtPO_3$ (5)** Cu_8-ring	17.2(4)–64.9(3) avg: 45.6(4)	0.0(2)–7.5(3) avg: 3.0(3)	17.2(4)–64.8(3) avg: 45.6(4)
Avg. **$Cu_{31}EtPO_3$ (5)**	*44.4(4)*	*4.5(3)*	*44.7(4)*
**$Cu_{29}EtPO_3$ (5)** Cu_{13}-ring	18(3)–57.0(3) avg: 39(3)	0.3(2)–26(2) avg: 9(2)	5(3)–60.7(3) avg: 38(3)
**$Cu_{29}EtPO_3$ (5)** Cu_9-ring	17.4(3)–67.5(3) avg: 45.4(3)	0.35(17)–11.4(3) avg: 4.4(3)	17.4(3)–67.9(3) avg: 45.3(3)
**$Cu_{29}EtPO_3$ (5)** Cu_7-ring	24(5)–70(4) avg: 54(5)	1.6(2)–9(3) avg: 5(3)	22(5)–70(4) avg: 53(5)
Avg. **$Cu_{29}EtPO_3$ (5)**	*46(3)*	*6(2)*	*45(3)*
**$Cu_{29}BuPO_3$ (6)** Cu_{13}-ring	8.8(3)–53.3(3) avg: 41.9(3)	0.5(2)–15.71(19) avg: 5.7(2)	8.1(3)–53.3(3) avg: 41.4(3)
**$Cu_{29}BuPO_3$ (6)** Cu_9-ring	10.9(3)–61.1(3) avg: 45.2(3)	0.2(2)–4.8(2) avg: 2.1(2)	9.8(3)–61.1(3) avg: 45.2(3)
**$Cu_{29}BuPO_3$ (6)** Cu_7-ring	19.2(3)–63.2(2) avg: 48.5(3)	1.48(19)–7.51(18) avg: 3.8(2)	17.7(3)–63.8(2) avg: 48.9(3)
Avg. **$Cu_{29}BuPO_3$ (6)**	*45.2(3)*	*3.9(2)*	*45.2(3)*

Table S11. Comparison of the dihedral, twist and fold angles (°) between pyrazolate moieties and adjacent Cu–O–Cu units in **7–12** (based on the major component in the case of disordered moieties).

	**DIHEDRAL ANGLE**	**TWIST ANGLE**	**FOLD ANGLE**
**$Cu_{31}{}^{n}C_{12}PO_3$ (7)** Cu_{14}-ring	11.6(4)–56.2(3) avg: 42.0(3)<br>4.2(8)–55.1(3) avg: 39.9(3)	0.0(4)–12.0(3) avg: 6.2(3)<br>0.0(2)–12.6(3) avg: 5.7(3)	8.8(5)–62.0(4) avg: 41.9(3)<br>4.1(8)–65.5(5) avg: 40.3(5)
**$Cu_{31}{}^{n}C_{12}PO_3$ (7)** Cu_9-ring	13.9(4)–62.6(3) avg: 47.3(3)<br>11.4(3)–68.1(3) avg: 47.3(3)	0.4(2)–9.4(3) avg: 2.8(3)<br>0.4(3)–9.9(3) avg: 3.0(3)	11.9(4)–62.6(3) avg: 47.0(3)<br>9.3(4)–68.1(3) avg: 46.9(3)
**$Cu_{31}{}^{n}C_{12}PO_3$ (7)** Cu_8-ring	24.4(3)–69.0(3) avg: 47.2(3)<br>22.4(3)–67.0(3) avg: 46.8(3)	1.1(2)–11.0(3) avg: 4.1(3)<br>0.2(2)–8.6(2) avg: 3.8(2)	24.4(3)–70.4(3) avg: 47.7(3)<br>22.5(4)–67.0(3) avg: 47.8(3)
Avg. **$Cu_{31}{}^{n}C_{12}PO_3$ (7)**	*45.5(3)*<br>*44.7(3)*	*4.4(3)*<br>*4.2(3)*	*45.5(3)*<br>*45.0(3)*
**$Cu_{31}BnPO_3$ (8)** Cu_{14}-ring	11.0(6)–54.3(4)<br>avg: 41.6(6)	0.9(3)–16.2(4)<br>avg: 5.9(4)	10.5(6)–59.1(6)<br>avg: 41.9(6)
**$Cu_{31}BnPO_3$ (8)** Cu_9-ring	5.4(6)–68.6(8)<br>avg: 46.2(8)	0.7(6)–8.7(4)<br>avg: 3.1(6)	1.0(14)–68.6(8)<br>avg: 45.8(8)
**$Cu_{31}BnPO_3$ (8)** Cu_8-ring	23.4(4)–68.1(3)<br>avg: 49.0(4)	0.4(3)–8.4(3)<br>avg: 3.6(3)	22.7(5)–68.9(3)<br>avg: 48.9(4)
Avg. **$Cu_{31}BnPO_3$ (8)**	*45.6(6)*	*4.2(4)*	*45.5(6)*
**$Cu_{28}PhPO_3$ (9)** Cu_{12}-ring	33.9(3)–56.0(3) avg: 43.4(3)<br>32.6(3)–53.9(3) avg: 42.9(3)	1.0(2)–8.4(2) avg: 4.7(2)<br>1.01(17)–7.89(17) avg: 4.3(2)	23.1(13)–55.8(3) avg: 41.1(3)<br>27.7(16)–53.8(3) avg: 41.6(3)
**$Cu_{28}PhPO_3$ (9)** Cu_{10}-ring	33.9(7)–61.4(3) avg: 52.2(5)<br>34.9(3)–62.4(3) avg: 51.7(3)	0.5(4)–10.8(3) avg: 3.5(4)<br>0.0(2)–10.3(3) avg: 3.0(3)	32.5(7)–61.4(3) avg: 51.8(5)<br>33.6(3)–62.4(3) avg: 51.5(3)
**$Cu_{28}PhPO_3$ (9)** Cu_6-ring	27.6(3)–65.2(2) avg: 50.1(3)<br>29.0(3)–65.2(2) avg: 50.2(3)	0.6(2)–4.7(3) avg: 1.8(2)<br>0.23(19)–1.5(2) avg: 0.9(2)	27.6(3)–65.3(2) avg: 50.1(3)<br>28.9(3)–65.2(2) avg: 50.2(3)
Avg. **$Cu_{28}PhPO_3$ (9)**	*48.6(3)*<br>*48.3(3)*	*3.3(2)*<br>*2.7(2)*	*47.7(3)*<br>*47.8(3)*
**$Cu_{27}PhPO_3$ (9)** Cu_{12}-ring	33.9(3)–56.0(3) avg: 43.4(3)<br>32.6(3)–53.9(3) avg: 42.9(3)	1.0(2)–8.4(2) avg: 4.7(2)<br>1.01(17)–7.89(17) avg: 4.3(2)	23.1(13)–55.8(3) avg: 41.1(3)<br>27.7(16)–53.8(3) avg: 41.6(3)
**$Cu_{27}PhPO_3$ (9)** Cu_9-ring	10.9(17)–61.4(3) avg: 42.9(3)<br>5(2)–69.7(6) avg: 42.3(6)	1.4(2)–17.6(17) avg: 5.6(2)<br>0.6(8)–19.5(9) avg: 6.0(9)	10.1(18)–61.4(3) avg: 41.3(3)<br>3(2)–68.6(6) avg: 41.7(6)
**$Cu_{27}PhPO_3$ (9)** Cu_6-ring	27.6(3)–65.2(2) avg: 50.1(3)<br>29.0(3)–65.2(2) avg: 50.2(3)	0.6(2)–4.7(3) avg: 1.8(2)<br>0.23(19)–1.5(2) avg: 0.9(2)	27.6(3)–65.3(2) avg: 50.1(3)<br>28.9(3)–65.2(2) avg: 50.2(3)
Avg. **$Cu_{27}PhPO_3$ (9)**	*45.5(3)*<br>*45.1(3)*	*4.0(2)*<br>*3.7(2)*	*44.2(3)*<br>*44.5(3)*
**$Cu_{31}PhPO_3$ (10)** Cu_{14}-ring	3.99(12)–55.11(16)<br>avg: 40.8(2)	0.7(2)–13.53(18)<br>avg: 5.4(2)	0.31(18)–69.2(5)<br>avg: 43.2(2)
**$Cu_{31}PhPO_3$ (10)** Cu_9-ring	12.2(2)–64.92(17)<br>avg: 45.5(2)	0.85(11)–7.17(11)<br>avg: 3.1(2)	11.0(2)–64.91(17)<br>avg: 45.1(2)
**$Cu_{31}PhPO_3$ (10)** Cu_8-ring	25.5(4)–66.5(2)<br>avg: 47.3(2)	0.86(11)–12.28(14)<br>avg: 5.2(2)	25.1(4)–66.5(2)<br>avg: 46.8(2)
Avg. **$Cu_{31}PhPO_3$ (10)**	*44.5(2)*	*4.6(2)*	*45.0(2)*
**$(Cu_{27}EtPO_3)_2$ (11)** Cu_{12}-ring	31.00(15)–50.77(13)<br>avg: 41.9(2)	0.12(11)–6.49(10)<br>avg: 3.2(1)	31.00(15)–50.75(13)<br>avg: 41.7(2)
**$(Cu_{27}EtPO_3)_2$ (11)** Cu_9-ring	5.76(14)–57.04(13)<br>avg: 37.1(2)	1.16(9)–14.24(9)<br>avg: 5.0(1)	5.59(14)–57.01(13)<br>avg: 36.7(2)
**$(Cu_{27}EtPO_3)_2$ (11)** Cu_6-ring	38.7(2)–64.16(14)<br>avg: 54.7(2)	0.48(10)–4.36(18)<br>avg: 2.6(2)	38.5(2)–64.19(14)<br>avg: 54.8(2)
Avg. **$(Cu_{27}EtPO_3)_2$ (11)**	*44.6(2)*	*3.6(1)*	*44.4(2)*
**$(Cu_{27}PrPO_3)_2$ (12)** Cu_{12}-ring	28.5(2)–58.87(18) avg: 41.5(2)<br>30.95(19)–56.07(17) avg: 42.7(2)	0.42(12)–10.46(14) avg: 4.8(2)<br>0.05(14)–9.20(13) avg: 4.1(2)	21.6(8)–58.69(18) avg: 39.6(2)<br>30.80(19)–55.99(17) avg: 42.5(2)
**$(Cu_{27}PrPO_3)_2$ (12)** Cu_9-ring	36.45(18)–63.35(17) avg: 50.5(2)<br>42.0(2)–62.14(17) avg: 51.3(2)	0.84(14)–10.19(13) avg: 3.7(2)<br>0.32(13)–7.38(14) avg: 3.5(2)	36.12(18)–63.34(17) avg: 50.3(2)<br>41.5(2)–62.15(17) avg: 51.5(2)
**$(Cu_{27}PrPO_3)_2$ (12)** Cu_6-ring	29.51(17)–65.69(16) avg: 53.9(2)<br>29.34(17)–68.52(17) avg: 54.5(2)	0.38(15)–3.08(12) avg: 1.7(2)<br>0.38(15)–3.98(12) avg: 1.8(2)	29.41(17)–65.68(16) avg: 54.0(2)<br>29.32(17)–69.20(17) avg: 54.6(2)
Avg. **$(Cu_{27}PrPO_3)_2$ (12)**	*49.0(2)*<br>*49.1(2)*	*3.2(2)*<br>*3.4(2)*	*48.9(2)*<br>*48.6(2)*

Table S12. Comparison of the dihedral, twist and fold angles (°) between adjacent pyrazolate moieties in **1**–**5** (based on the major component in the case of disordered moieties).

	DIHEDRAL ANGLE	TWIST ANGLE	FOLD ANGLE	CENTROID-CENTROID DISTANCE	
				D. A. > 35°	D. A. < 35°
$\mathbf{Cu_{27}MePO_3}$ **(1)** Cu_{12}-ring	46.68(17)–71.1(4)<br>avg: 55.2(2)	47.07(18)–72.4(5)<br>avg: 56.1(2)	3.1(3)–26.8(3)<br>avg: 12.3(3)	4.765(3)–5.167(6)<br>avg. of 12: 4.871(3)	–
$\mathbf{Cu_{27}MePO_3}$ **(1)** Cu_{9}-ring	17.23(17)–64.20(18)<br>avg: 39.7(2)	15.44(17)–57.58(19)<br>avg: 37.8(2)	0.50(15)–40.33(18)<br>avg: 12.9(2)	4.833(3)–5.150(5)<br>avg of 5: 4.943(3)	4.963(3)–5.123(4)<br>avg of 4: 5.043(3)
$\mathbf{Cu_{27}MePO_3}$ **(1)** Cu_{6}-ring	24.55(17)–35.79(17)<br>avg: 29.6(2)	0.8(2)–16.95(18)<br>avg: 9.9(2)	20.36(17)–35.78(17)<br>avg: 27.1(2)	–	5.008(3)–5.100(4)<br>avg of 6: 5.058(3)
Avg. $\mathbf{Cu_{27}MePO_3}$ **(1)**	*41.5(2)*	*34.6(2)*	*17.4(2)*	*4.907(3)*	*5.051(3)*
$\mathbf{Cu_{29}MePO_3}$ **(2)** Cu_{13}-ring	43.5(5)–69.3(4)<br>avg: 57.4(5)<br>38.8(4)–67.8(4)<br>avg: 57.4(4)	40.2(5)–69.8(4)<br>avg: 57.2(5)<br>38.8(4)–68.9(3)<br>avg: 57.8(4)	4.2(4)–45.8(8)<br>avg: 24.7(5)<br>2.8(4)–42.3(6)<br>avg: 20.2(4)	4.692(5)–4.927(4)<br>avg. of 13: 4.839(5)<br>4.734(4)–4.887(5)<br>avg. of 13: 4.831(5)	–<br>–
$\mathbf{Cu_{29}MePO_3}$ **(2)** Cu_{9}-ring	19.3(3)–56.4(3)<br>avg: 38.3(3)<br>18.7(3)–57.5(3)<br>avg: 39.1(3)	1.4(4)–57.5(3)<br>avg: 34.3(4)<br>0.7(3)–58.8(3)<br>avg: 34.7(3)	1.2(9)–59.0(8)<br>avg: 22.3(4)<br>1.7(3)–43.9(6)<br>avg: 22.6(3)	4.919(6)–4.976(4)<br>avg of 5: 4.949(5)<br>4.923(5)–4.998(5)<br>avg of 5: 4.961(5)	4.954(5)–5.178(4)<br>avg of 4: 5.093(5)<br>4.909(4)–5.192(4)<br>avg of 4: 5.069(4)
$\mathbf{Cu_{29}MePO_3}$ **(2)** Cu_{7}-ring	13.2(4)–58.5(4)<br>avg: 46.6(5)<br>13.2(4)–58.5(4)<br>avg: 46.6(5)	3.9(4)–36.5(4)<br>avg: 21.1(4)<br>3.9(4)–36.5(4)<br>avg: 21.1(4)	12.3(4)–73.8(11)<br>avg: 28.1(4)<br>12.3(4)–73.8(11)<br>avg: 28.1(4)	4.877(5)–5.036(4)<br>avg of 3: 4.887(5)<br>4.903(4)–5.003(4)<br>avg of 5: 4.948(4)	4.868(5)–5.109(5)<br>avg of 4: 5.064(5)<br>5.041(4), 5.084(5)<br>avg of 2: 5.063(5)
Avg. $\mathbf{Cu_{29}MePO_3}$ **(2)**	*42.7(4)*<br>*45.5(4)*	*37.5(4)*<br>*41.2(4)*	*25.0(4)*<br>*29.0(4)*	*4.892(5)*<br>*4.913(5)*	*5.079(5)*<br>*5.066(5)*
$\mathbf{Cu_{31}MePO_3}$ **(3)** Cu_{14}-ring	44.8(4)–68.6(5)<br>avg: 56.8(5)	29.8(5)–65.9(5)<br>avg: 55.6(5)	0.3(5)–49.7(8)<br>avg: 23.9(5)	4.700(7)–4.929(6)<br>avg. of 14: 4.818(7)	–
$\mathbf{Cu_{31}MePO_3}$ **(3)** Cu_{9}-ring	7.2(5)–63.1(5)<br>avg: 39.5(5)	2.3(5)–61.0(5)<br>avg: 37.3(5)	0.8(4)–29.8(12)<br>avg: 14.9(5)	4.812(7)–4.928(8)<br>avg of 6: 4.885(7)	5.030(6)–5.155(7)<br>avg of 3: 5.112(7)
$\mathbf{Cu_{31}MePO_3}$ **(3)** Cu_{8}-ring	21.6(4)–63.5(4)<br>avg: 37.6(4)	7.2(4)–55.4(4)<br>avg: 31.3(4)	0.3(5)–30.5(4)<br>avg: 17.7(4)	4.856(7)–4.966(6)<br>avg of 5: 4.892(6)	5.075(5)–5.118(9)<br>avg of 3: 5.093(6)
Avg. $\mathbf{Cu_{31}MePO_3}$ **(3)**	*44.6(5)*	*41.4(5)*	*18.8(5)*	*4.865(7)*	*5.103(7)*
$\mathbf{Cu_{31}EtPO_3}$ **(4)** Cu_{14}-ring	45.0(5)–68.6(6)<br>avg: 56.0(6)	33.8(6)–67.3(6)<br>avg: 55.6(6)	1.5(7)–53.9(10)<br>avg: 17.9(7)	4.668(10)–4.907(10)<br>avg. of 14: 4.802(10)	–
$\mathbf{Cu_{31}EtPO_3}$ **(4)** Cu_{9}-ring	14.5(5)–61.6(6)<br>avg: 37.1(5)	14.0(5)–58.3(6)<br>avg: 35.6(5)	3.1(5)–25.6(7)<br>avg: 13.5(5)	4.817(5)–4.930(8)<br>avg of 6: 4.886(8)	5.079(5)–5.135(9)<br>avg of 3: 5.113(8)
$\mathbf{Cu_{31}EtPO_3}$ **(4)** Cu_{8}-ring	23.1(4)–64.4(4)<br>avg: 42.3(4)	5.7(4)–64.6(4)<br>avg: 36.3(4)	5.3(6)–50.9(15)<br>avg: 27.9(6)	4.806(6)–5.066(6)<br>avg of 6: 4.916(6)	5.085(7), 5.121(8)<br>avg of 2: 5.103(7)
Avg. $\mathbf{Cu_{31}EtPO_3}$ **(4)**	*45.1(5)*	*42.5(5)*	*19.8(5)*	*4.868(8)*	*5.108(8)*
$\mathbf{Cu_{31}EtPO_3}$ **(5)** Cu_{14}-ring	43.6(4)–68.8(3)<br>avg: 56.9(4)	35.9(4)–68.5(4)<br>avg: 56.7(4)	3.3(4)–68.7(5)<br>avg: 28.7(4)	4.691(5)–4.944(5)<br>avg. of 14: 4.818(5)	–
$\mathbf{Cu_{31}EtPO_3}$ **(5)** Cu_{9}-ring	7.1(4)–57.8(3)<br>avg: 37.1(3)	1.9(3)–53.1(3)<br>avg: 33.9(3)	1.1(3)–43.3(6)<br>avg: 18.5(3)	4.828(5)–4.948(6)<br>avg of 6: 4.898(5)	5.106(4)–5.161(6)<br>avg of 3: 5.135(5)
$\mathbf{Cu_{31}EtPO_3}$ **(5)** Cu_{8}-ring	14.6(3)–59.1(4)<br>avg: 39.8(4)	3.0(3)–58.3(4)<br>avg: 31.7(4)	11.2(4)–37.6(3)<br>avg: 25.0(4)	4.793(5)–5.029(5)<br>avg of 6: 4.882(5)	5.093(6), 5.108(5)<br>avg of 2: 5.101(5)
Avg. $\mathbf{Cu_{31}EtPO_3}$ **(5)**	*44.6(4)*	*40.8(4)*	*24.1(4)*	*4.866(5)*	*5.118(5)*
$\mathbf{Cu_{29}EtPO_3}$ **(5)** Cu_{13}-ring	16(4)–68.8(3)<br>avg: 52(4)	5(4)–68.5(4)<br>avg: 47(4)	3.3(4)–68.7(5)<br>avg: 29(4)	4.40(3)–5.29(2)<br>avg. of 14: 4.81(3)	–
$\mathbf{Cu_{29}EtPO_3}$ **(5)** Cu_{9}-ring	7.1(4)–57.8(3)<br>avg: 37.1(3)	1.9(3)–53.1(3)<br>avg: 33.9(3)	1.1(3)–43.3(6)<br>avg: 18.5(3)	4.828(5)–4.948(6)<br>avg of 6: 4.898(5)	5.106(4)–5.161(6)<br>avg of 3: 5.135(5)
$\mathbf{Cu_{29}EtPO_3}$ **(5)** Cu_{7}-ring	13(3)–91(4)<br>avg: 51(4)	0(3)–85(5)<br>avg: 43(4)	13(3)–74(8)<br>avg: 39(4)	4.78(4)–5.02(5)<br>avg of 5: 4.90(5)	5.15(5), 5.48(5)<br>avg of 2: 5.32(5)
Avg. $\mathbf{Cu_{29}EtPO_3}$ **(5)**	*47(4)*	*41(4)*	*29(4)*	*4.87(5)*	*5.23(5)*

Table S13. Comparison of the dihedral, twist and fold angles (°) between adjacent pyrazolate moieties in **6–9** (based on the major component in the case of disordered moieties).

	DIHEDRAL ANGLE	TWIST ANGLE	FOLD ANGLE	CENTROID-CENTROID DISTANCE	
				D. A. > 35°	D. A. < 35°
**$Cu_{29}{}^{n}BuPO_3$ (6)** Cu_{13}-ring	40.1(3)–70.2(3)<br>avg: 56.9(3)	37.1(3)–68.2(3)<br>avg: 57.2(3)	4.1(4)–27.9(4)<br>avg: 16.1(4)	4.703(4)–4.924(4)<br>avg. of 13: 4.826(4)	–
**$Cu_{29}{}^{n}BuPO_3$ (6)** Cu_9-ring	17.6(4)–51.2(3)<br>avg: 37.5(3)	2.6(4)–50.8(3)<br>avg: 34.4(3)	0.3(3)–27.7(3)<br>avg: 10.5(3)	4.892(4)–4.956(4)<br>avg of 6: 4.917(4)	5.121(4)–5.131(4)<br>avg of 3: 5.127(4)
**$Cu_{29}{}^{n}BuPO_3$ (6)** Cu_7-ring	19.8(2)–53.3(2)<br>avg: 36.8(2)	4.0(2)–41.9(3)<br>avg: 24.9(3)	5.6(3)–40.7(2)<br>avg: 23.8(3)	4.893(4)–5.001(4)<br>avg of 5: 4.947(4)	5.068(4), 5.084(4)<br>avg of 2: 5.076(4)
Avg. **$Cu_{29}{}^{n}BuPO_3$ (6)**	*43.7(3)*	*38.8(3)*	*16.8(3)*	*4.897(4)*	*5.102(4)*
**$Cu_{29}{}^{n}C_{12}PO_3$ (7)** Cu_{14}-ring	43.8(10)–70.6(11)<br>avg: 57.7(3)<br>40.3(11)–66.7(3)<br>avg: 55.9(4)	37.2(11)–68.8(10)<br>avg: 56.6(3)<br>36.6(4)–66.3(4)<br>avg: 55.2(4)	3.5(4)–48.4(5)<br>avg: 19.9(3)<br>1.6(6)–54.0(5)<br>avg: 17.7(5)	4.729(5)–4.947(6)<br>avg. of 14: 4.827(6)<br>4.695(5)–4.911(6)<br>avg. of 14: 4.817(5)	–
**$Cu_{29}{}^{n}C_{12}PO_3$ (7)** Cu_9-ring	14.7(9)–60.7(3)<br>avg: 41.4(3)<br>7.7(4)–60.4(3)<br>avg: 40.1(4)	3.3(3)–57.6(3)<br>avg: 36.8(3)<br>3.0(3)–57.3(4)<br>avg: 36.2(4)	1.8(10)–41.6(6)<br>avg: 21.5(3)<br>2.6(4)–39.0(5)<br>avg: 20.3(4)	4.874(5)–4.939(4)<br>avg of 6: 4.906(4)<br>4.875(5)–4.938(3)<br>avg of 6: 4.908(4)	5.128(6)–5.155(5)<br>avg of 3: 5.141(5)<br>5.140(5)–5.172(5)<br>avg of 3: 5.153(5)
**$Cu_{29}{}^{n}C_{12}PO_3$ (7)** Cu_8-ring	23.3(3)–68.3(3)<br>avg: 47.3(3)<br>22.4(3)–63.6(3)<br>avg: 39.3(3)	6.6(3)–65.0(3)<br>avg: 41.5(3)<br>14.7(3)–61.9(4)<br>avg: 34.7(4)	5.2(3)–71.4(8)<br>avg: 37.6(3)<br>14.9(4)–42.1(6)<br>avg: 23.0(4)	4.848(5)–5.006(4)<br>avg of 7: 4.922(4)<br>4.778(6)–4.971(4)<br>avg of 5: 4.880(4)	5.056(4)<br><br>4.987(4)–5.123(4)<br>avg of 3: 5.070(4)
Avg. **$Cu_{29}{}^{n}C_{12}PO_3$ (7)**	*48.8(3)*<br>*45.1(3)*	*45.0(3)*<br>*42.0(3)*	*26.3(3)*<br>*20.3(3)*	*4.885(4)*<br>*4.868(4)*	*5.098(4)*<br>*5.112(4)*
**$Cu_{31}BnPO_3$ (8)** Cu_{14}-ring	45.1(4)–66.8(3)<br>avg: 56.1(4)	36.9(6)–68.1(4)<br>avg: 55.4(4)	1.2(7)–43.2(9)<br>avg: 22.2(7)	4.591(8)–4.910(5)<br>avg. of 14: 4.801(6)	–
**$Cu_{31}BnPO_3$ (8)** Cu_9-ring	10.0(4)–63.8(5)<br>avg: 40.4(5)	8.4(5)–63.7(5)<br>avg: 38.9(5)	5.5(4)–35.4(14)<br>avg: 15.5(5)	4.847(8)–5.006(7)<br>avg of 6: 4.992(8)	5.074(7)–5.251(12)<br>avg of 3: 5.163(8)
**$Cu_{31}BnPO_3$ (8)** Cu_8-ring	17.1(3)–59.7(3)<br>avg: 37.3(3)	3.1(3)–57.7(3)<br>avg: 30.8(3)	0.8(4)–35.7(3)<br>avg: 17.4(4)	4.818(5)–4.922(5)<br>avg of 5: 4.864(5)	5.085(4)–5.116(7)<br>avg of 3: 5.096(5)
Avg. **$Cu_{31}BnPO_3$ (8)**	*44.6(4)*	*41.7(4)*	*18.4(4)*	*4.886(6)*	*5.130(6)*
**$Cu_{28}PhPO_3$ (9)** Cu_{12}-ring	49.4(3)–67.6(3)<br>avg: 56.9(3)<br>49.5(2)–64.7(3)<br>avg: 55.8(3)	50.6(3)–65.7(4)<br>avg: 56.9(3)<br>50.9(3)–62.9(3)<br>avg: 55.8(3)	1.6(3)–57.3(5)<br>avg: 21.4(4)<br>0.1(3)–38.9(6)<br>avg: 19.3(3)	4.707(4)–4.909(4)<br>avg. of 12: 4.825(4)<br>4.748(3)–4.882(4)<br>avg. of 12: 4.821(4)	–<br><br>–
**$Cu_{28}PhPO_3$ (9)** Cu_{10}-ring	18.0(5)–47.3(5)<br>avg: 38.9(5)<br>14.2(4)–53.7(3)<br>avg: 38.6(4)	16.8(5)–48.1(5)<br>avg: 38.9(5)<br>9.9(4)–51.9(3)<br>avg: 37.9(4)	0.4(4)–17.4(3)<br>avg: 7.6(4)<br>2.1(4)–21.6(3)<br>avg: 9.8(4)	4.780(5)–4.958(4)<br>avg of 8: 4.866(5)<br>4.791(4)–4.970(4)<br>avg of 8: 4.862(4)	5.046(5), 5.057(6)<br>avg of 2: 5.052(5)<br>5.048(4), 5.128(5)<br>avg of 2: 5.088(4)
**$Cu_{28}PhPO_3$ (9)** Cu_6-ring	26.1(2)–52.2(3)<br>avg: 41.2(3)<br>29.7(2)–48.5(3)<br>avg: 40.9(3)	1.8(3)–38.3(3)<br>avg: 22.5(3)<br>2.4(3)–36.0(3)<br>avg: 22.0(3)	25.2(2)–61.6(8)<br>avg: 41.2(3)<br>28.9(2)–60.6(8)<br>avg: 42.1(3)	4.860(4)–4.973(3)<br>avg of 5: 4.899(4)<br>4.885(3)–4.966(3)<br>avg of 5: 4.912(3)	5.049(3)<br><br>5.016(3)
Avg. **$Cu_{28}PhPO_3$ (9)**	*45.7(4)*<br>*45.1(3)*	*39.4(4)*<br>*38.6(3)*	*23.4(4)*<br>*23.7(3)*	*4.863(4)*<br>*4.865(4)*	*5.051(4)*<br>*5.052(4)*
**$Cu_{27}PhPO_3$ (9)** Cu_{12}-ring	49.4(3)–67.6(3)<br>avg: 56.9(3)<br>49.5(2)–64.7(3)<br>avg: 55.8(3)	50.6(3)–65.7(4)<br>avg: 56.9(3)<br>50.9(3)–62.9(3)<br>avg: 55.8(3)	1.6(3)–57.3(5)<br>avg: 21.4(4)<br>0.1(3)–38.9(6)<br>avg: 19.3(3)	4.707(4)–4.909(4)<br>avg. of 12: 4.825(4)<br>4.748(3)–4.882(4)<br>avg. of 12: 4.821(4)	–<br><br>–
**$Cu_{27}PhPO_3$ (9)** Cu_9-ring	19.5(17)–46.4(3)<br>avg: 35(3)<br>4(3)–54(2)<br>avg: 32(3)	8.8(19)–46.6(3)<br>avg: 32(3)<br>14(3)–54(2)<br>avg: 32(3)	0.5(15)–23(3)<br>avg: 12(3)<br>0(3)–18.9(3)<br>avg: 6(3)	4.922(4)–5.67(2)<br>avg of 4: 5.14(2)<br>4.90(3)–5.305(17)<br>avg of 4: 5.02(2)	4.792(17)–5.240(14)<br>avg of 5: 5.01(2)<br>4.96(2)–5.23(2)<br>avg of 5: 5.06(2)
**$Cu_{27}PhPO_3$ (9)** Cu_6-ring	26.1(2)–52.2(3)<br>avg: 41.2(3)<br>29.7(2)–48.5(3)<br>avg: 40.9(3)	1.8(3)–38.3(3)<br>avg: 22.5(3)<br>2.4(3)–36.0(3)<br>avg: 22.0(3)	25.2(2)–61.6(8)<br>avg: 41.2(3)<br>28.9(2)–60.6(8)<br>avg: 42.1(3)	4.860(4)–4.973(3)<br>avg of 5: 4.899(4)<br>4.885(3)–4.966(3)<br>avg of 5: 4.912(3)	5.03(2)<br><br>5.04(2)
Avg. **$Cu_{27}PhPO_3$ (9)**	*44.4(3)*<br>*42.9(3)*	*37.1(3)*<br>*36.6(3)*	*24.9(3)*<br>*22.5(3)*	*4.955(4)*<br>*4.918(4)*	*5.079(5)*<br>*5.066(5)*

Table S14. Comparison of the dihedral, twist and fold angles (°) between adjacent pyrazolate moieties in **10–12** (based on the major component in the case of disordered moieties). In **11** and **12**, two $Cu_{27}RPO_3$ nanojar units (symmetry-related in **11**) are tethered by two adjacent RPO_3^{2-} moieties; therefore, only seven pyrazolate units are present in their Cu_9-rings.

	DIHEDRAL ANGLE	TWIST ANGLE	FOLD ANGLE	CENTROID-CENTROID DISTANCE	
				D. A. > 35°	D. A. < 35°
**$Cu_{31}PhPO_3$ (10)** Cu_{14}-ring	45.0(2)–64.5(2)<br>avg: 56.2(2)	34.7(3)–64.8(2)<br>avg: 55.5(2)	1.6(2)–75.7(6)<br>avg: 31.9(2)	4.693(3)–4.931(2)<br>avg. of 14: 4.821(3)	–
**$Cu_{31}PhPO_3$ (10)** Cu_9-ring	11.24(16)–63.51(15)<br>avg: 41.8(2)	4.51(15)–61.55(16)<br>avg: 37.8(2)	3.78(18)–39.7(4)<br>avg: 19.4(2)	4.844(2)–4.914(2)<br>avg of 6: 4.881(2)	5.105(2)–5.168(3)<br>avg of 3: 5.140(3)
**$Cu_{31}PhPO_3$ (10)** Cu_8-ring	24.9(7)–64.05(18)<br>avg: 47.6(2)	7.4(7)–63.06(16)<br>avg: 42.2(2)	0.7(6)–77.6(10)<br>avg: 29.2(2)	4.837(8)–5.007(3)<br>avg of 7: 4.919(5)	5.436(8)
Avg. **$Cu_{31}PhPO_3$ (10)**	*48.5(2)*	*45.2(2)*	*26.8(2)*	*4.874(3)*	*5.288(3)*
**$(Cu_{27}EtPO_3)_2$ (11)** Cu_{12}-ring	51.10(13)–60.13(13)<br>avg: 55.9(2)	51.34(14)–60.47(14)<br>avg: 56.6(2)	2.6(2)–23.26(15)<br>avg: 13.3(2)	4.742(3)–4.899(2)<br>avg. of 12: 4.836(2)	–
**$(Cu_{27}EtPO_3)_2$ (11)** Cu_9-ring	26.51(11)–55.75(12)<br>avg: 40.4(2)	25.81(12)–53.79(12)<br>avg: 39.2(2)	0.89(13)–24.11(14)<br>avg: 12.6(3)	4.872(2)–4.983(2)<br>avg of 4: 4.940(2)	4.961(2), 5.009(2)<br>avg of 2: 4.985(2)
**$(Cu_{27}EtPO_3)_2$ (11)** Cu_6-ring	21.86(12)–43.15(14)<br>avg: 30.3(2)	2.79(13)–18.0(2)<br>avg: 8.7(2)	19.2(2)–42.91(14)<br>avg: 28.5(2)	4.923(3), 4.977(2)<br>avg of 2: 4.950(2)	5.036(3)–5.091(2)<br>avg of 4: 5.066(2)
Avg. **$(Cu_{27}EtPO_3)_2$ (11)**	*42.2(2)*	*34.8(2)*	*18.0(2)*	*4.909(2)*	*5.026(2)*
**$(Cu_{27}PrPO_3)_2$ (12)** Cu_{12}-ring	40.43(19)–64.15(17)<br>avg: 55.2(2)<br>42.93(18)–64.69(17)<br>avg: 56.2(2)	41.9(2)–64.72(18)<br>avg: 55.1(2)<br>44.34(19)–65.72(19)<br>avg: 56.7(2)	3.6(2)–38.3(3)<br>avg: 22.2(3)<br>0.5(2)–27.8(2)<br>avg: 12.5(2)	4.751(3)–4.952(3)<br>avg. of 12: 4.845(3)<br>4.767(3)–4.932(3)<br>avg. of 12: 4.862(3)	–<br>–
**$(Cu_{27}PrPO_3)_2$ (12)** Cu_9-ring	14.22(18)–44.36(18)<br>avg: 34.3(2)<br>17.23(16)–52.07(19)<br>avg: 34.0(2)	2.2(2)–45.14(19)<br>avg: 29.3(2)<br>7.76(19)–52.68(19)<br>avg: 31.8(2)	3.24(19)–47(2)<br>avg: 21.7(2)<br>6.8(2)–18.54(18)<br>avg: 9.8(2)	4.952(3)–5.000(2)<br>avg of 4: 4.980(3)<br>4.922(2)–4.991(3)<br>avg of 4: 4.958(3)	5.070(3), 5.150(3)<br>avg of 2: 5.110(3)<br>5.118(3), 5.172(3)<br>avg of 2: 5.145(3)
**$(Cu_{27}PrPO_3)_2$ (12)** Cu_6-ring	19.61(15)–51.40(15)<br>avg: 34.3(2)<br>25.75(17)–45.92(15)<br>avg: 34.8(2)	8.51(17)–38.97(16)<br>avg: 19.6(2)<br>11.28(18)–34.62(16)<br>avg: 19.7(2)	15.99(15)–62(1)<br>avg: 36.4(2)<br>22.25(15)–32.83(16)<br>avg: 28.7(2)	4.934(2), 4.992(2)<br>avg of 2: 4.963(2)<br>4.935(2), 5.006(3)<br>avg of 2: 4.971(3)	5.038(2)–5.151(2)<br>avg of 4: 5.094(2)<br>5.034(2)–5.116(3)<br>avg of 4: 5.069(3)
Avg. **$(Cu_{27}PrPO_3)_2$ (12)**	*41.3(2)*<br>*41.7(2)*	*34.7(2)*<br>*36.1(2)*	*26.8(2)*<br>*17.0(2)*	*4.929(3)*<br>*4.930(3)*	*5.102(3)*<br>*5.107(3)*

Table S15. Copper coordination geometry indexes, $\tau_4 = (360 - \beta - \alpha)/141$ and $\tau_5 = (\beta - \alpha)/60$ in different Cu_x rings in **1–12** (where β and α are the two largest angles in the four- or five-coordinate species.

	τ_4	τ_5		τ_4	τ_5
**$Cu_{27}MePO_3$ (1)** Cu_{12}-ring	0.08–0.18<br>avg: 0.12	0.00–0.21<br>avg: 0.04	**$Cu_{31}{}^{n}C_{12}PO_3$ (7)** Cu_{14}-ring	0.08–0.24 avg: 0.16<br>0.09–0.23 avg: 0.16	0.00–0.12 avg: 0.06<br>0.01–0.16 avg: 0.05
**$Cu_{27}MePO_3$ (1)** Cu_9-ring	0.10–0.40<br>avg: 0.20	0.01–0.33<br>avg: 0.15	**$Cu_{31}{}^{n}C_{12}PO_3$ (7)** Cu_9-ring	0.04–0.15 avg: 0.10<br>0.04–0.16 avg: 0.10	0.00–0.12 avg: 0.05<br>0.00–0.09 avg: 0.04
**$Cu_{27}MePO_3$ (1)** Cu_6-ring	0.12–0.13<br>avg: 0.12	0.01–0.08<br>avg: 0.05	**$Cu_{31}{}^{n}C_{12}PO_3$ (7)** Cu_8-ring	0.09–0.17 avg: 0.12<br>0.10–0.13 avg: 0.12	0.03–0.28 avg: 0.13<br>0.04–0.20 avg: 0.11
Avg. **$Cu_{27}MePO_3$ (1)**	*0.15*	*0.08*	Avg. **$Cu_{31}{}^{n}C_{12}PO_3$ (7)**	*0.12*<br>*0.12*	*0.08*<br>*0.07*
**$Cu_{29}MePO_3$ (2)** Cu_{13}-ring	0.09–0.21 avg: 0.15<br>0.09–0.21 avg: 0.15	0.00–0.09 avg: 0.05<br>0.00–0.12 avg: 0.06	**$Cu_{31}BnPO_3$ (8)** Cu_{14}-ring	0.07–0.21<br>avg: 0.15	0.01–0.16<br>avg: 0.05
**$Cu_{29}MePO_3$ (2)** Cu_9-ring	0.08–0.16 avg: 0.12<br>0.07–0.21 avg: 0.12	0.01–0.14 avg: 0.05<br>0.00–0.15 avg: 0.04	**$Cu_{31}BnPO_3$ (8)** Cu_9-ring	0.06–0.18<br>avg: 0.11	0.00–0.17<br>avg: 0.08
**$Cu_{29}MePO_3$ (2)** Cu_7-ring	0.09–0.17 avg: 0.12<br>0.09–0.17 avg: 0.13	0.02–0.20 avg: 0.09<br>0.03–0.21 avg: 0.13	**$Cu_{31}BnPO_3$ (8)** Cu_8-ring	0.08–0.13<br>avg: 0.11	0.03–0.20<br>avg: 0.10
Avg. **$Cu_{29}MePO_3$ (2)**	*0.13*<br>*0.13*	*0.06*<br>*0.08*	Avg. **$Cu_{31}BnPO_3$ (8)**	*0.12*	*0.08*
**$Cu_{31}MePO_3$ (3)** Cu_{14}-ring	0.08–0.23<br>avg: 0.15	0.00–0.17<br>avg: 0.05	**$Cu_{28}PhPO_3$ (9)** Cu_{12}-ring	0.09–0.15 avg: 0.11<br>0.09–0.15 avg: 0.11	0.01–0.11 avg: 0.04<br>0.01–0.11 avg: 0.05
**$Cu_{31}MePO_3$ (3)** Cu_9-ring	0.05–0.13<br>avg: 0.09	0.01–0.10<br>avg: 0.04	**$Cu_{28}PhPO_3$ (9)** Cu_{10}-ring	0.03–0.16 avg: 0.09<br>0.05–0.15 avg: 0.09	0.00–0.17 avg: 0.07<br>0.01–0.20 avg: 0.07
**$Cu_{31}MePO_3$ (3)** Cu_8-ring	0.09–0.12<br>avg: 0.11	0.03–0.18<br>avg: 0.09	**$Cu_{28}PhPO_3$ (9)** Cu_6-ring	0.12–0.13 avg: 0.13<br>0.12–0.14 avg: 0.13	0.01–0.20 avg: 0.12<br>0.02–0.19 avg: 0.12
Avg. **$Cu_{31}MePO_3$ (3)**	*0.11*	*0.06*	Avg. **$Cu_{28}PhPO_3$ (9)**	*0.11*<br>*0.11*	*0.08*<br>*0.08*
**$Cu_{31}EtPO_3$ (4)** Cu_{14}-ring	0.08–0.22<br>avg: 0.15	0.00–0.14<br>avg: 0.04	**$Cu_{27}PhPO_3$ (9)** Cu_{12}-ring	0.09–0.15 avg: 0.11<br>0.09–0.15 avg: 0.11	0.01–0.11 avg: 0.04<br>0.01–0.11 avg: 0.05
**$Cu_{31}EtPO_3$ (4)** Cu_9-ring	0.04–0.12<br>avg: 0.09	0.02–0.10<br>avg: 0.05	**$Cu_{27}PhPO_3$ (9)** Cu_9-ring	0.08–0.26 avg: 0.16<br>0.09–0.24 avg: 0.14	0.02–0.27 avg: 0.12<br>0.05–0.25 avg: 0.11
**$Cu_{31}EtPO_3$ (4)** Cu_8-ring	0.09–0.15<br>avg: 0.12	0.01–0.17<br>avg: 0.10	**$Cu_{27}PhPO_3$ (9)** Cu_6-ring	0.12–0.13 avg: 0.13<br>0.12–0.14 avg: 0.13	0.01–0.20 avg: 0.12<br>0.02–0.19 avg: 0.12
Avg. **$Cu_{31}EtPO_3$ (4)**	*0.12*	*0.06*	Avg. **$Cu_{27}PhPO_3$ (9)**	*0.13*<br>*0.13*	*0.09*<br>*0.09*
**$Cu_{31}EtPO_3$ (5)** Cu_{14}-ring	0.08–0.23<br>avg: 0.15	0.00–0.16<br>avg: 0.05	**$Cu_{31}PhPO_3$ (10)** Cu_{14}-ring	0.08–0.24<br>avg: 0.15	0.00–0.10<br>avg: 0.05
**$Cu_{31}EtPO_3$ (5)** Cu_9-ring	0.04–0.13<br>avg: 0.09	0.01–0.13<br>avg: 0.05	**$Cu_{31}PhPO_3$ (10)** Cu_9-ring	0.05–0.18<br>avg: 0.12	0.01–0.19<br>avg: 0.06
**$Cu_{31}EtPO_3$ (5)** Cu_8-ring	0.10–0.15<br>avg: 0.12	0.01–0.19<br>avg: 0.10	**$Cu_{31}PhPO_3$ (10)** Cu_8-ring	0.09–0.17<br>avg: 0.12	0.07–0.25<br>avg: 0.14
Avg. **$Cu_{31}EtPO_3$ (5)**	*0.12*	*0.07*	Avg. **$Cu_{31}PhPO_3$ (10)**	*0.13*	*0.09*
**$Cu_{29}EtPO_3$ (5)** Cu_{13}-ring	0.08–0.22<br>avg: 0.16	0.00–0.49<br>avg: 0.11	**$(Cu_{27}EtPO_3)_2$ (11)** Cu_{12}-ring	0.09–0.16<br>avg: 0.13	0.00–0.15<br>avg: 0.06
**$Cu_{29}EtPO_3$ (5)** Cu_9-ring	0.04–0.13<br>avg: 0.09	0.01–0.13<br>avg: 0.05	**$(Cu_{27}EtPO_3)_2$ (11)** Cu_9-ring	0.09–0.35<br>avg: 0.17	0.09–0.35<br>avg: 0.17
**$Cu_{29}EtPO_3$ (5)** Cu_7-ring	0.10–0.35<br>avg: 0.18	0.01–0.27<br>avg: 0.12	**$(Cu_{27}EtPO_3)_2$ (11)** Cu_6-ring	0.12–0.13<br>avg: 0.12	0.02–0.27<br>avg: 0.13
Avg. **$Cu_{29}EtPO_3$ (5)**	*0.14*	*0.09*	Avg. **$(Cu_{27}EtPO_3)_2$ (11)**	*0.14*	*0.07*
**$Cu_{29}BuPO_3$ (6)** Cu_{13}-ring	0.08–0.20<br>avg: 0.14	0.01–0.11<br>avg: 0.05	**$(Cu_{27}PrPO_3)_2$ (12)** Cu_{12}-ring	0.07–0.16 avg: 0.12<br>0.07–0.18 avg: 0.13	0.00–0.11 avg: 0.04<br>0.00–0.09 avg: 0.03
**$Cu_{29}BuPO_3$ (6)** Cu_9-ring	0.05–0.17<br>avg: 0.11	0.00–0.31<br>avg: 0.10	**$(Cu_{27}PrPO_3)_2$ (12)** Cu_9-ring	0.07–0.12 avg: 0.10<br>0.05–0.13 avg: 0.10	0.03–0.14 avg: 0.07<br>0.00–0.12 avg: 0.06
**$Cu_{29}BuPO_3$ (6)** Cu_7-ring	0.09–0.13<br>avg: 0.12	0.02–0.19<br>avg: 0.09	**$(Cu_{27}PrPO_3)_2$ (12)** Cu_6-ring	0.11–0.14 avg: 0.12<br>0.11–0.15 avg: 0.13	0.02–0.15 avg: 0.07<br>0.05–0.15 avg: 0.09
Avg. **$Cu_{29}BuPO_3$ (6)**	*0.12*	*0.08*	Avg. **$(Cu_{27}PrPO_3)_2$ (12)**	*0.12*<br>*0.12*	*0.06*<br>*0.06*

Table S16. Comparison of the deviations (Å) of Cu atoms in different Cu_x rings from the Cu_x mean-plane in **1–12**.

	Deviation from Cu_m mean-plane		Deviation from Cu_m mean-plane
**$Cu_{27}MePO_3$ (1)** Cu_{12}-ring	0.029–0.439 avg: 0.227	**$Cu_{31}{}^nC_{12}PO_3$ (7)** Cu_{14}-ring	0.052–1.270 avg: 0.654<br>0.031–1.366 avg: 0.667
**$Cu_{27}MePO_3$ (1)** Cu_9-ring	0.014–0.519 avg: 0.331	**$Cu_{31}{}^nC_{12}PO_3$ (7)** Cu_9-ring	0.161–0.702 avg: 0.447<br>0.151–0.704 avg: 0.457
**$Cu_{27}MePO_3$ (1)** Cu_6-ring	0.013–0.049 avg: 0.028	**$Cu_{31}{}^nC_{12}PO_3$ (7)** Cu_8-ring	0.254–0.478 avg: 0.360<br>0.308–0.478 avg: 0.386
Avg. **$Cu_{27}MePO_3$ (1)**	*0.195*	Avg. **$Cu_{31}{}^nC_{12}PO_3$ (7)**	*0.222*<br>*0.246*
**$Cu_{29}MePO_3$ (2)** Cu_{13}-ring	0.085–1.032 avg: 0.547<br>0.082–0.922 avg: 0.472	**$Cu_{31}BnPO_3$ (8)** Cu_{14}-ring	0.005–1.427 avg: 0.663
**$Cu_{29}MePO_3$ (2)** Cu_9-ring	0.230–0.789 avg: 0.460<br>0.235–0.755 avg: 0.440	**$Cu_{31}BnPO_3$ (8)** Cu_9-ring	0.050–0.527 avg: 0.392
**$Cu_{29}MePO_3$ (2)** Cu_7-ring	0.056–0.371 avg: 0.236<br>0.029–0.326 avg: 0.194	**$Cu_{31}BnPO_3$ (8)** Cu_8-ring	0.246–0.499 avg: 0.374
Avg. **$Cu_{29}MePO_3$ (2)**	*0.414*<br>*0.369*	Avg. **$Cu_{31}BnPO_3$ (8)**	*0.476*
**$Cu_{31}MePO_3$ (3)** Cu_{14}-ring	0.079–1.493 avg: 0.689	**$Cu_{28}PhPO_3$ (9)** Cu_{12}-ring	0.078–0.561 avg: 0.297<br>0.124–0.584 avg: 0.342
**$Cu_{31}MePO_3$ (3)** Cu_9-ring	0.143–0.682 avg: 0.417	**$Cu_{28}PhPO_3$ (9)** Cu_{10}-ring	0.054–0.756 avg: 0.288<br>0.097–0.768 avg: 0.299
**$Cu_{31}MePO_3$ (3)** Cu_8-ring	0.299–0.500 avg: 0.392	**$Cu_{28}PhPO_3$ (9)** Cu_6-ring	0.034–0.138 avg: 0.082<br>0.053–0.155 avg: 0.098
Avg. **$Cu_{31}MePO_3$ (3)**	*0.499*	Avg. **$Cu_{28}PhPO_3$ (9)**	*0.222*<br>*0.246*
**$Cu_{31}EtPO_3$ (4)** Cu_{14}-ring	0.043–1.283 avg: 0.631	**$Cu_{27}PhPO_3$ (9)** Cu_{12}-ring	0.078–0.561 avg: 0.297<br>0.124–0.584 avg: 0.342
**$Cu_{31}EtPO_3$ (4)** Cu_9-ring	0.161–0.664 avg: 0.414	**$Cu_{27}PhPO_3$ (9)** Cu_9-ring	0.031–0.599 avg: 0.260<br>0.006–0.596 avg: 0.272
**$Cu_{31}EtPO_3$ (4)** Cu_8-ring	0.291–0.453 avg: 0.366	**$Cu_{27}PhPO_3$ (9)** Cu_6-ring	0.034–0.138 avg: 0.082<br>0.053–0.155 avg: 0.098
Avg. **$Cu_{31}EtPO_3$ (4)**	*0.470*	Avg. **$Cu_{27}PhPO_3$ (9)**	*0.213*<br>*0.237*
**$Cu_{31}EtPO_3$ (5)** Cu_{14}-ring	0.012–1.390 avg: 0.660	**$Cu_{31}PhPO_3$ (10)** Cu_{14}-ring	0.013–1.424 avg: 0.675
**$Cu_{31}EtPO_3$ (5)** Cu_9-ring	0.067–0.760 avg: 0.431	**$Cu_{31}PhPO_3$ (10)** Cu_9-ring	0.155–0.693 avg: 0.432
**$Cu_{31}EtPO_3$ (5)** Cu_8-ring	0.310–0.484 avg: 0.395	**$Cu_{31}PhPO_3$ (10)** Cu_8-ring	0.238–0.486 avg: 0.359
Avg. **$Cu_{31}EtPO_3$ (5)**	*0.495*	Avg. **$Cu_{31}PhPO_3$ (10)**	*0.489*
**$Cu_{29}EtPO_3$ (5)** Cu_{13}-ring	0.052–1.355 avg: 0.648	**$(Cu_{27}EtPO_3)_2$ (11)** Cu_{12}-ring	0.05–0.408 avg: 0.176
**$Cu_{29}EtPO_3$ (5)** Cu_9-ring	0.067–0.760 avg: 0.431	**$(Cu_{27}EtPO_3)_2$ (11)** Cu_9-ring	0.074–0.599 avg: 0.324
**$Cu_{29}EtPO_3$ (5)** Cu_7-ring	0.013–0.386 avg: 0.256	**$(Cu_{27}EtPO_3)_2$ (11)** Cu_6-ring	0.001–0.099 avg: 0.055
Avg. **$Cu_{29}EtPO_3$ (5)**	*0.445*	Avg. **$(Cu_{27}EtPO_3)_2$ (11)**	*0.185*
**$Cu_{29}BuPO_3$ (6)** Cu_{13}-ring	0.027–1.203 avg: 0.592	**$(Cu_{27}PrPO_3)_2$ (12)** Cu_{12}-ring	0.015–0.367 avg: 0.252<br>0.055–0.429 avg: 0.256
**$Cu_{29}BuPO_3$ (6)** Cu_9-ring	0.236–0.682 avg: 0.475	**$(Cu_{27}PrPO_3)_2$ (12)** Cu_9-ring	0.024–0.745 avg: 0.412<br>0.093–0.615 avg: 0.388
**$Cu_{29}BuPO_3$ (6)** Cu_7-ring	0.029–0.443 avg: 0.251	**$(Cu_{27}PrPO_3)_2$ (12)** Cu_6-ring	0.004–0.033 avg: 0.019<br>0.002–0.047 avg: 0.026
Avg. **$Cu_{29}BuPO_3$ (6)**	*0.439*	Avg. **$(Cu_{27}PrPO_3)_2$ (12)**	*0.228*<br>*0.223*

Table S17. Comparison of the P⋯Cu distances in different Cu_x rings in **1–12**.

	**P⋯Cu distance (Å)**		**P⋯Cu distance (Å)**
**$Cu_{27}MePO_3$ (1)** Cu_{12}-ring	6.5847(14)–6.9179(12)<br>avg: 6.751(2)	**$Cu_{31}{}^{n}C_{12}PO_3$ (7)** Cu_{14}-ring	6.886(3)–7.690(4) avg: 7.192(3)<br>6.920(2)–7.602(4) avg: 7.200(3)
**$Cu_{27}MePO_3$ (1)** Cu_9-ring	4.7740(12)–4.9001(12)<br>avg: 4.848(2)	**$Cu_{31}{}^{n}C_{12}PO_3$ (7)** Cu_9-ring	4.769(2)–5.233(2) avg: 4.996(2)<br>4.735(2)–5.268(2) avg: 4.999(2)
**$Cu_{27}MePO_3$ (1)** Cu_6-ring	5.0145(12)–5.0806(11)<br>avg: 5.041(2)	**$Cu_{31}{}^{n}C_{12}PO_3$ (7)** Cu_8-ring	5.140(3)–5.627(3) avg: 5.323(3)<br>5.148(3)–5.589(3) avg: 5.310(3)
Avg. **$Cu_{27}MePO_3$ (1)**	*5.546(2)*	Avg. **$Cu_{31}{}^{n}C_{12}PO_3$ (7)**	*5.837(3)*<br>*5.836(3)*
**$Cu_{29}MePO_3$ (2)** Cu_{13}-ring	6.679(2)–7.170(2) avg: 6.922(2)<br>6.697(3)–7.206(2) avg: 6.937(2)	**$Cu_{31}BnPO_3$ (8)** Cu_{14}-ring	6.9225(17)–7.620(2)<br>avg: 7.200(2)
**$Cu_{29}MePO_3$ (2)** Cu_9-ring	4.7977(18)–5.2189(19) avg: 4.979(2)<br>4.826(2)–5.2303(19) avg: 4.975(2)	**$Cu_{31}BnPO_3$ (8)** Cu_9-ring	4.7686(17)–5.266(5)<br>avg: 5.017(5)
**$Cu_{29}MePO_3$ (2)** Cu_7-ring	4.9251(17)–5.3549(18) avg: 5.114(2)<br>4.958(2)–5.3128(19) avg: 5.106(2)	**$Cu_{31}BnPO_3$ (8)** Cu_8-ring	5.1449(18)–5.5686(16)<br>avg: 5.316(2)
Avg. **$Cu_{29}MePO_3$ (2)**	*5.672(2)*<br>*5.673(2)*	Avg. **$Cu_{31}BnPO_3$ (8)**	*5.844(2)*
**$Cu_{31}MePO_3$ (3)** Cu_{14}-ring	6.867(3)–7.472(3)<br>avg: 7.151(3)	**$Cu_{28}PhPO_3$ (9)** Cu_{12}-ring	6.746(2)–7.1444(17)<br>avg: 6.880(2)
**$Cu_{31}MePO_3$ (3)** Cu_9-ring	4.825(3)–5.245(2)<br>avg: 5.027(3)	**$Cu_{28}PhPO_3$ (9)** Cu_{10}-ring	5.101(2)–5.3274(17)<br>avg: 5.202(2)
**$Cu_{31}MePO_3$ (3)** Cu_8-ring	5.095(3)–5.443(3)<br>avg: 5.249(3)	**$Cu_{28}PhPO_3$ (9)** Cu_6-ring	5.0610(16)–5.1799(18)<br>avg: 5.105(2)
Avg. **$Cu_{31}MePO_3$ (3)**	*5.809(3)*	Avg. **$Cu_{28}PhPO_3$ (9)**	*5.729(2)*
**$Cu_{31}EtPO_3$ (4)** Cu_{14}-ring	6.909(4)–7.604(3)<br>avg: 7.194(3)	**$Cu_{27}PhPO_3$ (9)** Cu_{12}-ring	6.746(2)–7.1444(17)<br>avg: 6.880(2)
**$Cu_{31}EtPO_3$ (4)** Cu_9-ring	4.809(3)–5.232(3)<br>avg: 5.035(3)	**$Cu_{27}PhPO_3$ (9)** Cu_9-ring	4.587(8)–5.196(2)<br>avg: 4.867(8)
**$Cu_{31}EtPO_3$ (4)** Cu_8-ring	5.106(2)–5.571(4)<br>avg: 5.296(3)	**$Cu_{27}PhPO_3$ (9)** Cu_6-ring	5.0610(16)–5.1799(18)<br>avg: 5.105(2)
Avg. **$Cu_{31}EtPO_3$ (4)**	*5.842(3)*	Avg. **$Cu_{27}PhPO_3$ (9)**	*5.617(2)*
**$Cu_{31}EtPO_3$ (5)** Cu_{14}-ring	6.893(10)–7.539(8)<br>avg: 7.186(10)	**$Cu_{31}PhPO_3$ (10)** Cu_{14}-ring	6.9513(9)–7.6405(9)<br>avg: 7.206(1)
**$Cu_{31}EtPO_3$ (5)** Cu_9-ring	4.736(9)–5.283(11)<br>avg: 5.040(10)	**$Cu_{31}PhPO_3$ (10)** Cu_9-ring	4.7138(10)–5.2559(10)<br>avg: 4.983(1)
**$Cu_{31}EtPO_3$ (5)** Cu_8-ring	5.154(8)–5.520(10)<br>avg: 5.278(10)	**$Cu_{31}PhPO_3$ (10)** Cu_8-ring	5.1768(8)–5.6288(9)<br>avg: 5.330(1)
Avg. **$Cu_{31}EtPO_3$ (5)**	*5.835(10)*	Avg. **$Cu_{31}PhPO_3$ (10)**	*5.840(1)*
**$Cu_{29}EtPO_3$ (5)** Cu_{13}-ring	6.69(5)–7.44(3)<br>avg: 7.00(4)	**$(Cu_{27}EtPO_3)_2$ (11)** Cu_{12}-ring	6.6383(17)–6.9591(12)<br>avg: 6.818(2)
**$Cu_{29}EtPO_3$ (5)** Cu_9-ring	4.73(4)–5.17(3)<br>avg: 4.92(4)	**$(Cu_{27}EtPO_3)_2$ (11)** Cu_9-ring	4.7132(11)–5.0823(14)<br>avg: 5.202(2)
**$Cu_{29}EtPO_3$ (5)** Cu_7-ring	4.81(4)–5.44(3)<br>avg: 5.18(4)	**$(Cu_{27}EtPO_3)_2$ (11)** Cu_6-ring	4.9991(18)–5.1525(9)<br>avg: 5.065(2)
Avg. **$Cu_{29}EtPO_3$ (5)**	*5.70(4)*	Avg. **$(Cu_{27}EtPO_3)_2$ (11)**	*5.598(2)*
**$Cu_{29}BuPO_3$ (6)** Cu_{13}-ring	6.675(4)–7.355(4)<br>avg: 6.971(4)	**$(Cu_{27}PrPO_3)_2$ (12)** Cu_{12}-ring	6.5888(12)–6.9045(15) avg: 6.780(2)<br>6.5885(13)–6.9702(15) avg: 6.764(2)
**$Cu_{29}BuPO_3$ (6)** Cu_9-ring	4.822(4)–5.194(4)<br>avg: 4.949(4)	**$(Cu_{27}PrPO_3)_2$ (12)** Cu_9-ring	4.6527(14)–4.9694(11) avg: 4.862(2)<br>4.7342(11)–5.0579(11) avg: 4.883(2)
**$Cu_{29}BuPO_3$ (6)** Cu_7-ring	5.006(5)–5.256(5)<br>avg: 5.143(5)	**$(Cu_{27}PrPO_3)_2$ (12)** Cu_6-ring	5.0120(14)–5.1062(15) avg: 5.071(2)<br>5.0058(11)–5.1263(15) avg: 5.064(2)
Avg. **$Cu_{29}BuPO_3$ (6)**	*5.688(4)*	Avg. **$(Cu_{27}PrPO_3)_2$ (12)**	*5.571(2)*<br>*5.570(2)*

Table S18. Selected bond lengths for **1** (Cu1–Cu9: Cu$_9$-ring; Cu10–Cu21: Cu$_{12}$-ring; Cu22–Cu27: Cu$_6$-ring).

P1– O1 1.520(2)	Cu7–O7 1.906(3)	Cu13–N24 1.962(4)	Cu21–O21 1.928(3)
P1–O2 1.525(3)	Cu7–O6 1.912(2)	Cu14–O13 1.912(3)	Cu21–N40 1.959(3)
P1–O3 1.509(3)	Cu7–N13 1.954(3)	Cu14–O14 1.926(2)	Cu21–N41 1.961(3)
P1–C1 1.801(4)	Cu7–N12 1.959(3)	Cu14–N27 1.944(4)	Cu22–O22 1.916(2)
Cu1–O1 1.898(4)	Cu8–O7 1.895(3)	Cu14–N26 1.973(3)	Cu22–O27 1.939(2)
Cu1–O9 1.915(3)	Cu8–O8 1.948(2)	Cu15–O15 1.928(2)	Cu22–N54 1.992(3)
Cu1–N18 1.971(4)	Cu8–N15 1.991(3)	Cu15–O14 1.931(3)	Cu22–N43 1.992(3)
Cu1–N1 1.980(7)	Cu8–N14 2.011(3)	Cu15–N29 1.965(4)	Cu23–O22 1.923(2)
Cu1–N1B 2.033(12)	Cu9–O9 1.915(3)	Cu15–N28 1.971(3)	Cu23–O23 1.948(3)
Cu2–N2 1.828(7)	Cu9–O8 1.947(2)	Cu16–O15 1.927(3)	Cu23–N45 2.003(4)
Cu2–O1 1.909(3)	Cu9–N16 2.023(3)	Cu16–O16 1.931(2)	Cu23–N44 2.021(3)
Cu2–O2 1.937(3)	Cu9–N17 2.028(3)	Cu16–N31 1.965(3)	Cu23–O11 2.352(3)
Cu2–N3 2 1.984(3)	Cu9–O20 2.373(2)	Cu16–N30 1.977(3)	Cu24–O24 1.925(2)
Cu2–N2B 2.246(12)	Cu10–O10 1.915(3)	Cu17–O16 1.918(2)	Cu24–O23 1.930(3)
Cu3–O3 1.931(3)	Cu10–O21 1.932(2)	Cu17–O17 1.938(3)	Cu24–N47 1.987(4)
Cu3–O2 1.939(3)	Cu10–N19B 1.957(12)	Cu17–N33 1.960(3)	Cu24–N46 2.003(3)
Cu3–N5 1.978(3)	Cu10–N42 1.970(4)	Cu17–N32 1.974(3)	Cu25–O24 1.925(3)
Cu3–N4 1.987(4)	Cu10–N19 1.999(10)	Cu18–O18 1.921(3)	Cu25–O25 1.945(2)
Cu4–O4 1.904(2)	Cu11–N20B 1.756(10)	Cu18–O17 1.925(2)	Cu25–N49 1.992(3)
Cu4–O3 1.909(3)	Cu11–O11 1.932(3)	Cu18–N35 1.964(3)	Cu25–N48 2.017(3)
Cu4–N6 1.959(3)	Cu11–O10 1.935(3)	Cu18–N34 1.978(3)	Cu25–O15 2.389(3)
Cu4–N7 1.965(3)	Cu11–N21 1.978(4)	Cu19–O18 1.926(2)	Cu26–O26 1.922(2)
Cu5–O4 1.908(3)	Cu11–N20 2.169(11)	Cu19–O19 1.931(2)	Cu26–O25 1.941(2)
Cu5–O5 1.941(2)	Cu12–O12 1.922(3)	Cu19–N37 1.969(3)	Cu26–N51 1.990(3)
Cu5–N9 1.979(3)	Cu12–O11 1.925(3)	Cu19–N36 1.973(3)	Cu26–N50 1.997(3)
Cu5–N8 1.989(3)	Cu12–N22 1.952(4)	Cu20–O20 1.928(2)	Cu27–O26 1.933(2)
Cu6–O6 1.917(3)	Cu12–N23 1.969(4)	Cu20–O19 1.929(2)	Cu27–O27 1.950(2)
Cu6–O5 1.942(2)	Cu13–O12 1.925(3)	Cu20–N38 1.960(3)	Cu27–N52 1.991(3)
Cu6–N11 1.976(3)	Cu13–O13 1.931(3)	Cu20–N39 1.979(3)	Cu27–N53 2.026(3)
Cu6–N10 1.986(3)	Cu13–N25 1.961(4)	Cu21–O20 1.923(2)	Cu27–O19 2.387(2)

Table S19. Selected bond lengths for UNIT 1 of **2** (Cu1_3–Cu7_3: Cu_7-ring; Cu8_3–Cu20_3: Cu_{13}-ring; Cu21_3–Cu29_3: Cu_9-ring; O1_1–O3_1: $MePO_3^{2-}$ anion).

P1_1–O2_1 1.518(4)	Cu7_3–O6_3 1.934(4)	Cu15_3–O14_3 1.910(4)	Cu22_3–N42_3 1.993(6)
P1_1–O1_1 1.526(4)	Cu7_3–N13_3 1.995(5)	Cu15_3–O15_3 1.919(4)	Cu23_3–O22_3 1.907(5)
P1_1–O3_1 1.530(4)	Cu7_3–N12_3 2.009(5)	Cu15_3–N29_3 1.961(5)	Cu23_3–O23_3 1.926(4)
P1_1–C1_1 1.799(6)	Cu8_3–O20_3 1.923(4)	Cu15_3–N28_3 1.965(6)	Cu23_3–N44_3 1.983(6)
Cu1_3–O7_3 1.942(4)	Cu8_3–O8_3 1.925(5)	Cu16_3–O15_3 1.921(4)	Cu23_3–N45_3 2.009(6)
Cu1_3–O1_3 1.953(4)	Cu8_3–N40_3 1.949(6)	Cu16_3–O16_3 1.930(4)	Cu24_3–O23_3 1.919(4)
Cu1_3–N1_3 2.003(5)	Cu8_3–N15_3 1.971(6)	Cu16_3–N31_3 1.965(6)	Cu24_3–O24_3 1.922(4)
Cu1_3–N14_3 2.024(5)	Cu9_3–O9_3 1.893(5)	Cu16_3–N30_3 1.972(5)	Cu24_3–N47_3 1.981(5)
Cu1_3–O20_3 2.412(5)	Cu9_3–O8_3 1.920(5)	Cu17_3–O16_3 1.910(5)	Cu24_3–N46_3 2.004(5)
Cu2_3–O2_3 1.908(4)	Cu9_3–N17_3 1.953(7)	Cu17_3–O17_3 1.917(5)	Cu25_3–O24_3 1.910(4)
Cu2_3–O1_3 1.915(4)	Cu9_3–N16_3 1.956(7)	Cu17_3–N33_3 1.969(7)	Cu25_3–O25_3 1.923(4)
Cu2_3–N2_3 1.961(5)	Cu10_3–O10_3 1.919(5)	Cu17_3–N32_3 1.970(6)	Cu25_3–N49_3 1.993(5)
Cu2_3–N3_3 1.986(6)	Cu10_3–O9_3 1.942(5)	Cu18_3–O17_3 1.919(5)	Cu25_3–N48_3 2.003(5)
Cu3_3–O3_3 1.919(4)	Cu10_3–N19_3 1.964(6)	Cu18_3–O18_3 1.924(4)	Cu26_3–O26_3 1.926(4)
Cu3_3–O2_3 1.970(5)	Cu10_3–N18_3 1.968(7)	Cu18_3–N34_3 1.964(6)	Cu26_3–O25_3 1.968(4)
Cu3_3–N5_3 1.970(6)	Cu11_3–O11_3 1.910(4)	Cu18_3–N35_3 1.966(5)	Cu26_3–N50_3 1.996(5)
Cu3_3–N4_3 2.001(5)	Cu11_3–O10_3 1.933(5)	Cu19_3–O19_3 1.914(4)	Cu26_3–N51_3 2.011(6)
Cu3_3–O11_3 2.346(4)	Cu11_3–N20_3 1.963(6)	Cu19_3–O18_3 1.930(4)	Cu26_3–O15_3 2.397(4)
Cu4_3–O3_3 1.901(5)	Cu11_3–N21_3 1.976(6)	Cu19_3–N36_3 1.957(6)	Cu27_3–O26_3 1.905(5)
Cu4_3–O4_3 1.908(5)	Cu12_3–O12_3 1.936(5)	Cu19_3–N37_3 1.971(5)	Cu27_3–O27_3 1.908(4)
Cu4_3–N7_3 1.948(6)	Cu12_3–O11_3 1.939(5)	Cu20_3–O20_3 1.908(4)	Cu27_3–N52_3 1.957(5)
Cu4_3–N6_3 1.973(6)	Cu12_3–N23_3 1.969(6)	Cu20_3–O19_3 1.920(4)	Cu27_3–N53_3 1.962(5)
Cu5_3–O4_3 1.936(4)	Cu12_3–N22_3 1.981(6)	Cu20_3–N39_3 1.957(6)	Cu28_3–O27_3 1.897(5)
Cu5_3–O5_3 1.940(4)	Cu13_3–O12_3 1.918(5)	Cu20_3–N38_3 1.976(5)	Cu28_3–O28_3 1.918(4)
Cu5_3–N9_3 1.993(5)	Cu13_3–O13_3 1.929(4)	Cu21_3–O21_3 1.897(5)	Cu28_3–N54_3 1.959(5)
Cu5_3–N8_3 2.006(6)	Cu13_3–N24_3 1.960(6)	Cu21_3–O29_3 1.915(4)	Cu28_3–N55_3 1.975(5)
Cu5_3–O14_3 2.367(4)	Cu13_3–N25_3 1.991(7)	Cu21_3–N41_3 1.985(6)	Cu29_3–O29_3 1.932(5)
Cu6_3–O5_3 1.929(4)	Cu14_3–O13_3 1.913(4)	Cu21_3–N58_3 1.988(6)	Cu29_3–O28_3 1.933(4)
Cu6_3–O6_3 1.953(4)	Cu14_3–O14_3 1.928(4)	Cu22_3–O21_3 1.884(5)	Cu29_3–N56_3 2.004(6)
Cu6_3–N11_3 1.977(5)	Cu14_3–N27_3 1.954(6)	Cu22_3–O22_3 1.892(5)	Cu29_3–N57_3 2.008(5)
Cu6_3–N10_3 2.006(5)	Cu14_3–N26_3 1.965(6)	Cu22_3–N43_3 1.981(6)	Cu29_3–O19_3 2.380(4)
Cu7_3–O7_3 1.931(4)			

Table S20. Selected bond lengths for UNIT 2 of **2** (Cu1_4–Cu7_4: Cu$_7$-ring; Cu8_4–Cu20_4: Cu$_{13}$-ring; Cu21_4–Cu29_4: Cu$_9$-ring; O1_2–O3_2: $MePO_3^{2-}$ anion).

P1_2–O2_2 1.519(5)	Cu7_4–O6_4 1.943(4)	Cu15_4–O15_4 1.907(4)	Cu22_4–N43_4 1.992(5)
P1_2–O1_2 1.524(4)	Cu7_4–N12_4 2.003(5)	Cu15_4–O14_4 1.910(4)	Cu23_4–O22_4 1.888(5)
P1_2–O3_2 1.533(5)	Cu7_4–N13_4 2.007(5)	Cu15_4–N28_4 1.957(5)	Cu23_4–O23_4 1.935(4)
P1_2–C1_2 1.798(6)	Cu8_4–O20_4 1.915(4)	Cu15_4–N29_4 1.960(5)	Cu23_4–N44_4 1.986(5)
Cu1_4–O7_4 1.942(4)	Cu8_4–O8_4 1.925(4)	Cu16_4–O15_4 1.915(4)	Cu23_4–N45_4 2.006(5)
Cu1_4–O1_4 1.953(4)	Cu8_4–N15_4 1.960(5)	Cu16_4–O16_4 1.931(4)	Cu24_4–O23_4 1.915(4)
Cu1_4–N1_4 1.995(6)	Cu8_4–N40_4 1.964(6)	Cu16_4–N31_4 1.950(6)	Cu24_4–O24_4 1.916(4)
Cu1_4–N14_4 2.038(5)	Cu9_4–O9_4 1.915(5)	Cu16_4–N30_4 1.983(6)	Cu24_4–N47_4 1.981(5)
Cu1_4–O20_4 2.404(4)	Cu9_4–O8_4 1.915(4)	Cu17_4–O16_4 1.915(5)	Cu24_4–N46_4 1.997(5)
Cu2_4–O2_4 1.911(4)	Cu9_4–N17_4 1.954(6)	Cu17_4–O17_4 1.926(5)	Cu25_4–O24_4 1.918(4)
Cu2_4–O1_4 1.918(4)	Cu9_4–N16_4 1.967(5)	Cu17_4–N33_4 1.949(7)	Cu25_4–O25_4 1.933(4)
Cu2_4–N2_4 1.974(6)	Cu10_4–O9_4 1.923(5)	Cu17_4–N32_4 1.968(6)	Cu25_4–N48_4 1.997(5)
Cu2_4–N3_4 1.991(5)	Cu10_4–O10_4 1.935(4)	Cu18_4–O17_4 1.910(5)	Cu25_4–N49_4 2.002(5)
Cu3_4 O3_4 1.909(5)	Cu10_4–N19_4 1.984(5)	Cu18_4–O18_4 1.929(5)	Cu26_4–O26_4 1.929(4)
Cu3_4–O2_4 1.965(4)	Cu10_4–N18_4 1.986(5)	Cu18_4–N34_4 1.953(6)	Cu26_4–O25_4 1.945(4)
Cu3_4–N5_4 1.975(6)	Cu11_4–O10_4 1.922(4)	Cu18_4–N35_4 1.980(6)	Cu26_4–N50_4 1.977(5)
Cu3_4–N4_4 1.978(5)	Cu11_4–O11_4 1.926(4)	Cu19_4–O19_4 1.916(4)	Cu26_4–N51_4 2.018(6)
Cu3_4–O11_4 2.364(4)	Cu11_4–N20_4 1.955(5)	Cu19_4–O18_4 1.927(4)	Cu26_4–O15_4 2.429(4)
Cu4_4–O3_4 1.893(4)	Cu11_4–N21_4 1.976(6)	Cu19_4–N36_4 1.943(6)	Cu27_4–O27_4 1.907(5)
Cu4_4–O4_4 1.916(5)	Cu12_4–O11_4 1.916(4)	Cu19_4–N37_4 1.980(5)	Cu27_4–O26_4 1.916(5)
Cu4_4–N6_4 1.935(6)	Cu12_4–O12_4 1.948(4)	Cu20_4–O19_4 1.922(4)	Cu27_4–N52_4 1.951(6)
Cu4_4–N7_4 1.955(5)	Cu12_4–N23_4 1.970(5)	Cu20_4–O20_4 1.922(4)	Cu27_4–N53_4 1.967(6)
Cu5_4–O4_4 1.934(4)	Cu12_4–N22_4 1.976(6)	Cu20_4–N39_4 1.954(5)	Cu28_4–O27_4 1.896(5)
Cu5_4–O5_4 1.935(4)	Cu13_4–O12_4 1.916(4)	Cu20_4–N38_4 1.955(6)	Cu28_4–O28_4 1.905(4)
Cu5_4–N9_4 1.961(5)	Cu13_4–O13_4 1.924(4)	Cu21_4–O21_4 1.915(5)	Cu28_4–N55_4 1.958(5)
Cu5_4–N8_4 1.990(5)	Cu13_4–N24_4 1.985(6)	Cu21_4–O29_4 1.921(4)	Cu28_4–N54_4 1.959(6)
Cu6_4–O5_4 1.924(4)	Cu13_4–N25_4 1.989(5)	Cu21_4–N41_4 1.980(5)	Cu29_4–O29_4 1.931(4)
Cu6_4–O6_4 1.961(4)	Cu14_4–O13_4 1.916(4)	Cu21_4–N58_4 2.004(6)	Cu29_4–O28_4 1.939(4)
Cu6_4–N11_4 1.987(5)	Cu14_4–O14_4 1.931(4)	Cu22_4–O21_4 1.887(5)	Cu29_4–N56_4 2.008(6)
Cu6_4–N10_4 2.007(5)	Cu14_4–N27_4 1.964(5)	Cu22_4–O22_4 1.902(5)	Cu29_4–N57_4 2.015(5)
Cu6_4–O16_4 2.427(5)	Cu14_4–N26_4 1.966(5)	Cu22_4–N42_4 1.969(5)	Cu29_4–O19_4 2.389(4)
Cu7_4–O7_4 1.920(4)			

Table S21. Selected bond lengths for **3** (Cu1–Cu8: Cu_8-ring; Cu9–Cu22: Cu_{14}-ring; Cu23–Cu31: Cu_9-ring).

P1–O2 1.504(6)
P1–O3 1.519(6)
P1–O1 1.534(6)
P1–C1 1.791(9)
Cu1–O8 1.896(6)
Cu1–O1 1.928(6)
Cu1–N16 1.985(8)
Cu1–N1 1.998(7)
Cu2–O1 1.922(5)
Cu2–O2 1.944(6)
Cu2–N3 1.994(7)
Cu2–N2 2.003(7)
Cu2–O10 2.402(6)
Cu3–O3 1.918(5)
Cu3–O2 1.954(5)
Cu3–N4 1.975(6)
Cu3–N5 2.004(6)
Cu4–O4 1.923(5)
Cu4–O3 1.931(5)
Cu4–N7 1.977(7)
Cu4–N6 2.006(6)
Cu5–O4 1.886(5)
Cu5–O5 1.928(5)
Cu5–N8 1.977(6)
Cu5–N9 1.986(6)
Cu6–O6 1.915(6)
Cu6_–O5 1.955(5)
Cu6–N10 2.000(7)
Cu6–N11 2.000(7)
Cu7–O6 1.928(6)
Cu7–O7 1.960(6)
Cu7–N13 1.977(8)
Cu7–N12 2.003(7)
Cu8–O8 1.893(6)
Cu8–O7 1.922(6)
Cu8–N14 1.955(8)
Cu8–N15 1.965(8)
Cu9–O22 1.928(6)
Cu9–O9 1.935(6)
Cu9–N17 1.961(7)
Cu9–N44 1.962(8)
Cu10–O9 1.912(6)
Cu10–O10 1.933(6)
Cu10–N18 1.959(7)
Cu10–N19 1.967(7)
Cu11–O10 1.920(6)
Cu11–O11 1.934(6)
Cu11–N21 1.961(8)
Cu11–N20 1.969(7)
Cu12–O12 1.918(5)
Cu12–O11 1.925(6)
Cu12–N22 1.969(7)
Cu12–N23 1.972(7)
Cu13–O13 1.916(5)
Cu13–O12 1.942(6)
Cu13–N24 1.948(7)
Cu13–N25 1.977(7)
Cu14–O14 1.909(5)
Cu14–O13 1.922(5)
Cu14–N26 1.958(7)
Cu14–N27 1.961(6)
Cu15–O14 1.903(5)
Cu15–O15 1.930(5)
Cu15–N29 1.973(7)
Cu15–N28 1.979(7)
Cu16–O15 1.907(6)
Cu16–O16 1.917(6)
Cu16–N30 1.963(7)
Cu16–N31 1.964(8)
Cu17–O17 1.905(6)
Cu17–O16 1.928(6)
Cu17–N32 1.939(8)
Cu17–N33 1.977(7)
Cu18–O18 1.903(6)
Cu18–O17 1.921(5)
Cu18–N34 1.940(8)
Cu18–N35 1.954(7)
Cu19–O19 1.912(6)
Cu19–O18 1.921(6)
Cu19–N37 1.939(8)
Cu19–N36 1.954(7)
Cu20–O20 1.916(6)
Cu20–O19 1.923(6)
Cu20–N38 1.946(8)
Cu20–N39 1.962(8)
Cu21–N41B 1.87(3)
Cu21–O21 1.905(6)
Cu21–O20 1.915(6)
Cu21–N40 1.955(8)
Cu21–N41 1.996(10)
Cu22–O22 1.893(6)
Cu22–O21 1.923(7)
Cu22–N43 1.960(9)
Cu22–N42 1.965(9)
Cu22–N42B 2.06(3)
Cu23–O31 1.937(6)
Cu23–O23 1.945(6)
Cu23–N62 1.979(8)
Cu23–N45 1.981(8)
Cu23–O9 2.383(6)
Cu24–O24 1.911(6)
Cu24–O23 1.911(7)
Cu24–N46 1.954(7)
Cu24–N47 1.970(9)
Cu25–O25 1.907(6)
Cu25–O24 1.912(6)
Cu25–N49 1.961(8)
Cu25–N48 1.977(7)
Cu26–O25 1.934(6)
Cu26–O26 1.949(6)
Cu26–N51 1.993(8)
Cu26–N50 2.014(8)
Cu26–O13 2.361(5)
Cu27–O27 1.846(6)
Cu27–O26 1.920(6)
Cu27–N53 1.951(8)
Cu27–N52 1.963(8)
Cu28–O28 1.920(6)
Cu28–O27 1.933(5)
Cu28–N55 1.970(7)
Cu28–N54 1.977(8)
Cu29–O29 1.933(6)
Cu29–O28 1.943(5)
Cu29–N57 2.009(7)
Cu29–N56 2.015(7)
Cu30–O30 1.912(6)
Cu30–O29 1.923(5)
Cu30–N59 1.981(8)
Cu30–N58 1.987(7)
Cu31–O30 1.898(6)
Cu31–O31 1.922(6)
Cu31–N60 1.967(7)
Cu31–N61 1.967(7)

Table S22. Selected bond lengths for **4** (Cu1–Cu8: Cu$_8$-ring; Cu9–Cu22: Cu$_{14}$-ring; Cu23–Cu31: Cu$_9$-ring).

P1_1 O1_1 1.518(7)	Cu7–N13 1.960(7)	Cu16–O17 1.900(7)	Cu24–O24 1.918(6)
P1_1 O2_1 1.503(6)	Cu7–N12 1.971(7)	Cu16–O16 1.936(6)	Cu24–N49 1.961(7)
P1_1 O3_1 1.496(8)	Cu8–O7 1.906(6)	Cu16–N33 1.965(8)	Cu24–N48 1.972(7)
P1_1 C1_1 1.783(16)	Cu8–O8 1.922(5)	Cu16–N32 1.967(9)	Cu25–O24 1.942(6)
C1_1 C2_1 1.53(2)	Cu8–N14 1.973(7)	Cu17–O17 1.907(6)	Cu25–O25 1.945(5)
Cu1–O8 1.930(5)	Cu8–N15 1.985(8)	Cu17–O18 1.922(6)	Cu25–N50 1.991(7)
Cu1–O1 1.959(6)	Cu9–O9 1.915(6)	Cu17–N35 1.956(8)	Cu25–N51 1.991(7)
Cu1–N1 1.995(6)	Cu9–O10 1.921(6)	Cu17–N34 1.960(8)	Cu25–O12 2.394(6)
Cu1–N16 2.000(7)	Cu9–N46 1.955(9)	Cu18–O18 1.905(7)	Cu26–O26 1.908(6)
Cu1–O9 2.436(6)	Cu9–N19 1.955(7)	Cu18–O19 1.914(6)	Cu26–O25 1.923(5)
Cu2–O2 1.931(5)	Cu10–O10 1.920(5)	Cu18–N36 1.964(8)	Cu26–N52 1.969(6)
Cu2–O1 1.948(6)	Cu10–O11 1.923(5)	Cu18–N37 1.965(9)	Cu26–N53 1.974(7)
Cu2–N2 1.981(7)	Cu10–N21 1.951(6)	Cu19–O19 1.907(7)	Cu27–O26 1.905(6)
Cu2–N3 1.997(7)	Cu10–N20 1.955(7)	Cu19–O20 1.915(6)	Cu27–O27 1.923(6)
Cu3–O3 1.910(5)	Cu11–O12 1.910(5)	Cu19–N39 1.945(10)	Cu27–N54 1.985(7)
Cu3–O2 1.942(6)	Cu11–O11 1.939(5)	Cu19–N38 1.969(8)	Cu27–N55 1.994(7)
Cu3–N5 1.985(7)	Cu11–N23 1.960(7)	Cu20–O21 1.895(6)	Cu28–O28 1.936(6)
Cu3–N4 1.990(7)	Cu11–N22 1.960(7)	Cu20–O20 1.919(7)	Cu28–O27 1.939(6)
Cu3–O13 2.432(6)	Cu12–O12 1.918(5)	Cu20–N40 1.951(8)	Cu28–N56 2.001(8)
Cu4–O3 1.893(6)	Cu12–O13 1.926(5)	Cu20–N41 1.961(9)	Cu28–N57 2.010(7)
Cu4–O4 1.918(6)	Cu12–N24 1.944(7)	Cu21–O22 1.918(6)	Cu29–O29 1.914(6)
Cu4–N6 1.954(7)	Cu12–N25 1.967(7)	Cu21–O21 1.921(6)	Cu29–O28 1.926(6)
Cu4–N7 1.991(7)	Cu13–O13 1.897(6)	Cu21–N43 1.969(8)	Cu29–N59 1.975(8)
Cu5–O5 1.910(6)	Cu13–O14 1.916(6)	Cu21–N42 1.974(8)	Cu29–N58 1.985(8)
Cu5–O4 1.967(6)	Cu13–N27 1.932(8)	Cu22–O22 1.914(6)	Cu30–O29 1.902(7)
Cu5–N9 1.998(7)	Cu13–N26 1.965(8)	Cu22–O9 1.930(6)	Cu30–O30 1.914(6)
Cu5–N8 2.008(7)	Cu14–O15 1.897(6)	Cu22–N45 1.948(8)	Cu30–N61 1.967(8)
Cu5–O16 2.398(6)	Cu14–O14 1.931(6)	Cu22–N44 1.970(8)	Cu30–N60 2.002(8)
Cu6–O5 1.917(6)	Cu14–N28 1.965(8)	Cu23–O23 1.894(6)	Cu31–O31 1.917(6)
Cu6–O6 1.959(6)	Cu14–N29 1.971(8)	Cu23–O31 1.923(6)	Cu31–O30 1.949(6)
Cu6–N10 1.971(8)	Cu15–O16 1.929(7)	Cu23–N47 1.961(7)	Cu31–N63 2.004(8)
Cu6–N11 1.985(7)	Cu15–O15 1.930(6)	Cu23–N64 1.969(8)	Cu31–N62 2.009(7)
Cu7–O7 1.887(6)	Cu15–N30 1.953(8)	Cu24–O23 1.896(6)	Cu31–O22 2.392(7)
Cu7–O6 1.907(6)	Cu15–N31 1.967(8)		

Table S23. Selected bond lengths for the major unit (Cu_{9+14+8}, 95%) of **5** (Cu1–Cu9: Cu_9-ring; Cu10–Cu23: Cu_{14}-ring; Cu24–Cu31: Cu_8-ring; O1_2–O3_2, O1C_2–O3C_2: $EtPO_3^{2-}$ anions with occupancy ratio 0.62/0.33).

P1_2–O1_2 1.540(8)
P1_2–O2_2 1.522(8)
P1_2–O3_2 1.521(8)
P1_2–C1_2 1.837(10)
C1_2–C2_2 1.524(11)
P1C_2–O1C_2 1.545(13)
P1C_2–O2C_2 1.523(14)
P1C_2–O3C_2 1.514(14)
P1C_2–C1C_2 1.828(15)
C1C_2–C2C_2 1.530(18)
Cu1–O9_1 1.907(4)
Cu1–O1_1 1.926(4)
Cu1–N1_1 1.963(5)
Cu1–N18 1.982(5)
Cu2–O1 1.934(4)
Cu2–O2 1.940(4)
Cu2–N3 2.005(5)
Cu2–N2 2.007(5)
Cu2–O11 2.379(4)
Cu3–O3 1.901(4)
Cu3–O2 1.909(4)
Cu3–N5 1.970(5)
Cu3–N4 1.987(5)
Cu4–O3 1.909(4)
Cu4–O4 1.932(4)
Cu4–N6 1.988(5)
Cu4–N7 1.989(5)
Cu5–O5 1.933(4)
Cu5–O4 1.940(4)
Cu5–N9 2.017(5)
Cu5–N8 2.017(5)
Cu6–O6 1.908(4)
Cu6–O5 1.935(4)
Cu6–N10 1.999(5)
Cu6–N11 2.012(4)
Cu7–O6 1.887(4)
Cu7–O7 1.911(4)
Cu7–N12 1.956(5)
Cu7–N13 1.969(5)
Cu8–O8 1.942(4)
Cu8–O7 1.951(3)
Cu8–N15 1.993(5)
Cu8–N14 2.001(5)
Cu8–O21 2.365(4)
Cu9–O9 1.901(4)
Cu9–O8 1.911(4)
Cu9–N16 1.953(5)
Cu9–N17 1.966(6)
Cu10–O10 1.913(4)
Cu10–O23 1.935(4)
Cu10–N46 1.963(5)
Cu10–N19 1.965(5)
Cu11–O11 1.922(4)
Cu11–O10 1.924(4)
Cu11–N21 1.958(5)
Cu11–N20 1.968(5)
Cu12–O11 1.921(4)
Cu12–O12 1.927(4)
Cu12–N23 1.964(5)
Cu12–N22 1.975(6)
Cu13–O12 1.908(4)
Cu13–O13 1.918(4)
Cu13–N24 1.942(5)
Cu13–N25 1.959(5)
Cu14–O13 1.921(4)
Cu14–O14 1.908(4)
Cu14–N26 1.974(5)
Cu14–N27 1.960(6)
Cu15–O14 1.903(4)
Cu15–O15 1.955(5)
Cu15–N28 1.964(6)
Cu15–N29 1.950(5)
Cu16–O15 1.906(5)
Cu16–O16 1.903(5)
Cu16–N30 1.958(5)
Cu16–N31 1.953(5)
Cu17–O16 1.940(5)
Cu17–O17 1.920(4)
Cu17–N32 1.957(5)
Cu17–N33 1.962(6)
Cu18–O17 1.906(4)
Cu18–O18 1.924(4)
Cu18–N34 1.970(5)
Cu18–N35 1.953(5)
Cu19–O18 1.919(5)
Cu19–O19 1.905(4)
Cu19–N36 1.952(6)
Cu19–N37 1.957(7)
Cu20–O19 1.935(5)
Cu20–O20 1.902(4)
Cu20–N39 1.969(6)
Cu20–N38 1.975(5)
Cu21–O21 1.916(4)
Cu21–O20 1.920(4)
Cu21–N41 1.957(4)
Cu21–N40 1.980(6)
Cu22–O21 1.917(4)
Cu22–O22 1.941(4)
Cu22–N43 1.952(5)
Cu22–N42 1.965(5)
Cu23–O22 1.926(4)
Cu23–O23 1.930(4)
Cu23–N44 1.969(5)
Cu23–N45 1.970(5)
Cu24–O24 1.929(4)
Cu24–O31 1.948(4)
Cu24–N47 2.017(5)
Cu24–N62 2.011(5)
Cu24–O10 2.413(4)
Cu25–O24.931(4)
Cu25–O25 1.899(4)
Cu25–N48 1.980(5)
Cu25–N49 1.973(5)
Cu26–O25 1.889(4)
Cu26–O26 1.911(4)
Cu26–N50 1.951(6)
Cu26–N51 1.956(5)
Cu27–O26 1.960(4)
Cu27–O27 1.921(4)
Cu27–N52 1.984(5)
Cu27–N53 2.005(6)
Cu28–O27 1.918(4)
Cu28–O28 1.946(4)
Cu28–N54 1.987(4)
Cu28–N55 2.003(5)
Cu29–O28 1.916(5)
Cu29–O29 1.884(4)
Cu29–N56 1.984(5)
Cu29–N57 1.950(6)
Cu30–O29 1.921(4)
Cu30–O30 1.940(4)
Cu30–N58 1.996(5)
Cu30–N59 2.001(5)
Cu31–O30 1.917(4)
Cu31–O31 1.954(4)
Cu31–N60 1.994(6)
Cu31–N61 1.960(4)

Table S24. Selected bond lengths for the minor unit (Cu$_{9+13+7}$, 5%) of **5** (Cu1–Cu9: Cu$_9$-ring; Cu10–Cu12, Cu20–Cu23, Cu53–Cu59: Cu$_{13}$-ring; Cu24, Cu30, Cu31, Cu65–Cu69: Cu$_7$-ring; O1B_2–O3B_2: EtPO$_3^{2-}$ anion with occupancy 0.05).

P1B_2–O3B_2 1.521(17)	Cu7–O7 1.911(4)	Cu54–N27B 1.959(17)	Cu23–N44 1.969(5)
P1B_2–O2B_2 1.527(17)	Cu7–N12 1.956(5)	Cu56–O14B 1.883(17)	Cu23–N45 1.970(5)
P1B_2–O1B_2 1.551(17)	Cu7–N13 1.969(5)	Cu56–O16B 1.956(18)	Cu24–O24B 2.016(15)
P1B_2–C1B_2 1.819(19)	Cu8–O8 1.942(4)	Cu56–N28B 1.953(17)	Cu24–O31 1.948(4)
C1B_2–C2B_2 1.52(2)	Cu8–O7 1.951(3)	Cu56–N31B 1.968(17)	Cu24–N47B 2.027(16)
Cu1–O9 1.907(4)	Cu8–N15 1.993(5)	Cu57–O16B 1.919(18)	Cu24–N62 2.011(5)
Cu1–O1 1.926(4)	Cu8–N14 2.001(5)	Cu57–O17B_1.886(18)	Cu24–O10 2.413(4)
Cu1–N1 1.963(5)	Cu8–O21 2.365(4)	Cu57–N32B 1.958(17)	Cu65–O24B 1.987(16)
Cu1–N18 1.982(5)	Cu9–O9 1.901(4)	Cu57–N33B 1.989(18)	Cu65–O25B 1.899(17)
Cu2–O1 1.934(4)	Cu9–O8 1.911(4)	Cu58–O17B 1.934(17)	Cu65–N48B 2.012(18)
Cu2–O2 1.940(4)	Cu9–N16 1.953(5)	Cu58–O18B 1.959(18)	Cu65–N49B 1.985(17)
Cu2–N3 2.005(5)	Cu9–N17 1.966(6)	Cu58–N34B 1.968(17)	Cu66–O25B 1.917(17)
Cu2–N2 2.007(5)	Cu10–O10 1.913(4)	Cu58–N35B 1.981(17)	Cu66–O26B 1.923(17)
Cu2–O11 2.379(4)	Cu10–O23 1.935(4)	Cu59–O18B 1.875(18)	Cu66–N50B 1.962(18)
Cu3–O3 1.901(4)	Cu10–N46 1.963(5)	Cu59–O19B 1.989(17)	Cu66–N51B 1.965(17)
Cu3–O2 1.909(4)	Cu10–N19 1.965(5)	Cu59–N36B 1.973(17)	Cu68–O26B 1.959(17)
Cu3–N5 1.970(5)	Cu11–O11 1.922(4)	Cu59–N37B 1.975(18)	Cu68–O28B_1.916(17)
Cu3–N4 1.987(5)	Cu11–O10 1.924(4)	Cu20–O19B 2.041(14)	Cu68–N52B 1.996(17)
Cu4–O3 1.909(4)	Cu11–N21 1.958(5)	Cu20–O20 1.902(4)	Cu68–N55B 2.022(18)
Cu4–O4 1.932(4)	Cu11–N20 1.968(5)	Cu20–N38B 2.025(16)	Cu69–O28B 1.937(19)
Cu4–N6 1.988(5)	Cu12–O11 1.921(4)	Cu20–N39 1.969(6)	Cu69–O29B 1.953(19)
Cu4–N7 1.989(5)	Cu12–O12 1.927(4)	Cu21–O21 1.916(4)	Cu69–N56B 2.002(18)
Cu5–O5 1.933(4)	Cu12–N22 1.975(6)	Cu21–O20 1.920(4)	Cu69–N57B 2.015(19)
Cu5–O4 1.940(4)	Cu12–N23 1.964(5)	Cu21–N41 1.957(4)	Cu30–O29B 1.941(16)
Cu5–N9 2.017(5)	Cu53–O12 2.140(19)	Cu21–N40 1.980(6)	Cu30–O30 1.940(4)
Cu5–N8 2.017(5)	Cu53–O13B_1 1.916(18)	Cu22–O21 1.917(4)	Cu30–N58B 1.993(16)
Cu6–O6 1.908(4)	Cu53–N24 2.458(18)	Cu22–O22 1.941(4)	Cu30–N59 2.001(5)
Cu6–O5 1.935(4)	Cu53–N25B_1 1.963(19)	Cu22–N43 1.952(5)	Cu31–O30 1.917(4)
Cu6–N10 1.999(5)	Cu54–O13B 1.932(17)	Cu22–N42 1.965(5)	Cu31–O31 1.954(4)
Cu6–N11 2.012(4)	Cu54–O14B 1.902(17)	Cu23–O22 1.926(4)	Cu31–N60 1.994(6)
Cu7–O6 1.887(4)	Cu54–N26B 1.975(17)	Cu23–O23 1.930(4)	Cu31–N61 1.960(4)

Table S25. Selected bond lengths for **6** (Cu1–Cu9: Cu$_9$-ring; Cu10–Cu22: Cu$_{13}$-ring; Cu23–Cu29: Cu$_7$-ring; O1_2–O3_2 and O1B_2–O3B_2: ^{n}BuPO$_3^{2-}$ anion disordered over two positions in an 82/18 ratio).

P1_2–O1_2 1.517(5)	Cu6–O5 1.914(3)	Cu14–N27 1.964(4)	Cu22–O22 1.911(3)
P1_2–O2_2 1.517(5)	Cu6–O6 1.941(4)	Cu14–N26 1.988(4)	Cu22–O21 1.927(3)
P1_2–O3_2 1.520(5)	Cu6–N11 1.993(4)	Cu15–O14 1.924(3)	Cu22–N42 1.962(4)
P1B_2–O2B_2 1.486(17)	Cu6–N10 2.017(5)	Cu15–O15 1.930(3)	Cu22–N43 1.963(4)
P1B_2–O1B_2 1.504(17)	Cu7–O7 1.918(4)	Cu15–N29 1.967(5)	Cu23–O23 1.921(3)
P1B_2–O3B_2 1.510(17)	Cu7–O6 1.950(3)	Cu15–N28 1.975(4)	Cu23–O29 1.945(3)
P1_2–C1_2 1.787(8)	Cu7–N12 2.012(5)	Cu16–O15 1.918(4)	Cu23–N58 1.987(4)
P1B_2–C1_2 1.93(3)	Cu7–N13 2.032(5)	Cu16–O16 1.931(4)	Cu23–N45 2.020(4)
C1_2–C2_2 1.571(8)	Cu8–O8 1.895(4)	Cu16–N30 1.974(5)	Cu23–O22 2.368(3)
C2_2–C3_2 1.510(10)	Cu8–O7 1.918(3)	Cu16–N31 1.979(4)	Cu24–O24 1.879(3)
C3_2–C4_2 1.556(11)	Cu8–N14 1.947(5)	Cu17–O16 1.910(3)	Cu24–O23 1.946(3)
Cu1–O1 1.921(3)	Cu8–N15 1.974(4)	Cu17 O17 1.918(3)	Cu24–N46 1.954(4)
Cu1–O9 1.943(4)	Cu9–O8 1.893(3)	Cu17–N32 1.971(4)	Cu24–N47 1.987(4)
Cu1–N1 1.987(5)	Cu9–O9 1.937(3)	Cu17–N33 1.950(9)	Cu25–O24 1.875(3)
Cu1–N18 1.990(4)	Cu9–N16 1.969(4)	Cu17–N33B 2.01(2)	Cu25–O25 1.932(3)
Cu1–O10 2.431(3)	Cu9–N17 1.976(4)	Cu18–O18 1.902(3)	Cu25–N49 1.972(4)
Cu2–O1 1.897(4)	Cu10–O22 1.924(3)	Cu18–O17 1.910(4)	Cu25–N48 1.974(4)
Cu2–O2 1.902(4)	Cu10–O10 1.927(3)	Cu18–N34 1.890(7)	Cu26–O25 1.931(3)
Cu2–N3 1.953(5)	Cu10–N19 1.962(5)	Cu18–N34B1 2.236(19)	Cu26–O26 1.944(3)
Cu2–N2 1.965(5)	Cu10–N44 1.968(5)	Cu18–N35 2.000(9)	Cu26–N51 1.969(4)
Cu3–O2 1.930(3)	Cu11–O11 1.922(3)	Cu18–N35B 1.879(15)	Cu26–N50 1.998(4)
Cu3–O3 1.943(3)	Cu11–O10 1.924(3)	Cu19–O19 1.909(3)	Cu26–O14 2.407(4)
Cu3–N4 1.991(5)	Cu11–N21 1.955(5)	Cu19–O18 1.911(3)	Cu27–O26 1.919(3)
Cu3–N5 2.009(4)	Cu11–N20 1.980(4)	Cu19–N36 1.902(11)	Cu27–O27 1.954(3)
Cu3–O13 2.373(4)	Cu12–O12 1.916(3)	Cu19–N36B 2.061(17)	Cu27–N53 1.970(4)
Cu4–O4 1.887(3)	Cu12–O11 1.938(3)	Cu19–N37 1.961(4)	Cu27–N52 2.009(4)
Cu4–O3 1.926(4)	Cu12–N23 1.980(4)	Cu20–O19 1.926(3)	Cu28–O27 1.942(3)
Cu4–N6 1.954(4)	Cu12–N22 1.987(5)	Cu20–O20 1.931(4)	Cu28–O28 1.942(3)
Cu4–N7 1.974(5)	Cu13–O12 1.911(3)	Cu20–N39 1.951(5)	Cu28–N54 1.990(4)
Cu5–O4 1.901(3)	Cu13–O13 1.932(3)	Cu20–N38 1.976(5)	Cu28–N55 2.002(4)
Cu5–O5 1.927(4)	Cu13–N25 1.956(4)	Cu21–O21 1.914(3)	Cu29–O29 1.925(3)
Cu5–N9 1.962(4)	Cu13–N24 1.965(5)	Cu21–O20 1.932(3)	Cu29–O28 1.946(3)
Cu5–N8 1.966(5)	Cu14–O13 1.917(3)	Cu21–N40 1.951(5)	Cu29–N56 1.993(4)
	Cu14–O14 1.942(3)	Cu21–N41 1.959(4)	Cu29–N57 2.016(4)

Table S26. Selected bond lengths for UNIT 1 of **7** (Cu1_1–Cu8_1: Cu$_8$-ring; Cu9_1–Cu22_1: Cu$_{14}$-ring; Cu23_1–Cu31_1: Cu$_9$-ring; O1_2–O3_2: $^nC_{12}PO_3^{2-}$ anion).

P1_2–O1_2 1.508(5)	Cu8_1–N14_1 1.978(5)	Cu16_1–N31_1 1.951(5)	Cu24_1–N47_1 1.978(5)
P1_2–O2_2 1.502(4)	Cu8_1–N15_1 2.000(5)	Cu16_1–N30_1 1.966(5)	Cu25_1–O25_1 1.932(4)
P1_2–O3_2 1.496(4)	Cu9_1–O22_1 1.915(4)	Cu17_1–O17_1 1.906(4)	Cu25_1–O24_1 1.939(3)
P1_2–C1_2 1.835(6)	Cu9_1–O9_1 1.930(3)	Cu17_1–O16_1 1.915(4)	Cu25_1–N48_1 2.003(5)
Cu1_1–O8_1 1.924(4)	Cu9_1–N17_1 1.957(5)	Cu17_1–N32_1 1.949(5)	Cu25_1–N49_1 2.007(4)
Cu1_1–O1_1 1.950(3)	Cu9_1–N44_1 1.973(4)	Cu17_1–N33_1 1.956(5)	Cu25_1–O12_1 2.349(4)
Cu1_1–N1_1 1.984(5)	Cu10_1–O9_1 1.919(4)	Cu18_1–O17_1 1.906(4)	Cu26_1–O26_1 1.892(4)
Cu1_1–N16_1 1.992(4)	Cu10_1–O10_1 1.924(3)	Cu18_1–O18_1 1.932(4)	Cu26_1–O25_1 1.920(4)
Cu2_1–O1_1 1.928(3)	Cu10_1–N19_1 1.946(5)	Cu18_1–N35_1 1.953(6)	Cu26_1–N51_1 1.961(5)
Cu2_1–O2_1 1.932(4)	Cu10_1–N18_1 1.970(4)	Cu18_1–N34_1 1.962(5)	Cu26_1–N50_1 1.988(5)
Cu2_1–N3_1 2.003(4)	Cu11_1–O10_1 1.926(4)	Cu19_1–O19_1 1.890(4)	Cu27_1–O26_1 1.906(4)
Cu2_1–N2_1 2.008(4)	Cu11_1–O11_1 1.929(3)	Cu19_1–O18_1 1.920(4)	Cu27_1–O27_1 1.931(4)
Cu3_1–O3_1 1.900(4)	Cu11_1–N21_1 1.956(5)	Cu19_1–N36_1 1.945(6)	Cu27_1–N53_1 1.969(5)
Cu3_1–O2_1 1.939(3)	Cu11_1–N20_1 1.964(5)	Cu19_1–N37_1 1.977(4)	Cu27_1–N52_1 2.000(5)
Cu3_1–N4_1 1.986(5)	Cu12_1–O12_1 1.924(4)	Cu20_1–O19_1 1.910(4)	Cu28_1–O28_1 1.934(4)
Cu3_1–N5_1 1.987(4)	Cu12_1–O11_1 1.927(3)	Cu20_1–O20_1 1.920(4)	Cu28_1–O27_1 1.935(4)
Cu4_1–O3_1 1.891(4)	Cu12_1–N22_1 1.973(5)	Cu20_1–N38_1 1.968(5)	Cu28_1–N54_1 1.999(5)
Cu4_1–O4_1 1.922(4)	Cu12_1–N23_1 1.979(4)	Cu20_1–N39_1 1.970(5)	Cu28_1–N55_1 1.995(10)
Cu4_1–N6_1 1.959(5)	Cu13_1–O13_1 1.917(4)	Cu21_1–O21_1 1.900(4)	Cu28_1–N55B_1 2.06(3)
Cu4_1–N7_1 1.966(5)	Cu13_1–O12_1 1.932(3)	Cu21_1–O20_1 1.908(4)	Cu29_1–O29_1 1.911(4)
Cu5_1–O5_1 1.925(3)	Cu13_1–N24_1 1.957(5)	Cu21_1–N40_1 1.956(5)	Cu29_1–O28_1 1.919(3)
Cu5_1–O4_1 1.954(4)	Cu13_1–N25_1 1.982(4)	Cu21_1–N41_1 1.965(5)	Cu29_1–N56_1 1.993(10)
Cu5_1–N8_1 1.972(4)	Cu14_1–O13_1 1.903(4)	Cu22_1–O22_1 1.924(4)	Cu29_1–N56B_1 1.99(3)
Cu5_1–N9_1 1.976(5)	Cu14_1–O14_1 1.913(4)	Cu22_1–O21_1 1.931(4)	Cu29_1–N57_1 1.978(4)
Cu6_1–O5_1 1.903(4)	Cu14_1–N26_1 1.946(5)	Cu22_1–N42_1 1.959(5)	Cu30_1–O29_1 1.896(4)
Cu6_1–O6_1 1.944(4)	Cu14_1–N27_1 1.966(15)	Cu22_1–N43_1 1.963(5)	Cu30_1–O30_1 1.915(4)
Cu6_1–N11_1 1.985(4)	Cu14_1–N27B_1 1.92(2)	Cu23_1–O23_1 1.908(3)	Cu30_1–N59_1 1.966(4)
Cu6_1–N10_1 1.989(4)	Cu15_1–O15_1 1.909(4)	Cu23_1–O31_1 1.908(4)	Cu30_1–N58_1 1.978(5)
Cu7_1–O7_1 1.896(4)	Cu15_1–O14_1 1.915(4)	Cu23_1–N45_1 1.956(5)	Cu31_1–O31_1 1.925(3)
Cu7_1–O6_1 1.916(4)	Cu15_1–N29_1 1.962(5)	Cu23_1–N62_1 1.981(4)	Cu31_1–O30_1 1.942(4)
Cu7_1–N13_1 1.957(5)	Cu15_1–N28_1 1.976(15)	Cu24_1–O23_1 1.900(4)	Cu31_1–N60_1 1.989(4)
Cu7_1–N12_1 1.977(5)	Cu15_1–N28B_1 1.90(2)	Cu24_1–O24_1 1.920(3)	Cu31_1–N61_1 2.008(5)
Cu8_1–O7_1 1.918(4)	Cu16_1–O15_1 1.907(4)	Cu24_1–N46_1 1.956(4)	Cu31_1–O22_1 2.376(4)
Cu8_1–O8_1 1.928(4)	Cu16_1–O16_1 1.929(4)		

Table S27. Selected bond lengths for UNIT 2 of **7** (Cu1_3–Cu8_3: Cu$_8$-ring; Cu9_3–Cu22_3: Cu$_{14}$-ring; Cu23_3–Cu31_3: Cu$_9$-ring; O1_4–O3_4: ^{n}C$_{12}$PO$_3^{2-}$ anion).

P1_4–O1_4 1.514(4)
P1_4–O2_4 1.509(4)
P1_4–O3_4 1.517(5)
P1_4–C1_4 1.809(6)
Cu1_3–O1_3 1.920(4)
Cu1_3–O8_3 1.939(4)
Cu1_3–N1_3 1.980(5)
Cu1_3–N16_3 1.993(4)
Cu1_3–O9_3 2.430(4)
Cu2_3–O1_3 1.891(4)
Cu2_3–O2_3 1.900(4)
Cu2_3–N2_3 1.963(5)
Cu2_3–N3_3 1.981(5)
Cu3_3–O3_3 1.921(4)
Cu3_3–O2_3 1.940(4)
Cu3_3–N4_3 1.996(5)
Cu3_3–N5_3 2.003(5)
Cu4_3–O3_3 1.917(4)
Cu4_3–O4_3 1.950(4)
Cu4_3–N6_3 1.992(5)
Cu4_3–N7_3 1.993(4)
Cu5_3–O5_3 1.888(4)
Cu5_3–O4_3 1.916(4)
Cu5_3–N8_3 1.961(5)
Cu5_3–N9_3 1.971(5)
Cu6_3–O5_3 1.908(4)
Cu6_3–O6_3 1.936(3)
Cu6_3–N10_3 1.983(4)
Cu6_3–N11_3 1.994(5)
Cu7_3–O6_3 1.923(4)
Cu7_3–O7_3 1.940(3)
Cu7_3–N12_3 1.984(4)
Cu7_3–N13_3 1.989(4)
Cu8_3–O8_3 1.908(4)
Cu8_3–O7_3 1.944(3)
Cu8_3–N14_3 1.965(4)
Cu8_3–N15_3 1.981(4)
Cu9_3–O22_3 1.912(4)
Cu9_3–O9_3 1.930(4)
Cu9_3–N44_3 1.949(5)
Cu9_3–N17_3 1.969(5)
Cu10_3–O9_3 1.905(4)
Cu10_3–O10_3 1.913(4)
Cu10_3–N19_3 1.934(5)
Cu10_3–N18_3 1.969(5)
Cu11_3–O11_3 1.903(4)
Cu11_3–O10_3 1.914(4)
Cu11_3–N21_3 1.965(5)
Cu11_3–N20_3 1.974(5)
Cu12_3–O11_3 1.894(4)
Cu12_3–O12_3 1.921(4)
Cu12_3–N23_3 1.941(6)
Cu12_3–N22_3 1.973(5)
Cu13_3–O13_3 1.905(4)
Cu13_3–O12_3 1.927(4)
Cu13_3–N25_3 1.959(5)
Cu13_3–N24_3 1.966(6)
Cu14_3–O13_3 1.898(4)
Cu14_3–O14_3 1.926(4)
Cu14_3–N26_3 1.950(6)
Cu14_3–N27_3 1.964(5)
Cu15_3–O15_3 1.913(5)
Cu15_3–O14_3 1.925(4)
Cu15_3–N28_3 1.943(6)
Cu15_3–N29_3 1.969(5)
Cu16_3–O15_3 1.899(4)
Cu16_3–O16_3 1.910(4)
Cu16_3–N30_3 1.966(6)
Cu16_3–N31_3 1.922(14)
Cu16_3–N31B_3 2.04(2)
Cu17_3–O17_3 1.903(4)
Cu17_3–O16_3 1.920(4)
Cu17_3–N32_3 1.946(11)
Cu17_3–N32B_3 1.99(2)
Cu17_3–N33_3 1.966(6)
Cu18_3–O17_3 1.917(4)
Cu18_3–O18_3 1.921(3)
Cu18_3–N34_3 1.958(5)
Cu18_3–N35_3 1.959(5)
Cu19_3–O18_3 1.924(4)
Cu19_3–O19_3 1.929(4)
Cu19_3–N37_3 1.965(5)
Cu19_3–N36_3 1.967(5)
Cu20_3–O20_3 1.915(4)
Cu20_3–O19_3 1.922(4)
Cu20_3–N39_3 1.953(5)
Cu20_3–N38_3 1.966(5)
Cu21_3–O21_3 1.926(4)
Cu21_3–O20_3 1.937(4)
Cu21_3–N40_3 1.957(5)
Cu21_3–N41_3 1.96(3)
Cu21_3–N41B_3 1.98(2)
Cu22_3–O22_3 1.916(4)
Cu22_3–O21_3 1.929(3)
Cu22_3–N42_3 1.874(18)
Cu22_3–N42B_3 2.000(17)
Cu22_3–N43_3 1.967(4)
Cu23_3–O23_3 1.901(4)
Cu23_3–O31_3 1.909(4)
Cu23_3–N45_3 1.965(5)
Cu23_3–N62_3 1.974(4)
Cu24_3–O23_3 1.911(4)
Cu24_3–O24_3 1.921(3)
Cu24_3–N46_3 1.971(4)
Cu24_3–N47_3 1.983(5)
Cu25_3–O24_3 1.928(4)
Cu25_3–O25_3 1.947(4)
Cu25_3–N48_3 1.998(5)
Cu25_3–N49_3 2.014(5)
Cu26_3–O26_3 1.900(4)
Cu26_3–O25_3 1.924(4)
Cu26_3–N50_3 1.978(5)
Cu26_3–N51_3 1.979(5)
Cu27_3–O26_3 1.894(4)
Cu27_3–O27_3 1.918(4)
Cu27_3–N52_3 1.964(5)
Cu27_3–N53_3 1.980(5)
Cu28_3–O28_3 1.932(4)
Cu28_3–O27_3 1.937(4)
Cu28_3–N55_3 1.989(5)
Cu28_3–N54_3 2.006(4)
Cu28_3–O18_3 2.384(4)
Cu29_3–O29_3 1.905(4)
Cu29_3–O28_3 1.922(4)
Cu29_3–N57_3 1.949(5)
Cu29_3–N56_3 1.976(5)
Cu30_3–O30_3 1.904(4)
Cu30_3–O29_3 1.906(3)
Cu30_3–N58_3 1.956(5)
Cu30_3–N59_3 1.989(4)
Cu31_3–O30_3 1.920(3)
Cu31_3–O31_3 1.943(4)
Cu31_3–N61_3 1.984(4)
Cu31_3–N60_3 2.014(4)
Cu31_3–O22_3 2.389(4)

Table S28. Selected bond lengths for **8** (Cu1–Cu9 and Cu1, Cu2B–Cu7B, Cu8, Cu9: Cu$_9$-ring partially disordered over two positions with 0.56/0.44 occupancy; Cu10–Cu23: Cu$_{14}$-ring; Cu24–Cu31: Cu$_8$-ring; O1_2–O3_2, C1_2–C7_2: BnPO$_3^{2-}$ anion).

P1_2–O1_2 1.531(4)	Cu5–N8 1.989(10)	Cu12–O12 1.895(5)	Cu22–O21 1.925(4)
P1_2–O2_2 1.519(4)	Cu5–N9 1.950(10)	Cu12–N22 1.943(7)	Cu22–O22 1.903(4)
P1_2–O3_2 1.525(4)	Cu5B–O4B 1.898(9)	Cu12–N23 1.947(7)	Cu22–N42 1.955(6)
P1_2–C1_2 1.824(8)	Cu5B–O5B 1.905(10)	Cu13–O12 1.926(4)	Cu22–N43 1.959(5)
C1_2–C2_2 1.470(12)	Cu5B–N8B 1.968(11)	Cu13–O13 1.922(6)	Cu23–O22 1.921(4)
C2_2–C3_2 1.407(14)	Cu5B–N9B 1.948(12)	Cu13–N24 1.942(8)	Cu23–O23 1.921(4)
C3_2–C4_2 1.358(15)	Cu6–O5 1.928(9)	Cu13–N25 1.950(6)	Cu23–N44 1.954(5)
C4_2–C5_2 1.39(2)	Cu6–O6 1.958(9)	Cu14–O13 1.918(4)	Cu23–N45 1.954(6)
C5_2–C6_2 1.42(2)	Cu6–O17 2.332(6)	Cu14–O14 1.924(5)	Cu24–O24 1.956(4)
C6_2–C7_2 1.402(16)	Cu6–N10 2.025(11)	Cu14–N26 1.974(7)	Cu24–O31 1.911(4)
C7_2–C2_2 1.401(12)	Cu6–N11 2.031(10)	Cu14–N27 1.965(6)	Cu24–N47 1.975(5)
Cu1–O1 1.921(4)	Cu6B–O5B 1.939(11)	Cu15–O14 1.926(5)	Cu24–N62 1.994(6)
Cu1–O9 1.926(4)	Cu6B–O6B 1.957(11)	Cu15–O15 1.936(5)	Cu25–O24 1.932(4)
Cu1–N1 1.991(6)	Cu6B–N10B 2.010(11)	Cu15–N28 1.972(7)	Cu25–O25 1.873(4)
Cu1–N18 1.977(5)	Cu6B–N11B 2.019(12)	Cu15–N29 1.956(7)	Cu25–N48 1.964(5)
Cu2–O1 1.996(8)	Cu7–O6 1.912(9)	Cu16–O15 1.922(5)	Cu25–N49 1.969(6)
Cu2–O2 1.916(9)	Cu7–O7 1.925(9)	Cu16–O16 1.925(4)	Cu26–O25 1.915(4)
Cu2–N2 1.943(9)	Cu7–N12 1.964(9)	Cu16–N30 1.969(6)	Cu26–O26 1.915(5)
Cu2–N3 1.978(9)	Cu7–N13 1.948(9)	Cu16–N31 1.962(6)	Cu26–N50 1.964(6)
Cu2B–O1 1.811(10)	Cu7B–O6B 1.932(11)	Cu17–O16 1.940(5)	Cu26–N51 1.987(5)
Cu2B–O2B 1.918(10)	Cu7B–O7B 1.916(10)	Cu17–O17 1.914(4)	Cu27–O26 1.925(4)
Cu2B–N2 2.000(11)	Cu7B–N12B 1.946(11)	Cu17–N32 1.947(5)	Cu27–O27 1.963(4)
Cu2B–N3B 1.995(11)	Cu7B–N13B 1.935(10)	Cu17–N33 1.953(6)	Cu27–O14 2.398(4)
Cu3–O2 1.941(8)	Cu8–O7 1.940(8)	Cu18–O17 1.922(5)	Cu27–N52 2.013(6)
Cu3–O3 1.938(9)	Cu8–O7B 1.902(10)	Cu18–O18 1.915(4)	Cu27–N53 1.994(5)
Cu3–O13 2.273(6)	Cu8–O8 1.927(4)	Cu18–N34 1.955(5)	Cu28–O27 1.947(4)
Cu3–N4 2.005(9)	Cu8–N14B 1.989(11)	Cu18–N35 1.953(6)	Cu28–O28 1.924(4)
Cu3–N5 1.966(8)	Cu8–N14 2.013(9)	Cu19–O18 1.897(4)	Cu28–N54 1.976(5)
Cu3B–O2B 1.940(10)	Cu8–N15 1.983(5)	Cu19–O19 1.927(4)	Cu28–N55 1.989(5)
Cu3B–O3B 1.935(11)	Cu9–O8 1.943(4)	Cu19–N36 1.975(6)	Cu29–O28 1.927(4)
Cu3B–N4B 2.006(10)	Cu9–O9 1.939(4)	Cu19–N37 1.938(12)	Cu29–O29 1.918(4)
Cu3B–N5B 1.976(10)	Cu9–N16 2.022(6)	Cu19–N37B 2.057(16)	Cu29–N56 1.998(4)
Cu4–O3 1.909(8)	Cu9–N17 2.006(6)	Cu20–O19 1.922(4)	Cu29–N57 1.966(5)
Cu4–O4 1.905(8)	Cu10–O10 1.914(4)	Cu20–O20 1.905(4)	Cu30–O29 1.899(4)
Cu4–N6 1.973(9)	Cu10–O23 1.919(4)	Cu20–N38 1.843(12)	Cu30–O30 1.921(4)
Cu4–N7 1.974(9)	Cu10–N19 1.966(6)	Cu20–N38B 2.194(16)	Cu30–N58 1.965(5)
Cu4B–O3B 1.928(10)	Cu10–N46 1.960(6)	Cu20–N39 1.978(6)	Cu30–N59 1.981(5)
Cu4B–O4B 1.914(9)	Cu11–O10 1.914(4)	Cu21–O20 1.921(4)	Cu31–O30 1.940(4)
Cu4B–N6B 1.964(10)	Cu11–O11 1.911(4)	Cu21–O21 1.908(4)	Cu31–O31 1.918(4)
Cu4B–N7B 1.980(11)	Cu11–N20 1.961(6)	Cu21–N40 1.944(6)	Cu31–N60 1.991(5)
Cu5–O4 1.897(8)	Cu11–N21 1.959(6)	Cu21–N41 1.955(7)	Cu31–N61 1.993(5)
Cu5–O5 1.903(9)	Cu12–O11 1.917(4)		

Table S29. Selected bond lengths for the major component ($Cu_{10+12+6}$, 82%) in UNIT 1 of **9** (Cu1–Cu10: Cu_{10}-ring; Cu11–Cu22: Cu_{12}-ring; Cu23–Cu28: Cu_6-ring; O1_1–O3_1, O1B_1–O3B_1, C1_1–C6_1: $PhPO_3^{2-}$ anion with its PO_3 unit disordered over two positions with 0.86/0.14 occupancy).

P1_1–O1_1 1.515(5)	Cu5–N8 1.956(8)	Cu13–N24 1.947(5)	Cu21–N40 1.973(5)
P1_1–O2_1 1.518(4)	Cu5–N9 1.972(6)	Cu13–N25 1.969(4)	Cu22–O22 1.927(3)
P1_1–O3_1 1.504(4)	Cu6–O5 1.935(5)	Cu14–O13 1.918(3)	Cu22–O21 1.940(3)
P1_1–C1_1 1.802(6)	Cu6–O6 1.902(5)	Cu14–O14 1.934(3)	Cu22–N42 1.970(4)
P1B_1–O1B_1 1.528(15)	Cu6–N10 1.966(6)	Cu14–N27 1.957(5)	Cu22–N43 1.974(4)
P1B_1–O2B_1 1.467(14)	Cu6–N11 1.970(8)	Cu14–N26 1.975(4)	Cu23–O23 1.940(3)
P1B_1–O3B_1 1.540(15)	Cu7–O6 1.919(6)	Cu15–O14 1.923(4)	Cu23–O28 1.943(3)
P1B_1–C1_1 1.802(6)	Cu7–O7 1.903(5)	Cu15–O15 1.947(4)	Cu23–N56 1.990(4)
C1_1–C2_1 1.390(8)	Cu7–N12 1.945(7)	Cu15–N29 1.957(5)	Cu23–N45 1.991(4)
C2_1–C3_1 1.385(10)	Cu7–N13 1.972(8)	Cu15–N28 1.987(5)	Cu23–O22 2.340(3)
C3_1–C4_1 1.397(12)	Cu8–O7 1.928(5)	Cu16–O15 1.923(4)	Cu24–O24 1.927(4)
C4_1–C5_1 1.341(12)	Cu8–O8 1.947(5)	Cu16–O16 1.937(3)	Cu24–O23 1.933(3)
C5_1–C6_1 1.380(10)	Cu8–N14 2.006(5)	Cu16–N31 1.958(5)	Cu24–N46 1.979(4)
C1_1–C6_1 1.401(8)	Cu8–N15 1.994(6)	Cu16–N30 1.979(4)	Cu24–N47 1.983(5)
Cu1–O1 1.914(4)	Cu8–O19 2.318(5)	Cu17–O16 1.931(3)	Cu25–O25 1.930(3)
Cu1–O10 1.932(3)	Cu9–O8 1.883(7)	Cu17–O17 1.935(3)	Cu25–O24 1.946(3)
Cu1–N1 1.961(5)	Cu9–O9 1.900(4)	Cu17–N33 1.969(4)	Cu25–N48 1.977(5)
Cu1–N20 1.990(5)	Cu9–N16 1.978(6)	Cu17–N32 1.971(4)	Cu25–N49 1.995(4)
Cu2–O1 1.868(5)	Cu9–N17 1.968(5)	Cu18–O17 1.929(4)	Cu25–O14 2.378(4)
Cu2–O2 1.903(5)	Cu10–O9 1.915(4)	Cu18–O18 1.925(3)	Cu26–O25 1.933(3)
Cu2–N2 1.948(6)	Cu10–O10 1.917(3)	Cu18–N34 1.963(4)	Cu26–O26 1.941(3)
Cu2–N3 1.952(6)	Cu10–N18 1.969(5)	Cu18–N35 1.990(5)	Cu26–N51 1.996(4)
Cu3–O2 1.953(5)	Cu10–N19 1.987(4)	Cu19–O19 1.922(3)	Cu26–N50 1.996(4)
Cu3–O3 1.952(5)	Cu11–O22 1.925(3)	Cu19–O18 1.924(4)	Cu26–O16 2.361(4)
Cu3–N4 1.970(7)	Cu11–O11 1.957(3)	Cu19–N37 1.971(5)	Cu27–O27 1.933(3)
Cu3–N5 2.047(7)	Cu11–N21 1.962(4)	Cu19–N36 1.973(5)	Cu27–O26 1.942(3)
Cu3–O13 2.346(5)	Cu11–N44 1.986(5)	Cu20–O19 1.911(4)	Cu27–N52 1.986(4)
Cu4–O3 1.921(5)	Cu12–O11 1.928(3)	Cu20–O20 1.928(3)	Cu27–N53 1.994(5)
Cu4–O4 1.898(6)	Cu12–O12 1.940(3)	Cu20–N39 1.945(5)	Cu27–O18 2.407(4)
Cu4–N6 2.002(8)	Cu12–N22 1.950(4)	Cu20–N38 1.974(4)	Cu28–O27 1.921(3)
Cu4–N7 1.971(7)	Cu12–N23 1.978(4)	Cu21–O21 1.925(3)	Cu28–O28 1.941(3)
Cu5–O4 1.910(5)	Cu13–O13 1.920(4)	Cu21–O20 1.939(3)	Cu28–N55 1.976(4)
Cu5–O5 1.930(5)	Cu13–O12 1.938(3)	Cu21–N41 1.960(4)	Cu28–N54 1.984(4)

Table S30. Selected bond lengths for the minor component (Cu_{9+12+6}, 18%) in UNIT 1 of **9** (Cu1, Cu2B–Cu7B, Cu9, Cu10: Cu_9-ring; Cu11–Cu22: Cu_{12}-ring; Cu23–Cu28: Cu_6-ring; O1_1–O3_1, O1B_1–O3B_1, C1_1–C6_1: $PhPO_3^{2-}$ anion with its PO_3 unit disordered over two positions with 0.86/0.14 occupancy).

P1_1–O1_1 1.515(5)	Cu5B–N8B 1.971(15)	Cu14–O14 1.934(3)	Cu22–O21 1.940(3)
P1_1–O2_1 1.518(4)	Cu5B–N9B 1.979(15)	Cu14–N27 1.957(5)	Cu22–N42 1.970(4)
P1_1–O3_1 1.504(4)	Cu6B–O5B 1.938(13)	Cu14–N26 1.975(4)	Cu22–N43 1.974(4)
P1_1–C1_1 1.802(6)	Cu6B–O6B 1.918(13)	Cu15–O14 1.923(4)	Cu23–O23 1.940(3)
P1B_1–O1B_1 1.528(15)	Cu6B–O17 2.399(8)	Cu15–O15 1.947(4)	Cu23–O28 1.943(3)
P1B_1–O2B_1 1.467(14)	Cu6B–N10B 2.003(15)	Cu15–N29 1.957(5)	Cu23–N56 1.990(4)
P1B_1–O3B_1 1.540(15)	Cu6B–N11B 2.77(3)	Cu15–N28 1.987(5)	Cu23–N45 1.991(4)
P1B_1–C1_1 1.802(6)	Cu7B–O6B 1.931(14)	Cu16–O15 1.923(4)	Cu23–O22 2.340(3)
C1_1–C2_1 1.390(8)	Cu7B–O7B 1.902(17)	Cu16–O16 1.937(3)	Cu24–O24 1.927(4)
C2_1–C3_1 1.385(10)	Cu7B–N12B 1.978(15)	Cu16–N31 1.958(5)	Cu24–O23 1.933(3)
C3_1–C4_1 1.397(12)	Cu7B–N13B 2.001(15)	Cu16–N30 1.979(4)	Cu24–N46 1.979(4)
C4_1–C5_1 1.341(12)	Cu9–O7B 2.07(3)	Cu17–O16 1.931(3)	Cu24–N47 1.983(5)
C5_1–C6_1 1.380(10)	Cu9–O9 1.900(4)	Cu17–O17 1.935(3)	Cu25–O25 1.930(3)
C1_1–C6_1 1.401(8)	Cu9–N14B 1.91(2)	Cu17–N33 1.969(4)	Cu25–O24 1.946(3)
Cu1–O1 1.914(4)	Cu9–N17 1.968(5)	Cu17–N32 1.971(4)	Cu25–N48 1.977(5)
Cu1–O10 1.932(3)	Cu10–O9 1.915(4)	Cu18–O17 1.929(4)	Cu25–N49 1.995(4)
Cu1–N1 1.961(5)	Cu10–O10 1.917(3)	Cu18–O18 1.925(3)	Cu25–O14 2.378(4)
Cu1–N20 1.990(5)	Cu10–N18 1.969(5)	Cu18–N34 1.963(4)	Cu26–O25 1.933(3)
Cu2B–O1 2.157(14)	Cu10–N19 1.987(4)	Cu18–N35 1.990(5)	Cu26–O26 1.941(3)
Cu2B–O2B 1.898(16)	Cu11–O22 1.925(3)	Cu19–O19 1.922(3)	Cu26–N51 1.996(4)
Cu2B–N2 2.101(16)	Cu11–O11 1.957(3)	Cu19–O18 1.924(4)	Cu26–N50 1.996(4)
Cu2B–N3B 1.975(18)	Cu11– N21 1.962(4)	Cu19–N37 1.971(5)	Cu26–O16 2.361(4)
Cu3B–O2B 1.961(16)	Cu11–N44 1.986(5)	Cu19–N36 1.973(5)	Cu27–O27 1.933(3)
Cu3B–O3B 1.962(15)	Cu12–O11 1.928(3)	Cu20–O19 1.911(4)	Cu27–O26 1.942(3)
Cu3B–N4B 1.964(15)	Cu12–O12 1.940(3)	Cu20–O20 1.928(3)	Cu27–N52 1.986(4)
Cu3B–N5B 2.046(16)	Cu12–N22 1.950(4)	Cu20–N39 1.945(5)	Cu27–N53 1.994(5)
Cu4B–O3B 1.936(15)	Cu12–N23 1.978(4)	Cu20–N38 1.974(4)	Cu27–O18 2.407(4)
Cu4B–O4B 1.881(16)	Cu13–O13 1.920(4)	Cu21–O21 1.925(3)	Cu28–O27 1.921(3)
Cu4B–N6B 2.060(16)	Cu13–O12 1.938(3)	Cu21–O20 1.939(3)	Cu28–O28 1.941(3)
Cu4B–N7B 1.960(16)	Cu13–N24 1.947(5)	Cu21–N41 1.960(4)	Cu28–N54 1.984(4)
Cu5B–O4B 1.930(15)	Cu13–N25 1.969(4)	Cu21–N40 1.973(5)	Cu28–N55 1.976(4)
Cu5B–O5B 1.916(14)	Cu14–O13 1.918(3)	Cu22–O22 1.927(3)	

Table S31. Selected bond lengths for the major component ($Cu_{10+12+6}$, 91%) in UNIT 2 of **9** (Cu1–Cu10: Cu_{10}-ring; Cu11–Cu22: Cu_{12}-ring; Cu23–Cu28: Cu_6-ring; O1_2–O3_2, O1B_2–O3B_2, C1_2–C6_2: $PhPO_3^{2-}$ anion with its PO_3 unit disordered over two positions with 0.89/0.11 occupancy).

P1_2–O1_2 1.509(4)	Cu5–N8 1.974(5)	Cu13–N24 1.958(5)	Cu21–N41 1.959(4)
P1_2–O2_2 1.526(4)	Cu5–N9 1.964(5)	Cu13–N25 1.981(5)	Cu22–O21 1.947(3)
P1_2–O3_2 1.537(4)	Cu6–O5 1.929(4)	Cu14–O13 1.923(4)	Cu22–O22 1.932(3)
P1_2–C1_2 1.811(5)	Cu6–O6 1.917(4)	Cu14–O14 1.930(3)	Cu22–N42 1.988(4)
P1B_2–O2B_2 1.480(15)	Cu6–N10 1.984(5)	Cu14–N26 1.968(5)	Cu22–N43 1.967(4)
P1B_2–O1B_2 1.540(16)	Cu6–N11 1.968(5)	Cu14–N27 1.956(5)	Cu23–O23 1.949(3)
P1B_2–O3B_2 1.546(16)	Cu7–O6 1.906(4)	Cu15–O14 1.928(3)	Cu23–O28 1.935(3)
P1B_2–C1_2 1.811(5)	Cu7–O7 1.916(4)	Cu15–O15 1.936(4)	Cu23–O22 2.339(3)
C1_2–C2_2 1.395(8)	Cu7–N12 1.975(5)	Cu15–N28 1.975(5)	Cu23–N45 1.998(4)
C2_2–C3_2 1.377(9)	Cu7–N13 1.962(5)	Cu15–N29 1.953(4)	Cu23–N56 1.987(4)
C3_2–C4_2 1.391(11)	Cu8–O7 1.937(4)	Cu16–O15 1.933(3)	Cu24–O23 1.944(3)
C4_2–C5_2 1.353(11)	Cu8–O8 1.942(4)	Cu16–O16 1.939(3)	Cu24–O24 1.922(3)
C5_2–C6_2 1.390(9)	Cu8–O19 2.371(4)	Cu16–N30 1.965(4)	Cu24–N46 1.985(4)
C6_2–C1_2 1.388(7)	Cu8–N14 2.010(5)	Cu16–N31 1.968(4)	Cu24–N47 1.989(5)
Cu1–O1 1.901(3)	Cu8–N15 2.005(5)	Cu17–O16 1.938(3)	Cu25–O24 1.929(3)
Cu1–O10 1.925(3)	Cu9–O8 1.908(4)	Cu17–O17 1.945(3)	Cu25–O25 1.942(3)
Cu1–N1 1.983(5)	Cu9–O9 1.891(4)	Cu17–N32 1.969(5)	Cu25–O14 2.395(3)
Cu1–N20 1.980(4)	Cu9–N16 1.954(5)	Cu17–N33 1.977(4)	Cu25–N48 1.992(5)
Cu2–O1 1.918(4)	Cu9–N17 1.939(5)	Cu18–O17 1.931(3)	Cu25–N49 1.982(4)
Cu2–O2 1.905(4)	Cu10–O9 1.919(3)	Cu18–O18 1.928(3)	Cu26–O25 1.939(3)
Cu2–N2 1.926(5)	Cu10–O10 1.934(3)	Cu18–N34 1.953(4)	Cu26–O26 1.936(3)
Cu2–N3 1.958(5)	Cu10–N18 1.990(5)	Cu18–N35 1.973(4)	Cu26–O16 2.328(3)
Cu3–O2 1.927(4)	Cu10–N19 1.991(4)	Cu19–O18 1.928(3)	Cu26–N50 1.996(4)
Cu3–O3 1.941(4)	Cu11–O22 1.932(3)	Cu19–O19 1.919(3)	Cu26–N51 1.995(4)
Cu3–O13 2.342(4)	Cu11–O11 1.934(3)	Cu19–N36 1.953(4)	Cu27–O26 1.934(3)
Cu3–N4 2.013(6)	Cu11–N21 1.969(4)	Cu19–N37 1.966(4)	Cu27–O27 1.943(3)
Cu3–N5 2.002(5)	Cu11–N44 1.986(4)	Cu20–O19 1.916(3)	Cu27–O18 2.399(4)
Cu4–O3 1.905(4)	Cu12–O12 1.934(3)	Cu20–O20 1.935(3)	Cu27–N52 1.986(4)
Cu4–O4 1.899(4)	Cu12–O11 1.936(4)	Cu20–N38 1.979(4)	Cu27–N53 1.987(4)
Cu4–N6 1.953(6)	Cu12–N22 1.967(4)	Cu20–N39 1.948(4)	Cu28–O27 1.937(3)
Cu4–N7 1.962(5)	Cu12–N23 1.981(5)	Cu21–O20 1.930(3)	Cu28–O28 1.937(3)
Cu5–O4 1.899(4)	Cu13–O12 1.936(4)	Cu21–O21 1.928(3)	Cu28–N54 1.997(4)
Cu5–O5 1.933(4)	Cu13–O13 1.905(3)	Cu21–N40 1.977(4)	Cu28–N55 1.987(4)

Table S32. Selected bond lengths for the minor component (Cu_{9+12+6}, 9%) in UNIT 2 of **9** (Cu1, Cu2B–Cu7B, Cu9, Cu10: Cu_9-ring; Cu11–Cu22: Cu_{12}-ring; Cu23–Cu28: Cu_6-ring; O1_2–O3_2, O1B_2–O3B_2, C1_2–C6_2: $PhPO_3^{2-}$ anion with its PO_3 unit disordered over two positions with 0.89/0.11 occupancy).

P1_2–O1_2 1.509(4)	Cu5B–O5B 1.909(16)	Cu14–O14 1.930(3)	Cu22–O22 1.932(3)
P1_2–O2_2 1.526(4)	Cu5B–N8B 2.005(16)	Cu14–N26 1.968(5)	Cu22–N42 1.988(4)
P1_2–O3_2 1.537(4)	Cu5B–N9B 1.965(16)	Cu14–N27 1.956(5)	Cu22–N43 1.967(4)
P1_2–C1_2 1.811(5)	Cu6B–O5B 1.925(17)	Cu15–O14 1.928(3)	Cu23–O23 1.949(3)
P1B_2–O2B_2 1.480(15)	Cu6B–O6B 1.927(16)	Cu15–O15 1.936(4)	Cu23–O28 1.935(3)
P1B_2–O1B_2 1.540(16)	Cu6B–N10B 1.994(16)	Cu15–N28 1.975(5)	Cu23–O22 2.339(3)
P1B_2–O3B_2 1.546(16)	Cu6B–N11B 1.988(16)	Cu15–N29 1.953(4)	Cu23–N45 1.998(4)
P1B_2–C1_2 1.811(5)	Cu7B–O6B 1.904(15)	Cu16–O15 1.933(3)	Cu23–N56 1.987(4)
C1_2–C2_2 1.395(8)	Cu7B–O7B 1.914(16)	Cu16–O16 1.939(3)	Cu24–O23 1.944(3)
C2_2–C3_2 1.377(9)	Cu7B–N12B 1.997(16)	Cu16–N30 1.965(4)	Cu24–O24 1.922(3)
C3_2–C4_2 1.391(11)	Cu7B–N13B 1.973(16)	Cu16–N31 1.968(4)	Cu24–N46 1.985(4)
C4_2–C5_2 1.353(11)	Cu8B–O7B 1.907(18)	Cu17–O16 1.938(3)	Cu24–N47 1.989(5)
C5_2–C6_2 1.390(9)	Cu8B–O9 1.974(19)	Cu17–O17 1.945(3)	Cu25–O24 1.929(3)
C6_2–C1_2 1.388(7)	Cu8B–N14B 1.994(18)	Cu17–N32 1.969(5)	Cu25–O25 1.942(3)
Cu1–O1 1.901(3)	Cu8B–N17 2.305(15)	Cu17–N33 1.977(4)	Cu25–O14 2.395(3)
Cu1–O10 1.925(3)	Cu10–O9 1.919(3)	Cu18–O17 1.931(3)	Cu25–N48 1.992(5)
Cu1–N1 1.983(5)	Cu10–O10 1.934(3)	Cu18–O18 1.928(3)	Cu25–N49 1.982(4)
Cu1–N20 1.980(4)	Cu10–N18 1.990(5)	Cu18–N34 1.953(4)	Cu26–O25 1.939(3)
Cu2B–O1 1.84(2)	Cu10–N19 1.991(4)	Cu18–N35 1.973(4)	Cu26–O26 1.936(3)
Cu2B–O2B 1.906(18)	Cu11–O11 1.934(3)	Cu19–O18 1.928(3)	Cu26–O16 2.328(3)
Cu2B–N2 2.139(18)	Cu11–O22 1.932(3)	Cu19–O19 1.919(3)	Cu26–N50 1.996(4)
Cu2B–N3B 1.955(18)	Cu11–N21 1.969(4)	Cu19–N36 1.953(4)	Cu26–N51 1.995(4)
Cu3B–O2B 1.931(16)	Cu11–N44 1.986(4)	Cu19–N37 1.966(4)	Cu27–O26 1.934(3)
Cu3B–O3B 1.944(17)	Cu12–O11 1.936(4)	Cu20–O19 1.916(3)	Cu27–O27 1.943(3)
Cu3B–N4B 2.020(16)	Cu12–O12 1.934(3)	Cu20–O20 1.935(3)	Cu27–O18 2.399(4)
Cu3B–N5B 2.025(16)	Cu12–N22 1.967(4)	Cu20–N38 1.979(4)	Cu27–N52 1.986(4)
Cu4B–O3B 1.915(15)	Cu12–N23 1.981(5)	Cu20–N39 1.948(4)	Cu27–N53 1.987(4)
Cu4B–O4B 1.897(14)	Cu13–O12 1.936(4)	Cu21–O20 1.930(3)	Cu28–O27 1.937(3)
Cu4B–O15 2.308(11)	Cu13–O13 1.905(3)	Cu21–O21 1.928(3)	Cu28–O28 1.937(3)
Cu4B–N6B 1.998(16)	Cu13–N24 1.958(5)	Cu21–N40 1.977(4)	Cu28–N54 1.997(4)
Cu4B–N7B 1.967(16)	Cu13–N25 1.981(5)	Cu21–N41 1.959(4)	Cu28–N55 1.987(4)
Cu5B–O4B 1.909(15)	Cu14–O13 1.923(4)	Cu22–O21 1.947(3)	

Table S33. Selected bond lengths for **10** (Cu1–Cu8: Cu_8-ring; Cu9–Cu22: Cu_{14}-ring; Cu23–Cu31: Cu_9-ring; O1_2–O3_2, C1_2–C6_2: $PhPO_3^{2-}$ anion).

P1_2–O1_2 1.519(2)
P1_2–O2_2 1.518(2)
P1_2–O3_2 1.530(2)
P1_2–C1_2 1.837(3)
C1_2–C6_2 1.396(4)
C1_2–C2_2 1.402(4)
C2_2–C3_2 1.394(5)
C3_2–C4_2 1.375(6)
C4_2–C5_2 1.382(6)
C5_2–C6_2 1.391(5)
Cu1–O1 1.934(2)
Cu1–O8 1.921(2)
Cu1–N16 1.983(3)
Cu1–N1 1.997(3)
Cu2–O1 1.927(2)
Cu2–O2 1.951(2)
Cu2–N3 1.985(3)
Cu2–N2 2.002(3)
Cu3–O2 1.945(2)
Cu3–O3 1.929(2)
Cu3–N4 1.985(3)
Cu3–N5 2.078(12)
Cu3–N5B 1.933(13)
Cu3–O12 2.438(2)
Cu4–O4 1.915(2)
Cu4–O3 1.931(2)
Cu4–N6 1.993(14)
Cu4–N6B 2.002(14)
Cu4–N7 1.976(3)
Cu5–O4 1.896(2)
Cu5–O5 1.926(2)
Cu5–N8 1.965(3)
Cu5–N9 1.966(3)
Cu6–O6 1.920(2)
Cu6–O5 1.957(2)
Cu6–N10 1.975(3)
Cu6–N11B 1.98(2)
Cu6–N11 1.992(8)
Cu7–O6 1.918(2)
Cu7–O7 1.942(2)
Cu7–N12 1.992(7)
Cu7–N12B 1.95(2)
Cu7–N13 1.988(3)
Cu8–O8 1.896(2)
Cu8–O7 1.921(2)
Cu8–N15 1.974(3)
Cu8–N14 1.994(3)
Cu9–O9 1.921(2)
Cu9–O22 1.922(2)
Cu9–N17 1.958(3)
Cu9–N44 1.960(3)
Cu10–O9 1.920(2)
Cu10–O10 1.931(2)
Cu10–N19 1.956(3)
Cu10–N18 1.978(3)
Cu11–O10 1.927(2)
Cu11–O11 1.929(2)
Cu11–N21 1.963(3)
Cu11–N20 1.965(3)
Cu12–O11 1.925(2)
Cu12–O12 1.930(2)
Cu12–N22 1.956(3)
Cu12–N23 1.974(3)
Cu13–O13 1.910(2)
Cu13–O12 1.932(2)
Cu13–N24 1.947(3)
Cu13–N25 1.981(3)
Cu14–O14 1.911(2)
Cu14–O13 1.927(2)
Cu14–N26 1.966(3)
Cu14–N27 1.975(3)
Cu15–O14 1.911(2)
Cu15–O15 1.914(2)
Cu15–N28 1.965(3)
Cu15–N29 1.981(3)
Cu16–O16 1.906(2)
Cu16–O15 1.912(2)
Cu16–N30 1.952(3)
Cu16–N31 1.961(3)
Cu17–O16 1.916(2)
Cu17–O17 1.921(2)
Cu17–N33 1.958(3)
Cu17–N32 1.964(3)
Cu18–O18 1.910(2)
Cu18–O17 1.919(2)
Cu18–N34 1.955(3)
Cu18–N35 1.963(3)
Cu19–O18 1.904(2)
Cu19–O19 1.933(2)
Cu19–N37 1.958(3)
Cu19–N36 1.963(3)
Cu20–O20 1.904(2)
Cu20–O19 1.926(2)
Cu20–N38 1.959(3)
Cu20–N39 1.966(3)
Cu21–O20 1.905(2)
Cu21–O21 1.926(3)
Cu21–N41 1.965(3)
Cu21–N40 1.973(3)
Cu22–O22 1.907(2)
Cu22–O21 1.923(2)
Cu22–N42 1.963(3)
Cu22–N43 1.972(3)
Cu23–O31 1.905(2)
Cu23–O23 1.912(2)
Cu23–N62 1.952(3)
Cu23–N45 1.977(3)
Cu24–O24 1.940(2)
Cu24–O23 1.943(2)
Cu24–N47 2.002(3)
Cu24–N46 2.011(3)
Cu24–O9 2.349(2)
Cu25–O25 1.908(2)
Cu25–O24 1.917(2)
Cu25–N49 1.959(3)
Cu25–N48 1.983(3)
Cu26–O25 1.900(2)
Cu26–O26 1.915(2)
Cu26–N50 1.965(3)
Cu26–N51 1.981(3)
Cu27–O26 1.936(2)
Cu27–O27 1.945(2)
Cu27–N52 2.002(3)
Cu27–N53 2.011(3)
Cu27–O13 2.341(2)
Cu28–O28 1.899(2)
Cu28–O27 1.906(2)
Cu28–N55 1.954(3)
Cu28–N54 1.971(3)
Cu29–O28 1.921(2)
Cu29–O29 1.925(2)
Cu29–N57 1.968(3)
Cu29–N56 1.988(3)
Cu30–O29 1.932(2)
Cu30–O30 1.937(2)
Cu30–N59 2.002(3)
Cu30–N58 2.023(3)
Cu31–O31 1.919(2)
Cu31–O30 1.919(2)
Cu31–N61 1.980(3)
Cu31–N60 1.981(3)

Table S34. Selected bond lengths for **11** (Cu1–Cu6: Cu_6-ring; Cu7–Cu18: Cu_{12}-ring; Cu19–Cu27: Cu_9-ring; O1_1–O3_1, C1_1, C2_1: incarcerated $EtPO_3^{2-}$ anion; O1_2–O3_2, C1_2, C2_2: bridging $EtPO_3^{2-}$ anion).

P1_1–O2_1 1.5273(17)
P1_1–O3_1 1.5277(17)
P1_1–O1_1 1.5300(17)
P1_1–C1_1 1.809(2)
C1_1–C2_1 1.523(3)
P1_2–O1_2 1.5086(18)
P1_2–O2_2 1.5657(18)
P1_2–O3_2 1.5091(19)
P1_2–C1_2 1.797(3)
C1_2–C2_2 1.536(5)
Cu1–O1 1.9124(18)
Cu1–O6 1.9430(17)
Cu1–N1 2.003(2)
Cu1–N12 1.976(2)
Cu2–O1 1.9246(18)
Cu2–O2 1.9419(17)
Cu2–N2 2.001(2)
Cu2–N3 1.979(2)
Cu3–O2 1.9432(18)
Cu3–O3 1.9339(17)
Cu3–O10 2.3511(17)
Cu3–N4 1.996(2)
Cu3–N5 2.029(2)
Cu4–O3 1.9191(17)
Cu4–O4 1.9323(17)
Cu4–N6 1.977(2)
Cu4–N7 1.987(2)
Cu5–O4 1.9427(17)
Cu5–O5 1.9333(17)
Cu5–O14 2.3733(17)
Cu5–N9 1.993(2)
Cu5–N8 2.024(2)
Cu6–O5 1.9302(17)
Cu6–O6 1.9444(18)
Cu6–O16 2.3904(18)
Cu6–N10 1.993(2)
Cu6–N11 2.023(2)
Cu7–O7 1.9002(19)
Cu7–O18 1.9340(18)
Cu7–N13 1.975(2)
Cu7–N36 1.978(2)
Cu8–O7 1.9000(19)
Cu8–O8 1.9434(18)
Cu8–N14 1.980(2)
Cu8–N15 1.974(2)
Cu9–O8 1.9277(19)
Cu9–O9 1.9215(17)
Cu9–N16 1.958(2)
Cu9–N17 1.973(3)
Cu10–O9 1.9255(18)
Cu10–O10 1.9200(17)
Cu10–N18 1.959(2)
Cu10–N19 1.959(2)
Cu11–O10 1.9243(17)
Cu11–O11 1.9322(18)
Cu11–N20 1.971(2)
Cu11–N21 1.953(2)
Cu12–O11 1.9272(17)
Cu12–O12 1.9453(18)
Cu12–N22 1.973(2)
Cu12–N23 1.981(2)
Cu13–O12 1.9420(17)
Cu13–O13 1.9147(18)
Cu13–N24 1.955(2)
Cu13–N25 1.979(2)
Cu14–O13 1.9258(18)
Cu14–O14 1.9291(18)
Cu14–N26 1.971(2)
Cu14–N27 1.969(2)
Cu15–O14 1.9257(18)
Cu15–O15 1.9336(18)
Cu15–N28 1.979(2)
Cu15–N29 1.956(2)
Cu16–O15 1.9328(18)
Cu16–O16 1.9222(17)
Cu16–N30 1.951(2)
Cu16–N31 1.979(2)
Cu17–O16 1.9371(18)
Cu17–O17 1.9257(18)
Cu17–N32 1.972(2)
Cu17–N33 1.973(2)
Cu18–O17 1.9111(18)
Cu18–O18 1.9347(19)
Cu18–N34 1.969(3)
Cu18–N35 1.965(2)
Cu19–O19 1.8744(18)
Cu19–O27 1.9054(17)
Cu19–O2_2 1.9605(17)
Cu19–O2_2′ 1.9600(17)
Cu20–O19 1.9025(18)
Cu20–O20 1.9210(17)
Cu20–O3_2′ 1.9246(18)
Cu20–N37 1.963(2)
Cu21–O20 1.9443(17)
Cu21–O21 1.9340(16)
Cu21–N38 1.966(2)
Cu21–N39 1.995(2)
Cu22–O21 1.9472(16)
Cu22–O22 1.9104(18)
Cu22–N40 1.977(2)
Cu22–N41 1.987(2)
Cu23–O22 1.9024(17)
Cu23–O23 1.9112(18)
Cu23–N42 1.964(2)
Cu23–N43 1.967(2)
Cu24–O23 1.9272(17)
Cu24–O24 1.9399(18)
Cu24–O13 2.4049(18)
Cu24–N44 1.996(2)
Cu24–N45 2.003(2)
Cu25–O24 1.9349(18)
Cu25–O25 1.9070(18)
Cu25–N46 1.968(2)
Cu25–N47 1.980(2)
Cu26–O25 1.9162(18)
Cu26–O26 1.9345(17)
Cu26–N48 1.985(2)
Cu26–N49 1.958(2)
Cu27–O26 1.9294(18)
Cu27–O27 1.9754(18)
Cu27–O17 2.3354(18)
Cu27–O1_2′ 1.9475(17)
Cu27–N50 1.982(2)

Table S35. Selected bond lengths for **12** (Cu1_1–Cu9_1 and Cu1_2–Cu9_2: Cu$_9$-rings; Cu10_1–Cu21_1 and Cu10_2–Cu21_2: Cu$_{12}$-rings; Cu22_1–Cu27_1 and Cu22_2–Cu27_2: Cu$_6$-rings; O1_5–O3_5, C1_5–C3_5 and O1_6–O3_6, C1_6–C3_6: incarcerated ^{n}PrPO$_3^{2-}$ anions; O1_3–O3_3, C1_3–C3_3 and O1_4–O3_4, C1_4–C3_4: bridging ^{n}PrPO$_3^{2-}$ anions).

P1_3–O1_3 1.576(2)	Cu5_1–O14_1 2.375(2)	Cu13_1–N21_1 1.975(3)	Cu21_1–N37_1 1.960(3)
P1_3–O2_3 1.504(2)	Cu6_1–O6_1 1.900(2)	Cu13_1–N20_1 1.977(3)	Cu21_1–N36_1 1.988(3)
P1_3–O3_3 1.504(2)	Cu6_1–O5_1 1.933(2)	Cu14_1–O14_1 1.916(2)	Cu22_1–O27_1 1.923(2)
P1_3–C1_3 1.797(4)	Cu6_1–N8_1 1.971(3)	Cu14_1–O13_1 1.938(2)	Cu22_1–O22_1 1.940(2)
C1_3–C2_3 1.503(5)	Cu6_1–N9_1 1.975(3)	Cu14_1–N22_1 1.960(3)	Cu22_1–N39_1 1.991(3)
C2_3–C3_3 1.489(6)	Cu7_1–O6_1 1.900(2)	Cu14_1–N23_1 1.968(3)	Cu22_1–N50_1 1.996(3)
P1_5–O1_5 1.526(2)	Cu7_1–O7_1 1.928(2)	Cu15_1–O14_1 1.923(2)	Cu22_1–N50B_1 1.98(2)
P1_5–O2_5 1.522(2)	Cu7_1–N11_1 1.957(3)	Cu15_1–O15_1 1.926(2)	Cu23_1–O23_1 1.931(2)
P1_5–O3_5 1.522(2)	Cu7_1–N10_1 1.977(3)	Cu15_1–N25_1 1.963(3)	Cu23_1–O22_1 1.941(2)
P1_5–C1_5 1.818(3)	Cu8_1–O7_1 1.926(2)	Cu15_1–N24_1 1.968(3)	Cu23_1–N40_1 2.007(3)
C1_5–C2_5 1.527(5)	Cu8_1–O8_1 1.974(2)	Cu16_1–O16_1 1.922(2)	Cu23_1–N41_1 2.027(3)
C2_5–C3_5 1.511(5)	Cu8_1–N13_1 1.992(3)	Cu16_1–O15_1 1.930(2)	Cu23_1–O11_1 2.364(2)
Cu1_1–O9_1 1.875(2)	Cu8_1–N12_1 2.037(3)	Cu16_1–N27_1 1.968(3)	Cu24_1–O23_1 1.919(2)
Cu1_1–O1_1 1.899(2)	Cu8_1–O18_1 2.352(2)	Cu16_1–N26_1 1.970(3)	Cu24_1–O24_1 1.941(2)
Cu1_1–O1_3 1.969(2)	Cu9_1–O9_1 1.894(2)	Cu17_1–O16_1 1.925(2)	Cu24_1–N43_1 1.984(3)
Cu1_1–O1_4 1.982(2)	Cu9_1–O8_1 1.917(2)	Cu17_1–O17_1 1.931(2)	Cu24_1–N42_1 1.997(3)
Cu2_1–O2_1 1.931(2)	Cu9_1–O3_4 1.919(2)	Cu17_1–N28_1 1.965(3)	Cu24_1–O13_1 2.430(2)
Cu2_1–O2_3 1.936(2)	Cu9_1–N14_1 1.959(3)	Cu17_1–N29_1 1.972(3)	Cu25_1–O25_1 1.919(2)
Cu2_1–O1_1 1.962(2)	Cu10_1–O10_1 1.920(2)	Cu18_1–O17_1 1.914(2)	Cu25_1–O24_1 1.947(2)
Cu2_1–N1_1 1.984(3)	Cu10_1–O21_1 1.941(2)	Cu18_1–O18_1 1.929(2)	Cu25_1–N44_1 1.999(3)
Cu2_1–O10_1 2.365(2)	Cu10_1–N15_1 1.973(3)	Cu18_1–N31_1 1.960(3)	Cu25_1–N45_1 2.011(3)
Cu3_1–O3_1 1.918(2)	Cu10_1–N38_1 1.985(3)	Cu18_1–N30_1 1.963(3)	Cu25_1–O15_1 2.377(2)
Cu3_1–O2_1 1.940(2)	Cu11_1–O10_1 1.927(2)	Cu19_1–O19_1 1.918(2)	Cu26_1–O25_1 1.923(2)
Cu3_1–N2_1 1.972(3)	Cu11_1–O11_1 1.934(2)	Cu19_1–O18_1 1.919(2)	Cu26_1–O26_1 1.941(2)
Cu3_1–N3_1 1.988(3)	Cu11_1–N17_1 1.980(3)	Cu19_1–N33_1 1.961(3)	Cu26_1–N47_1 1.995(3)
Cu4_1–O3_1 1.902(2)	Cu11_1–N16_1 1.981(3)	Cu19_1–N32_1 1.968(3)	Cu26_1–N46_1 1.996(3)
Cu4_1–O4_1 1.923(2)	Cu12_1–O11_1 1.926(2)	Cu20_1–O20_1 1.906(2)	Cu26_1–O17_1 2.394(2)
Cu4_1–N5_1 1.968(3)	Cu12_1–O12_1 1.932(3)	Cu20_1–O19_1 1.930(2)	Cu27_1–O27_1 1.919(2)
Cu4_1–N4_1 1.989(3)	Cu12_1–N19_1 1.971(3)	Cu20_1–N35_1 1.970(3)	Cu27_1–O26_1 1.945(2)
Cu5_1–O4_1 1.930(2)	Cu12_1–N18_1 1.979(3)	Cu20_1–N34_1 1.983(3)	Cu27_1–N48_1 2.009(3)
Cu5_1–O5_1 1.943(2)	Cu13_1–O12_1 1.923(2)	Cu21_1–O20_1 1.896(3)	Cu27_1–N49_1 2.005(3)
Cu5_1–N7_1 2.014(3)	Cu13_1–O13_1 1.935(2)	Cu21_1–O21_1 1.937(3)	Cu27_1–N49B_1 2.08(2)
Cu5_1–N6_1 2.030(3)			

Table S35. (continuation)

P1_4–O1_4 1.560(2)
P1_4–O2_4 1.498(3)
P1_4–O3_4 1.494(3
P1_4–C1B_4 1.772(16)
P1_4–C1_4 1.803(6)
C1_4–C2_4 1.505(9)
C2_4–C3_4 1.508(15)
C1B_4–C2B_4 1.487(16)
C2B_4–C3B_4 1.494(19)
P1_6–O1_6 1.521(2)
P1_6–O2_6 1.516(2)
P1_6–O3_6 1.518(2)
P1_6–C1_6 1.810(3)
C1_6–C2_6 1.520(4)
C2_6–C3_6 1.526(5)
Cu1_2–O9_2 1.879(2)
Cu1_2–O1_2 1.907(2)
Cu1_2–O1_3 1.961(2)
Cu1_2–O1_4 1.972(2)
Cu2_2–O3_3 1.935(2)
Cu2_2–O2_2 1.948(2)
Cu2_2–O1_2 1.960(2)
Cu2_2–N1_2 1.990(3)
Cu2_2–O10_2 2.381(2)
Cu3_2–O3_2 1.913(2)
Cu3_2–O2_2 1.949(2)
Cu3_2–N2_2 1.957(3)
Cu3_2–N3_2 2.002(3)
Cu4_2–O3_2 1.911(2)
Cu4_2–O4_2 1.914(2)
Cu4_2–N5_2 1.971(3)
Cu4_2–N4_2 1.975(3)
Cu5_2–O4_2 1.927(2)
Cu5_2–O5_2 1.951(2)
Cu5_2–N7_2 2.009(3)
Cu5_2–N6_2 2.020(3)
Cu5_2–O14_2 2.388(2)
Cu6_2–O6_2 1.897(2)
Cu6_2–O5_2 1.928(2)
Cu6_2–N9_2 1.966(3)
Cu6_2–N8_2 1.968(3)
Cu7_2–O6_2 1.901(2)
Cu7_2–O7_2 1.916(2)
Cu7_2–N11_2 1.971(3)
Cu7_2–N10_2 1.978(3)
Cu8_2–O7_2 1.911(2)
Cu8_2–O8_2 1.977(2)
Cu8_2–N12_2 2.050(3)
Cu8_2–N13_2 1.977(3)
Cu8_2–O18_2 2.329(2)
Cu9_2–O9_2 1.889(2)
Cu9_2–O2_4 1.909(3)
Cu9_2–O8_2 1.917(2)
Cu9_2–N14_2 1.958(3)
Cu10_2–O10_2 1.905(2)
Cu10_2–O21_2 1.939(2)
Cu10_2–N38_2 1.960(3)
Cu10_2–N15_2 1.972(3)
Cu11_2–O10_2 1.915(2)
Cu11_2–O11_2 1.934(2)
Cu11_2–N17_2 1.960(3)
Cu11_2–N16_2 1.969(3)
Cu12_2–O11_2 1.931(2)
Cu12_2–O12_2 1.933(2)
Cu12_2–N18_2 1.967(3)
Cu12_2–N19_2 1.972(3)
Cu13_2–O12_2 1.920(2)
Cu13_2–O13_2 1.933(2)
Cu13_2–N21_2 1.962(3)
Cu13_2–N20_2 1.964(3)
Cu14_2–O14_2 1.919(2)
Cu14_2–O13_2 1.928(2)
Cu14_2–N23_2 1.967(3)
Cu14_2–N22_2 1.970(3)
Cu15_2–O14_2 1.917(2)
Cu15_2–O15_2 1.932(2)
Cu15_2–N25_2 1.960(3)
Cu15_2–N24_2 1.979(3)
Cu16_2–O16_2 1.921(2)
Cu16_2–O15_2 1.930(2)
Cu16_2–N27_2 1.964(3)
Cu16_2–N26_2 1.977(3)
Cu17_2–O16_2 1.923(2)
Cu17_2–O17_2 1.930(2)
Cu17_2–N28_2 1.971(3)
Cu17_2–N29_2 1.972(3)
Cu18_2–O18_2 1.920(2)
Cu18_2–O17_2 1.932(2)
Cu18_2–N30_2 1.949(3)
Cu18_2–N31_2 1.976(3)
Cu19_2–O19_2 1.922(2)
Cu19_2–O18_2 1.922(2)
Cu19_2–N33_2 1.952(3)
Cu19_2–N32_2 1.963(3)
Cu20_2–O20_2 1.905(2)
Cu20_2–O19_2 1.926(2)
Cu20_2–N35_2 1.971(3)
Cu20_2–N34_2 1.975(3)
Cu21_2–O20_2 1.899(2)
Cu21_2–O21_2 1.927(2)
Cu21_2–N36_2 1.967(3)
Cu21_2–N37_2 1.970(3)
Cu22_2–O27_2 1.927(2)
Cu22_2–O22_2 1.930(2)
Cu22_2–N50_2 1.980(3)
Cu22_2–N39_2 1.998(3)
Cu23_2–O23_2 1.933(2)
Cu23_2–O22_2 1.940(2)
Cu23_2–N40_2 1.997(3)
Cu23_2–N41_2 2.014(3)
Cu23_2–O11_2 2.350(2)
Cu24_2–O23_2 1.926(2)
Cu24_2–O24_2 1.937(2)
Cu24_2–N43_2 1.987(3)
Cu24_2–N42_2 1.990(3)
Cu24_2–O13_2 2.407(2)
Cu25_2–O25_2 1.914(2)
Cu25_2–O24_2 1.944(2)
Cu25_2–N44_2 1.999(3)
Cu25_2–N45_2 2.017(3)
Cu25_2–O15_2 2.365(2)
Cu26_2–O25_2 1.924(2)
Cu26_2–O26_2 1.935(2)
Cu26_2–N47_2 1.988(3)
Cu26_2–N46_2 2.000(3)
Cu26_2–O17_2 2.398(2)
Cu27_2–O27_2 1.927(2)
Cu27_2–O26_2 1.934(2)
Cu27_2–N49_2 1.988(3)
Cu27_2–N48_2 2.018(3)

Table S36. Hydrogen bonding data for **1** (O1_2–O9_2: Cu_9-ring; O10_2–O21_2: Cu_{12}-ring; O22_2–O27_2: Cu_6-ring; O1_1–O3_1: $MePO_3^{2-}$ anion).

D*—H···*A	***D*—H (Å)**	**H···*A* (Å)**	***D*···*A* (Å)**	***D*—H···*A* (°)**
O1_2—H1O_2···O2_1	0.84(2)	1.97(3)	2.773(4)	161(6)
O2_2—H2O_2···O2_1	0.82(2)	2.23(3)	2.995(3)	156(5)
O3_2—H3O_2···O3_1	0.82(2)	2.03(2)	2.839(3)	172(5)
O4_2—H4O_2···O3_1	0.840(19)	1.98(2)	2.805(3)	166(5)
O5_2—H5O_2···O3_1	0.823(19)	2.13(2)	2.918(4)	161(5)
O6_2—H6O_2···O1_1	0.82(2)	1.98(2)	2.785(4)	167(5)
O7_2—H7O_2···O1_1	0.83(2)	2.00(2)	2.828(3)	174(5)
O8_2—H8O_2···O1_1	0.849(19)	2.18(2)	2.994(4)	160(4)
O9_2—H9O_2···O2_1	0.82(2)	2.00(2)	2.798(4)	165(5)
O10_2—H10O_2···O22_2	0.83(2)	1.93(2)	2.758(4)	175(6)
O11_2—H11O_2···O2_2	0.83(2)	1.94(2)	2.751(4)	166(6)
O12_2—H12O_2···O23_2	0.84(2)	1.96(2)	2.783(3)	167(5)
O13_2—H13O_2···O3_2	0.82(2)	2.00(3)	2.796(4)	164(6)
O14_2—H14O_2···O24_2	0.84(2)	1.94(2)	2.775(4)	172(5)
O15_2—H15O_2···O5_2	0.84(2)	1.91(2)	2.746(4)	174(5)
O16_2—H16O_2···O25_2	0.825(19)	1.96(2)	2.785(3)	176(5)
O17_2—H17O_2···O6_2	0.83(2)	2.09(2)	2.901(4)	168(5)
O18_2—H18O_2···O26_2	0.836(19)	1.92(2)	2.756(3)	174(5)
O19_2—H19O_2···O8_2	0.83(2)	1.95(2)	2.777(3)	178(5)
O20_2—H20O_2···O27_2	0.831(19)	1.94(2)	2.760(3)	171(5)
O21_2—H21O_2···O9_2	0.81(2)	2.10(2)	2.888(4)	164(5)
O22_2—H22O_2···O2_1	0.828(19)	1.90(2)	2.713(3)	166(5)
O23_2—H23O_2···O3_1	0.83(2)	2.51(3)	3.239(3)	147(4)
O24_2—H24O_2···O3_1	0.83(2)	1.95(3)	2.739(3)	159(5)
O25_2—H25O_2···O1_1	0.815(19)	2.43(3)	3.184(4)	155(4)
O26_2—H26O_2···O1_1	0.83(2)	1.92(2)	2.730(3)	166(5)
O27_2—H27O_2···O2_1	0.834(19)	2.40(3)	3.135(3)	147(4)
O1_2—H1O_2···O2_1	0.84(2)	1.97(3)	2.773(4)	161(6)

Table S37. Hydrogen bonding data for UNIT 1 of **2** (O1_3–O7_3: Cu_7-ring; O8_3–O20_3: Cu_{13}-ring; O21_3–O29_3: Cu_9-ring; O1_1–O3_1: $MePO_3^{2-}$ anion).

D*—H···*A	***D*—H (Å)**	**H···*A* (Å)**	***D*···*A* (Å)**	***D*—H···*A* (°)**
O1_3—H1O_3···O2_1	0.85(2)	2.10(5)	2.909(6)	160(7)
O2_3—H2O_3···O1_1	0.84(2)	1.96(6)	2.772(6)	162(8)
O3_3—H3O_3···O1_1	0.83(2)	2.20(7)	2.804(6)	129(7)
O5_3—H5O_3···O3_1	0.84(2)	2.04(8)	2.812(6)	153(6)
O6_3—H6O_3···O3_1	0.83(2)	2.15(6)	2.901(7)	150(7)
O7_3—H7O_3···O2_1	0.84(2)	2.03(8)	2.818(7)	158(5)
O8_3—H8O_3···O1_3	0.84(2)	2.12(6)	2.940(6)	164(9)
O10_3—H10O_3···O2_3	0.85(2)	1.98(8)	2.821(7)	172(6)
O11_3—H11O_3···O23_3	0.84(2)	1.87(5)	2.703(6)	169(6)
O12_3—H12O_3···O3_3	0.84(2)	2.03(7)	2.846(7)	165(7)
O13_3—H13O_3···O4_3	0.844(18)	2.15(6)	2.919(7)	152(5)
O14_3—H14O_3···O25_3	0.83(2)	2.07(6)	2.804(6)	150(6)
O15_3—H15O_3···O5_3	0.84(2)	1.92(4)	2.746(6)	169(8)
O16_3—H16O_3···O26_3	0.83(2)	2.01(8)	2.747(7)	150(7)
O17_3—H17O_3···O6_3	0.84(2)	2.05(5)	2.855(6)	159(6)
O18_3—H18O_3···O28_3	0.83(2)	1.98(8)	2.798(7)	172(7)
O19_3—H19O_3···O7_3	0.83(2)	2.10(6)	2.876(5)	154(7)
O20_3—H20O_3···O29_3	0.84(2)	1.94(8)	2.728(7)	156(6)
O23_3—H23O_3···O1_1	0.83(2)	1.94(5)	2.724(7)	158(7)
O24_3—H24O_3···O1_1	0.83(2)	2.25(7)	3.053(7)	163(6)
O25_3—H25O_3···O3_1	0.83(2)	2.16(5)	2.935(6)	154(6)
O26_3—H26O_3···O3_1	0.84(2)	1.92(5)	2.731(6)	164(6)
O28_3—H28O_3···O2_1	0.83(2)	2.20(4)	2.959(6)	152(7)
O29_3—H29O_3···O2_1	0.84(2)	1.92(6)	2.756(6)	172(6)

Table S38. Hydrogen bonding data for UNIT 2 of **2** (O1_4–O7_4: Cu_7-ring; O8_4–O20_4: Cu_{13}-ring; O21_4–O29_4: Cu_9-ring; O1_2–O3_2: $MePO_3^{2-}$ anion).

D*—H···*A	***D*—H (Å)**	**H···*A* (Å)**	***D*···*A* (Å)**	***D*—H···*A* (°)**
O1_4—H1O_4···O1_2	0.84(2)	2.12(5)	2.956(6)	167(7)
O2_4—H2O_4···O2_2	0.83(2)	2.02(6)	2.743(6)	144(6)
O3_4—H3O_4···O2_2	0.83(2)	2.01(4)	2.822(6)	166(7)
O5_4—H5O_4···O3_2	0.84(2)	1.98(6)	2.779(7)	159(7)
O6_4—H6O_4···O3_2	0.85(2)	2.12(7)	2.939(8)	160(7)
O7_4—H7O_4···O1_2	0.84(2)	1.96(8)	2.775(7)	162(6)
O8_4—H8O_4···O1_4	0.84(2)	2.11(5)	2.933(6)	166(7)
O10_4—H10O_4···O2_4	0.84(2)	1.97(7)	2.814(7)	170(6)
O11_4—H11O_4···O23_4	0.83(2)	2.11(5)	2.707(5)	129(5)
O12_4—H12O_4···O3_4	0.83(2)	2.09(6)	2.886(7)	161(6)
O13_4—H13O_4···O4_4	0.84(2)	2.01(7)	2.845(7)	170(6)
O14_4—H14O_4···O25_4	0.83(2)	2.07(8)	2.793(6)	145(7)
O15_4—H15O_4···O5_4	0.82(2)	1.94(5)	2.764(6)	173(9)
O16_4—H16O_4···O26_4	0.83(2)	1.94(8)	2.760(7)	171(7)
O17_4—H17O_4···O6_4	0.83(2)	2.04(5)	2.872(6)	177(7)
O18_4—H18O_4···O28_4	0.84(2)	1.94(8)	2.784(7)	176(7)
O19_4—H19O_4···O7_4	0.84(2)	2.08(6)	2.893(5)	165(5)
O20_4—H20O_4···O29_4	0.83(2)	1.91(7)	2.729(7)	169(6)
O21_4—H21O_4···O1_2	0.83(2)	2.44(8)	3.237(7)	162(10)
O23_4—H23O_4···O2_2	0.83(2)	1.91(4)	2.693(7)	155(8)
O24_4—H24O_4···O2_2	0.84(2)	2.36(7)	3.160(7)	158(6)
O25_4—H25O_4···O3_2	0.83(2)	2.11(6)	2.925(7)	167(7)
O26_4—H26O_4···O3_2	0.86(2)	1.90(6)	2.740(6)	166(7)
O28_4—H28O_4···O1_2	0.84(2)	2.08(3)	2.912(6)	170(7)
O29_4—H29O_4···O1_2	0.83(2)	1.96(5)	2.784(6)	172(8)

Table S39. Hydrogen bonding data for **3** (O1_1–O8_1: Cu_8-ring; O9_1–O22_1: Cu_{14}-ring; O23_1–O31_1: Cu_9-ring; O1_2–O3_2: $MePO_3^{2-}$ anion).

D*—H···*A	***D*—H (Å)**	**H···*A* (Å)**	***D*···*A* (Å)**	***D*—H···*A* (°)**
O1_1—H1O_1···O2_2	0.84(2)	2.04(3)	2.866(8)	171(10)
O3_1—H3O_1···O3_2	0.84(2)	2.02(3)	2.845(7)	170(9)
O4_1—H4O_1···O3_2	0.83(2)	2.17(4)	2.961(8)	161(10)
O5_1—H5O_1···O1_2	0.84(2)	2.25(3)	3.068(8)	165(10)
O6_1—H6O_1···O1_2	0.84(2)	2.08(4)	2.892(8)	164(10)
O7_1—H7O_1···O1_2	0.84(2)	2.31(4)	3.116(9)	161(10)
O8_1—H8O_1···O2_2	0.83(2)	2.32(6)	3.068(9)	150(11)
O9_1—H9O_1···O1_1	0.83(2)	1.98(3)	2.813(8)	174(11)
O10_1—H10O_1···O23_1	0.84(2)	1.97(3)	2.798(8)	169(10)
O11_1—H11O_1···O2_1	0.84(2)	2.02(4)	2.826(8)	161(10)
O12_1—H12O_1···O25_1	0.84(2)	2.00(3)	2.817(8)	165(10)
O13_1—H13O_1···O3_1	0.83(2)	1.98(4)	2.772(8)	159(10)
O14_1—H14O_1···O26_1	0.84(2)	1.95(2)	2.787(7)	174(2)
O15_1—H15O_1···O4_1	0.84(2)	2.62(9)	3.168(8)	124(9)
O16_1—H16O_1···O5_1	0.84(2)	2.06(3)	2.886(8)	170(11)
O17_1—H17O_1···O28_1	0.843(18)	2.103(18)	2.921(8)	164(7)
O17_1—H17O_1···N55_1	0.843(18)	2.69(8)	3.279(10)	128(8)
O18_1—H18O_1···O6_1	0.847(18)	2.013(18)	2.843(8)	166(8)
O19_1—H19O_1···O29_1	0.837(18)	2.108(18)	2.920(8)	163(7)
O20_1—H20O_1···O7_1	0.84(2)	2.13(5)	2.912(8)	155(11)
O22_1—H22O_1···O31_1	0.84(2)	1.95(3)	2.768(9)	167(11)
O23_1—H23O_1···O2_2	0.84(2)	2.14(2)	2.980(8)	179(12)
O25_1—H25O_1···O3_2	0.879(17)	2.143(17)	3.010(8)	169(8)
O26_1—H26O_1···O3_2	0.84(2)	2.00(3)	2.814(8)	166(10)
O27_1—H27O_1···O3_2	0.83(2)	2.62(6)	3.330(9)	144(9)
O28_1—H28O_1···O1_2	0.83(2)	2.20(5)	2.959(8)	151(10)
O29_1—H29O_1···O1_2	0.84(2)	2.14(3)	2.968(7)	169(10)
O31_1—H31O_1···O2_2	0.83(2)	2.19(8)	2.851(8)	137(10)

Table S40. Hydrogen bonding data for **4** (O1_2–O8_2: Cu_8-ring; O9_2–O22_2: Cu_{14}-ring; O23_2–O31_2: Cu_9-ring; O1_1–O3_1: $EtPO_3^{2-}$ anion).

D*—H···*A	***D*—H (Å)**	**H···*A* (Å)**	***D*···*A* (Å)**	***D*—H···*A* (°)**
O2_2—H2O_2···O2_1	0.84(2)	2.07(5)	2.854(9)	155(9)
O3_2—H3O_2···O2_1	0.84(2)	2.18(9)	2.931(9)	150(11)
O5_2—H5O_2···O1_1	0.83(2)	2.30(7)	3.121(12)	178(11)
O6_2—H6O_2···O1_1	0.874(17)	2.24(12)	3.099(16)	168(8)
O7_2—H7O_2···O3_1	0.83(2)	2.41(15)	3.151(17)	147(8)
O8_2—H8O_2···O3_1	0.84(2)	1.99(8)	2.825(12)	178(14)
O9_2—H9O_2···O31_2	0.83(2)	1.98(10)	2.808(9)	173(8)
O10_2—H10O_2···O1_2	0.845(19)	2.01(4)	2.828(8)	161(9)
O11_2—H11O_2···O24_2	0.836(17)	2.00(8)	2.826(9)	169(12)
O12_2—H12O_2···O2_2	0.83(2)	1.96(12)	2.792(11)	177(8)
O13_2—H13O_2···O25_2	0.84(2)	1.93(7)	2.763(7)	167(11)
O15_2—H15O_2···O4_2	0.84(2)	2.07(9)	2.904(11)	171(14)
O16_2—H16O_2···O27_2	0.83(2)	2.21(4)	3.011(9)	163(11)
O17_2—H17O_2···O5_2	0.83(2)	2.04(9)	2.802(9)	153(8)
O18_2—H18O_2···O28_2	0.83(2)	2.16(13)	2.963(13)	168(8)
O19_2—H19O_2···O6_2	0.849(17)	2.01(2)	2.853(8)	169(9)
O21_2—H21O_2···O30_2	0.84(2)	2.05(12)	2.843(12)	156(9)
O22_2—H22O_2···O8_2	0.833(18)	2.00(6)	2.814(7)	164(8)
O24_2—H24O_2···O2_1	0.84(2)	2.31(12)	3.095(13)	156(8)
O25_2—H25O_2···O2_1	0.85(2)	2.02(9)	2.768(13)	146(8)
O27_2—H27O_2···O1_1	0.840(19)	2.21(5)	3.014(13)	159(7)
O28_2—H28O_2···O1_1	0.83(2)	2.11(9)	2.842(11)	147(8)
O30_2—H30O_2···O3_1	0.83(2)	2.07(7)	2.896(10)	169(9)
O31_2—H31O_2···O3_1	0.83(2)	2.23(7)	2.987(12)	150(10)

Table S41. Hydrogen bonding data for the major unit (Cu_{9+14+8}, 95%) of **5** (O1_1–O9_1: Cu_9-ring; O10_1–O23_1: Cu_{14}-ring; O24_1–O31_1: Cu_8-ring; O1_2–O3_2, O1C_2–O3C_2: $EtPO_3^{2-}$ anions with occupancy ratio 0.62/0.33).

D*—H···*A	***D*—H (Å)**	**H···*A* (Å)**	***D*···*A* (Å)**	***D*—H···*A* (°)**
O1_1—H1O_1···O3C_2	0.83(2)	2.04(5)	2.842(14)	166(8)
O2_1—H2O_1···O3_2	0.84(2)	2.00(6)	2.830(9)	169(7)
O2_1—H2O_1···O3C_2	0.84(2)	2.15(7)	2.969(17)	166(8)
O4_1—H4O_1···O1_2	0.84(2)	2.12(4)	2.961(11)	176(8)
O4_1—H4O_1···O1C_2	0.84(2)	1.93(3)	2.751(17)	165(6)
O5_1—H5O_1···O1_2	0.82(2)	2.03(4)	2.826(8)	161(8)
O5_1—H5O_1···O1C_2	0.82(2)	2.37(4)	3.132(15)	153(6)
O6_1—H6O_1···O2C_2	0.83(2)	2.43(5)	3.167(15)	149(8)
O7_1—H7O_1···O2_2	0.83(2)	2.14(8)	2.926(12)	158(6)
O7_1—H7O_1···O2C_2	0.83(2)	1.97(9)	2.75(2)	157(7)
O8_1—H8O_1···O2_2	0.83(2)	2.03(8)	2.831(12)	160(7)
O8_1—H8O_1···O2C_2	0.83(2)	2.43(8)	3.19(2)	151(6)
O10_1—H10O_1···O1_1	0.83(2)	1.98(3)	2.803(5)	168(7)
O11_1—H11O_1···O24_1	0.83(2)	1.99(4)	2.818(5)	170(8)
O12_1—H12O_1···O2_1	0.83(2)	1.99(7)	2.810(6)	168(7)
O14_1—H14O_1···O26_1	0.829(18)	2.09(5)	2.887(6)	160(6)
O15_1—H15O_1···O4_1	0.862(16)	2.13(8)	2.982(10)	171(6)
O16_1—H16O_1···O27_1	0.881(15)	1.93(3)	2.811(6)	174(8)
O17_1—H17O_1···O5_1	0.992(15)	2.13(2)	2.976(5)	142.1(16)
O18_1—H18O_1···O28_1	0.839(15)	2.00(5)	2.836(6)	177(6)
O19_1—H19O_1···O29_1	0.903(17)	2.28(3)	3.158(6)	165(6)
O20_1—H20O_1···O7_1	0.84(2)	1.96(4)	2.787(5)	169(7)
O21_1—H21O_1···O30_1	0.84(2)	1.99(9)	2.771(7)	159(7)
O22_1—H22O_1···O8_1	0.83(2)	1.98(3)	2.813(5)	177(8)
O23_1—H23O_1···O31_1	0.841(16)	2.01(4)	2.839(6)	167(6)
O24_1—H24O_1···O3_2	0.84(2)	2.01(4)	2.836(8)	167(8)
O24_1—H24O_1···O3C_2	0.84(2)	2.07(5)	2.901(14)	171(7)
O25_1—H25O_1···O3_2	0.83(2)	1.97(7)	2.808(11)	176(5)
O26_1—H26O_1···O1C_2	0.84(2)	2.25(10)	3.05(2)	162(6)
O27_1—H27O_1···O1_2	0.84(2)	2.25(7)	3.034(11)	160(8)
O27_1—H27O_1···O1C_2	0.84(2)	2.29(6)	3.100(17)	172(2)
O28_1—H28O_1···O1_2	0.83(2)	2.24(5)	3.007(8)	153(5)
O30_1—H30O_1···O2_2	0.84(2)	2.02(3)	2.850(9)	173(8)
O30_1—H30O_1···O2C_2	0.84(2)	2.15(3)	2.946(16)	159(7)

Table S42. Hydrogen bonding data for the minor unit (Cu_{9+13+7}, 5%) of **5** (O1_1–O9_1: Cu_9-ring; O10_1–O12_1, O13B_1–O19B_1, O20_1–O23_1: Cu_{13}-ring; O30_1, O31_1, O24B_1–O29B_1: Cu_7-ring; O1B_2–O3B_2: $EtPO_3^{2-}$ anion with occupancy ratio 0.05).

D*—H···*A	***D*—H (Å)**	**H···*A* (Å)**	***D*···*A* (Å)**	***D*—H···*A* (°)**
O1_1—H1O_1···O3B_2	0.83(2)	1.95(5)	2.76(4)	169(7)
O3_1—H3O_1···O1B_2	0.84(2)	1.75(9)	2.58(4)	166(8)
O4_1—H4O_1···O1B_2	0.84(2)	2.04(5)	2.76(4)	142(5)
O6_1—H6O_1···O2B_2	0.83(2)	1.93(5)	2.72(4)	158(8)
O7_1—H7O_1···O2B_2	0.83(2)	2.13(11)	2.92(6)	158(7)
O9_1—H9O_1···O3B_2	0.82(2)	1.96(7)	2.75(5)	160(9)
O10_1—H10O_1···O1_1	0.83(2)	1.98(3)	2.803(5)	168(7)
O11_1—H11O_1···O24_1	0.83(2)	1.99(4)	2.818(5)	170(8)
O12_1—H12O_1···O2_1	0.83(2)	1.99(7)	2.810(6)	168(7)
O13B_1—H13P_1···O3_1	0.84	2.122(5)	2.89(4)	152(2)
O16B_1—H16P_1···O26B_1	0.84	2.00(3)	2.76(5)	149.7(16)
O17B_1—H17P_4···O5_1	0.84	1.801(5)	2.48(3)	137(2)
O18B_1—H18P_1···O28B_1	0.84	1.90(3)	2.67(4)	151(3)
O20_1—H20O_1···O7_1	0.84(2)	1.96(4)	2.787(5)	169(7)
O21_1—H21O_1···O30_1	0.84(2)	1.99(9)	2.771(7)	159(7)
O22_1—H22O_1···O8_1	0.83(2)	1.98(3)	2.813(5)	177(8)
O23_1—H23O_1···O31_1	0.841(16)	2.01(4)	2.839(6)	167(6)
O24B_1—H24P_1···O3B_2	0.84	2.20(4)	2.97(7)	154(4)
O25B_1—H25P_1···O1B_2	0.84	1.85(3)	2.67(5)	162(3)
O26B_1—H26P_1···O1B_2	0.84	1.81(3)	2.65(4)	177.9(17)
O28B_1—H28P_1···O2B_2	0.84	2.21(3)	3.05(4)	177(3)
O29B_1—H29P_1···O2B_2	0.84	2.20(4)	2.99(5)	154(3)
O31_1—H31O_1···O3B_2	0.82(2)	2.15(5)	2.896(4)	148(7)

Table S43. Hydrogen bonding data for **6** (O1_1–O9_1: Cu_9-ring; O10_1–O22_1: Cu_{13}-ring; O23_1–O29_1: Cu_7-ring; O1_2–O3_2 and O1B_2–O3B_2: $^{n}BuPO_3^{2-}$ anion disordered over two positions in an 82/18 ratio).

D*—H···*A	***D*—H (Å)**	**H···*A* (Å)**	***D*···*A* (Å)**	***D*—H···*A* (°)**
O1_1—H1O_1···O3_2	0.84(2)	1.97(3)	2.762(6)	156(7)
O1_1—H1O_1···O3B_2	0.84(2)	2.36(4)	3.156(18)	157(7)
O2_1—H2O_1···O3_2	0.83(2)	1.95(3)	2.762(5)	168(7)
O2_1—H2O_1···O2B_2	0.83(2)	2.47(4)	3.210(18)	149(6)
O3_1—H3O_1···O2B_2	0.84(2)	1.78(3)	2.609(16)	170(7)
O4_1—H4O_1···O2_2	0.85(2)	2.10(2)	2.943(6)	175(7)
O4_1—H4O_1···O2B_2	0.85(2)	2.42(5)	3.167(19)	148(6)
O5_1—H5O_1···O2_2	0.82(2)	2.03(3)	2.799(5)	156(7)
O5_1—H5O_1···O1B_2	0.82(2)	2.31(4)	3.056(18)	151(6)
O6_1—H6O_1···O1B_2	0.82(2)	1.81(3)	2.627(15)	170(7)
O7_1—H7O_1···O1_2	0.82(2)	2.02(2)	2.825(5)	166(6)
O7_1—H7O_1···O1B_2	0.82(2)	2.33(4)	3.068(16)	150(6)
O8_1—H8O_1···O1_2	0.82(2)	2.09(3)	2.887(5)	164(7)
O9_1—H9O_1···O1_2	0.82(2)	2.52(4)	3.262(5)	151(6)
O9_1—H9O_1···O3B_2	0.82(2)	1.93(3)	2.749(16)	176(7)
O10_1—H10O_1···O23_1	0.83(2)	1.95(3)	2.723(5)	155(6)
O11_1—H11O_1···O1_1	0.82(2)	2.18(3)	2.949(5)	157(7)
O12_1—H12O_1···O2_1	0.82(2)	2.21(4)	2.944(5)	150(7)
O13_1—H13O_1···O25_1	0.83(2)	1.89(2)	2.710(5)	172(7)
O14_1—H14O_1···O3_1	0.83(2)	1.98(2)	2.796(5)	168(6)
O15_1—H15O_1···O26_1	0.85(2)	2.02(3)	2.821(5)	157(7)
O16_1—H16O_1···O5_1	0.85(2)	2.00(2)	2.842(5)	168(7)
O17_1—H17O_1···O27_1	0.83(2)	2.10(2)	2.923(5)	170(7)
O18_1—H18O_1···O6_1	0.84(2)	2.00(2)	2.833(5)	172(7)
O19_1—H19O_1···O28_1	0.83(2)	2.12(3)	2.930(4)	166(7)
O20_1—H20O_1···O7_1	0.82(2)	2.06(2)	2.876(5)	170(7)
O21_1—H21O_1···O29_1	0.82(2)	1.97(2)	2.787(4)	172(6)
O22_1—H22O_1···O9_1	0.82(2)	2.03(3)	2.791(5)	154(6)
O23_1—H23O_1···O3_2	0.83(2)	2.44(3)	3.233(5)	160(6)
O23_1—H23O_1···O3B_2	0.83(2)	1.98(3)	2.775(16)	162(6)
O24_1—H24O_1···O3_2	0.83(2)	2.03(2)	2.854(5)	172(7)
O25_1—H25O_1···O3_2	0.84(2)	2.47(3)	3.245(5)	155(6)
O25_1—H25O_1···O2B_2	0.84(2)	1.98(4)	2.753(17)	152(6)
O26_1—H26O_1···O2_2	0.84(2)	2.00(3)	2.771(5)	153(6)
O26_1—H26O_1···O2B_2	0.84(2)	1.96(3)	2.785(16)	166(6)
O27_1—H27O_1···O2_2	0.84(2)	2.18(4)	2.916(5)	146(6)
O27_1—H27O_1···O1B_2	0.84(2)	2.05(3)	2.866(17)	165(6)
O28_1—H28O_1···O1_2	0.84(2)	2.15(3)	2.939(5)	156(6)
O28_1—H28O_1···O1B_2	0.84(2)	2.06(3)	2.862(18)	159(6)
O29_1—H29O_1···O1_2	0.81(2)	1.96(3)	2.743(5)	161(6)
O29_1—H29O_1···O3B_2	0.81(2)	2.16(4)	2.919(17)	156(6)

Table S44. Hydrogen bonding data for UNIT 1 of **7** (O1_1–O8_1: Cu_8-ring; O9_1–O22_1: Cu_{14}-ring; O23_1–O31_1: Cu_9-ring; O1_2–O3_2: $^{n}C_{12}PO_3^{2-}$ anion).

D*—H···*A	***D*—H (Å)**	**H···*A* (Å)**	***D*···*A* (Å)**	***D*—H···*A* (°)**
O1_1—H1O_1···O3_2	0.84(2)	2.51(3)	3.284(6)	155(6)
O2_1—H2O_1···O2_2	0.84(2)	2.08(4)	2.909(6)	168(7)
O3_1—H3O_1···O2_2	0.83(2)	2.10(7)	2.884(8)	159(6)
O5_1—H5O_1···O1_2	0.802(16)	2.47(7)	3.272(9)	167(4)
O6_1—H6O_1···O1_2	0.85(2)	2.21(6)	3.010(7)	158(5)
O8_1—H8O_1···O3_2	0.81(2)	2.05(3)	2.858(5)	176(5)
O9_1—H9O_1···O31_1	0.81(2)	2.01(4)	2.785(5)	159(5)
O10_1—H10O_1···O1_1	0.83(2)	2.03(7)	2.831(7)	170(4)
O11_1—H11O_1···O24_1	0.83(2)	1.97(4)	2.803(5)	176(5)
O12_1—H12O_1···O2_1	0.83(2)	1.99(5)	2.808(5)	172(6)
O13_1—H13O_1···O25_1	0.84(2)	1.96(6)	2.762(6)	160(5)
O14_1—H14O_1···O26_1	0.845(19)	2.51(4)	3.191(6)	138(3)
O15_1—H15O_1···O4_1	0.81(2)	2.14(4)	2.898(4)	155(4)
O16_1—H16O_1···O27_1	0.818(18)	2.25(6)	3.003(7)	153(4)
O17_1—H17O_1···O5_1	0.82(2)	2.00(4)	2.799(5)	165(4)
O18_1—H18O_1···O28_1	0.82(2)	2.24(7)	3.045(7)	167(4)
O19_1—H19O_1···O6_1	0.807(19)	2.21(6)	2.931(5)	148(7)
O21_1—H21O_1···O30_1	0.83(2)	1.97(3)	2.790(5)	169(6)
O22_1—H22O_1···O8_1	0.81(2)	1.98(5)	2.776(5)	161(5)
O23_1—H23O_1···O3_2	0.82(2)	2.61(6)	3.395(8)	164(4)
O24_1—H24O_1···O2_2	0.81(2)	2.53(6)	3.263(8)	151(6)
O25_1—H25O_1···O2_2	0.83(2)	1.96(4)	2.772(5)	167(6)
O26_1—H26O_1···O2_2	0.83(2)	2.32(4)	3.109(5)	160(6)
O27_1—H27O_1···O1_2	0.83(2)	2.36(3)	3.126(6)	152(4)
O28_1—H28O_1···O1_2	0.83(2)	1.99(3)	2.807(6)	166(4)
O29_1—H29O_1···O1_2	0.84(2)	2.54(6)	3.266(7)	146(5)
O30_1—H30O_1···O3_2	0.813(19)	2.22(3)	2.941(5)	147(5)
O31_1—H31O_1···O3_2	0.820(19)	2.01(5)	2.816(8)	168(4)

Table S45. Hydrogen bonding data for UNIT 2 of **7** (O1_3–O8_3: Cu_8-ring; O9_3–O22_3: Cu_{14}-ring; O23_3–O31_3: Cu_9-ring; O1_4–O3_4: $^{n}C_{12}PO_3^{2-}$ anion).

D*—H···*A	***D*—H (Å)**	**H···*A* (Å)**	***D*···*A* (Å)**	***D*—H···*A* (°)**
O1_3—H1O_3···O2_4	0.85(2)	2.47(4)	3.280(7)	160(5)
O2_3—H2O_3···O3_4	0.806(16)	2.23(6)	3.030(7)	170(5)
O3_3—H3O_3···O3_4	0.852(16)	2.29(9)	3.108(9)	167(6)
O5_3—H5O_3···O1_4	0.84(2)	2.08(7)	2.899(7)	169(8)
O6_3—H6O_3···O1_4	0.83(2)	2.15(3)	2.915(6)	154(4)
O7_3—H7O_3···O2_4	0.83(2)	2.60(3)	3.307(6)	145(5)
O8_3—H8O_3···O2_4	0.82(2)	2.04(3)	2.838(5)	162(6)
O9_3—H9O_3···O31_3	0.809(18)	2.09(2)	2.799(5)	147(5)
O10_3—H10O_3···O23_3	0.83(2)	2.35(5)	3.072(5)	146(6)
O11_3—H11O_3···O2_3	0.858(16)	2.11(5)	2.951(5)	164(6)
O12_3—H12O_3···O24_3	0.84(2)	2.15(7)	2.996(7)	172(5)
O13_3—H13O_3···O3_3	0.83(2)	2.02(4)	2.845(6)	176(7)
O14_3—H14O_3···O25_3	0.82(2)	2.17(8)	2.961(7)	163(4)
O15_3—H15O_3···O4_3	0.83(2)	2.10(4)	2.906(5)	162(8)
O17_3—H17O_3···O27_3	0.816(19)	1.97(6)	2.761(6)	166(6)
O18_3—H18O_3···O6_3	0.84(2)	2.03(5)	2.807(5)	156(4)
O19_3—H19O_3···O28_3	0.807(17)	2.08(3)	2.839(5)	156(6)
O20_3—H20O_3···O7_3	0.82(2)	2.04(8)	2.846(7)	164(5)
O21_3—H21O_3···O30_3	0.85(2)	1.97(4)	2.802(5)	164(7)
O22_3—H22O_3···O8_3	0.82(2)	1.97(4)	2.782(6)	168(5)
O24_3—H24O_3···O3_4	0.84(2)	2.03(3)	2.833(6)	160(8)
O25_3—H25O_3···O3_4	0.83(2)	2.24(4)	3.050(5)	166(5)
O26_3—H26O_3···O1_4	0.83(2)	2.35(3)	3.135(5)	157(4)
O27_3—H27O_3···O1_4	0.83(2)	2.06(4)	2.789(5)	148(5)
O28_3—H28O_3···O1_4	0.84(2)	2.44(7)	3.182(8)	149(6)
O30_3—H30O_3···O2_4	0.82(2)	2.04(6)	2.860(7)	169(5)
O31_3—H31O_3···O2_4	0.834(19)	2.09(3)	2.914(5)	171(4)

Table S46. Hydrogen bonding data for **8** (O1_1–O9_1 and O1_1, O2B_1–O7B_1, O8_1, O9_1: Cu_9-ring partially disordered over two positions with 0.56/0.44 occupancy; O10_1–O23_1: Cu_{14}-ring; O24_1–O31_1: Cu_8-ring; O1_2–O3_2: $BnPO_3^{2-}$ anion).

D*—H···*A	***D*—H (Å)**	**H···*A* (Å)**	***D*···*A* (Å)**	***D*—H···*A* (°)**
O1_1—H1O_1···O1_2	0.919(14)	2.626(19)	3.353(6)	136(2)
O2_1—H2O_1···O1_2	0.839(12)	2.098(5)	2.909(10)	163.0(7)
O2B_1—H2P_1···O1_2	0.836(15)	1.917(5)	2.677(12)	150.4(7)
O3_1—H3O_1···O1_2	0.838(11)	2.306(5)	3.063(9)	150.5(6)
O3B_1—H3P_1···O1_2	0.919(13)	2.083(5)	3.003(11)	179.3(7)
O5_1—H5O_1···O2_2	0.840(9)	2.066(4)	2.900(10)	172.2(7)
O5B_1—H5P_1···O2_2	0.845(11)	2.150(4)	2.969(11)	163.0(10)
O6_1—H6O_1···O2_2	0.850(9)	1.914(4)	2.693(11)	151.7(10)
O6B_1—H6P_1···O2_2	0.870(12)	2.034(4)	2.898(12)	172.7(11)
O7_1—H7O_1···O2_2	0.830(16)	2.597(5)	3.377(10)	157.3(7)
O7B_1—H7P_1···O2_2	0.886(12)	2.601(5)	3.340(11)	141.5(15)
O8_1—H8O_1···O3_2	0.83(2)	2.12(4)	2.913(5)	159(8)
O9_1—H9O_1···O3_2	0.83(2)	2.11(4)	2.881(5)	156(8)
O10_1—H10O_1···O24_1	0.84(2)	2.08(3)	2.901(6)	166(9)
O12_1—H12O_1···O2_1	0.83(2)	1.85(4)	2.659(13)	164(9)
O12_1—H12O_1···O2B_1	0.83(2)	2.13(3)	2.950(16)	167(9)
O13_1—H13O_1···O26_1	0.84(2)	1.99(3)	2.816(5)	170(9)
O14_1—H14O_1···O3_1	0.83(2)	1.90(2)	2.735(12)	176(9)
O14_1—H14O_1···O3B_1	0.83(2)	2.06(3)	2.879(14)	168(9)
O15_1—H15O_1···O27_1	0.83(2)	2.02(4)	2.816(6)	160(9)
O16_1—H16O_1···O5_1	0.84(2)	1.91(3)	2.740(13)	169(9)
O16_1—H16O_1···N9_1	0.84(2)	2.69(8)	3.192(14)	120(7)
O16_1—H16O_1···O5B_1	0.84(2)	2.10(3)	2.939(16)	176(9)
O17_1—H17O_1···O28_1	0.865(18)	1.934(18)	2.768(6)	162(6)
O18_1—H18O_1···O6_1	0.84(2)	2.08(4)	2.870(16)	156(8)
O18_1—H18O_1···O6B_1	0.84(2)	1.93(4)	2.724(19)	157(9)
O19_1—H19O_1···O29_1	0.83(2)	2.63(8)	3.186(6)	125(8)
O19_1—H19O_1···O6B_1	0.83(2)	2.56(6)	3.294(16)	148(9)
O20_1—H20O_1···O30_1	0.85(2)	2.05(4)	2.870(6)	161(9)
O21_1—H21O_1···O8_1	0.84(2)	2.09(3)	2.902(7)	162(9)
O22_1—H22O_1···O31_1	0.83(2)	2.09(8)	2.820(6)	146(6)
O23_1—H23O_1···O9_1	0.851(17)	2.121(18)	2.947(6)	164(6)
O23_1—H23O_1···N18_1	0.851(17)	2.69(6)	3.290(7)	129(6)
O24_1—H24O_1···O3_2	0.83(2)	2.54(4)	3.285(6)	151(7)
O25_1—H25O_1···O1_2	0.84(2)	2.18(2)	3.014(6)	176(9)
O26_1—H26O_1···O1_2	0.84(2)	1.99(2)	2.830(6)	179(8)
O27_1—H27O_1···O1_2	0.83(2)	2.66(3)	3.449(6)	160(7)
O28_1—H28O_1···O2_2	0.82(2)	2.18(5)	2.900(6)	147(7)
O29_1—H29O_1···O2_2	0.84(2)	2.33(4)	3.125(6)	158(7)
O30_1—H30O_1···O3_2	0.84(2)	2.38(4)	3.170(6)	157(7)
O31_1—H31O_1···O3_2	0.83(2)	2.26(2)	3.089(6)	175(8)

Table S47. Hydrogen bonding data for the major component ($Cu_{6+12+10}$, 82%) in UNIT 1 of **9** (O1_3–O10_3: Cu_{10}-ring; O11_3–O22_3: Cu_{12}-ring; O23_3–O28_3: Cu_6-ring; O1_1–O3_1, O1B_1–O3B_1: $PhPO_3^{2-}$ anion with its PO_3 unit disordered over two positions with 0.86/0.14 occupancy).

D*—H···*A	***D*—H (Å)**	**H···*A* (Å)**	***D*···*A* (Å)**	***D*—H···*A* (°)**
O1_3—H1O_3···O1_1	0.84(2)	2.17(6)	2.935(6)	153(7)
O3_3—H3O_3···O2_1	0.84(2)	2.15(7)	2.971(7)	173(10)
O3_3—H3O_3···O2B_1	0.84(2)	2.01(8)	2.75(2)	149(7)
O4_3—H4O_3···O2_1	0.85(2)	2.15(8)	2.957(8)	161(10)
O5_3—H5O_3···O2_1	0.83(2)	2.31(8)	3.035(7)	146(7)
O5_3—H5O_3···O3B_1	0.83(2)	2.32(7)	3.13(3)	166(8)
O6_3—H6O_3···O3B_1	0.84(2)	2.33(8)	3.04(2)	142(9)
O7_3—H7O_3···O3_1	0.853(18)	2.11(3)	2.945(6)	165(7)
O7_3—H7O_3···O3B_1	0.853(18)	2.19(5)	2.99(2)	155(7)
O8_3—H8O_3···O3_1	0.84(2)	2.05(6)	2.885(7)	172(10)
O9_3—H9O_3···O1B_1	0.83(2)	2.09(6)	2.91(2)	164(7)
O10_3—H10O_3···O1_1	0.83(2)	2.20(5)	2.931(7)	148(7)
O10_3—H10O_3···O1B_1	0.83(2)	2.10(6)	2.79(2)	140(7)
O11_3—H11O_3···O23_3	0.83(2)	1.96(5)	2.744(4)	156(6)
O12_3—H12O_3···O2_3	0.84(2)	2.07(5)	2.866(11)	157(7)
O13_3—H13O_3···O24_3	0.83(2)	1.95(6)	2.742(5)	160(7)
O14_3—H14O_3···O3_3	0.83(2)	1.90(5)	2.727(9)	172(8)
O15_3—H15O_3···O25_3	0.83(2)	1.95(6)	2.738(5)	157(7)
O16_3—H16O_3···O5_3	0.82(2)	1.87(4)	2.674(7)	165(7)
O17_3—H17O_3···O26_3	0.83(2)	1.97(5)	2.756(5)	161(6)
O18_3—H18O_3···O7_3	0.85(2)	1.94(4)	2.756(7)	161(7)
O19_3—H19O_3···O27_3	0.83(2)	1.91(6)	2.712(4)	163(7)
O20_3—H20O_3···O8_3	0.84(2)	1.95(5)	2.763(10)	164(6)
O21_3—H21O_3···O28_3	0.83(2)	1.98(4)	2.762(4)	156(7)
O22_3—H22O_3···O10_3	0.83(2)	1.88(5)	2.685(5)	163(6)
O23_3—H23O_3···O1_1	0.83(2)	2.01(4)	2.797(6)	172(5)
O23_3—H23O_3···O1_1	0.83(2)	2.46(6)	3.20(2)	150(5)
O24_3—H24O_3···O2B_2	0.83(2)	2.01(4)	2.85(2)	167(5)
O25_3—H25O_3···O2_1	0.82(2)	1.97(7)	2.782(6)	175(6)
O26_3—H26O_3···O3B_1	0.83(2)	2.03(5)	2.79(3)	153(5)
O27_3—H27O_3···O3_1	0.84(2)	1.99(4)	2.810(5)	166(6)
O28_3—H28O_3···O1B_1	0.85(2)	1.95(5)	2.77(2)	163(6)

Table S48. Hydrogen bonding data for the minor component (Cu_{6+12+9}, 18%) in UNIT 1 of **9** (O1_3, O2B_3–O7B_3, O9_3, O10_3: Cu_9-ring; O11_3–O22_3: Cu_{12}-ring; O23_3–O28_3: Cu_6-ring; O1_1–O3_1, O1B_1–O3B_1: $PhPO_3^{2-}$ anion with its PO_3 unit disordered over two positions with 0.86/0.14 occupancy).

D*—H···*A	***D*—H (Å)**	**H···*A* (Å)**	***D*···*A* (Å)**	***D*—H···*A* (°)**
O1_3—H1O_3···O1_1	0.84(2)	2.17(6)	2.935(6)	153(7)
O2B_3—H2P_3···O1_1	0.85(3)	2.388(5)	3.19(3)	158(2)
O2B_3—H2P_3···O2B_1	0.85(3)	2.107(17)	2.90(4)	156(2)
O3B_3—H3P_3···O2_1	0.85(2)	1.910(5)	2.76(2)	177(2)
O3B_3—H3PO_3···O2B_1	0.85(2)	1.89(2)	2.62(3)	142.2(13)
O4B_3—H4P_3···O2_1	0.84(2)	1.800(5)	2.53(2)	144.3(11)
O5B_3—H5P_3···O2_1	0.845(16)	2.003(2)	2.787(15)	154.1(15)
O6B_3—H6P_3···O3_1	0.841(18)	1.900(5)	2.553(17)	133.5(16)
O7B_3—H7P_3···O1B_1	0.84(3)	2.40(2)	3.17(3)	154(4)
O9_3—H9O_3···O1B_1	0.83(2)	2.09(6)	2.91(2)	164(7)
O10_3—H10O_3···O1_1	0.83(2)	2.20(5)	2.931(7)	148(7)
O10_3—H10O_3···O1B_1	0.83(2)	2.10(6)	2.79(2)	140(7)
O11_3—H11O_3···O23_3	0.83(2)	1.96(5)	2.744(4)	156(6)
O12_3—H12O_3···O2B_3	0.84(2)	2.28(7)	3.06(5)	154(6)
O13_3—H13O_3···O24_3	0.83(2)	1.95(6)	2.742(5)	160(7)
O14_3—H14O_3···O3B_3	0.83(2)	1.95(7)	2.78(4)	179(6)
O15_3—H15O_3···O25_3	0.83(2)	1.95(6)	2.738(5)	157(7)
O17_3—H17O_3···O26_3	0.83(2)	1.97(5)	2.756(5)	161(6)
O18_3—H18O_3···O6B_3	0.85(2)	2.08(5)	2.90(3)	163(7)
O19_3—H19O_3···O27_3	0.83(2)	1.91(6)	2.712(4)	163(7)
O20_3—H20O_3···O7B_3	0.84(2)	2.19(7)	3.01(5)	171(6)
O21_3—H21O_3···O28_3	0.83(2)	1.98(4)	2.762(4)	156(7)
O22_3—H22O_3···O10_3	0.83(2)	1.88(5)	2.685(5)	163(6)
O23_3—H23O_3···O1_1	0.83(2)	2.01(4)	2.797(6)	172(5)
O23_3—H23O_3···O1_1	0.83(2)	2.46(6)	3.20(2)	150(5)
O24_3—H24O_3···O2B_2	0.83(2)	2.01(4)	2.85(2)	167(5)
O25_3—H25O_3···O2_1	0.82(2)	1.97(7)	2.782(6)	175(6)
O26_3—H26O_3···O3B_1	0.83(2)	2.03(5)	2.79(3)	153(5)
O27_3—H27O_3···O3_1	0.84(2)	1.99(4)	2.810(5)	166(6)
O28_3—H28O_3···O1B_1	0.85(2)	1.95(5)	2.77(2)	163(6)

Table S49. Hydrogen bonding data for the major component ($Cu_{6+12+10}$, 91%) in UNIT 2 of **9** (O1_4–O10_4: Cu_{10}-ring; O11_4–O22_4: Cu_{12}-ring; O23_4–O28_4: Cu_6-ring; O1_2–O3_2, O1B_2–O3B_2: $PhPO_3^{2-}$ anion with its PO_3 unit disordered over two positions with 0.89/0.11 occupancy).

D*—H···*A	***D*—H (Å)**	**H···*A* (Å)**	***D*···*A* (Å)**	***D*—H···*A* (°)**
O1_4—H1O_4···O1_2	0.84(2)	2.07(7)	2.87(3)	160(6)
O2_4—H2O_4···O1_2	0.84(2)	2.19(5)	2.900(5)	142(7)
O3_4—H3O_4···O1_2	0.84(2)	2.10(5)	2.918(6)	164(7)
O3_4—H3O_4···O1B_2	0.84(2)	2.21(5)	3.01(3)	160(7)
O4_4—H4O_4···O1B_2	0.84(2)	2.12(7)	2.95(3)	167(6)
O5_4—H5O_4···O1B_2	0.84(2)	2.29(5)	3.10(2)	164(6)
O5_4—H5O_4···O2_2	0.84(2)	2.22(4)	2.974(5)	151(6)
O6_4—H6O_4···O2_2	0.84(2)	2.23(7)	2.989(6)	152(7)
O7_4—H7O_4···O2_2	0.84(2)	2.11(3)	3.044(6)	155(6)
O7_4—H7O_4···O2B_2	0.84(2)	1.98(5)	2.806(17)	167(7)
O8_4—H8O_4···O2B_2	0.881(15)	2.21(6)	2.91(2)	136(6)
O8_4—H8O_4···O3_2	0.881(15)	2.16(3)	3.038(6)	176(7)
O9_4—H9O_4···O3_2	0.83(2)	2.13(6)	2.946(6)	170(5)
O10_4—H10O_4···O3_2	0.82(2)	2.13(4)	2.930(5)	164(6)
O10_4—H10O_4···O3B_2	0.82(2)	2.16(4)	2.90(3)	151(6)
O11_4—H11O_4···O23_4	0.84(2)	2.01(6)	2.754(4)	147(6)
O12_4—H12O_4···O2_4	0.84(2)	2.11(5)	2.773(7)	136(7)
O13_4—H13O_4···O24_4	0.83(2)	1.98(4)	2.728(5)	150(7)
O14_4—H14O_4···O3_4	0.85(2)	1.94(5)	2.791(6)	176(7)
O15_4—H15O_4···O25_4	0.83(2)	1.95(5)	2.763(4)	168(6)
O16_4—H16O_4···O5_4	0.84(2)	1.86(5)	2.674(5)	164(6)
O17_4—H17O_4···O26_4	0.84(2)	1.94(5)	2.741(4)	160(6)
O18_4—H18O_4···O7_4	0.86(2)	1.93(5)	2.776(5)	172(6)
O19_4—H19O_4···O27_4	0.85(2)	1.95(4)	2.744(4)	156(5)
O20_4—H20O_4···O8_4	0.82(2)	2.18(6)	2.852(6)	139(5)
O21_4—H21O_4···O28_4	0.83(2)	1.93(4)	2.738(4)	166(6)
O22_4—H22O_4···O10_4	0.83(2)	1.89(4)	2.698(4)	164(6)
O23_4—H23O_4···O3B_2	0.84(2)	1.93(5)	2.72(3)	156(5)
O24_4—H24O_4···O1_2	0.84(2)	2.03(5)	2.853(5)	168(5)
O25_4—H25O_4···O1B_2	0.83(2)	2.05(5)	2.81(3)	152(5)
O26_4—H26O_4···O2_2	0.83(2)	2.04(6)	2.784(5)	150(5)
O27_4—H27O_4···O2B_2	0.85(2)	1.92(5)	2.76(2)	167(5)
O28_4—H28O_4···O3_2	0.829(17)	2.00(3)	2.786(5)	157(4)

Table S50. Hydrogen bonding data for the minor component (Cu_{6+12+9}, 9%) in UNIT 2 of **9** (O1_4, O2B_4–O7B_4, O9_4, O10_4: Cu_9-ring; O11_4–O22_4: Cu_{12}-ring; O23_4–O28_4: Cu_6-ring; O1_2–O3_2, O1B_2–O3B_2: $PhPO_3^{2-}$ anion with its PO_3 unit disordered over two positions with 0.89/0.11 occupancy).

D*—H···*A	***D*—H (Å)**	**H···*A* (Å)**	***D*···*A* (Å)**	***D*—H···*A* (°)**
O1_4—H1O_4···O1_2	0.84(2)	2.07(7)	2.87(3)	160(6)
O2B_4—H2P_4···O1_2	0.90(3)	1.964(4)	2.59(3)	125(3)
O2B_4—H2P_4···O3B_2	0.90(3)	2.21(2)	3.03(3)	150(4)
O3B_4—H3P_4···O1_2	0.839(17)	1.899(5)	2.537(17)	131.8(17)
O4B_4—H4P_4···O2_2	0.85(2)	2.008(4)	2.86(2)	177.4(16)
O6B_4—H6P_4···O2_2	0.84(2)	1.752(4)	2.579(2)	163.9(12)
O6B_4—H6P_4···O2B_2	0.84(2)	1.73(2)	2.51(3)	152.4(16)
O7B_4—H7P_4···O3_2	0.84(2)	1.965(4)	2.80(3)	155.4(19)
O9_4—H9O_4···O3_2	0.83(2)	2.13(6)	2.946(6)	170(5)
O10_4—H10O_4···O3_2	0.82(2)	2.13(4)	2.930(5)	164(6)
O10_4—H10O_4···O3B_2	0.82(2)	2.16(4)	2.90(3)	151(6)
O11_4—H11O_4···O23_4	0.84(2)	2.01(6)	2.754(4)	147(6)
O12_4—H12O_4···O2B_4	0.84(2)	2.49(7)	3.12(5)	133(7)
O13_4—H13O_4···O24_4	0.83(2)	1.98(4)	2.728(5)	150(7)
O14_4—H14O_4···O3B_4	0.85(2)	2.01(7)	2.81(4)	156(7)
O15_4—H15O_4···O25_4	0.83(2)	1.95(5)	2.763(4)	168(6)
O17_4—H17O_4···O26_4	0.84(2)	1.94(5)	2.741(4)	160(6)
O18_4—H18O_4···O6B_4	0.86(2)	1.91(7)	2.75(4)	166(6)
O19_4—H19O_4···O27_4	0.85(2)	1.95(4)	2.744(4)	156(5)
O20_4—H20O_4···O7B_4	0.82(2)	2.29(8)	3.01(5)	147(5)
O21_4—H21O_4···O28_4	0.83(2)	1.93(4)	2.738(4)	166(6)
O22_4—H22O_4···O10_4	0.83(2)	1.89(4)	2.698(4)	164(6)
O23_4—H23O_4···O3B_2	0.84(2)	1.93(5)	2.72(3)	156(5)
O24_4—H24O_4···O1_2	0.84(2)	2.03(5)	2.853(5)	168(5)
O25_4—H25O_4···O1B_2	0.83(2)	2.05(5)	2.81(3)	152(5)
O26_4—H26O_4···O2_2	0.83(2)	2.04(6)	2.784(5)	150(5)
O27_4—H27O_4···O2B_2	0.85(2)	1.92(5)	2.76(2)	167(5)
O28_4—H28O_4···O3_2	0.829(17)	2.00(3)	2.786(5)	157(4)

Table S51. Hydrogen bonding data for **10** (O1_1–O8_1: Cu_8-ring; O9_1–O22_1: Cu_{14}-ring; O23_1–O31_1: Cu_9-ring; O1_2–O3_2: $PhPO_3^{2-}$ anion).

D*—H···*A	***D*—H (Å)**	**H···*A* (Å)**	***D*···*A* (Å)**	***D*—H···*A* (°)**
O1_1—H1O_1···O1_2	0.822(19)	2.02(2)	2.824(3)	166(4)
O3_1—H3O_1···O2_2	0.824(19)	2.13(2)	2.919(3)	160(4)
O4_1—H4O_1···O2_2	0.814(19)	2.36(3)	3.114(3)	154(4)
O5_1—H5O_1···O3_2	0.824(19)	2.34(2)	3.143(3)	166(4)
O6_1—H6O_1···O3_2	0.824(19)	2.34(2)	3.159(3)	177(4)
O7_1—H7O_1···O3_2	0.832(19)	2.48(2)	3.268(3)	158(4)
O8_1—H8O_1···O1_2	0.825(19)	2.27(2)	3.057(3)	159(4)
O9_1—H9O_1···O1_1	0.826(19)	1.96(2)	2.778(3)	172(5)
O10_1—H10O_1···O24_1	0.827(19)	1.98(2)	2.790(3)	168(5)
O11_1—H11O_1···O2_1	0.829(19)	2.00(2)	2.820(3)	173(4)
O12_1—H12O_1···O26_1	0.812(19)	2.00(2)	2.815(3)	179(5)
O13_1—H13O_1···O3_1	0.832(19)	1.94(2)	2.774(3)	176(5)
O14_1—H14O_1···O27_1	0.835(19)	1.94(2)	2.771(3)	179(5)
O16_1—H16O_1···O5_1	0.825(19)	2.08(2)	2.905(3)	177(5)
O17_1—H17O_1···O29_1	0.823(19)	2.13(2)	2.952(3)	176(4)
O18_1—H18O_1···O6_1	0.809(19)	2.00(2)	2.784(3)	163(5)
O19_1—H19O_1···O30_1	0.818(19)	2.22(2)	3.038(3)	173(5)
O20_1—H20O_1···O7_1	0.835(19)	2.07(2)	2.895(3)	168(5)
O21_1—H21O_1···O31_1	0.825(19)	2.51(4)	3.130(3)	132(4)
O22_1—H22O_1···O23_1	0.831(19)	1.92(2)	2.753(3)	179(6)
O23_1—H23O_1···O1_2	0.807(19)	1.99(2)	2.791(3)	173(4)
O24_1—H24O_1···O1_2	0.817(19)	2.21(2)	2.995(3)	162(4)
O26_1—H26O_1···O2_2	0.820(19)	2.15(2)	2.943(3)	163(4)
O27_1—H27O_1···O2_2	0.802(19)	2.01(2)	2.805(3)	172(4)
O29_1—H29O_1···O3_2	0.806(19)	2.09(2)	2.880(3)	166(4)
O30_1—H30O_1···O3_2	0.827(19)	2.06(2)	2.858(3)	163(4)
O31_1—H31O_1···O1_2	0.826(19)	2.56(3)	3.331(3)	157(4)

Table S52. Hydrogen bonding data for **11** (O1_3–O6_3: Cu_6-ring; O7_3–O18_3: Cu_{12}-ring; O19_3–O27_3: Cu_9-ring; O1_1–O3_1: $EtPO_3^{2-}$ anion).

D*—H···*A	***D*—H (Å)**	**H···*A* (Å)**	***D*···*A* (Å)**	***D*—H···*A* (°)**
O1_3—H1O_3···O3_1	0.832(19)	1.83(3)	2.662(2)	173(3)
O2_3—H2O_3···O3_1	0.787(19)	2.513(19)	3.252(2)	157(3)
O3_3—H3O_3···O1_1	0.811(18)	1.95(2)	2.7487(18)	166(4)
O4_3—H4O_3···O1_1	0.796(18)	2.50(3)	3.203(3)	148(4)
O5_3—H5O_3···O2_1	0.815(18)	2.04(3)	2.840(3)	168(4)
O6_3—H6O_3···O3_1	0.825(19)	2.44(5)	3.194(3)	152(4)
O7_3—H7O_3···O1_3	0.813(19)	1.93(4)	2.734(3)	170(3)
O8_3—H8O_3···O20_3	0.819(19)	2.14(3)	2.948(2)	166(3)
O9_3—H9O_3···O2_3	0.824(18)	2.00(5)	2.823(4)	170(3)
O10_3—H10O_3···O21_3	0.812(18)	1.96(2)	2.7612(19)	172(4)
O11_3—H11O_3···O3_3	0.801(18)	1.96(4)	2.752(3)	173(3)
O12_3—H12O_3···O23_3	0.802(19)	2.08(2)	2.8666(18)	166(4)
O13_3—H13O_3···O4_3	0.801(18)	1.96(3)	2.755(3)	171(4)
O14_3—H14O_3···O24_3	0.815(18)	1.94(3)	2.751(3)	176(4)
O15_3—H15O_3···O5_3	0.816(18)	1.99(3)	2.792(3)	166(4)
O16_3—H16O_3···O26_3	0.826(18)	1.96(4)	2.784(3)	178(3)
O17_3—H17O_3···O6_3	0.840(18)	1.93(3)	2.768(2)	175(4)
O18_3—H18O_3···O27_3	0.846(19)	2.08(3)	2.917(2)	170(3)
O19_3—H19O_3···O3_1	0.813(18)	2.08(3)	2.850(3)	159(4)
O20_3—H20O_3···O3_1	0.797(18)	2.23(5)	3.013(4)	166(3)
O21_3—H21O_3···O1_1	0.818(18)	2.08(4)	2.873(3)	165(3)
O22_3—H22O_3···O1_1	0.814(18)	2.05(4)	2.837(3)	165(3)
O23_3—H23O_3···O1_1	0.803(18)	2.07(3)	2.866(3)	169(4)
O24_3—H24O_3···O2_1	0.803(18)	2.06(3)	2.846(3)	167(5)
O25_3—H25O_3···O2_1	0.821(18)	2.07(3)	2.875(3)	166(4)
O26_3—H26O_3···O2_1	0.825(18)	1.98(3)	2.783(3)	164(4)
O27_3—H27O_3···O3_1	0.832(18)	2.29(3)	3.104(2)	167(4)

Table S53. Hydrogen bonding data for **12** (O1_1–O9_1 and O1_2–O9_2: Cu_9-rings; O10_1–O21_1 and O10_2–O21_2: Cu_{12}-rings; O22_1–O27_1 and O22_2–O27_2: Cu_6-rings; O1_5–O3_5 and O1_6–O3_6: ${}^{n}PrPO_3^{2-}$ incarcerated anions).

D*—H···*A	***D*—H (Å)**	**H···*A* (Å)**	***D*···*A* (Å)**	***D*—H···*A* (°)**
O1_1—H1O_1···O3_5	0.832(19)	2.09(3)	2.911(3)	170(4)
O2_1—H2O_1···O2_5	0.831(19)	2.03(3)	2.844(3)	166(4)
O3_1—H3O_1···O2_5	0.826(19)	1.99(4)	2.793(3)	164(4)
O4_1—H4O_1···O2_5	0.835(19)	2.02(3)	2.808(3)	157(4)
O5_1—H5O_1···O1_5	0.834(19)	2.15(3)	2.944(3)	159(4)
O6_1—H6O_1···O1_5	0.826(19)	1.99(2)	2.800(3)	167(4)
O7_1—H7O_1···O1_5	0.827(19)	2.01(4)	2.816(3)	164(4)
O8_1—H8O_1···O3_5	0.813(19)	2.29(4)	3.065(3)	160(4)
O9_1—H9O_1···O3_5	0.834(19)	1.98(4)	2.792(3)	166(4)
O10_1—H10O_1···O22_1	0.825(19)	1.94(4)	2.739(3)	165(4)
O11_1—H11O_1···O2_1	0.835(19)	1.93(3)	2.761(3)	173(4)
O12_1—H12O_1···O23_1	0.823(19)	1.94(4)	2.753(3)	168(4)
O13_1—H13O_1···O4_1	0.831(19)	1.96(4)	2.785(3)	169(4)
O14_1—H14O_1···O24_1	0.828(19)	1.92(3)	2.735(3)	170(4)
O15_1—H15O_1···O5_1	0.824(19)	1.99(2)	2.800(3)	169(4)
O16_1—H16O_1···O25_1	0.830(19)	1.93(2)	2.756(3)	173(4)
O17_1—H17O_1···O7_1	0.841(19)	1.92(2)	2.753(3)	171(4)
O18_1—H18O_1···O26_1	0.837(19)	1.94(4)	2.756(3)	165(4)
O19_1—H19O_1···O8_1	0.833(19)	2.01(4)	2.844(3)	174(4)
O20_1—H20O_1···O27_1	0.813(19)	1.96(4)	2.754(3)	166(4)
O21_1—H21O_1···O1_1	0.832(19)	2.12(4)	2.945(3)	172(4)
O22_1—H22O_1···O3_5	0.821(19)	2.37(3)	3.146(3)	157(4)
O23_1—H23O_1···O2_5	0.833(19)	1.97(4)	2.776(3)	164(4)
O25_1—H25O_1···O1_5	0.826(19)	1.93(2)	2.734(3)	165(4)
O27_1—H27O_1···O3_5	0.831(19)	1.91(4)	2.691(3)	156(4)
O1_2—H1O_2···O3_6	0.811(19)	2.01(3)	2.815(3)	170(4)
O2_2—H2O_2···O2_6	0.824(19)	2.03(3)	2.997(4)	151(4)
O3_2—H3O_2···O2_6	0.828(19)	1.98(4)	2.801(3)	171(4)
O4_2—H4O_2···O2_6	0.815(19)	1.96(3)	2.744(3)	162(4)
O5_2—H5O_2···O1_6	0.816(19)	2.54(4)	3.267(4)	149(3)
O6_2—H6O_2···O1_6	0.827(19)	2.03(2)	2.839(3)	164(4)
O7_2—H7O_2···O1_6	0.819(19)	1.92(4)	2.729(4)	171(4)
O8_2—H8O_2···O3_6	0.824(19)	2.50(4)	3.251(4)	153(4)
O9_2—H9O_2···O3_6	0.827(19)	2.00(4)	2.811(3)	168(4)
O10_2—H10O_2···O22_2	0.818(19)	1.91(3)	2.720(3)	171(4)
O11_2—H11O_2···O2_2	0.832(19)	1.98(3)	2.802(3)	172(4)
O12_2—H12O_2···O23_2	0.820(19)	1.96(4)	2.747(3)	161(4)
O13_2—H13O_2···O4_2	0.822(19)	1.91(4)	2.734(3)	176(4)
O14_2—H14O_2···O24_2	0.819(19)	1.96(3)	2.758(3)	166(4)
O15_2—H15O_2···O5_2	0.806(19)	2.00(2)	2.793(3)	169(4)
O16_2—H16O_2···O25_2	0.835(19)	1.90(2)	2.730(3)	172(4)
O17_2—H17O_2···O7_2	0.826(19)	1.93(2)	2.746(3)	169(4)
O18_2—H18O_2···O26_2	0.830(19)	1.91(4)	2.736(3)	171(4)
O19_2—H19O_2···O8_2	0.828(19)	2.05(4)	2.874(3)	170(4)
O20_2—H20O_2···O27_2	0.832(19)	1.93(4)	2.760(3)	172(4)
O21_2—H21O_2···O1_2	0.832(19)	2.08(4)	2.905(3)	169(4)
O22_2—H22O_2···O3_6	0.815(19)	2.22(3)	2.993(3)	157(4)
O23_2—H23O_2···O2_6	0.818(19)	2.05(4)	2.843(4)	164(4)
O25_2—H25O_2···O1_6	0.819(19)	1.93(2)	2.717(3)	160(4)
O26_2—H26O_2···O1_6	0.806(19)	2.34(3)	3.075(4)	153(4)
O27_2—H27O_2···O3_6	0.837(19)	1.96(4)	2.738(3)	155(4)

3. NMR SPECTROSCOPIC DATA

Table S54. Variable-temperature ^{1}H NMR chemical shifts (ppm) in DMSO-d_6 of nanojars **Cu_nCO_3** (in green), **Cu_nSO_4** (in light blue), **Cu_nMePO_3** (in orange), **Cu_nEtPO_3** (in violet), **$Cu_n{}^nPrPO_3$** (in magenta), **$Cu_n{}^nBuPO_3$** (in red), **Cu_nPhPO_3** (in olive) and **Cu_nBnPO_3** (in blue). Missing values are due to lack of significant amounts of a particular species in the mixture and/or to inability of unambiguous assignment due to overlap or excessive broadening. Ambient temperatures varied between 20–23 °C (see individual VT-NMR plots for actual temperatures).

NANOJAR	20–23 °C	30 °C	40 °C	50 °C	60 °C	70 °C	80 °C	90 °C	100 °C	110 °C	120 °C	130 °C	140 °C	150 °C
**Cu_{27} (6+12+9)**														
Cu_9 ring, pz-4-*H*	37.63<br>34.83<br>32.75<br>32.87<br>32.16<br>31.95<br>30.07<br>28.83	37.23<br>34.66<br>32.64<br>32.64<br>31.98<br>31.78<br>30.00<br>28.72	36.75<br>34.31<br>32.47<br>32.38<br>31.78<br>31.56<br>29.89<br>28.59	36.29<br>33.93<br>32.28<br>32.11<br>31.56<br>31.33<br>29.78<br>28.47	35.84<br>33.55<br>32.06<br>31.84<br>31.34<br>31.09<br>29.66<br>28.34	35.41<br>33.17<br>31.83<br>31.56<br>31.10<br>30.85<br>29.54<br>28.20	35.00<br>32.77<br>31.57<br>31.27<br>30.86<br>30.60<br>29.41<br>28.05	34.59<br>32.39<br>31.31<br>30.97<br>30.60<br>30.34<br>29.27<br>27.91	34.20<br>32.01<br>31.03<br>30.67<br>30.34<br>30.07<br>29.13<br>27.74	33.82<br>31.64<br>30.72<br>30.36<br>30.07<br>29.80<br>28.98<br>27.58	33.44<br>31.29<br>30.43<br>30.04<br>29.80<br>29.51<br>28.83<br>27.41	33.09<br>30.94<br>30.11<br>29.74<br>29.52<br>29.22<br>28.66<br>27.24	32.72<br>30.63<br>29.83<br>29.45<br>29.24<br>28.93<br>28.50<br>27.06	32.39<br>30.28<br>29.53<br>29.16<br>28.97<br>28.64<br>28.32<br>26.87
Cu_9 ring, pz-3,5-H_2	33.24<br>31.03<br>28.51<br>28.48<br>27.92<br>27.74<br>26.39<br>~25.3	32.88<br>30.89<br>28.43<br>28.30<br>27.78<br>27.62<br>26.33<br>25.25	32.45<br>30.62<br>28.30<br>28.12<br>27.62<br>27.45<br>26.24<br>25.16	32.04<br>30.31<br>28.16<br>27.92<br>27.45<br>27.28<br>26.14<br>25.07	31.64<br>30.01<br>28.00<br>27.72<br>27.28<br>27.11<br>26.04<br>24.97	31.26<br>29.69<br>27.83<br>27.52<br>27.10<br>26.93<br>25.94<br>24.88	30.89<br>29.37<br>27.65<br>27.31<br>26.91<br>26.74<br>25.84<br>24.78	30.53<br>29.05<br>27.45<br>27.09<br>26.72<br>26.55<br>25.72<br>24.68	30.17<br>28.73<br>27.24<br>26.86<br>26.53<br>26.35<br>25.60<br>24.57	29.83<br>28.42<br>27.02<br>26.64<br>26.32<br>26.15<br>25.48<br>24.45	29.50<br>28.11<br>26.80<br>26.41<br>26.12<br>25.94<br>25.35<br>24.34	29.19<br>27.82<br>26.57<br>26.18<br>25.91<br>25.73<br>25.23<br>24.21	28.85<br>27.52<br>26.35<br>25.96<br>25.70<br>25.51<br>25.10<br>24.09	28.56<br>27.24<br>26.11<br>25.73<br>25.49<br>25.29<br>24.95<br>23.96
Cu_6 ring, pz-4-*H*	31.26<br>~33.70<br>31.18<br>32.43<br>31.29<br>31.17<br>31.35<br>30.92	31.34<br>~33.65<br>31.22<br>31.49<br>31.36<br>31.23<br>31.42<br>31.01	31.41<br>33.58<br>31.25<br>31.52<br>31.39<br>31.27<br>31.47<br>31.08	31.44<br>33.50<br>31.27<br>31.52<br>31.40<br>31.29<br>31.51<br>31.13	31.45<br>33.40<br>31.25<br>31.50<br>31.38<br>31.28<br>31.51<br>31.15	31.43<br>33.27<br>31.22<br>31.47<br>31.35<br>31.26<br>31.48<br>31.16	31.39<br>33.15<br>31.17<br>31.41<br>31.30<br>31.22<br>31.43<br>31.14	31.34<br>33.00<br>31.10<br>31.35<br>31.24<br>31.16<br>31.37<br>31.11	31.27<br>32.85<br>31.03<br>31.27<br>31.16<br>31.09<br>31.30<br>31.06	31.18<br>32.69<br>30.95<br>31.19<br>31.07<br>31.01<br>31.21<br>31.00	31.09<br>32.53<br>30.86<br>31.10<br>30.98<br>30.90<br>31.12<br>30.94	31.00<br>32.36<br>30.76<br>31.00<br>30.88<br>30.78<br>31.03<br>30.86	30.88<br>32.18<br>30.66<br>30.89<br>30.76<br>30.66<br>30.92<br>30.76	30.76<br>31.98<br>30.55<br>30.78<br>30.65<br>30.53<br>30.80<br>30.66
Cu_6 ring, pz-3,5-H_2	28.24<br>30.18<br>27.88<br>28.10<br>~27.97<br>27.87<br>28.05<br>27.49	28.30<br>30.18<br>27.93<br>28.15<br>28.02<br>27.93<br>28.11<br>27.56	28.36<br>30.15<br>27.95<br>28.17<br>28.06<br>27.94<br>28.16<br>27.62	28.40<br>30.08<br>27.97<br>28.18<br>28.07<br>27.97<br>28.18<br>27.68	28.43<br>30.01<br>28.00<br>28.17<br>28.06<br>28.01<br>28.19<br>27.73	28.47<br>29.92<br>27.94<br>28.15<br>28.04<br>27.95<br>28.16<br>27.76	~28.4<br>29.82<br>27.91<br>28.11<br>28.00<br>27.92<br>28.13<br>27.79	~28.3<br>29.70<br>27.87<br>28.05<br>27.94<br>27.88<br>28.09<br>27.80	~28.2<br>29.58<br>27.81<br>27.99<br>27.88<br>27.81<br>28.03<br>27.80	28.16<br>29.46<br>27.74<br>27.92<br>27.80<br>27.75<br>27.96<br>27.80	28.09<br>29.32<br>27.66<br>27.85<br>27.73<br>27.68<br>27.88<br>27.78	28.00<br>29.17<br>27.57<br>27.77<br>27.64<br>27.60<br>27.81<br>27.76	27.89<br>29.02<br>27.48<br>27.72<br>27.55<br>27.52<br>27.73<br>27.73	27.78<br>28.86<br>27.38<br>~27.6<br>27.45<br>27.45<br>~27.6<br>27.69
Cu_{12} ring, pz-4-*H(9)*	27.77<br>28.04<br>27.63<br>27.69<br>27.66<br>27.58<br>27.47<br>27.08	27.81<br>28.07<br>27.67<br>27.74<br>27.70<br>27.62<br>27.50<br>27.11	27.84<br>28.13<br>27.71<br>27.77<br>27.74<br>27.65<br>27.54<br>27.14	27.87<br>28.19<br>27.74<br>27.80<br>27.77<br>27.68<br>27.57<br>27.17	27.89<br>28.22<br>27.77<br>27.81<br>27.79<br>27.70<br>27.59<br>27.18	27.90<br>28.25<br>27.78<br>27.82<br>27.79<br>27.70<br>27.60<br>27.20	27.90<br>28.27<br>27.78<br>27.82<br>27.79<br>27.70<br>27.61<br>27.20	27.89<br>28.27<br>27.78<br>27.81<br>27.78<br>27.69<br>27.61<br>27.20	27.87<br>28.27<br>27.77<br>27.79<br>27.76<br>27.67<br>27.59<br>27.19	27.85<br>28.25<br>27.74<br>27.76<br>27.73<br>27.65<br>27.58<br>27.18	27.82<br>28.22<br>27.71<br>27.73<br>27.70<br>27.61<br>27.55<br>27.16	27.78<br>28.19<br>27.67<br>27.69<br>27.65<br>27.55<br>27.53<br>27.13	27.74<br>28.15<br>27.63<br>27.65<br>27.60<br>27.50<br>27.50<br>27.10	27.69<br>28.10<br>27.58<br>27.60<br>27.55<br>27.45<br>27.44<br>27.07
Cu_{12} ring, pz-4-*H(6)*	26.35<br>26.44<br>26.05<br>26.05<br>26.00<br>25.90<br>25.76<br>25.34	26.39<br>26.46<br>26.09<br>26.09<br>26.04<br>25.93<br>25.79<br>25.36	26.42<br>26.51<br>26.12<br>26.11<br>26.06<br>25.95<br>25.82<br>25.38	26.44<br>26.56<br>26.14<br>26.13<br>26.08<br>25.97<br>25.85<br>25.39	26.46<br>26.59<br>26.15<br>26.13<br>26.09<br>25.97<br>25.86<br>25.40	26.47<br>26.60<br>26.16<br>26.13<br>26.09<br>25.97<br>25.86<br>25.39	26.47<br>26.60<br>26.15<br>26.11<br>26.07<br>25.96<br>25.86<br>25.38	26.46<br>26.60<br>26.13<br>26.09<br>26.05<br>25.93<br>25.84<br>25.37	26.43<br>26.59<br>26.11<br>26.06<br>26.02<br>25.90<br>25.82<br>25.34	26.40<br>26.56<br>26.07<br>26.02<br>25.98<br>25.86<br>25.79<br>25.31	26.37<br>26.53<br>26.03<br>25.97<br>25.93<br>25.82<br>25.76<br>25.28	26.33<br>26.48<br>25.98<br>25.92<br>25.88<br>25.77<br>25.73<br>25.24	26.28<br>26.44<br>25.92<br>25.87<br>25.82<br>25.70<br>25.68<br>25.20	26.23<br>26.40<br>25.86<br>25.80<br>25.75<br>25.62<br>25.62<br>25.15
Cu_{12} ring, pz-3,5-H_2*(9)*	22.45<br>22.56<br>22.29<br>22.33<br>22.30<br>22.23<br>22.16<br>22.03	22.47<br>22.57<br>22.31<br>22.35<br>22.31<br>22.25<br>22.18<br>22.06	22.49<br>22.62<br>22.32<br>22.36<br>22.33<br>22.26<br>22.20<br>22.08	22.49<br>22.63<br>22.34<br>22.37<br>22.34<br>22.27<br>22.21<br>22.11	22.50<br>22.65<br>22.34<br>22.37<br>22.34<br>22.27<br>22.21<br>22.12	22.49<br>22.66<br>22.34<br>22.36<br>22.33<br>22.26<br>22.21<br>22.13	22.48<br>22.66<br>22.33<br>22.35<br>22.32<br>22.25<br>22.20<br>22.13	22.46<br>22.65<br>22.32<br>22.33<br>22.30<br>22.23<br>22.19<br>22.12	22.44<br>22.63<br>22.29<br>22.30<br>22.27<br>22.21<br>22.17<br>22.10	22.41<br>22.62<br>22.27<br>22.27<br>22.24<br>22.18<br>22.14<br>22.08	22.37<br>22.59<br>22.23<br>22.24<br>22.20<br>22.14<br>22.12<br>22.05	22.34<br>22.55<br>22.19<br>22.20<br>22.16<br>22.10<br>22.09<br>22.02	22.29<br>22.51<br>22.15<br>22.16<br>22.11<br>22.06<br>22.06<br>21.98	22.25<br>22.45<br>22.10<br>22.11<br>22.06<br>22.01<br>22.01<br>21.94
Cu_{12} ring, pz-3,5-H_2*(6)*	22.43<br>22.56<br>22.16<br>22.18<br>22.13<br>22.05<br>21.94<br>21.92	22.45<br>22.57<br>22.18<br>22.20<br>22.15<br>22.07<br>21.95<br>21.93	22.46<br>22.62<br>22.20<br>22.21<br>22.16<br>22.08<br>21.97<br>21.94	22.46<br>22.63<br>22.20<br>22.21<br>22.16<br>22.08<br>21.97<br>21.95	22.47<br>22.65<br>22.21<br>22.21<br>22.16<br>22.08<br>21.97<br>21.95	22.46<br>22.63<br>22.20<br>22.19<br>22.15<br>22.07<br>21.97<br>21.95	22.45<br>22.63<br>22.19<br>22.18<br>22.13<br>22.05<br>21.96<br>21.94	22.43<br>22.62<br>22.18<br>22.15<br>22.11<br>22.03<br>21.94<br>21.93	22.40<br>22.60<br>22.15<br>22.13<br>22.08<br>22.00<br>21.92<br>21.91	22.37<br>22.57<br>22.12<br>22.09<br>22.04<br>21.96<br>21.89<br>21.89	22.33<br>22.54<br>22.09<br>22.06<br>22.00<br>21.93<br>21.86<br>21.86	22.30<br>22.50<br>22.04<br>22.01<br>21.96<br>21.88<br>21.84<br>21.84	22.25<br>22.45<br>22.00<br>21.97<br>21.90<br>21.84<br>21.80<br>21.80	22.20<br>22.40<br>21.95<br>21.92<br>21.85<br>21.78<br>21.74<br>21.77

Table S54. (continued)

NANOJAR	20–23 °C	30 °C	40 °C	50 °C	60 °C	70 °C	80 °C	90 °C	100 °C	110 °C	120 °C	130 °C	140 °C	150 °C
**Cu_{27} (6+12+9)**														
Cu_{12} ring, O*H(6)*	−29.65<br>−26.18<br>−26.14<br>−25.03<br>−24.63<br>−24.21<br>−23.51<br>−24.57	−29.69<br>−26.24<br>−26.16<br>−25.06<br>−24.65<br>−24.24<br>−23.54<br>−24.55	−29.72<br>−26.33<br>−26.18<br>−25.06<br>−24.65<br>−24.27<br>−23.57<br>−24.52	−29.73<br>−26.47<br>−26.18<br>−25.05<br>−24.65<br>−24.28<br>−23.58<br>−24.47	−29.72<br>−26.57<br>−26.17<br>−25.03<br>−24.63<br>−24.29<br>−23.58<br>−24.41	−29.69<br>−26.62<br>−26.15<br>−25.00<br>−24.60<br>−24.28<br>−23.57<br>−24.35	−29.64<br>−26.68<br>−26.13<br>−24.96<br>−24.56<br>−24.26<br>−23.56<br>−24.28	−29.66<br>−26.68<br>−26.09<br>−24.90<br>−24.50<br>−24.23<br>−23.53<br>−24.20	−29.50<br>−26.65<br>−26.03<br>−24.84<br>−24.45<br>−24.18<br>−23.50<br>−24.11	−29.40<br>−26.66<br>−25.97<br>−24.75<br>−24.38<br>−24.13<br>−23.47<br>−24.01	−29.30<br>−26.51<br>−25.90<br>−24.68<br>−24.30<br>−24.07<br>−23.42<br>−23.92	−29.14<br>−26.50<br>−25.82<br>−24.60<br>−24.23<br>−24.00<br>−23.36<br>−23.82	~−29.0<br>−26.50<br>−25.72<br>−24.51<br>−24.14<br>−23.92<br>−23.31<br>−23.73	~−28.9<br>−26.37<br>−25.62<br>−24.42<br>−24.05<br>−23.85<br>−23.26<br>−23.62
Cu_{12} ring, O*H(9)*	−32.50<br>−42.02<br>−35.29<br>−34.94<br>−34.76<br>−34.55<br>−31.71<br>−34.79	−32.77<br>−42.05<br>−35.34<br>−35.03<br>−34.89<br>−34.64<br>−31.84<br>−34.95	−33.05<br>−41.99<br>−35.36<br>−35.09<br>−34.96<br>−34.74<br>−32.03<br>−35.05	−33.25<br>−41.93<br>−35.35<br>~−35.1<br>−35.00<br>−34.79<br>−32.24<br>−35.13	−33.41<br>−41.85<br>−35.29<br>−35.00<br>−34.98<br>−34.79<br>−32.45<br>−35.16	−33.53<br>−41.75<br>−35.18<br>−34.93<br>−34.91<br>−34.75<br>−32.66<br>−35.15	−33.63<br>−41.72<br>−35.07<br>−34.84<br>−34.82<br>−34.69<br>−32.89<br>−35.08	−33.76<br>−41.46<br>−34.94<br>−34.71<br>−34.70<br>−34.58<br>−33.11<br>~−35.0	−33.69<br>−41.24<br>−34.79<br>−34.57<br>−34.57<br>−34.46<br>−33.33<br>~−35.0	−33.71<br>−41.08<br>−34.63<br>−34.39<br>−34.42<br>−34.31<br>−33.56<br>−34.75	−33.70<br>−40.82<br>−34.46<br>−34.24<br>−34.24<br>−34.11<br>−33.79<br>−34.58	−33.66<br>−40.74<br>−34.29<br>−34.06<br>−34.07<br>−33.88<br>~−34.0<br>−34.40	−33.61<br>−40.34<br>−34.11<br>−33.86<br>−33.86<br>−33.61<br>−34.16<br>−34.23	−33.55<br>−40.11<br>−33.93<br>−33.66<br>−33.65<br>−33.39<br>−34.37<br>~−34.0
Cu_{6} ring, O*H*	− 41.25<br>−44.39<br>−41.30<br>−41.15<br>~−40.98<br>−40.40<br>−34.14<br>−38.60	−41.30<br>−44.40<br>−41.30<br>−41.10<br>~−40.92<br>−40.36<br>−34.28<br>~−38.5	−41.34<br>−44.38<br>−41.25<br>−41.03<br>~−40.86<br>−40.29<br>−34.41<br>−38.39	−41.35<br>−44.28<br>−41.18<br>−40.92<br>~−40.79<br>−40.19<br>−34.52<br>−38.31	−41.35<br>~−44.2<br>−41.08<br>−40.79<br>~−40.75<br>−40.07<br>−34.55<br>−38.18	−41.33<br>~−44.0<br>−40.94<br>−40.63<br>~−40.49<br>−39.91<br>−34.53<br>−38.04	−41.28<br>~−43.8<br>−40.78<br>−40.45<br>−40.30<br>−39.74<br>−34.46<br>−37.88	−41.21<br>~−43.7<br>−40.60<br>−40.24<br>−40.08<br>−39.55<br>−34.37<br>−37.69	−41.11<br>~−43.6<br>−40.40<br>−40.01<br>−39.85<br>−39.33<br>−34.25<br>−37.49	−41.00<br>~−43.4<br>−40.18<br>−39.75<br>−39.61<br>−39.08<br>−34.11<br>−37.27	−40.86<br>−42.98<br>−39.93<br>−39.50<br>−39.34<br>−38.82<br>−33.96<br>−37.04	−40.72<br>−42.72<br>−39.68<br>−39.23<br>−39.07<br>−38.53<br>~−33.8<br>−36.80	−40.59<br>−42.45<br>−39.41<br>−38.95<br>−38.77<br>−38.23<br>−33.63<br>~−36.5	−40.35<br>−42.21<br>−39.14<br>−38.67<br>−38.47<br>−37.90<br>−33.41<br>~−36.3
Cu_{9} ring, O*H*	−68.08<br>−52.95<br>−45.10<br>−44.35<br>~−40.98<br>−41.33<br>−39.68<br>−38.86	−66.96<br>−52.97<br>−45.42<br>−44.22<br>~−40.92<br>−41.30<br>−39.68<br>~−38.5	−65.63<br>−52.91<br>−45.80<br>−44.18<br>~−40.86<br>−41.28<br>−39.65<br>−38.06	−64.38<br>−52.63<br>−46.10<br>−44.16<br>~−40.79<br>−41.27<br>−39.58<br>−37.61	−63.19<br>~−52.3<br>−46.37<br>−44.13<br>~−40.75<br>−41.28<br>−39.50<br>−37.20	−62.04<br>~−52.0<br>−46.57<br>−44.09<br>−40.71<br>−41.30<br>−39.38<br>−36.79	−60.95<br>~−51.8<br>−46.70<br>−44.02<br>−40.68<br>−41.30<br>−39.25<br>−36.40	−59.91<br>~−51.2<br>−46.75<br>−43.94<br>−40.65<br>−41.30<br>−39.10<br>−36.03	−58.90<br>−50.63<br>−46.73<br>−43.82<br>−40.60<br>−41.29<br>−38.93<br>−35.65	−57.90<br>−50.14<br>−46.65<br>−43.66<br>−40.55<br>−41.27<br>−38.75<br>~−35.3	−56.98<br>−49.64<br>−46.51<br>−43.51<br>−40.48<br>−41.24<br>−38.53<br>~−34.9	−56.07<br>−49.11<br>−46.30<br>−43.31<br>−40.40<br>−41.22<br>−38.30<br>~−34.5	−55.21<br>−48.63<br>−46.05<br>−43.09<br>−40.29<br>−41.15<br>−38.06<br>~−34.1	−54.36<br>−48.11<br>−45.76<br>−42.85<br>−40.18<br>−41.06<br>−37.81<br>~−33.8
**Cu_{28} (6+12+10)**														
Cu_{10} ring, pz-4-*H*	–<br>40.27<br>–<br>–<br>–<br>–<br>36.66<br>–	–<br>39.99<br>–<br>–<br>–<br>–<br>36.39<br>–	–<br>39.46<br>–<br>–<br>–<br>–<br>36.02<br>–	–<br>38.94<br>–<br>–<br>–<br>–<br>35.63<br>–	–<br>38.42<br>–<br>–<br>–<br>–<br>–<br>–	–<br>37.92<br>–<br>–<br>–<br>–<br>–<br>–	–<br>37.43<br>–<br>–<br>–<br>–<br>–<br>–	–<br>36.94<br>–<br>–<br>–<br>–<br>–<br>–	–<br>36.47<br>–<br>–<br>–<br>–<br>–<br>–	–<br>36.01<br>–<br>–<br>–<br>–<br>–<br>–	–<br>35.56<br>–<br>–<br>–<br>–<br>–<br>–	–<br>35.12<br>–<br>–<br>–<br>–<br>–<br>–	–<br>34.69<br>–<br>–<br>–<br>–<br>–<br>–	–<br>34.28<br>–<br>–<br>–<br>–<br>–<br>–
Cu_{10} ring, pz-3,5-H_2	–<br>34.50<br>–<br>–<br>–<br>–<br>31.51<br>–	–<br>34.27<br>–<br>–<br>–<br>–<br>31.28<br>–	–<br>33.81<br>–<br>–<br>–<br>–<br>30.97<br>–	–<br>33.37<br>–<br>–<br>–<br>–<br>30.65<br>–	–<br>32.94<br>–<br>–<br>–<br>–<br>–<br>–	–<br>32.51<br>–<br>–<br>–<br>–<br>–<br>–	–<br>32.08<br>–<br>–<br>–<br>–<br>–<br>–	–<br>31.67<br>–<br>–<br>–<br>–<br>–<br>–	–<br>31.27<br>–<br>–<br>–<br>–<br>–<br>–	–<br>30.89<br>–<br>–<br>–<br>–<br>–<br>–	–<br>30.51<br>–<br>–<br>–<br>–<br>–<br>–	–<br>30.15<br>–<br>–<br>–<br>–<br>–<br>–	–<br>29.81<br>–<br>–<br>–<br>–<br>–<br>–	–<br>29.41<br>–<br>–<br>–<br>–<br>–<br>–
Cu_{6} ring, pz-4-*H*	–<br>33.61<br>–<br>–<br>–<br>–<br>31.90<br>–	–<br>33.63<br>–<br>–<br>–<br>–<br>31.98<br>–	–<br>33.67<br>–<br>–<br>–<br>–<br>32.06<br>–	–<br>33.67<br>–<br>–<br>–<br>–<br>32.12<br>–	–<br>33.64<br>–<br>–<br>–<br>–<br>–<br>–	–<br>33.60<br>–<br>–<br>–<br>–<br>–<br>–	–<br>33.52<br>–<br>–<br>–<br>–<br>–<br>–	–<br>33.41<br>–<br>–<br>–<br>–<br>–<br>–	–<br>33.31<br>–<br>–<br>–<br>–<br>–<br>–	–<br>33.17<br>–<br>–<br>–<br>–<br>–<br>–	–<br>33.03<br>–<br>–<br>–<br>–<br>–<br>–	–<br>32.86<br>–<br>–<br>–<br>–<br>–<br>–	–<br>32.70<br>–<br>–<br>–<br>–<br>–<br>–	–<br>32.54<br>–<br>–<br>–<br>–<br>–<br>–
Cu_{6} ring, pz-3,5-H_2	–<br>30.77<br>–<br>–<br>–<br>–<br>28.74<br>–	–<br>30.79<br>–<br>–<br>–<br>–<br>28.80<br>–	–<br>30.80<br>–<br>–<br>–<br>–<br>28.87<br>–	–<br>30.79<br>–<br>–<br>–<br>–<br>28.90<br>–	–<br>30.74<br>–<br>–<br>–<br>–<br>–<br>–	–<br>30.68<br>–<br>–<br>–<br>–<br>–<br>–	–<br>30.59<br>–<br>–<br>–<br>–<br>–<br>–	–<br>30.48<br>–<br>–<br>–<br>–<br>–<br>–	–<br>30.37<br>–<br>–<br>–<br>–<br>–<br>–	–<br>30.24<br>–<br>–<br>–<br>–<br>–<br>–	–<br>30.10<br>–<br>–<br>–<br>–<br>–<br>–	–<br>29.92<br>–<br>–<br>–<br>–<br>–<br>–	–<br>29.61<br>–<br>–<br>–<br>–<br>–<br>–	–<br>29.41<br>–<br>–<br>–<br>–<br>–<br>–
Cu_{12} ring, pz-4-*H(10)*	–<br>28.97<br>–<br>–<br>–<br>–<br>28.51<br>–	–<br>29.00<br>–<br>–<br>–<br>–<br>28.55<br>–	–<br>29.06<br>–<br>–<br>–<br>–<br>28.60<br>–	–<br>29.09<br>–<br>–<br>–<br>–<br>28.63<br>–	–<br>29.12<br>–<br>–<br>–<br>–<br>–<br>–	–<br>29.14<br>–<br>–<br>–<br>–<br>–<br>–	–<br>29.14<br>–<br>–<br>–<br>–<br>–<br>–	–<br>29.12<br>–<br>–<br>–<br>–<br>–<br>–	–<br>29.11<br>–<br>–<br>–<br>–<br>–<br>–	–<br>29.08<br>–<br>–<br>–<br>–<br>–<br>–	–<br>29.03<br>–<br>–<br>–<br>–<br>–<br>–	–<br>28.98<br>–<br>–<br>–<br>–<br>–<br>–	–<br>28.92<br>–<br>–<br>–<br>–<br>–<br>–	–<br>28.86<br>–<br>–<br>–<br>–<br>–<br>–
Cu_{12} ring, pz-4-*H(6)*	–<br>27.48<br>–<br>–<br>–<br>–<br>26.90<br>–	–<br>27.50<br>–<br>–<br>–<br>–<br>26.93<br>–	–<br>27.56<br>–<br>–<br>–<br>–<br>26.97<br>–	–<br>27.60<br>–<br>–<br>–<br>–<br>26.99<br>–	–<br>27.61<br>–<br>–<br>–<br>–<br>–<br>–	–<br>27.63<br>–<br>–<br>–<br>–<br>–<br>–	–<br>27.61<br>–<br>–<br>–<br>–<br>–<br>–	–<br>27.60<br>–<br>–<br>–<br>–<br>–<br>–	–<br>27.56<br>–<br>–<br>–<br>–<br>–<br>–	–<br>27.52<br>–<br>–<br>–<br>–<br>–<br>–	–<br>27.47<br>–<br>–<br>–<br>–<br>–<br>–	–<br>27.41<br>–<br>–<br>–<br>–<br>–<br>–	–<br>27.35<br>–<br>–<br>–<br>–<br>–<br>–	–<br>~27.3<br>–<br>–<br>–<br>–<br>–<br>–

Table S54. (continued)

NANOJAR	20–23 °C	30 °C	40 °C	50 °C	60 °C	70 °C	80 °C	90 °C	100 °C	110 °C	120 °C	130 °C	140 °C	150 °C
**Cu_{28} (6+12+10)**														
Cu_{12} ring, pz-3,5-H_2*(10)*	–<br>23.40<br>–<br>–<br>–<br>–<br>22.97<br>–	–<br>23.41<br>–<br>–<br>–<br>–<br>22.99<br>–	–<br>23.43<br>–<br>–<br>–<br>–<br>23.00<br>–	–<br>23.44<br>–<br>–<br>–<br>–<br>23.01<br>–	–<br>23.46<br>–<br>–<br>–<br>–<br>–<br>–	–<br>23.44<br>–<br>–<br>–<br>–<br>–<br>–	–<br>23.43<br>–<br>–<br>–<br>–<br>–<br>–	–<br>23.40<br>–<br>–<br>–<br>–<br>–<br>–	–<br>23.37<br>–<br>–<br>–<br>–<br>–<br>–	–<br>23.32<br>–<br>–<br>–<br>–<br>–<br>–	–<br>23.27<br>–<br>–<br>–<br>–<br>–<br>–	–<br>23.23<br>–<br>–<br>–<br>–<br>–<br>–	–<br>23.17<br>–<br>–<br>–<br>–<br>–<br>–	–<br>23.09<br>–<br>–<br>–<br>–<br>–<br>–
Cu_{12} ring, pz-3,5-H_2*(6)*	–<br>23.23<br>–<br>–<br>–<br>–<br>22.93<br>–	–<br>23.24<br>–<br>–<br>–<br>–<br>22.95<br>–	–<br>23.27<br>–<br>–<br>–<br>–<br>22.96<br>–	–<br>23.29<br>–<br>–<br>–<br>–<br>22.97<br>–	–<br>23.29<br>–<br>–<br>–<br>–<br>–<br>–	–<br>23.29<br>–<br>–<br>–<br>–<br>–<br>–	–<br>23.27<br>–<br>–<br>–<br>–<br>–<br>–	–<br>23.24<br>–<br>–<br>–<br>–<br>–<br>–	–<br>23.23<br>–<br>–<br>–<br>–<br>–<br>–	–<br>23.18<br>–<br>–<br>–<br>–<br>–<br>–	–<br>23.14<br>–<br>–<br>–<br>–<br>–<br>–	–<br>23.09<br>–<br>–<br>–<br>–<br>–<br>–	–<br>23.05<br>–<br>–<br>–<br>–<br>–<br>–	–<br>22.98<br>–<br>–<br>–<br>–<br>–<br>–
Cu_{12} ring, O*H(6)*	–<br>−32.98<br>–<br>–<br>–<br>–<br>−30.64<br>–	–<br>−32.91<br>–<br>–<br>–<br>–<br>−30.62<br>–	–<br>−32.74<br>–<br>–<br>–<br>–<br>−30.59<br>–	–<br>−32.57<br>–<br>–<br>–<br>–<br>−30.69<br>–	–<br>−32.42<br>–<br>–<br>–<br>–<br>–<br>–	–<br>−32.19<br>–<br>–<br>–<br>–<br>–<br>–	–<br>−32.01<br>–<br>–<br>–<br>–<br>–<br>–	–<br>−31.79<br>–<br>–<br>–<br>–<br>–<br>–	–<br>−31.53<br>–<br>–<br>–<br>–<br>–<br>–	–<br>−31.32<br>–<br>–<br>–<br>–<br>–<br>–	–<br>−31.06<br>–<br>–<br>–<br>–<br>–<br>–	–<br>−30.79<br>–<br>–<br>–<br>–<br>–<br>–	–<br>−30.53<br>–<br>–<br>–<br>–<br>–<br>–	–<br>~−30.2<br>–<br>–<br>–<br>–<br>–<br>–
Cu_{12} ring, O*H(10)*	–<br>−43.47<br>–<br>–<br>–<br>–<br>–<br>–	–<br>−43.56<br>–<br>–<br>–<br>–<br>–<br>–	–<br>−43.69<br>–<br>–<br>–<br>–<br>–<br>–	–<br>−43.78<br>–<br>–<br>–<br>–<br>–<br>–	–<br>−43.81<br>–<br>–<br>–<br>–<br>–<br>–	–<br>−43.84<br>–<br>–<br>–<br>–<br>–<br>–	–<br>−43.85<br>–<br>–<br>–<br>–<br>–<br>–	–<br>−43.76<br>–<br>–<br>–<br>–<br>–<br>–	–<br>−43.61<br>–<br>–<br>–<br>–<br>–<br>–	–<br>−43.41<br>–<br>–<br>–<br>–<br>–<br>–	–<br>~−43.2<br>–<br>–<br>–<br>–<br>–<br>–	–<br>~−43.1<br>–<br>–<br>–<br>–<br>–<br>–	–<br>~−43.0<br>–<br>–<br>–<br>–<br>–<br>–	–<br>~−42.9<br>–<br>–<br>–<br>–<br>–<br>–
Cu_6 ring, O*H*	–<br>−51.49<br>–<br>–<br>–<br>–<br>−42.36<br>–	–<br>−51.66<br>–<br>–<br>–<br>–<br>−42.45<br>–	–<br>−51.95<br>–<br>–<br>–<br>–<br>−42.58<br>–	–<br>−52.13<br>–<br>–<br>–<br>–<br>−42.70<br>–	–<br>−52.27<br>–<br>–<br>–<br>–<br>–<br>–	–<br>−52.16<br>–<br>–<br>–<br>–<br>–<br>–	–<br>~−52.0<br>–<br>–<br>–<br>–<br>–<br>–	–<br>−52.07<br>–<br>–<br>–<br>–<br>–<br>–	–<br>−51.88<br>–<br>–<br>–<br>–<br>–<br>–	–<br>−51.66<br>–<br>–<br>–<br>–<br>–<br>–	–<br>−51.35<br>–<br>–<br>–<br>–<br>–<br>–	–<br>−51.08<br>–<br>–<br>–<br>–<br>–<br>–	–<br>−50.69<br>–<br>–<br>–<br>–<br>–<br>–	–<br>−50.27<br>–<br>–<br>–<br>–<br>–<br>–
Cu_{10} ring, O*H*	–<br>−57.75<br>–<br>–<br>–<br>–<br>−44.76<br>–	–<br>−57.18<br>–<br>–<br>–<br>–<br>−44.29<br>–	–<br>−56.10<br>–<br>–<br>–<br>–<br>−43.63<br>–	–<br>−55.05<br>–<br>–<br>–<br>–<br>−42.88<br>–	–<br>−53.95<br>–<br>–<br>–<br>–<br>–<br>–	–<br>−52.94<br>–<br>–<br>–<br>–<br>–<br>–	–<br>~−51.9<br>–<br>–<br>–<br>–<br>–<br>–	–<br>~−51.0<br>–<br>–<br>–<br>–<br>–<br>–	–<br>−50.02<br>–<br>–<br>–<br>–<br>–<br>–	–<br>−49.09<br>–<br>–<br>–<br>–<br>–<br>–	–<br>−48.19<br>–<br>–<br>–<br>–<br>–<br>–	–<br>−47.29<br>–<br>–<br>–<br>–<br>–<br>–	–<br>−46.48<br>–<br>–<br>–<br>–<br>–<br>–	–<br>−45.67<br>–<br>–<br>–<br>–<br>–<br>–

Table S54. (continued)

NANOJAR	20–23 °C	30 °C	40 °C	50 °C	60 °C	70 °C	80 °C	90 °C	100 °C	110 °C	120 °C	130 °C	140 °C	150 °C
**Cu_{29} (7+13+9)**														
Cu_9 ring, pz-4-*H*	33.87<br>31.84<br>29.07<br>29.27<br>29.03<br>29.23<br>29.11<br>28.04	33.68<br>31.75<br>29.00<br>29.14<br>28.90<br>29.09<br>29.03<br>27.94	33.44<br>31.56<br>28.91<br>28.99<br>28.77<br>28.93<br>28.92<br>27.83	33.19<br>31.38<br>28.82<br>28.84<br>28.63<br>28.76<br>28.80<br>27.71	32.95<br>31.18<br>28.71<br>28.69<br>28.49<br>28.60<br>28.68<br>27.60	32.70<br>30.98<br>28.61<br>28.54<br>28.35<br>28.44<br>28.56<br>27.49	32.46<br>30.78<br>28.50<br>28.39<br>28.21<br>28.29<br>28.44<br>27.38	32.23<br>30.59<br>28.39<br>28.24<br>28.07<br>–<br>28.31<br>27.27	31.99<br>30.40<br>28.29<br>28.08<br>27.92<br>–<br>28.20<br>27.16	31.76<br>30.21<br>28.17<br>~27.9<br>~27.8<br>–<br>–<br>27.05	31.51<br>~30.00<br>28.04<br>~27.8<br>~27.6<br>–<br>–<br>26.94	31.29<br>~29.85<br>27.91<br>~27.7<br>27.53<br>–<br>–<br>26.84	31.05<br>~29.67<br>27.80<br>27.54<br>27.39<br>–<br>–<br>26.73	30.81<br>29.49<br>27.67<br>27.40<br>27.26<br>–<br>–<br>26.62
Cu_9 ring, pz-3,5-*H_2*	29.79<br>28.63<br>26.15<br>26.12<br>25.73<br>26.06<br>–<br>~25.3	29.62<br>28.56<br>26.10<br>26.02<br>25.64<br>26.10<br>–<br>25.25	29.42<br>28.39<br>26.00<br>25.90<br>25.55<br>26.13<br>–<br>25.16	29.21<br>28.21<br>25.91<br>25.78<br>25.45<br>26.15<br>–<br>25.13	29.00<br>28.04<br>25.82<br>25.66<br>25.35<br>26.16<br>–<br>25.10	28.79<br>27.85<br>25.72<br>25.53<br>25.25<br>26.17<br>–<br>25.06	28.59<br>27.67<br>25.62<br>25.41<br>25.14<br>26.16<br>–<br>25.02	~28.3<br>27.49<br>25.52<br>25.28<br>25.03<br>–<br>–<br>24.97	~28.2<br>27.31<br>25.41<br>25.15<br>24.92<br>–<br>–<br>24.92	27.96<br>27.13<br>25.30<br>25.03<br>24.81<br>–<br>–<br>24.86	~27.8<br>26.95<br>25.18<br>24.90<br>24.70<br>–<br>–<br>24.80	27.57<br>26.78<br>25.06<br>24.77<br>24.58<br>–<br>–<br>24.72	27.36<br>~26.59<br>24.95<br>24.66<br>24.46<br>–<br>–<br>24.65	27.16<br>~26.45<br>24.83<br>24.54<br>24.34<br>–<br>–<br>24.57
Cu_7 ring, pz-4-*H*	30.81<br>31.55<br>31.08<br>30.83<br>–<br>30.86<br>30.97<br>30.15	30.84<br>31.53<br>31.08<br>30.89<br>–<br>30.89<br>30.97<br>30.18	30.85<br>31.50<br>31.06<br>30.93<br>–<br>30.91<br>30.97<br>30.21	30.85<br>31.47<br>31.04<br>30.95<br>–<br>30.91<br>30.95<br>30.23	30.84<br>31.41<br>31.00<br>30.96<br>–<br>30.90<br>30.93<br>30.19	30.81<br>31.35<br>30.96<br>30.95<br>–<br>~30.9<br>30.89<br>30.15	30.76<br>31.29<br>30.90<br>30.92<br>–<br>30.85<br>30.84<br>30.10	30.71<br>31.21<br>30.83<br>30.88<br>–<br>–<br>30.79<br>30.05	30.65<br>31.12<br>30.76<br>30.83<br>–<br>–<br>30.72<br>29.99	30.58<br>31.03<br>30.69<br>30.77<br>–<br>–<br>30.65<br>29.93	30.49<br>~30.94<br>30.58<br>30.70<br>–<br>–<br>30.58<br>29.86	30.42<br>~30.86<br>30.49<br>30.62<br>–<br>–<br>30.51<br>29.79	30.32<br>~30.67<br>30.38<br>30.54<br>–<br>–<br>30.43<br>29.71	30.22<br>~30.48<br>30.28<br>30.45<br>–<br>–<br>30.31<br>29.63
Cu_7 ring, pz-3,5-*H_2*	27.41<br>28.16<br>27.32<br>27.13<br>–<br>26.54<br>–<br>26.53	27.43<br>28.16<br>27.33<br>27.18<br>–<br>26.53<br>–<br>26.51	27.44<br>28.13<br>27.32<br>27.23<br>–<br>26.52<br>–<br>26.51	27.43<br>28.11<br>27.29<br>27.23<br>–<br>26.50<br>–<br>26.47	27.42<br>~28.08<br>27.28<br>27.24<br>–<br>26.47<br>–<br>26.46	27.38<br>28.02<br>27.24<br>27.23<br>–<br>26.44<br>–<br>26.41	27.35<br>~27.97<br>27.20<br>27.21<br>–<br>26.40<br>–<br>26.38	27.30<br>~27.90<br>27.14<br>~27.2<br>–<br>–<br>–<br>26.33	27.24<br>27.82<br>27.08<br>27.12<br>–<br>–<br>–<br>26.28	27.18<br>27.73<br>27.02<br>27.07<br>–<br>–<br>–<br>26.22	27.10<br>27.65<br>26.92<br>27.00<br>–<br>–<br>–<br>26.16	27.02<br>27.56<br>26.83<br>26.93<br>–<br>–<br>–<br>26.09	26.93<br>~27.48<br>26.74<br>26.86<br>–<br>–<br>–<br>26.01	26.88<br>27.35<br>26.65<br>26.78<br>–<br>–<br>–<br>25.93
Cu_{13} ring, pz-4-*H*	27.12<br>27.79<br>26.99<br>27.18<br>27.07<br>26.98<br>26.96<br>26.76	27.18<br>27.82<br>27.03<br>27.23<br>27.12<br>27.03<br>27.00<br>26.80	27.24<br>27.87<br>27.09<br>27.27<br>27.17<br>27.07<br>27.06<br>26.84	27.30<br>27.92<br>27.14<br>27.30<br>27.21<br>27.11<br>27.11<br>26.87	27.34<br>27.95<br>27.18<br>27.32<br>27.23<br>27.13<br>27.14<br>26.89	27.38<br>27.96<br>27.21<br>27.33<br>27.25<br>27.15<br>27.16<br>26.91	27.40<br>27.98<br>27.23<br>27.33<br>27.25<br>27.16<br>27.18<br>26.92	27.42<br>27.98<br>27.24<br>27.33<br>27.25<br>–<br>27.18<br>26.92	27.43<br>27.96<br>27.24<br>27.32<br>27.24<br>–<br>27.17<br>26.91	27.42<br>27.95<br>27.24<br>27.30<br>27.22<br>–<br>27.16<br>26.90	27.41<br>27.93<br>27.22<br>27.27<br>27.20<br>–<br>27.14<br>26.89	27.40<br>27.90<br>27.20<br>27.24<br>27.16<br>–<br>27.13<br>26.86	27.37<br>27.87<br>27.17<br>27.20<br>27.12<br>–<br>27.10<br>26.83	27.33<br>~27.81<br>27.13<br>27.16<br>27.08<br>–<br>27.05<br>26.80
Cu_{13} ring, pz-3,5-*H_2*	22.02<br>22.51<br>21.98<br>~22.16<br>22.01<br>21.96<br>21.86<br>21.63	22.05<br>22.53<br>22.00<br>~22.17<br>22.04<br>21.98<br>21.89<br>21.64	22.08<br>22.54<br>22.02<br>~22.19<br>22.06<br>22.00<br>21.92<br>21.64	22.11<br>22.56<br>22.04<br>~22.19<br>22.08<br>22.02<br>21.94<br>21.65	22.13<br>22.57<br>22.06<br>~22.19<br>22.08<br>22.02<br>21.96<br>21.65	22.14<br>22.57<br>22.06<br>~22.18<br>22.08<br>22.02<br>21.97<br>21.64	22.15<br>22.56<br>22.07<br>~22.17<br>22.08<br>22.02<br>21.96<br>21.63	22.15<br>22.56<br>22.06<br>~22.15<br>22.06<br>–<br>21.95<br>21.61	22.14<br>22.53<br>22.05<br>~22.13<br>22.04<br>–<br>21.94<br>21.59	22.13<br>22.50<br>22.03<br>~22.09<br>22.02<br>–<br>21.92<br>21.57	22.11<br>22.47<br>22.00<br>~22.06<br>22.00<br>–<br>21.90<br>21.54	22.09<br>22.44<br>21.97<br>~22.01<br>21.96<br>–<br>21.88<br>21.51	22.06<br>22.39<br>21.94<br>~21.97<br>21.90<br>–<br>21.85<br>21.48	22.02<br>22.34<br>21.90<br>~21.92<br>21.85<br>–<br>21.80<br>21.44
Cu_{13} ring, O*H*	−29.32<br>−30.24<br>−27.78<br>−27.79<br>−26.38<br>–<br>–<br>−27.18	−29.37<br>−30.25<br>−27.82<br>−27.83<br>−26.51<br>–<br>–<br>−27.17	−29.42<br>−30.24<br>−27.83<br>−27.86<br>−26.62<br>–<br>–<br>−27.16	−29.47<br>−30.24<br>−27.87<br>−27.87<br>−26.72<br>–<br>–<br>−27.13	−29.53<br>−30.29<br>−27.91<br>−27.87<br>−26.80<br>–<br>–<br>−27.11	−29.56<br>−30.28<br>−27.92<br>−27.87<br>−26.86<br>–<br>–<br>−27.07	~−29.6<br>−30.27<br>−27.93<br>−27.85<br>−26.89<br>–<br>–<br>−27.03	~−29.6<br>−30.21<br>−27.93<br>−27.81<br>−26.91<br>–<br>–<br>−26.99	~−29.5<br>−30.13<br>−27.92<br>−27.76<br>~−26.9<br>–<br>–<br>−26.94	~−29.4<br>~−30.1<br>−27.89<br>−27.70<br>−26.79<br>–<br>–<br>−26.89	~−29.3<br>~−30.0<br>−27.86<br>−27.66<br>−26.63<br>–<br>–<br>−26.83	–<br>~−29.9<br>−27.81<br>−27.58<br>−26.50<br>–<br>–<br>−26.77	–<br>~−29.8<br>−27.76<br>−27.49<br>−26.39<br>–<br>–<br>−26.69	–<br>~−29.7<br>−27.70<br>−27.42<br>−26.26<br>–<br>–<br>−26.62
Cu_7 ring, O*H*	−36.88<br>−42.22<br>−36.25<br>−35.69<br>–<br>–<br>–<br>–	−36.83<br>−42.13<br>−36.18<br>−35.46<br>–<br>–<br>–<br>–	−36.83<br>−42.05<br>−36.06<br>~−35.2<br>–<br>–<br>–<br>–	−36.65<br>−41.93<br>−35.93<br>~−35.1<br>–<br>–<br>–<br>–	−36.53<br>−41.76<br>−35.79<br>~−34.8<br>–<br>–<br>–<br>–	−36.42<br>−41.61<br>−35.65<br>−34.59<br>–<br>–<br>–<br>–	−36.27<br>−41.46<br>−35.50<br>−34.35<br>–<br>–<br>–<br>–	−36.13<br>−41.29<br>−35.35<br>−34.09<br>–<br>–<br>–<br>–	−35.96<br>−41.11<br>−35.20<br>−33.84<br>–<br>–<br>–<br>–	−35.81<br>−40.94<br>−35.03<br>−33.57<br>–<br>–<br>–<br>–	−35.64<br>−40.77<br>−34.87<br>−33.35<br>–<br>–<br>–<br>–	–<br>~−40.6<br>−34.70<br>−33.10<br>–<br>–<br>–<br>–	–<br>~−40.5<br>−34.54<br>−32.86<br>–<br>–<br>–<br>–	–<br>~−40.3<br>−34.39<br>−32.65<br>–<br>–<br>–<br>–
Cu_9 ring, O*H*	−47.36<br>−45.81<br>−38.84<br>–<br>–<br>–<br>–<br>–	−46.88<br>−45.53<br>−38.60<br>–<br>–<br>–<br>–<br>–	−46.28<br>−45.04<br>−38.29<br>–<br>–<br>–<br>–<br>–	−45.68<br>−44.57<br>−37.98<br>–<br>–<br>–<br>–<br>–	−45.09<br>−44.07<br>−37.66<br>–<br>–<br>–<br>–<br>–	−44.51<br>−43.60<br>−37.35<br>–<br>–<br>–<br>–<br>–	−43.95<br>−43.12<br>−37.03<br>–<br>–<br>–<br>–<br>–	−43.40<br>−42.65<br>−36.72<br>–<br>–<br>–<br>–<br>–	−42.85<br>−42.20<br>−36.41<br>–<br>–<br>–<br>–<br>–	−42.31<br>−41.76<br>−36.10<br>–<br>–<br>–<br>–<br>–	−41.79<br>−41.35<br>−35.79<br>–<br>–<br>–<br>–<br>–	–<br>~−40.9<br>−35.48<br>–<br>–<br>–<br>–<br>–	–<br>~−40.5<br>−35.18<br>–<br>–<br>–<br>–<br>–	–<br>~−40.1<br>−34.89<br>–<br>–<br>–<br>–<br>–

Table S54. (continued)

NANOJAR	20–23 °C	30 °C	40 °C	50 °C	60 °C	70 °C	80 °C	90 °C	100 °C	110 °C	120 °C	130 °C	140 °C	150 °C
**Cu_{29} (8+13+8)**														
Cu_8 ring, pz-4-*H*	28.52<br>28.33<br>27.75<br>–<br>–<br>–<br>–<br>–	28.51<br>28.31<br>27.79<br>–<br>–<br>–<br>–<br>–	28.49<br>28.30<br>27.82<br>–<br>–<br>–<br>–<br>–	28.47<br>28.28<br>27.85<br>–<br>–<br>–<br>–<br>–	28.44<br>28.25<br>27.87<br>–<br>–<br>–<br>–<br>–	28.41<br>28.22<br>27.88<br>–<br>–<br>–<br>–<br>–	28.37<br>28.19<br>27.88<br>–<br>–<br>–<br>–<br>–	28.33<br>28.14<br>27.87<br>–<br>–<br>–<br>–<br>–	28.28<br>28.11<br>27.86<br>–<br>–<br>–<br>–<br>–	28.23<br>28.05<br>27.84<br>–<br>–<br>–<br>–<br>–	28.18<br>28.01<br>27.81<br>–<br>–<br>–<br>–<br>–	28.13<br>27.95<br>27.77<br>–<br>–<br>–<br>–<br>–	28.06<br>27.89<br>27.72<br>–<br>–<br>–<br>–<br>–	27.99<br>27.82<br>27.67<br>–<br>–<br>–<br>–<br>–
Cu_8 ring, pz-3,5-H_2	25.58<br>25.63<br>23.84<br>–<br>–<br>–<br>–<br>–	25.58<br>25.63<br>23.87<br>–<br>–<br>–<br>–<br>–	25.56<br>25.61<br>23.90<br>–<br>–<br>–<br>–<br>–	25.54<br>25.60<br>23.92<br>–<br>–<br>–<br>–<br>–	25.52<br>25.56<br>23.94<br>–<br>–<br>–<br>–<br>–	25.49<br>25.55<br>23.95<br>–<br>–<br>–<br>–<br>–	25.45<br>25.52<br>23.95<br>–<br>–<br>–<br>–<br>–	25.41<br>25.47<br>23.94<br>–<br>–<br>–<br>–<br>–	25.37<br>25.43<br>23.92<br>–<br>–<br>–<br>–<br>–	25.32<br>25.38<br>23.91<br>–<br>–<br>–<br>–<br>–	25.27<br>25.34<br>23.88<br>–<br>–<br>–<br>–<br>–	25.22<br>25.27<br>23.85<br>–<br>–<br>–<br>–<br>–	25.15<br>25.23<br>23.81<br>–<br>–<br>–<br>–<br>–	25.08<br>25.15<br>23.77<br>–<br>–<br>–<br>–<br>–
Cu_{13} ring, pz-4-*H*	25.91<br>26.59<br>~26.0<br>–<br>–<br>–<br>–<br>–	26.00<br>26.65<br>~26.1<br>–<br>–<br>–<br>–<br>–	26.11<br>26.74<br>26.20<br>–<br>–<br>–<br>–<br>–	26.20<br>26.82<br>26.27<br>–<br>–<br>–<br>–<br>–	26.28<br>26.88<br>26.34<br>–<br>–<br>–<br>–<br>–	26.36<br>26.94<br>26.39<br>–<br>–<br>–<br>–<br>–	26.42<br>26.98<br>26.44<br>–<br>–<br>–<br>–<br>–	26.47<br>27.02<br>26.47<br>–<br>–<br>–<br>–<br>–	26.51<br>27.03<br>26.51<br>–<br>–<br>–<br>–<br>–	26.54<br>27.05<br>26.54<br>–<br>–<br>–<br>–<br>–	26.55<br>27.06<br>26.56<br>–<br>–<br>–<br>–<br>–	26.56<br>27.06<br>26.53<br>–<br>–<br>–<br>–<br>–	26.56<br>27.05<br>26.52<br>–<br>–<br>–<br>–<br>–	26.56<br>27.03<br>26.50<br>–<br>–<br>–<br>–<br>–
Cu_{13} ring, pz-3,5-H_2	21.21<br>21.67<br>21.22<br>–<br>–<br>–<br>–<br>–	21.27<br>21.70<br>21.27<br>–<br>–<br>–<br>–<br>–	21.33<br>21.75<br>21.34<br>–<br>–<br>–<br>–<br>–	21.39<br>21.79<br>21.39<br>–<br>–<br>–<br>–<br>–	21.43<br>21.84<br>21.44<br>–<br>–<br>–<br>–<br>–	21.47<br>21.85<br>21.48<br>–<br>–<br>–<br>–<br>–	21.50<br>21.87<br>21.51<br>–<br>–<br>–<br>–<br>–	21.53<br>21.89<br>21.53<br>–<br>–<br>–<br>–<br>–	21.54<br>21.89<br>21.54<br>–<br>–<br>–<br>–<br>–	21.55<br>21.89<br>21.55<br>–<br>–<br>–<br>–<br>–	21.54<br>21.87<br>21.55<br>–<br>–<br>–<br>–<br>–	21.54<br>21.85<br>21.54<br>–<br>–<br>–<br>–<br>–	21.52<br>21.82<br>21.52<br>–<br>–<br>–<br>–<br>–	21.50<br>21.81<br>21.51<br>–<br>–<br>–<br>–<br>–
Cu_{13} ring, O*H*	−26.70<br>−28.57<br>–<br>–<br>–<br>–<br>–<br>–	−26.91<br>−28.65<br>–<br>–<br>–<br>–<br>–<br>–	−27.14<br>−28.86<br>–<br>–<br>–<br>–<br>–<br>–	−27.34<br>−29.08<br>–<br>–<br>–<br>–<br>–<br>–	−27.53<br>−29.18<br>–<br>–<br>–<br>–<br>–<br>–	−27.74<br>−29.34<br>–<br>–<br>–<br>–<br>–<br>–	−27.92<br>−29.49<br>–<br>–<br>–<br>–<br>–<br>–	−28.10<br>−29.60<br>–<br>–<br>–<br>–<br>–<br>–	−28.25<br>−29.69<br>–<br>–<br>–<br>–<br>–<br>–	−28.39<br>−29.76<br>–<br>–<br>–<br>–<br>–<br>–	−28.54<br>−29.89<br>–<br>–<br>–<br>–<br>–<br>–	−28.65<br>−29.93<br>–<br>–<br>–<br>–<br>–<br>–	−28.78<br>−29.99<br>–<br>–<br>–<br>–<br>–<br>–	−28.86<br>−30.07<br>–<br>–<br>–<br>–<br>–<br>–
Cu_8 ring, O*H*	−33.61<br>−38.88<br>–<br>–<br>–<br>–<br>–<br>–	−33.34<br>−38.57<br>–<br>–<br>–<br>–<br>–<br>–	−33.05<br>−38.37<br>–<br>–<br>–<br>–<br>–<br>–	−32.81<br>−38.01<br>–<br>–<br>–<br>–<br>–<br>–	−32.53<br>−37.76<br>–<br>–<br>–<br>–<br>–<br>–	−32.29<br>−37.56<br>–<br>–<br>–<br>–<br>–<br>–	−32.05<br>−37.31<br>–<br>–<br>–<br>–<br>–<br>–	−31.83<br>−37.11<br>–<br>–<br>–<br>–<br>–<br>–	−31.63<br>−36.86<br>–<br>–<br>–<br>–<br>–<br>–	−31.44<br>−36.73<br>–<br>–<br>–<br>–<br>–<br>–	−31.27<br>−36.52<br>–<br>–<br>–<br>–<br>–<br>–	−31.10<br>−36.37<br>–<br>–<br>–<br>–<br>–<br>–	−30.96<br>−36.04<br>–<br>–<br>–<br>–<br>–<br>–	−30.81<br>−35.11<br>–<br>–<br>–<br>–<br>–<br>–

Table S54. (continued)

NANOJAR	20–23 °C	30 °C	40 °C	50 °C	60 °C	70 °C	80 °C	90 °C	100 °C	110 °C	120 °C	130 °C	140 °C	150 °C
**Cu_{31} (8+14+9)**														
Cu_9 ring, pz-4-*H*	–<br>32.95<br>–<br>–<br>–<br>–<br>–<br>–	–<br>32.79<br>–<br>–<br>–<br>–<br>–<br>–	33.86<br>32.62<br>29.88<br>–<br>–<br>–<br>–<br>–	33.64<br>32.39<br>29.85<br>–<br>–<br>–<br>–<br>–	33.43<br>32.19<br>29.83<br>–<br>–<br>–<br>–<br>–	33.21<br>31.99<br>29.81<br>–<br>–<br>–<br>–<br>–	33.00<br>31.77<br>29.78<br>–<br>–<br>–<br>–<br>–	32.78<br>31.60<br>29.74<br>–<br>–<br>–<br>–<br>–	32.56<br>31.40<br>29.69<br>–<br>–<br>–<br>–<br>–	32.34<br>31.21<br>29.64<br>–<br>–<br>–<br>–<br>–	32.13<br>31.03<br>29.58<br>–<br>–<br>–<br>–<br>–	31.92<br>30.86<br>29.51<br>–<br>–<br>–<br>–<br>–	–<br>30.70<br>29.44<br>–<br>–<br>–<br>–<br>–	–<br>30.51<br>29.37<br>–<br>–<br>–<br>–<br>–
Cu_9 ring, pz-3,5-H_2	–<br>28.73<br>–<br>–<br>–<br>–<br>–<br>–	–<br>28.76<br>–<br>–<br>–<br>–<br>–<br>–	~29.3<br>28.57<br>–<br>–<br>–<br>–<br>–<br>–	~29.1<br>28.40<br>–<br>–<br>–<br>–<br>–<br>–	~29.0<br>28.25<br>–<br>–<br>–<br>–<br>–<br>–	~28.8<br>28.10<br>–<br>–<br>–<br>–<br>–<br>–	~28.6<br>27.93<br>26.15<br>–<br>–<br>–<br>–<br>–	~28.3<br>27.78<br>29.12<br>–<br>–<br>–<br>–<br>–	~28.1<br>27.63<br>26.08<br>–<br>–<br>–<br>–<br>–	~28.0<br>27.49<br>26.03<br>–<br>–<br>–<br>–<br>–	~27.8<br>27.33<br>25.97<br>–<br>–<br>–<br>–<br>–	27.57<br>27.20<br>25.91<br>–<br>–<br>–<br>–<br>–	–<br>27.06<br>25.84<br>–<br>–<br>–<br>–<br>–	–<br>26.91<br>25.77<br>–<br>–<br>–<br>–<br>–
Cu_8 ring, pz-4-*H*	–<br>30.16<br>–<br>–<br>–<br>–<br>–<br>–	–<br>30.18<br>–<br>–<br>–<br>–<br>–<br>–	~30.9<br>30.19<br>29.50<br>–<br>–<br>–<br>–<br>–	~30.85<br>30.19<br>29.39<br>–<br>–<br>–<br>–<br>–	30.75<br>30.19<br>29.30<br>–<br>–<br>–<br>–<br>–	30.66<br>30.16<br>29.22<br>–<br>–<br>–<br>–<br>–	30.58<br>30.12<br>29.13<br>–<br>–<br>–<br>–<br>–	~30.5<br>30.08<br>29.04<br>–<br>–<br>–<br>–<br>–	~30.4<br>30.04<br>28.95<br>–<br>–<br>–<br>–<br>–	30.30<br>29.99<br>28.86<br>–<br>–<br>–<br>–<br>–	30.20<br>29.93<br>28.76<br>–<br>–<br>–<br>–<br>–	30.11<br>29.85<br>28.65<br>–<br>–<br>–<br>–<br>–	–<br>29.79<br>28.56<br>–<br>–<br>–<br>–<br>–	–<br>29.72<br>28.45<br>–<br>–<br>–<br>–<br>–
Cu_8 ring, pz-3,5-H_2	–<br>26.83<br>–<br>–<br>–<br>–<br>–<br>–	–<br>26.86<br>–<br>–<br>–<br>–<br>–<br>–	~27.1<br>26.88<br>–<br>–<br>–<br>–<br>–<br>–	27.09<br>26.88<br>–<br>–<br>–<br>–<br>–<br>–	27.02<br>26.88<br>26.00<br>–<br>–<br>–<br>–<br>–	26.96<br>26.86<br>25.93<br>–<br>–<br>–<br>–<br>–	26.89<br>26.83<br>25.86<br>–<br>–<br>–<br>–<br>–	26.82<br>26.78<br>25.79<br>–<br>–<br>–<br>–<br>–	26.74<br>26.74<br>25.71<br>–<br>–<br>–<br>–<br>–	~26.6<br>26.69<br>25.63<br>–<br>–<br>–<br>–<br>–	~26.5<br>26.63<br>25.54<br>–<br>–<br>–<br>–<br>–	~26.5<br>26.57<br>25.44<br>–<br>–<br>–<br>–<br>–	–<br>26.51<br>25.35<br>–<br>–<br>–<br>–<br>–	–<br>26.44<br>25.25<br>–<br>–<br>–<br>–<br>–
Cu_{14} ring, pz-4-*H*	28.24<br>28.73<br>27.95<br>–<br>–<br>–<br>–<br>–	28.30<br>28.76<br>27.99<br>–<br>–<br>–<br>–<br>–	28.36<br>28.80<br>28.05<br>–<br>–<br>–<br>–<br>–	28.41<br>28.85<br>~28.1<br>–<br>–<br>–<br>–<br>–	28.44<br>28.88<br>~28.0<br>–<br>–<br>–<br>–<br>–	28.46<br>28.89<br>28.18<br>–<br>–<br>–<br>–<br>–	28.48<br>28.91<br>28.20<br>–<br>–<br>–<br>–<br>–	28.48<br>28.89<br>28.21<br>–<br>–<br>–<br>–<br>–	28.47<br>28.88<br>28.21<br>–<br>–<br>–<br>–<br>–	28.45<br>28.86<br>28.19<br>–<br>–<br>–<br>–<br>–	28.43<br>28.83<br>28.17<br>–<br>–<br>–<br>–<br>–	28.40<br>28.78<br>28.14<br>–<br>–<br>–<br>–<br>–	–<br>28.73<br>28.10<br>–<br>–<br>–<br>–<br>–	–<br>28.67<br>28.05<br>–<br>–<br>–<br>–<br>–
Cu_{14} ring, pz-3,5-H_2	22.66<br>22.91<br>22.34<br>–<br>–<br>–<br>–<br>–	22.68<br>22.93<br>22.37<br>–<br>–<br>–<br>–<br>–	22.70<br>22.94<br>22.39<br>–<br>–<br>–<br>–<br>–	22.71<br>22.94<br>22.41<br>–<br>–<br>–<br>–<br>–	22.71<br>22.95<br>22.43<br>–<br>–<br>–<br>–<br>–	22.70<br>22.94<br>22.43<br>–<br>–<br>–<br>–<br>–	22.69<br>22.92<br>22.42<br>–<br>–<br>–<br>–<br>–	22.67<br>22.91<br>22.41<br>–<br>–<br>–<br>–<br>–	22.65<br>22.88<br>22.39<br>–<br>–<br>–<br>–<br>–	22.61<br>22.85<br>22.36<br>–<br>–<br>–<br>–<br>–	22.57<br>22.80<br>22.33<br>–<br>–<br>–<br>–<br>–	22.53<br>22.76<br>22.29<br>–<br>–<br>–<br>–<br>–	–<br>22.71<br>22.24<br>–<br>–<br>–<br>–<br>–	–<br>22.66<br>22.19<br>–<br>–<br>–<br>–<br>–
Cu_{14} ring, O*H*	–<br>−28.28<br>–<br>–<br>–<br>–<br>–<br>–	–<br>−28.02<br>−25.16<br>–<br>–<br>–<br>–<br>–	–<br>−27.93<br>−25.00<br>–<br>–<br>–<br>–<br>–	–<br>−27.95<br>−24.96<br>–<br>–<br>–<br>–<br>–	–<br>−27.81<br>−24.93<br>–<br>–<br>–<br>–<br>–	–<br>−27.76<br>−24.92<br>–<br>–<br>–<br>–<br>–	–<br>−27.72<br>−24.91<br>–<br>–<br>–<br>–<br>–	–<br>−27.63<br>−24.89<br>–<br>–<br>–<br>–<br>–	–<br>−27.53<br>−24.85<br>–<br>–<br>–<br>–<br>–	–<br>−27.53<br>−24.80<br>–<br>–<br>–<br>–<br>–	–<br>−27.33<br>−24.73<br>–<br>–<br>–<br>–<br>–	–<br>−27.23<br>−24.66<br>–<br>–<br>–<br>–<br>–	–<br>−27.09<br>−24.58<br>–<br>–<br>–<br>–<br>–	–<br>−26.97<br>−24.48<br>–<br>–<br>–<br>–<br>–
Cu_8 ring, O*H*	–<br>–<br>–<br>–<br>–<br>–<br>–<br>–	–<br>–<br>–<br>–<br>–<br>–<br>–<br>–	–<br>–<br>–<br>–<br>–<br>–<br>–<br>–	–<br>–<br>–<br>–<br>–<br>–<br>–<br>–	–<br>−35.72<br>–<br>–<br>–<br>–<br>–<br>–	–<br>−35.67<br>–<br>–<br>–<br>–<br>–<br>–	–<br>−35.61<br>~−32.2<br>–<br>–<br>–<br>–<br>–	–<br>−35.53<br>~−32.0<br>–<br>–<br>–<br>–<br>–	–<br>−35.47<br>−31.88<br>–<br>–<br>–<br>–<br>–	–<br>−35.47<br>−31.78<br>–<br>–<br>–<br>–<br>–	–<br>−35.27<br>−31.68<br>–<br>–<br>–<br>–<br>–	–<br>−35.20<br>−31.56<br>–<br>–<br>–<br>–<br>–	–<br>−35.06<br>−31.47<br>–<br>–<br>–<br>–<br>–	–<br>−35.00<br>−31.38<br>–<br>–<br>–<br>–<br>–
Cu_9 ring, O*H*	–<br>–<br>–<br>–<br>–<br>–<br>–<br>–	–<br>–<br>–<br>–<br>–<br>–<br>–<br>–	–<br>−41.73<br>–<br>–<br>–<br>–<br>–<br>–	–<br>−40.19<br>–<br>–<br>–<br>–<br>–<br>–	–<br>−39.47<br>–<br>–<br>–<br>–<br>–<br>–	–<br>−38.89<br>–<br>–<br>–<br>–<br>–<br>–	–<br>−38.36<br>−34.31<br>–<br>–<br>–<br>–<br>–	–<br>−37.85<br>−33.86<br>–<br>–<br>–<br>–<br>–	–<br>−37.37<br>−33.48<br>–<br>–<br>–<br>–<br>–	–<br>−36.92<br>−33.07<br>–<br>–<br>–<br>–<br>–	–<br>−36.53<br>−32.68<br>–<br>–<br>–<br>–<br>–	–<br>−36.16<br>−32.31<br>–<br>–<br>–<br>–<br>–	–<br>−35.81<br>−31.94<br>–<br>–<br>–<br>–<br>–	–<br>−35.41<br>−31.55<br>–<br>–<br>–<br>–<br>–

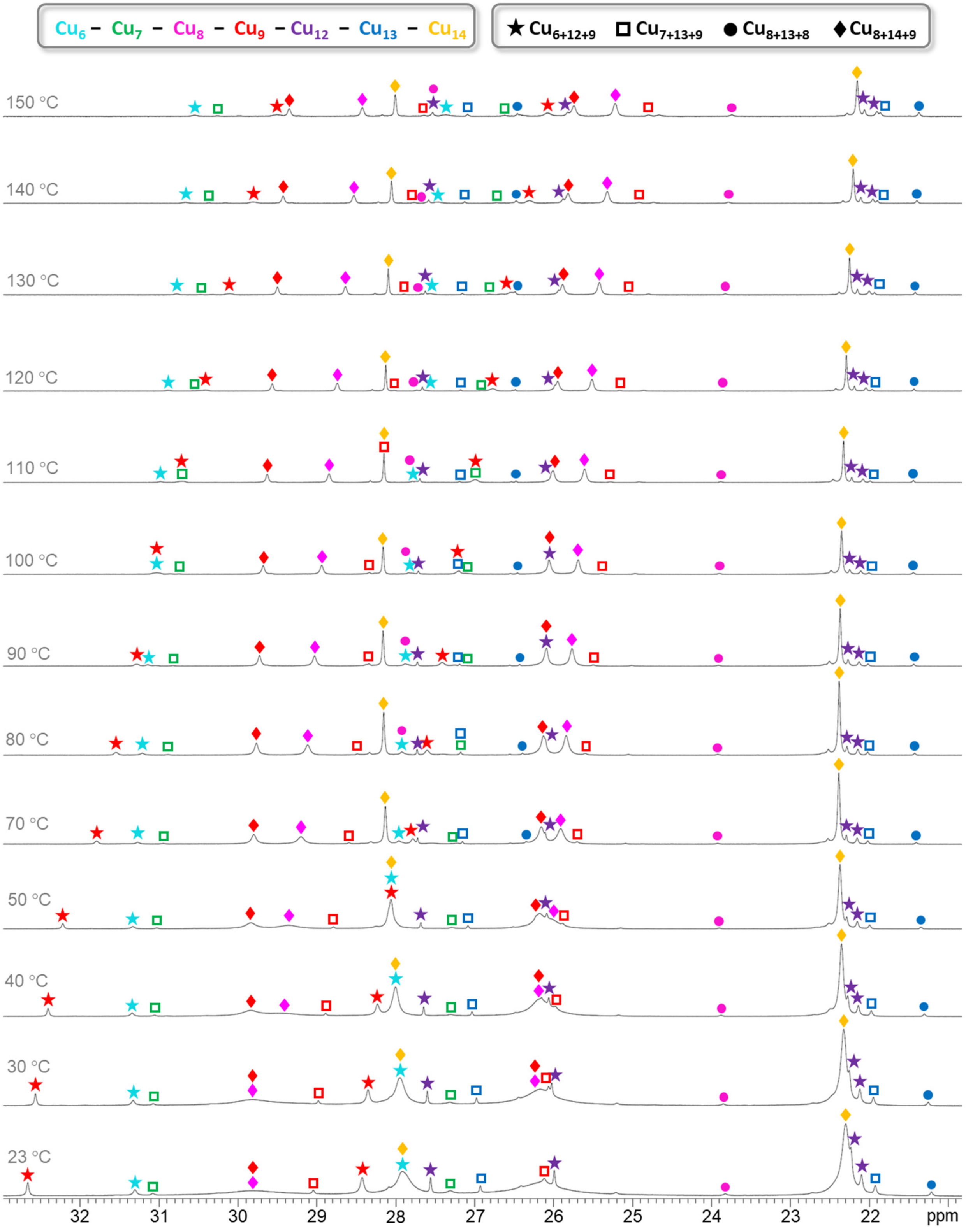

Figure S20. Variable-temperature ^1H NMR spectra in DMSO-d_6 of a Cu_nMePO_3 (n = 27−31) nanojar mixture consisting mostly of $Cu_{31}MePO_3$, showing pyrazolate proton signals in the 21−33 ppm window. The temperatures shown are the target temperatures of the probe.

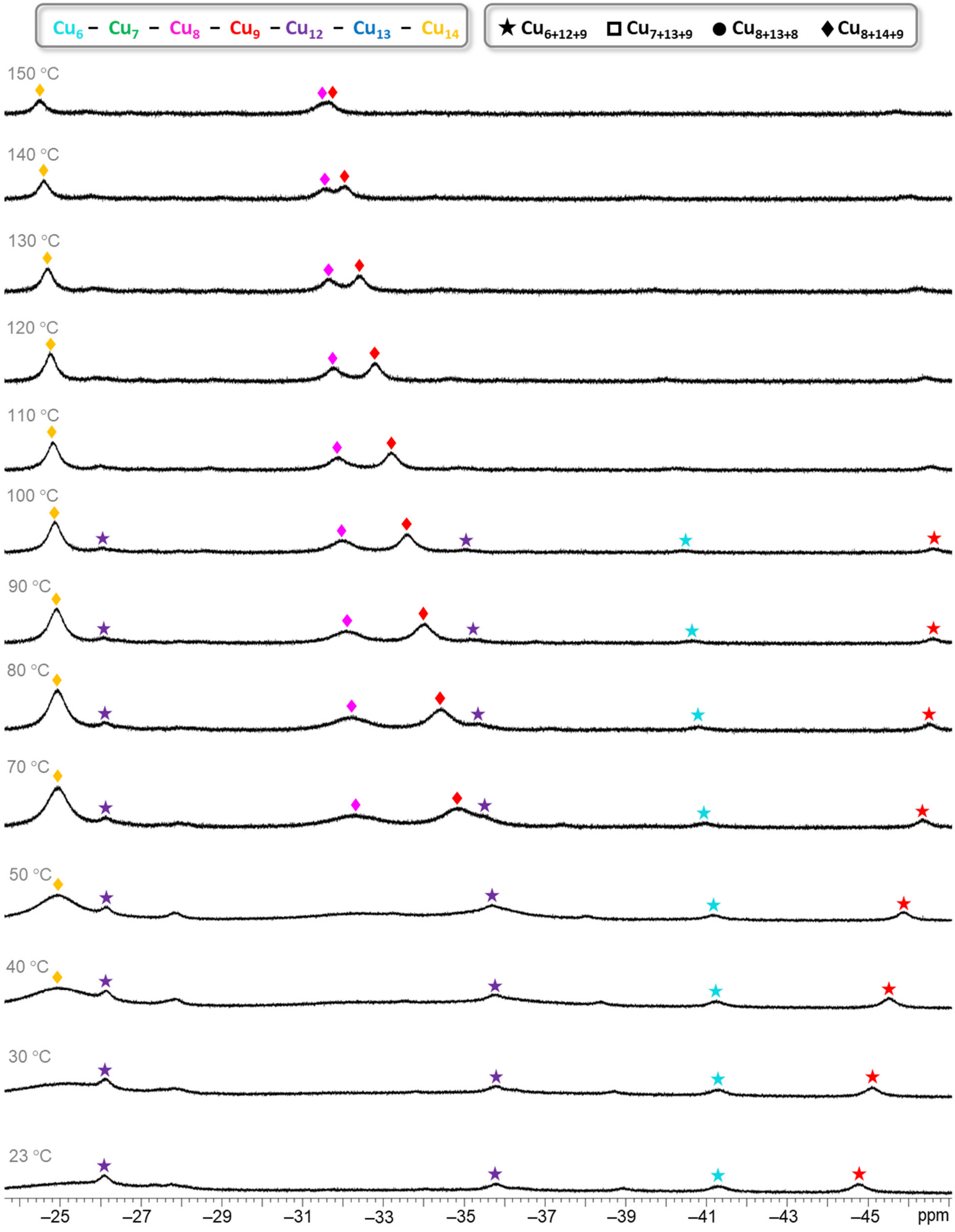

Figure S21. Variable-temperature ^1H NMR spectra in DMSO-d_6 of a Cu_nMePO_3 (n = 27−31) nanojar mixture consisting mostly of $Cu_{31}MePO_3$, showing OH proton signals in the (−24)–(−47) ppm window. The temperatures shown are the target temperatures of the probe.

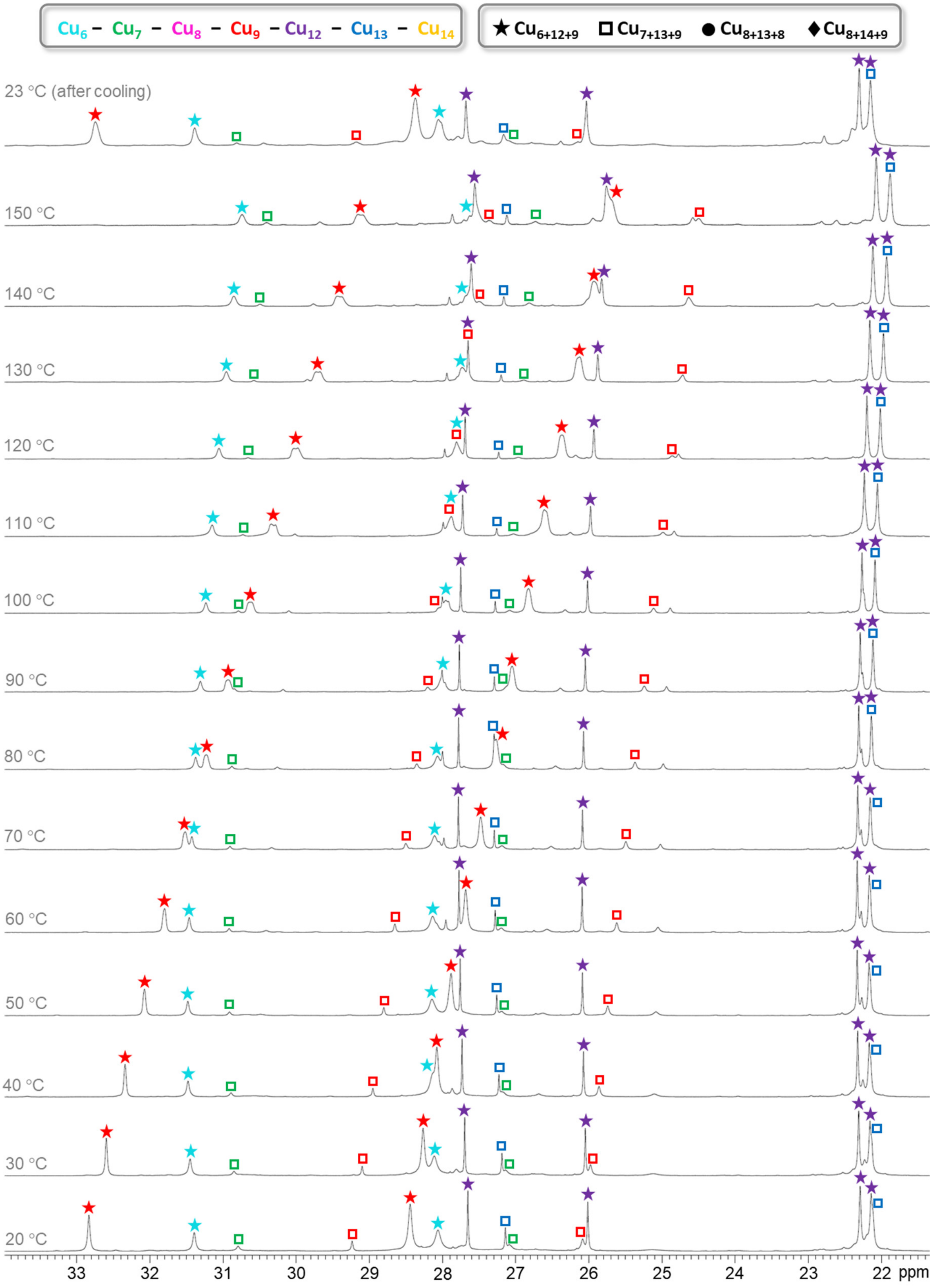

Figure S22. Variable-temperature ^{1}H NMR spectra of the Cu_nEtPO_3 (n = 27–31) nanojar mixture in DMSO-d_6, showing pyrazolate proton signals in the 21–33 ppm window. The temperatures shown are the target temperatures of the probe.

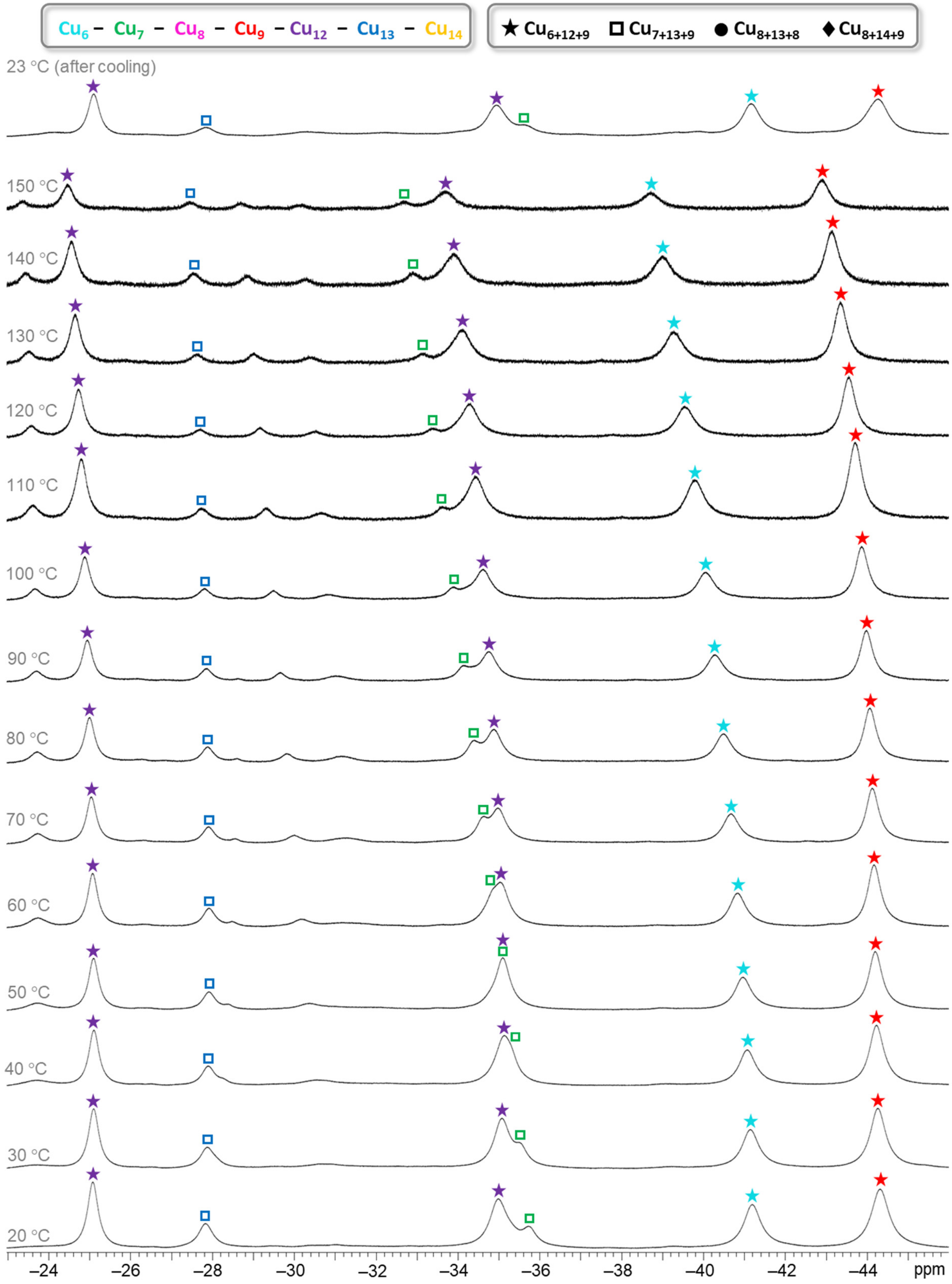

Figure S23. Variable-temperature ^{1}H NMR spectra of the Cu_nEtPO_3 (n = 27−31) nanojar mixture in DMSO-d_6, showing OH proton signals in the (−24)−(−45) ppm window. The given temperatures are the target temperatures of the probe.

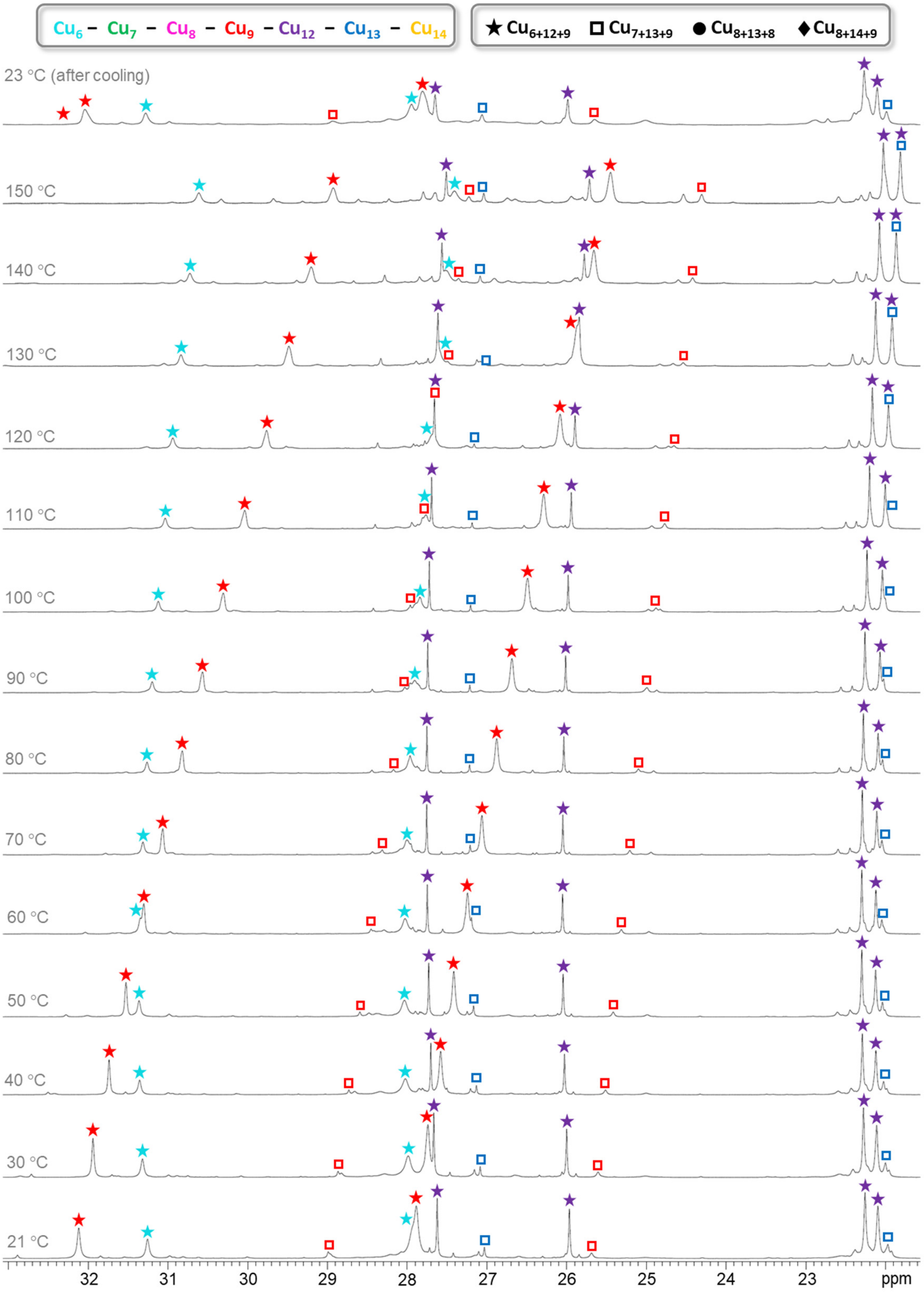

Figure S24. Variable-temperature ^{1}H NMR spectra of the $Cu_n{}^{n}PrPO_3$ (n = 27−31) nanojar mixture in DMSO-d_6, showing pyrazolate proton signals in the 21−33 ppm window. The temperatures shown are the target temperatures of the probe.

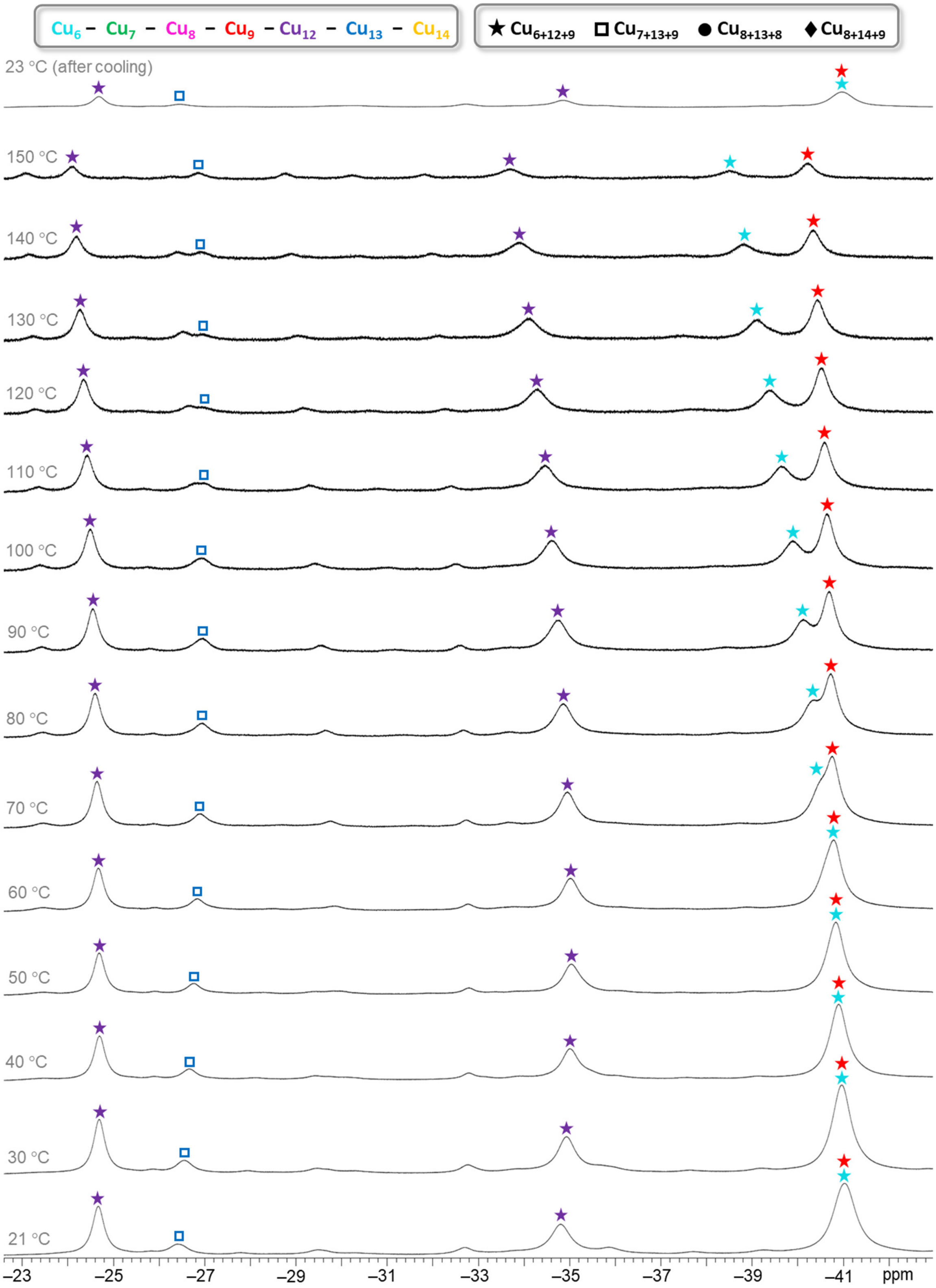

Figure S25. Variable-temperature ^{1}H NMR spectra of the $Cu_n{}^{n}PrPO_3$ (n = 27−31) nanojar mixture in DMSO-d_6, showing OH proton signals in the (−24)−(−42) ppm window. The given temperatures are the target temperatures of the probe.

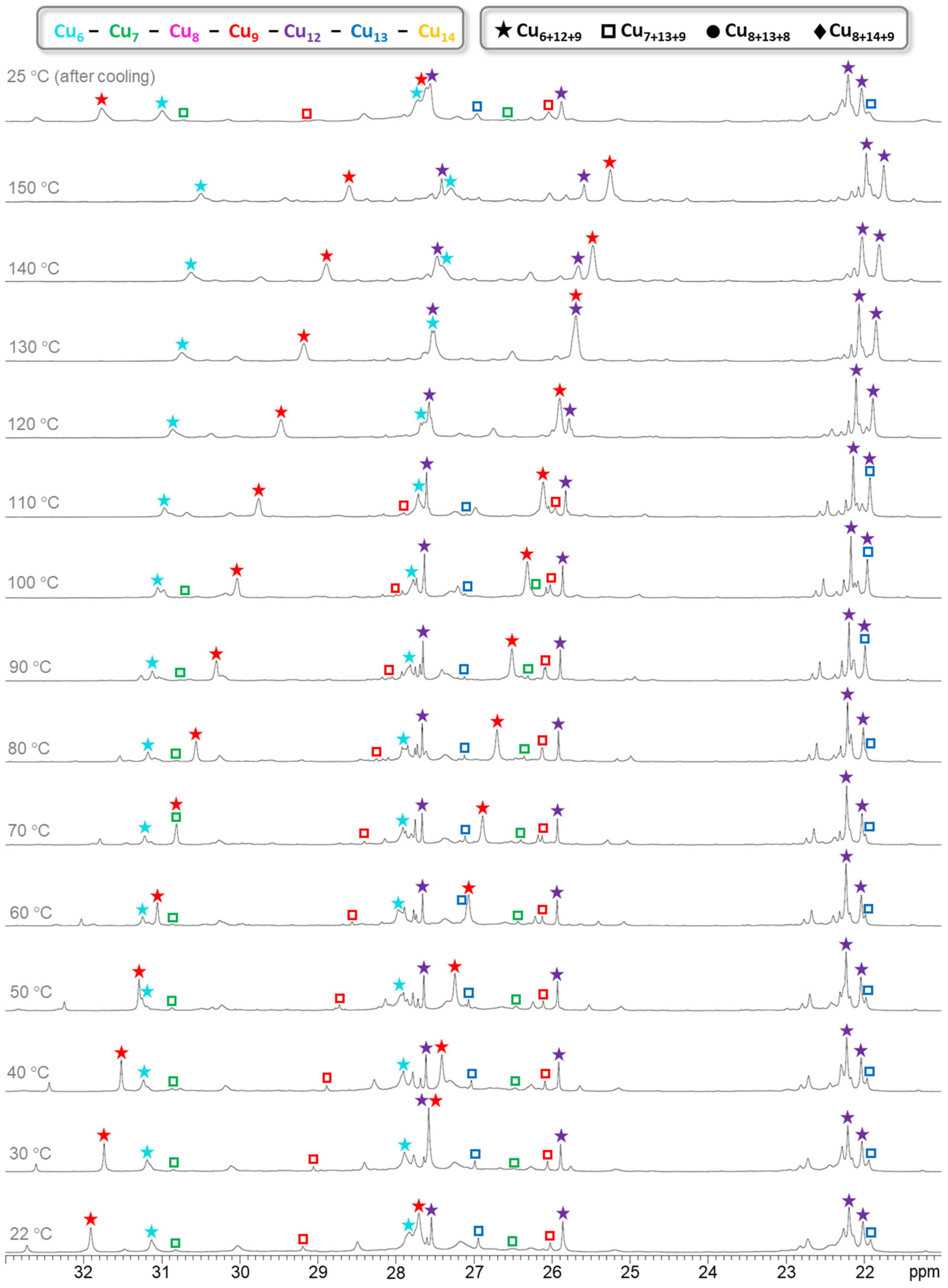

Figure S26. Variable-temperature ^{1}H NMR spectra of the $Cu_n{}^nBuPO_3$ (n = 27−31) nanojar mixture in DMSO-d_6, showing pyrazolate proton signals in the 21−33 ppm window. The temperatures shown are the target temperatures of the probe.

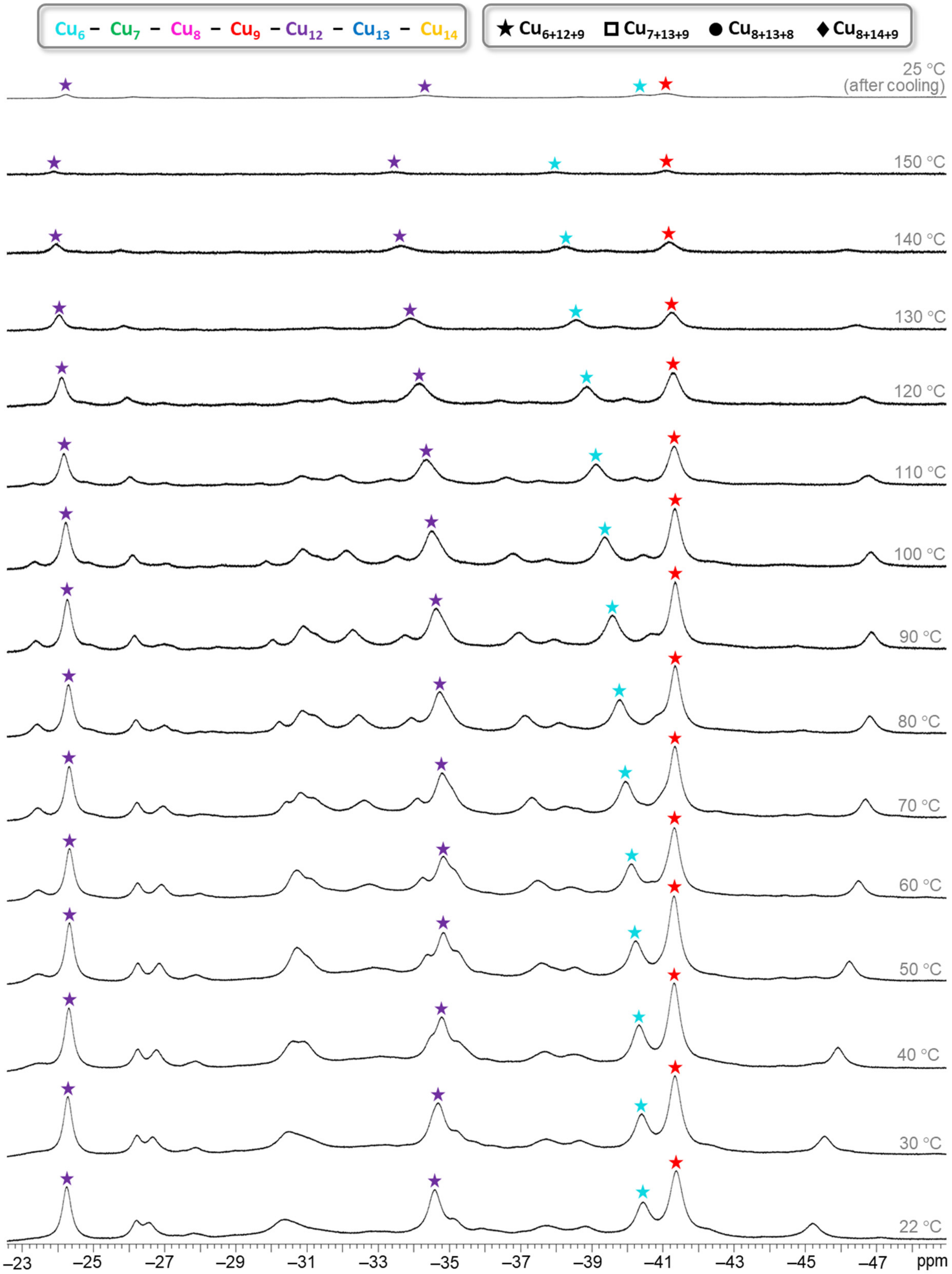

Figure S27. Variable-temperature ^1H NMR spectra of the $Cu_n{}^nBuPO_3$ (n = 27−31) nanojar mixture in DMSO-d_6, showing OH proton signals in the (−23)−(−48) ppm window. The given temperatures are the target temperatures of the probe.

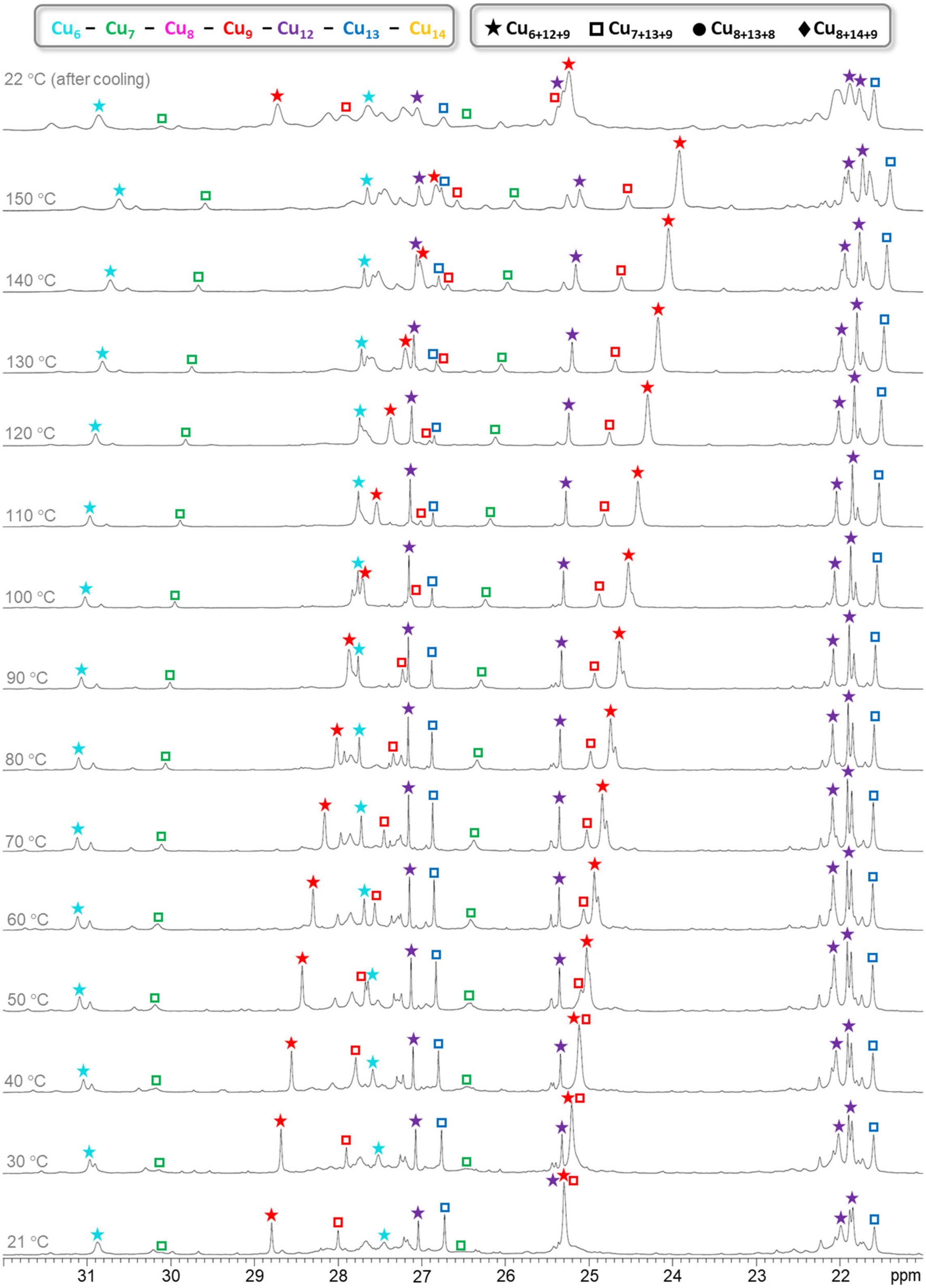

Figure S28. Variable-temperature ^1H NMR spectra of the Cu_nBnPO_3 (n = 27−31) nanojar mixture in DMSO-d_6, showing pyrazolate proton signals in the 21−32 ppm window. The temperatures shown are the target temperatures of the probe.

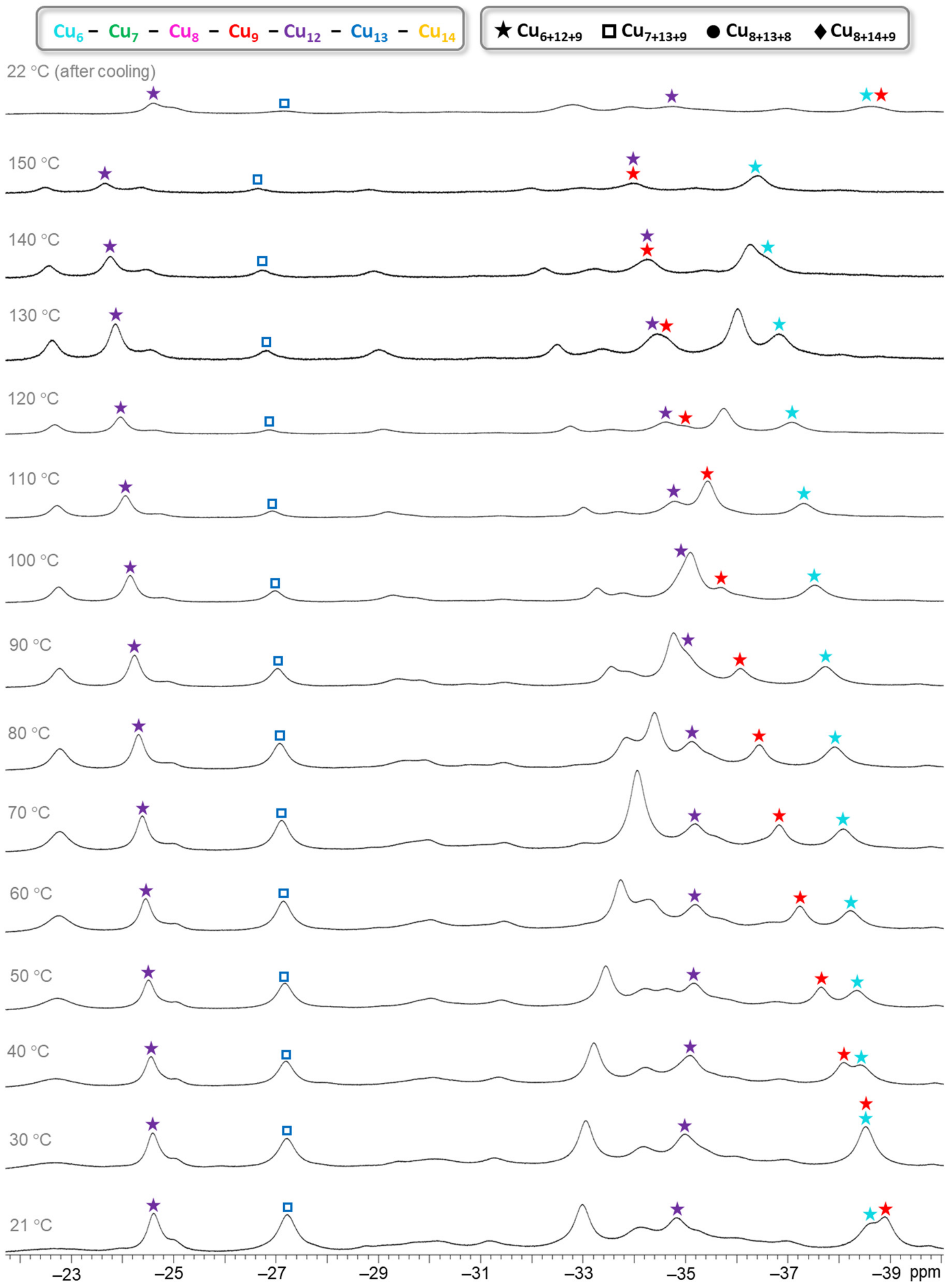

Figure S29. Variable-temperature ^{1}H NMR spectra of the Cu_nBnPO_3 (n = 27−31) nanojar mixture in DMSO-d_6, showing OH proton signals in the (−22)−(−40) ppm window. The given temperatures are the target temperatures of the probe.

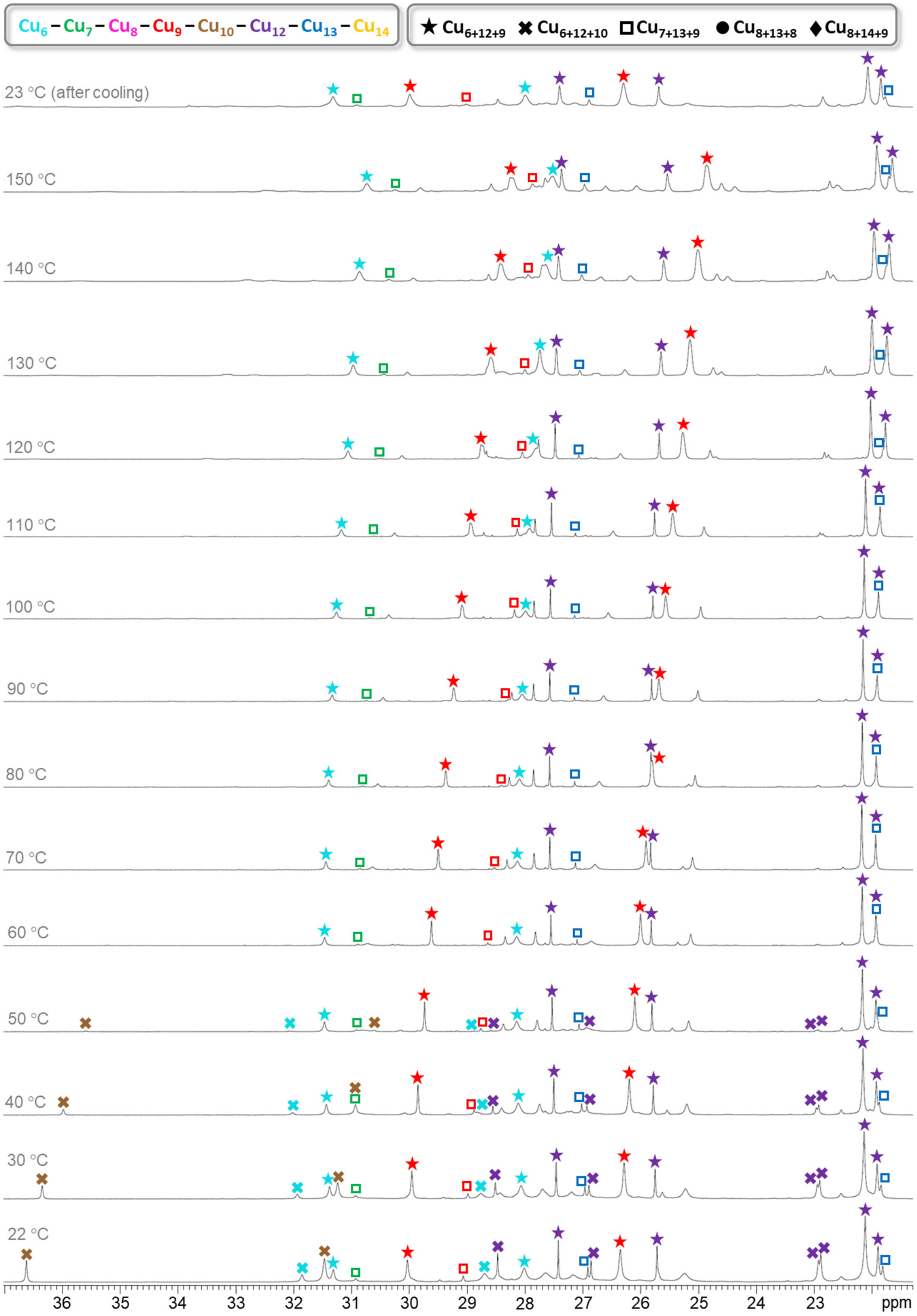

Figure S30. Variable-temperature ^1H NMR spectra of the Cu_nPhPO_3 (n = 27−31) nanojar mixture in DMSO-d_6, showing pyrazolate proton signals in the 21−37 ppm window. The temperatures shown are the target temperatures of the probe.

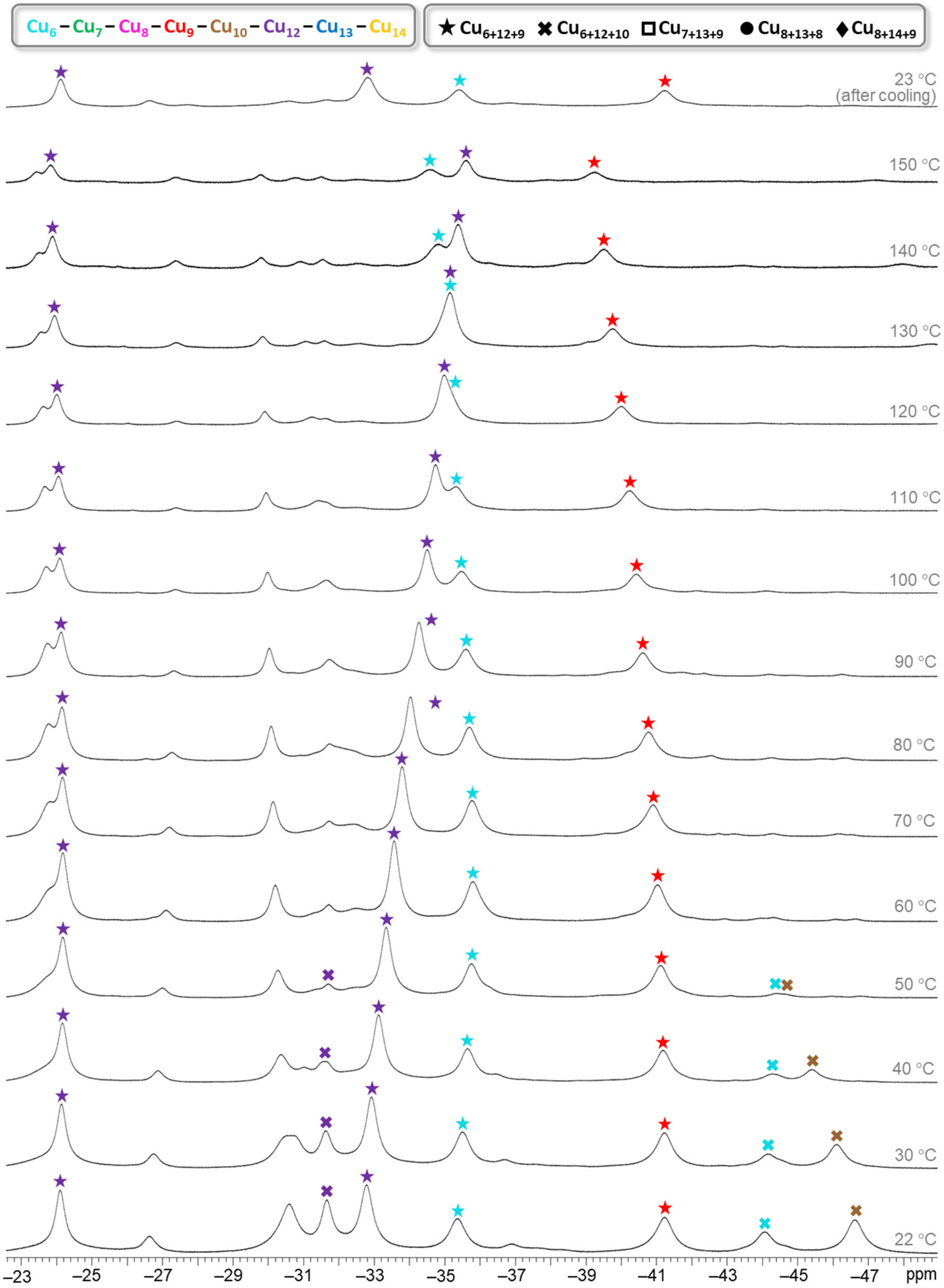

Figure S31. Variable-temperature ^{1}H NMR spectra of the Cu_nPhPO_3 (n = 27−31) nanojar mixture in DMSO-d_6, showing OH proton signals in the (−24)−(−48) ppm window. The given temperatures are the target temperatures of the probe.

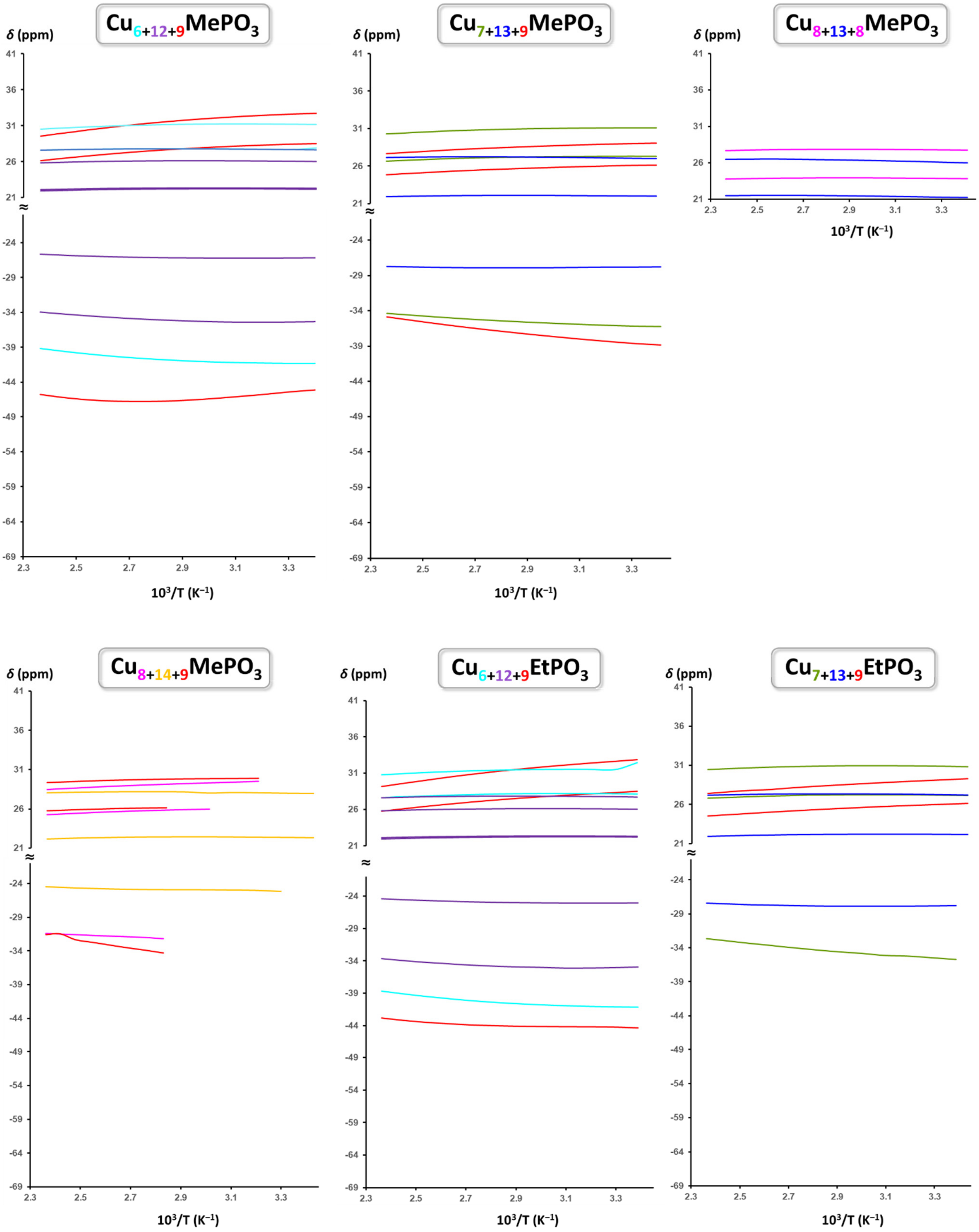

Figure S32. Curie plots for different Cu_x ring protons in the Cu_nMePO_3 and Cu_nEtPO_3 nanojars in DMSO-d_6, illustrating the influence of the size of the ring on chemical shift, as well as the difference in chemical shift for the same Cu_x-ring in different nanojars.

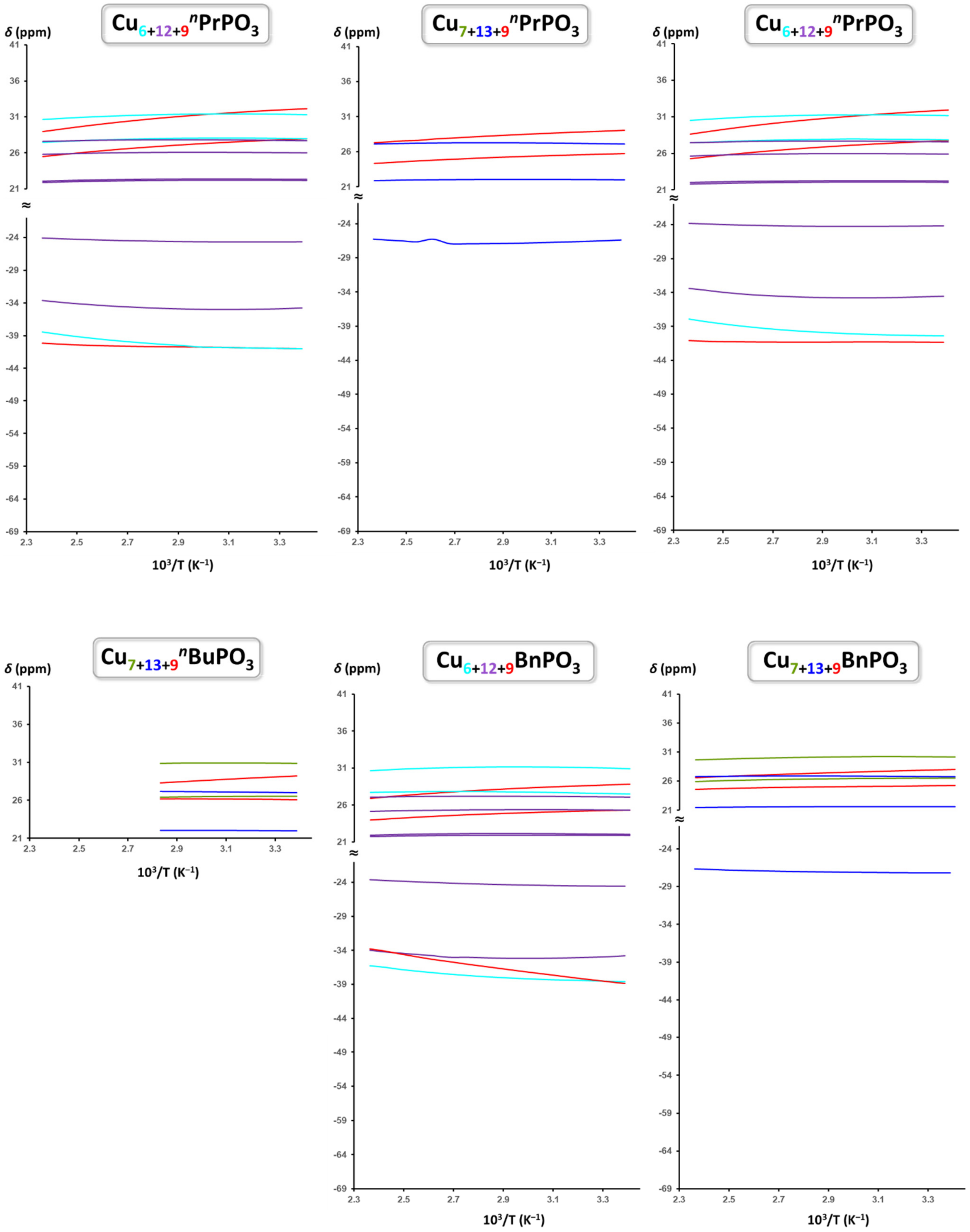

Figure S33. Curie plots for different Cu_x ring protons in the $Cu_n{}^{n}PrPO_3$, $Cu_n{}^{n}BuPO_3$ and Cu_nBnPO_3 nanojars in DMSO-d_6, illustrating the influence of the size of the ring on chemical shift, as well as the difference in chemical shift for the same Cu_x-ring in different nanojars.

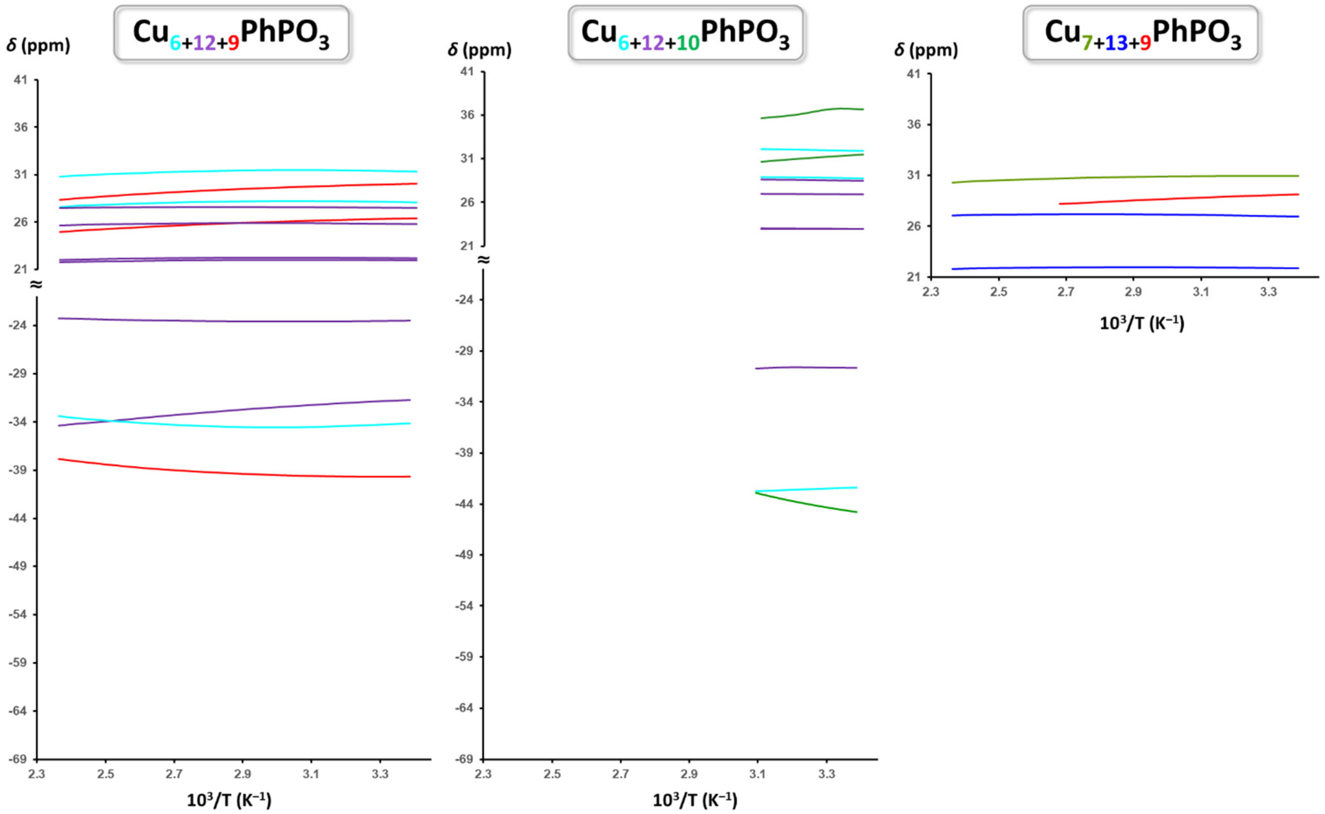

Figure S34. Curie plots for different Cu_x ring protons in the Cu_nPhPO_3 nanojars in DMSO-d_6, illustrating the influence of the size of the ring on chemical shift, as well as the difference in chemical shift for the same Cu_x-ring in different nanojars.

4. REFERENCES

[1] van der Sluis, P.; Spek, A. L. BYPASS: An Effective Method for the Refinement of Crystal Structures Containing Disordered Solvent Regions. *Acta Crystallogr., Sect. A: Found. Crystallogr.* **1990**, *A46*, 194–201.
